# Developing multitarget coumarin based anti-breast cancer agents: synthesis and molecular modeling study

**DOI:** 10.1038/s41598-023-40232-3

**Published:** 2023-08-17

**Authors:** Fiby N. Takla, Waleed A. Bayoumi, Shahenda M. El-Messery, Magda N. A. Nasr

**Affiliations:** 1 Department of Pharmaceutical Organic Chemistry, Faculty of Pharmacy, Mansoura University, Mansoura, 35516 Egypt; 2 Department of Pharmaceutical Chemistry, Faculty of Pharmacy, Delta University for Science and Technology, International Coastal Road, Gamasa City, 35712 Egypt

**Keywords:** Biochemistry, Cancer, Chemical biology, Computational biology and bioinformatics, Drug discovery, Diseases, Health care, Chemistry

## Abstract

A new series of 7-substituted coumarin scaffolds containing a methyl ester moiety at the C^4^-position were synthesized and tested for their in vitro anti-proliferative activity against MCF-7 and MDA-MB-231 breast cancer cell lines using Doxorubicin (DOX) as reference. Compounds **2** and **8** showed noticeable selectivity against MCF-7 with IC_50_ = 6.0 and 5.8 µM, respectively compared to DOX with IC_50_ = 5.6 µM. Compounds **10**, **12,** and **14** exhibited considerable selectivity against Estrogen Negative cells with IC_50_ = 2.3, 3.5, and 1.9 µM, respectively) compared to DOX with (IC_50_ = 7.3 µM). The most promising compounds were tested as epidermal growth factor receptor and aromatase (ARO) enzymes inhibitors using erlotinib and exemestane (EXM) as standards, respectively. Results proved that compound **8** elicited the highest inhibitory activity (94.73% of the potency of EXM), while compounds **10** and **12** displayed 97.67% and 81.92% of the potency of Erlotinib, respectively. Further investigation showed that the promising candidates** 8**, **10**, and **12** caused cell cycle arrest at G0–G1 and S phases and induced apoptosis. The mechanistic pathway was confirmed by elevating caspases-9 and Bax/Bcl-2 ratio. A set of in silico methods was also performed including docking, bioavailability ADMET screening and QSAR study

## Introduction

One of the prominent causes of cancer mortality in women is breast cancer (BC), the second-most common cancer^1^. About 75% of BC is caused by the estrogen receptor (ER)^2^.

Inhibition of estrogen biosynthesis is one effective approach for hormone-dependent BC therapy in postmenopausal women^3^. The aromatase enzyme (ARO) is one of the enzymatic mechanisms that may regulate the increased estrogen levels found in BC cells. Exemestane (EXM) (**Ia**) is among the reported powerful aromatase inhibitors (AIs)^4^.

On the other hand, the most often overexpressed receptors in BC cells are tyrosine kinase receptors (TKRs), e.g., the epidermal growth factor receptor (EGFR)^5^. Thus, targeting those receptors is a promising way to develop novel anticancer drugs. In this context, the first reported EGFR inhibitor highly expressed in different forms of cancers is Erlotinib (**Ib**)^6,7^.

Moreover, targeting various apoptotic pathways is also an effective approach for all types of cancer^8,9^. Any pathway stage can be targeted for cancer treatment^10^.

In light of the above information, developing and synthesizing novel anticancer candidates with increased selectivity is essential. The varied biological characteristics of coumarin-containing drugs, especially anticancer activity, have generated much interest^11–13^. Investigations into the biological properties of coumarins have found that they can target multiple cancer pathways, including aromatase inhibition, kinase inhibition, cell cycle arrest, and angiogenesis suppression^14,15^.

**Figure 1:**
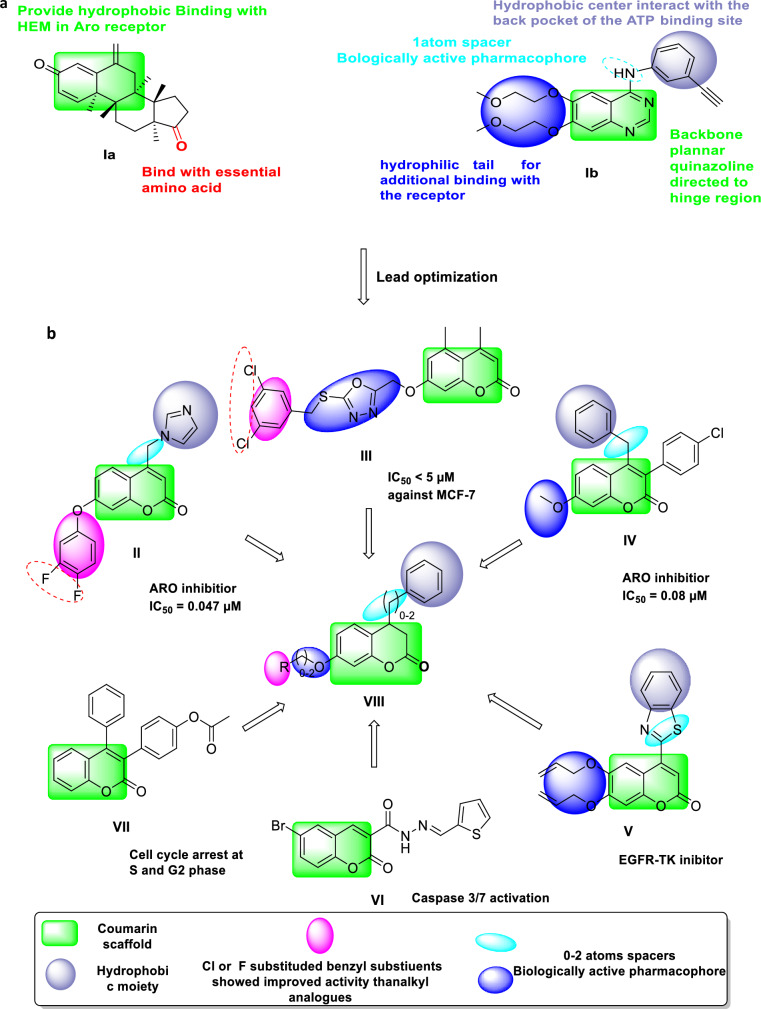
(**a)** Main structural features of EXM (**Ia**) and Erlotinib (**Ib**). **(b)** Design chart with reported coumarin derivatives as a potent anti-breast cancer agent.

Studying the structures of EXM (**Ia**)^16,17^ and erlotinib (**Ib**)^18^ (Fig. 1a) has provided important structural information which helped us in the rational design of new potent ARO/EGFR inhibitors.

Importantly, various anticancer candidates based upon coumarin skeletons (**II-VII**) and their structural characteristics were selected as the main pharmacophore in developing multitargeted anti-BC^19–23^. In this work, the molecular design was mostly based on two strategies:

In the first strategy, based on the previous evidence, we have selected EXM (**Ia**) and Erlotinib (**Ib**) as lead anti-BC compounds (Fig. 1a). Secondly, structural optimization of the lead compounds through the following (Fig. 1b):

1. The planar coumarin (1,2-benzopyrone) scaffold is a bioisostere to the quinazoline scaffold in erlotinib and tetrahydronaphtalene-1,7-dione nucleus in EXM. Such a skeleton will be essential for anticancer activity^24,25^.
2. The hydrophobic phenyl ring attached to the C^4^-position of the coumarin nucleus by introducing a spacer is previously considered a biologically active pharmacophore that is considered to be crucial for the activity^26–29^.
3. The reported SAR research demonstrated that conjugates containing benzyl groups at the C^7^-position exhibited more activity than their alkyl analogs. Introducing F or Cl groups on the benzyl ring resulted in good activity^19,23^.
4. The rotatable atoms of the linker enable the phenyl group to close the entrance cavity in the aromatase binding site^30^.

Based on the above findings, we worked on synthesizing 7-substituted coumarin derivatives with flexible C^4^-position ester functionality to examine their impact on two BC cell lines, MCF-7 and MDA-MB-231 cell lines, as well as normal breast cells MCF-10A. The synthetic compounds were tested in vitro for their ability to inhibit the EGFR and ARO enzymes.

The most potent compounds, such as cell cycle examination and apoptosis markers, were selected for additional studies to discover the molecular process behind their anticancer activity.

Moreover, molecular modeling studies were conducted to identify the two enzymes’ interaction patterns and investigate the relationship between their physicochemical features and inhibitory action. Additionally, QSAR analysis was performed.

## Results

### Chemistry

**Figure 2:**
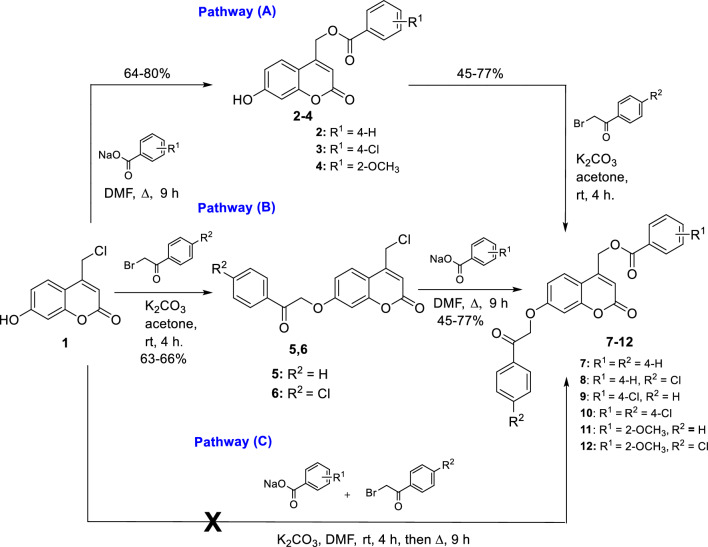
Synthetic pathways of the new coumarin derivatives (**7–12**).

**Figure 3:**
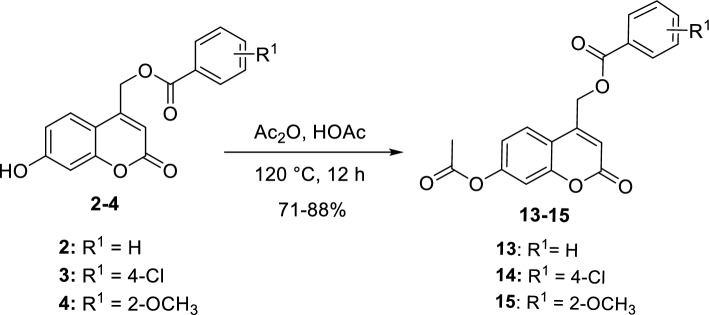
Synthesis of new acetyl coumarin derivatives (**13–15**).

The steps in Figs. 2 and 3 were used to synthesize the intermediates and target molecules. The requisite 4-chloromethyl-7-hydroxycoumarin (**1**)^31^ was first synthesized through a Pechman cyclocondensation reaction involving acid-catalyzed condensation of resorcinol with ethyl 4-chloroacetoacetate in a readily scalable procedure^32,33^.

7-Hydroxycoumarin derivatives **2**–**4** were obtained through the nucleophilic substitution reaction from compound **1** with carboxylate ion bearing Na ion as the countercation in DMF (Fig. 2). The structures of new intermediates derivatives **2**–**4** were confirmed using spectral data. ^1^H NMR spectrum showed the appearance of exchangeable OH protons at 10.69–10.72 ppm and CH_2_ protons at 5.50–5.60 ppm. Besides, ^13^C NMR exhibited signals at 165.6–166.9 assigned to C=O of the carboxylate group. All the aromatic carbons appeared in the expected region. Moreover, the IR spectrum showed a characteristic broad band at 3100–3200 cm^−1^ corresponding to the OH group and a typical carbonyl group absorption at 1690–1720 cm^−1^ corresponding to two carbonyl groups.

The intermediates **5** and **6** were synthesized through benzoylation of the phenolic 7-OH group in **1** with the appropriate phenacyl bromide in the presence of acetone and K_2_CO_3_. Confirmation of structures by spectral data revealed the appearance of singlet ^1^H NMR signal at δ 5.77–5.79 characteristic for the newly formed –OCH_2_– and appearance of new C=O group ^13^C NMR signal at δ 193.3–194.8.

New Target coumarin derivatives **7–12** were obtained from **1** through two pathways: nucleophilic substitution of **1** and benzoylation of **2**–**4** (pathway A, Fig. 2)^34,35^. The order of these two steps can be reversed to produce the same target coumarin derivatives **7–12** (Pathway B, Fig. 2). It was observed that pathway B was less favorable than A in relation to the yield and purity of products. This may be due to competitive alkylation at the acidic methylenic group at position-4. The bulky benzoate group will hinder this side reaction sterically more than the chloride group, which goes along with the literature^31^. The reported yields (45–77%) and compound's characteristics of **7**–**12** were based on pathway A. It is worth reporting that the one-pot reaction was tried, but unfortunately, the yield and purity of products were poor (Pathway C, Fig. 2).

The structures of **7**–**12** were confirmed by spectral data in which the exchangeable proton OH disappeared in their ^1^H NMR. A singlet signal of COCH_2_ protons appeared at δ 5.78–5.81 ppm, and another singlet for CH_2_Cl appeared upfield at δ 5.60–5.64 ppm. The aromatic protons were found at the expected region and pattern. Moreover, ^13^C NMR spectra revealed the presence of three carbonyl groups at the ranges of 196.4–193.3, 165.6–164.8, and 162.9–161.6, corresponding to benzoyl C=O, benzoate C=O, and coumarin C=O; respectively.

As shown in Fig. 3, the acetylation reaction was carried out with a mixture of acetic anhydride and acetic acid to yield O-acetylated coumarins (**13–15**)^36^. The presence of the acetyl group was confirmed by a singlet peak at 2.33–2.34 ppm corresponding to COCH_3_ in ^1^H NMR and a signal of C=O at 169.2–169.3 in ^13^C NMR. In addition, the singlet signal of OCH_2_ in ^1^H NMR spectrum was found at 5.62–5.67 ppm. The aromatic protons were found at their expected aromatic regions. ^13^C NMR spectra proved the presence of three carbonyl groups at the ranges of 169.2–169.4, 165.4–165.6, and 158.9–159.8 corresponding to acetyl C=O, benzoate C=O, and coumarin C=O, respectively. All the chemical synthetic steps were found adequate and concise with appropriate yields.

### In vitro biological evaluation

#### Measurement of in vitro cytotoxic activity by MTT assay

For the MCF-7 cell line, results shown in Table 1 revealed that the most powerful compounds were methyl benzoate derivatives **2** and **8** with IC_50_ = 6.0 µM and 5.8 µM, respectively, compared to IC_50_ = 5.6 µM for the standard drug DOX.

**Table 1:** In vitro cytotoxic activities (IC_50_, µM and S.D values) of compounds **1**–**15** against MCF-7, MDA-MB-231 and nonmalignant cells MCF-10A cell lines.

Comp. No.	MCF-7 IC_50_, µM	MDA-MBA-231 IC_50_, µM	MCF-10A IC_50_, µM	SI for MCF-10A
1	115.3 ± 1.47	353.7 ± 4.06	441.5 ± 4.53	3.8 MCF7 1.2 MDA
2	**6.0 ± 0.11**	51.9 ± 0.84	185.7 ± 2.68	30.8 MCF7 3.5 MDA
3	36.6 ± 0.74	67.5 ± 1.22	369.1 ± 5.93	10.1 MCF7 5.5 MDA
4	17.2 ± 0.34	**6.4 ± 0.11**	127.5 ± 2.03	7.4 MCF7 20 MDA
5	39.5 ± 0.79	**5.8 ± 0.1**	83.9 ± 1.35	2.1 MCF7 14.5 MDA
6	217.4 ± 4.77	56.6 ± 1.12	156.3 ± 2.76	0.72 MCF7 2.8 MDA
7	77.5 ± 1.94	56.7 ± 1.28	101.2 ± 2.04	1.3 MCF7 1.8 MDA
8	**5.8 ± 0.16**	27.5 ± 0.67	79.9 ± 1.74	13.6 MCF7 2.9 MDA
9	100.4 ± 2.73	10.4 ± 0.25	301.1 ± 6.56	3 MCF7 29 MDA
10	95.8 ± 2.81	**2.3 ± 0.06**	76.8 ± 1.81	0.8 MCF7 33.4 MDA
11	130.7 ± 3.52	61.2 ± 1.48	178.2 ± 3.86	1.4 MCF7 2.9 MDA
12	13.2 ± 0.38	**3.5 ± 0.09**	72.1 ± 1.68	5.5 MCF7 20.6 MDA
13	111.1 ± 2.28	30.7 ± 0.56	490.4 ± 8.1	4.4 MCF7 15.9 MDA
14	284.9 ± 6.41	**1.9 ± 0.03**	263.1 ± 4.77	0.9 MCF7 138.4 MDA
15	17.1 ± 0.38	74.9 ± 1.52	312.2 ± 5.62	18.2 MCF7 4.1 MDA
Doxorubicin	5.6 ± 0.18	7.3 ± 0.22	22.8 ± 0.6	4.1 MCF7 3.1 MDA

Significant values are in bold.

For MDA-MB-231, the most promising action was reported for compounds **4**, **5**, methyl benzoate derivatives **10**, **12**, and compound **14** with IC_50_ values of 6.4, 5.8, 2.3, 3.5, and 1.9 µM, respectively, compared to the standard drug DOX which had IC_50_ value of 7.3 µM.

#### Structure–activity relationship (SAR) study

Starting compound **1** was devoid of activity against both types of cell lines.

The introduction of benzoate substituent at position-4 in the coumarin scaffold was observed to potentiate the activity. This is possibly explained by specific hydrophobic interactions of the phenyl group.

Evidently, the compound bearing unsubstituted benzoate **2** exhibited superior selectivity against MCF-7 than against MDA-MB-231 with IC_50_ = 6 µM versus IC_50_ = 51.9 µM, respectively, compared to the reference drug.

Incorporation of the electron-withdrawing group at position-4 on the aromatic moiety in compound **3** showed moderate selectivity towards MCF-7 than MDA-MB-231. Conversely, the substitution of an electron-donating methoxy group at position-2 as in compound **4** resulted in a beneficial selectivity against MDA-MB-231 than MCF-7 cell lines was IC_50_ values = 6.4 and 17.2 µM, respectively.

The activity of 7-(2-oxo-2-phenylethoxy)-4-methyl benzoate coumarin series (**7–12)** varied according to the type of substituents compared to intermediates **2–4**. Incorporation of 2-oxo-2-phenylethoxy at position-7 of coumarin moiety in compounds **7, 9,** and **11** diminished the activity against the MCF-7 cell line while resulting in poor anti-proliferative activity against the MDA-MB-231 cell line. Compound **9** bearing halogen atom at *p*-position was identified as the most significant cytotoxic candidate among the series revealing potent selectivity against the MDA-MB-231 cell line with IC_50_ value of 10.4 µM.

In general, mono substitution with an electron-withdrawing group on phenyl moiety such as p-chloro atom located at C^7^- of coumarin ring in compounds **8**, **10**, **12** and **14** dramatically improved the activity against both cell lines, in comparison to the unsubstituted derivatives. In this context, it could be stressed that the target compound **8** showed high selectivity against MCF-7 than MDA-MB-231 (IC_50_ = 5.8 µM versus IC_50_ = 27.5 µM), while compounds **10**, **12** and **14** showed superior selectivity against MDA-MB-231 with IC_50_ = 2.3, 3.5 and 1.9 µM, respectively. Fortunately, compound **12** was the most potent among this series against both MCF-7 and MDA-MB-231 with IC_50_ = 13.2 µM and 3.5 µM, respectively.

Surprisingly, compound **6** with an electron-withdrawing chlorine atom exhibited a lower activity level against both cell lines, which was assumed to involve* H*-bonds with amino acids in the ligand-binding domain.

Concerning coumarins **13**–**15** carrying 7-acetyloxy group, derivative **14** showed superior selectivity against MDA-MB-231 than MCF-7 by 150 times with (IC_50_ = 1.9 µM). In contrast, compound **15** showed moderate anti-proliferative activity against MCF-7 (IC_50_ = 17.1 µM).

#### In vitro cytotoxicity against nonmalignant human cells

The compounds were also examined against the human *nonmalignant cells* MCF10A (breast) to investigate their selectivity between *nonmalignant cells* and cancerous cells. Compared to DOX (IC_50_ = 12.4 µM), the most active compounds **8**, **10**, **12** and **14** were less toxic to MCF10A cells (IC_50_ = 79.9, 76.8, 72.1 and 263.1 µM, respectively) with good SI values (more than 10) (Table 1).

#### In vitro* enzyme inhibition assays*

The most active compounds were subjected further to in vitro enzyme inhibition via ELISA enzyme assay.

- EGFR enzyme inhibition assay

MDA-MB-231 cell lines could express mainly EGFR enzyme, whereas MCF-7 cell lines only produced aromatase enzyme^23^. So, an enzyme assay study was performed for ARO and EGFR enzymes in MCF-7 and MDA-MB-231 cell lines, respectively.

It was observed that compounds **10** and **12** showed the highest inhibition effect towards EGFR, displaying 97.67% and 81.92% of the potency of erlotinib, respectively (Table 2, Supplementary Fig. S1). Therefore, their potent selectivity against MDA-MB-231 might be due to EFGR inhibition pathway.

- (ARO) enzyme inhibition assay

**Table 2:** IC_50_, Erlotinib and percentage above IC_50_ of erlotinib towards EGFR.

Compound No.	EGFR inhibition IC_50_ **µM**	Reference **Erlotinib µM**	Percentage above Reference IC_50_
2	0.864 ± 0.054		9.722
3	0.974 ± 0.06		8.624
4	0.438 ± 0.027		19.178
6	0.154 ± 0.008		54.545
7	0.341 ± 0.019		24.633
8	1.1 ± 0.066		7.636
9	0.131 ± 0.009		64.122
10	**0.086 ± 0.006**	0.084 ± 0.005	**97.674**
13	0.363 ± 0.020		23.14
5	0.316 ± 0.0066		54.886
11	1.833 ± 0.038		7.91
12	**0.177 ± 0.0018**	0.145 ± 0.003	**81.92**
14	2.44 ± 0.051		5.942

Significant values are in bold.

To convert androgens into estrogens, the MCF-7 cell line was shown to possess enough aromatase enzyme activity^37^. Results are presented in Table 3, Supplementary Fig. S2, and revealed that target compound **8** is considered the most potent AIs with (IC_50_ value = 0.114 µM), compared to EXM (IC_50_ value = 0.108 µM), and this described the elevated value of IC_50_ in MCF-7 (5.8 µM). Furthermore, target compound **2** exhibited half ARO inhibitory activity as that of reference EXM.

**Table 3:** IC_50_ and percentage above IC_50_ of *exemestane* towards ARO.

Compound No.	ARO inhibition IC_50_ (µM)	Percentage above Reference IC_50_
2	**0.216 ± 0.01**	**50**
3	2.13 ± 0.115	5.07
4	1.557 ± 0.086	6.936
5	1.067 ± .057	10.121
6	3.935 ± 0.215	2.744
7	1.832 ± 0.099	5.895
8	**0.114 ± 0.007**	**94.736**
9	3.001 ± 0.162	3.598
10	3.005 ± 0.163	3.594
11	5.98 ± 0.326	1.806
12	0.384 ± 0.02	28.125
*EXM*	0.108 ± 0.006	**–**

Significant values are in bold.

#### Cell cycle arrest

Results are presented in Table 4, Supplementary Fig. S3. Regarding the MCF-7 cell line, the percentage of apoptotic cells was increased significantly in the pre-G1 phase by 18.67 folds with significant arrest in G0-G1 and S phases by 1.508 and 1.33 folds, respectively compared to the untreated control cells for compound **8**. For MDA-MB-231 cell lines, when treated with compounds **10, 12** and **14**, the percentage of apoptotic cells in the pre-G1 phase increased by 16.22, 22.46 and 19.9 folds with consequential cell cycle arrest at the S phase by 1.59, 1.08 and 1.38 folds, respectively. Results indicated that compound **8** tested against the MCF-7 cell line and compounds **10**, **12** and **14** tested against the MDA-MB-231 cell line showed pre-G1 apoptosis and cell cycle arrest at the S phase in addition to G0-G1 by compound **8**.

**Table 4:** Results of cell cycle analysis in MCF-7 and MDA-MB-231 cell lines expressed as % of cells in each phase for the selected compounds (**8** in MCF-7 and **10**, **12** and **14** in MDA-MB-231).

Compound No.	Percentage cell count (% cells)				*p*-value
	%Pre-G1	%G0-G1	%S	%G2/M	
8/MCF7	31.19^ae^ ^AB^	55.83^c^ ^ACD^	39.85 ^CE^	4.32^a^ ^BDE^	*P* < 0.001*
Control MCF7	1.67^abc^ ^ABC^	52.93^b^ ^ADE^	29.91^b^ ^BDF^	17.16^ab^ ^cCEF^	*P* < 0.001*
10/MDA-MB-231	33.43^bdf^ ^AB^	51.97^ad^ ^AC^	43.82^bc^ ^D^	4.21^b^ ^BCD^	*P* < 0.001*
12/MDA-MB-231	46.28^cdg^ ^ABC^	63.58^abc^ ^ADE^	29.97 ^BDF^	6.45^c^ ^CEF^	*P* < 0.001*
14/MDA-MB-231	41.02^iklmC^	54.69^ikBD^	38.22^ABE^	7.09^dACDE^	*P* < 0.001*
Control MDA-MB-231	2.06^efg^ ^ABC^	61.39^d^ ^ADE^	27.51^c^ ^BDF^	11.1 ^CEF^	*P* < 0.001*
*p* value	*p* < 0.001	*p* = 0.02*	*p* = 0.03*	*p* = 0.03*	

Similar superscripted small letters in same column denote significant difference between groups by Z test.

Similar superscripted capital letters in same row denote significant difference between different stages by Z test.

#### Apoptosis determination using annexin V-FITC/PI dual staining assay.

To further assure the apoptotic ability of compounds **8**, **10**, **12** and **14** using dual staining with propidium iodide (PI) and annexin-V-FITC, a flow cytometric analysis was carried out to distinguish between living, early, late apoptotic and necrotic cells. Propidium iodide stains late apoptotic and necrotic cells’ DNA, while annexin-V binds powerfully and distinctively to phosphatidylserine (PS) on their surfaces and fluoresces green^38^. The assay results are provided in Table 5 and (Supplementary Figs. S4 and S5) as fluorescence-activated cell sorter (FACS) and cytometry profiles with PI on the Y-axis and annexin V-FITC on the X-axis. In addition, each profile’s quadrant has four sections: necrosis, late and early apoptosis, and living cells, clockwise from the upper left. Results are demonstrated in Table 5, indicating that compound **8** showed % apoptosis 31.19 for MCF-7 cells while % apoptosis for MDA-MB-231 cells of compounds **10**, **12** and **14** was found to be 33.43, 46.28 and 41.02, respectively; compared to 1.67 and 2.06 for control untreated MCF-7 and MDA-MB-231.

**Table 5:** Results of annexin V-FTIC/PI dual staining assay expressed as % Apoptosis and % Necrosis induced by compound **8** in MCF-7 cells and compounds **10**, **12** and **14** in MDA-MB-231 cells.

Compound No.	% Apoptosis			% Necrosis
	Total	Early	Late	
8/MCF7	31.19^abc^	4.94^abcA^	19.66^acA^	6.59
Control MCF7	1.67^ade^	0.25^ade^	0.19^ade^	1.23
10/MDA-MB-231	33.43^df^	9.61^df^	17.83^df^	5.99
12/MDA-MB-231	46.28^be^	16.75^beg^	23.02^eg^	6.51
14/MDA-MB-231	41.02^gh^	23.61^ilmn^	13.22^hik^	4.19
Control MDA-MB-231	2.06^cf^	0.64^cfg^	0.18^cf^	1.24
*p* value	< 0.001*	< 0.001*	< 0.001*	0.07

Similar superscripted small letters in same column denote significant difference between groups by Z test.

Similar superscripted capital letters in same row denote significant difference between different stages by Z test.

#### Effect on the levels of Bax/Bcl-2

Apoptosis is provoked in a cell through two major apoptotic pathways, the extrinsic and intrinsic pathways. By overexpressing anti-apoptotic proteins like Bcl-2 or downregulating pro-apoptotic proteins like Bax through the intrinsic apoptotic pathway, cancer cells can develop apoptotic resistance^39^. In this study, we measured the levels of Bax and Bcl2 to evaluate the effects of compounds **8**, **10**, **12** and **14,** which showed promising apoptosis-inducing activity on the intrinsic apoptotic pathway. As shown in (Supplementary Table S6 and Supplementary Fig. S7), the tested compounds **8**, **10, 12** and **14** significantly increased the expression of the pro-apoptotic protein Bax by 5.27, 5.27, 5.22 and 4.9 folds, respectively, with a consequent decline in the anti-apoptotic protein Bcl-2 levels by 0.44, 0.377, 0.271 and 0.329 folds, respectively compared to the control. As a result, a significant increase in the Bax/Bcl-2 ratio was observed, supporting the claim that the tested compounds can improve the therapeutic response in breast cancer.

#### Caspase-9 activation

Caspase-9, activated in apoptotic cells by intrinsic pathways, can be used as a marker for the induction of apoptosis by anticancer agents.^40^. In the present study, the levels of caspase-9 were then measured in the compounds that showed a noticeable increase in the Bax/Bcl-2 ratio to identify a possible pathway for the antiproliferative activity of the most promising compounds. Significantly elevated levels of caspase-9 protein level in treated samples were observed in (Supplementary Table S8 and Supplementary Fig. S9) by 6.71 folds for compound 8, while compounds **10, 12** and **14** resulted in 6.42-, 7.33- and 4.37-folds elevation, respectively, compared to control cells. These results suggested that all tested compounds could induce a significant overexpression in caspase-9 protein level higher than that elicited by control, assuming that apoptosis was probably due to the activation of caspase-9 protein.

### Statistical analysis and data interpretation

Data analysis was performed by SPSS software, version 25 (SPSS Inc., PASW Statistics for Windows version 25. Chicago: SPSS Inc.). Apoptosis was described using numbers and percentages. Quantitative data were described using mean ± Standard deviation for normally distributed data after testing normality using Shapiro Wilk test. The significance of the obtained results was judged at the (≤ 0.05) level. Chi-Square and Z-tests were used to compare qualitative data between groups as appropriate. One Way ANOVA test was used to compare more than 2 independent groups with Post Hoc Tukey test to detect pair-wise comparison.

### In silico computational study

#### Docking into EGFR crystal structure

Re-docking results of the co-crystallized ligand (Erlotinib) and its binding mode^18,30,41^ were described in (Table 6, Supplementary Figs. S10, and S11).

**Table 6:** RMSD values and docking binding scores of the docked compounds.

Compound No.	RMSD (Å)	Binding score (S) Kcal/mol
Erlotinib	1.8362	− 7.5766
EXM	1.3178	− 8.2968
ASD	1.4491	− 8.6748
2	1.6493,	− 7.5426
8	1.8402	− 7.4464
10	1.925	− 8.1655
12	1.543	− 8.3211

Docking results showed that compounds **10** and **12** exhibited good binding interactions and docking scores similar to Erlotinib (Table 6). It is suggested that the planarity of quinazoline moiety in erlotinib seems important for stabilizing the EGFR binding site. Similarly, the planar nature of the coumarin scaffold in compounds **10** and **12** allowed them to be embedded into the active site of EGFR, as shown in their 2D interactions (Supplementary Fig. S12) and 3D binding modes as in Fig. 5. It was found that the C=O group of the coumarin skeleton overlapped with the N_1_ atom of the quinazoline moiety of erlotinib, which equivalently served to anchor with Met 769, which was considered the key interaction improving the stabilization. By investigating the binding site of the receptor, the presence of *o*-methoxy or *p*-chloro phenyl moiety at position 4-methyl carboxylate of coumarin nucleus in compounds **10** and **12**, respectively, were observed to be positioned in the hydrophobic pocket, which included side chains Leu 764, Leu 753, Met 742, Ile 720 and Ile 765 as for the ethynyl-phenyl group in erlotinib^42^ (Supplementary Fig. S13).

**Figure 4:**
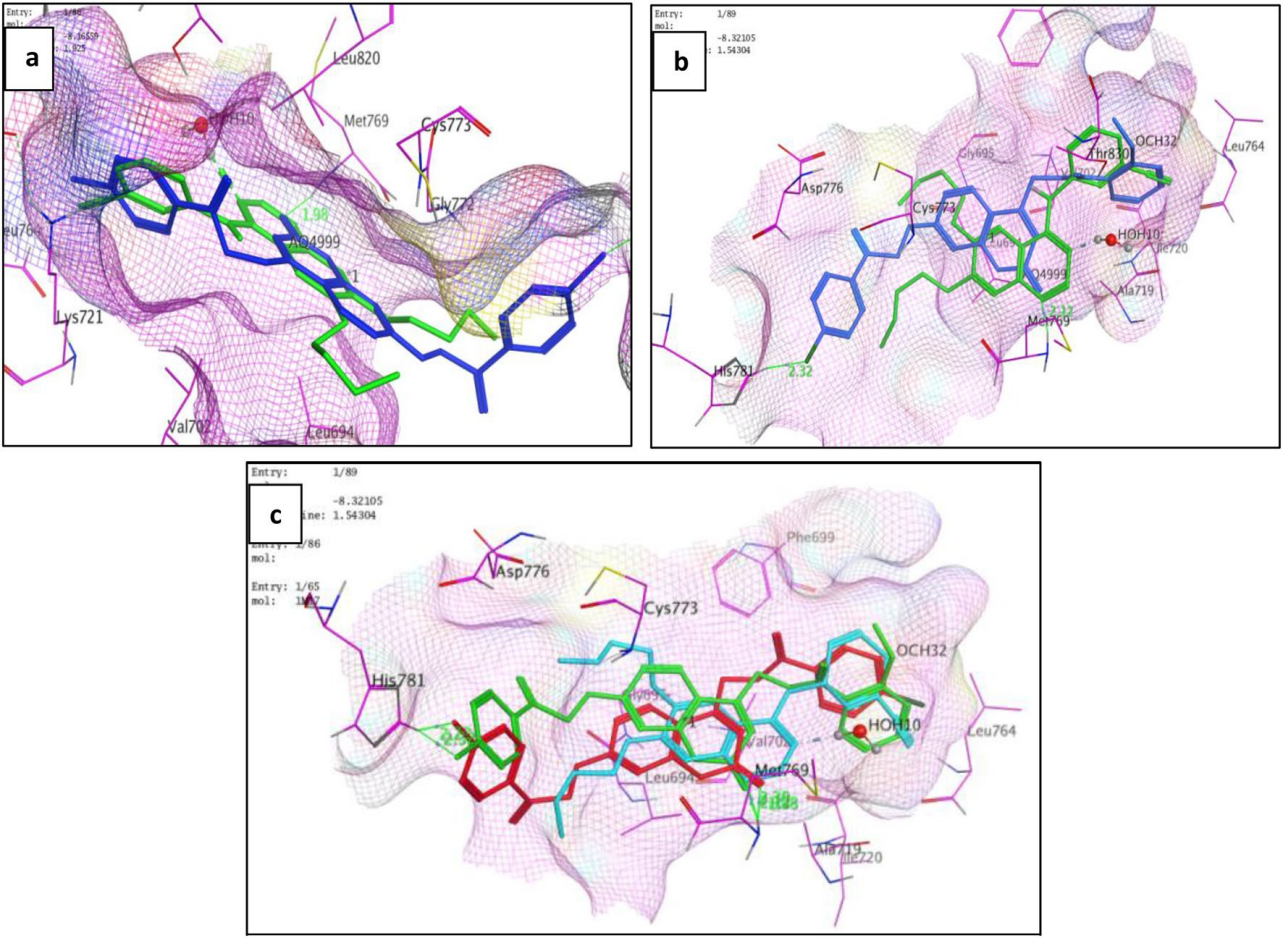
(**a**) Binding mode of compound **10** (blue) and erlotinib (green), (**b**) Binding mode of compound **12** (blue) and erlotinib (green), (**c**) Binding mode of compound **10** (red), compound **12** (green) and erlotinib (blue) occupying the active site. The 2 compounds had the same binding mode as the native ligand. They bound with the key residue Met 769. The presence of *p*-chloro atom improved their stability. The hydrophobic side chain in both compounds changed their orientation to embed in hydrophobic pocket. The coumarin moiety was buried in the pocket in parallel to quinazoline nucleus in erlotinib.

Moreover, additional halogen bond interactions were shown with the amino acid His 781 with the chloride atom on C_4_ of the phenyloxy side chain at C_7_ of the coumarin nucleus with bond length (2.22 Å for compound **10** and 2.32 Å for compound **12**). This interaction appears to help their immobilization; in the same way, the ether side chain in erlotinib as illustrated in (Fig. 4).

In order to rationalize the biological outcomes, it was believed that the poor EGFR inhibitory action of compounds **11** and **14** was related to their failure to bind with the essential amino acid and their low docking score of -5.7394 and -5.4982 kcal/ mol, respectively (Supplementary Fig. S14). Hence, the acquired data revealed that the binding modes of the investigated compounds were consistent with their EGFR inhibitory action.

On the other hand, despite compound **8** showing the same binding features as the most potent EGFR inhibitors **10** and **12** into the active site with a docking score of -6.7717 kcal/ mol and RMSD = 1.9314 Å, it unexpectedly possessed weak EGFR inhibitory activity. However, it could be stated that other factors that affect activity related to lipophilicity and solubility may be involved.

#### Docking into ARO crystal structure

Results of re-docked ligands (ASD and EXM) are summarized in (Table 6) and their binding modes are described in (Supplementary Fig. S15). Aromatase enzymes comprise a heme prosthetic group containing iron at the active reaction center^43^.

**Figure 5:**
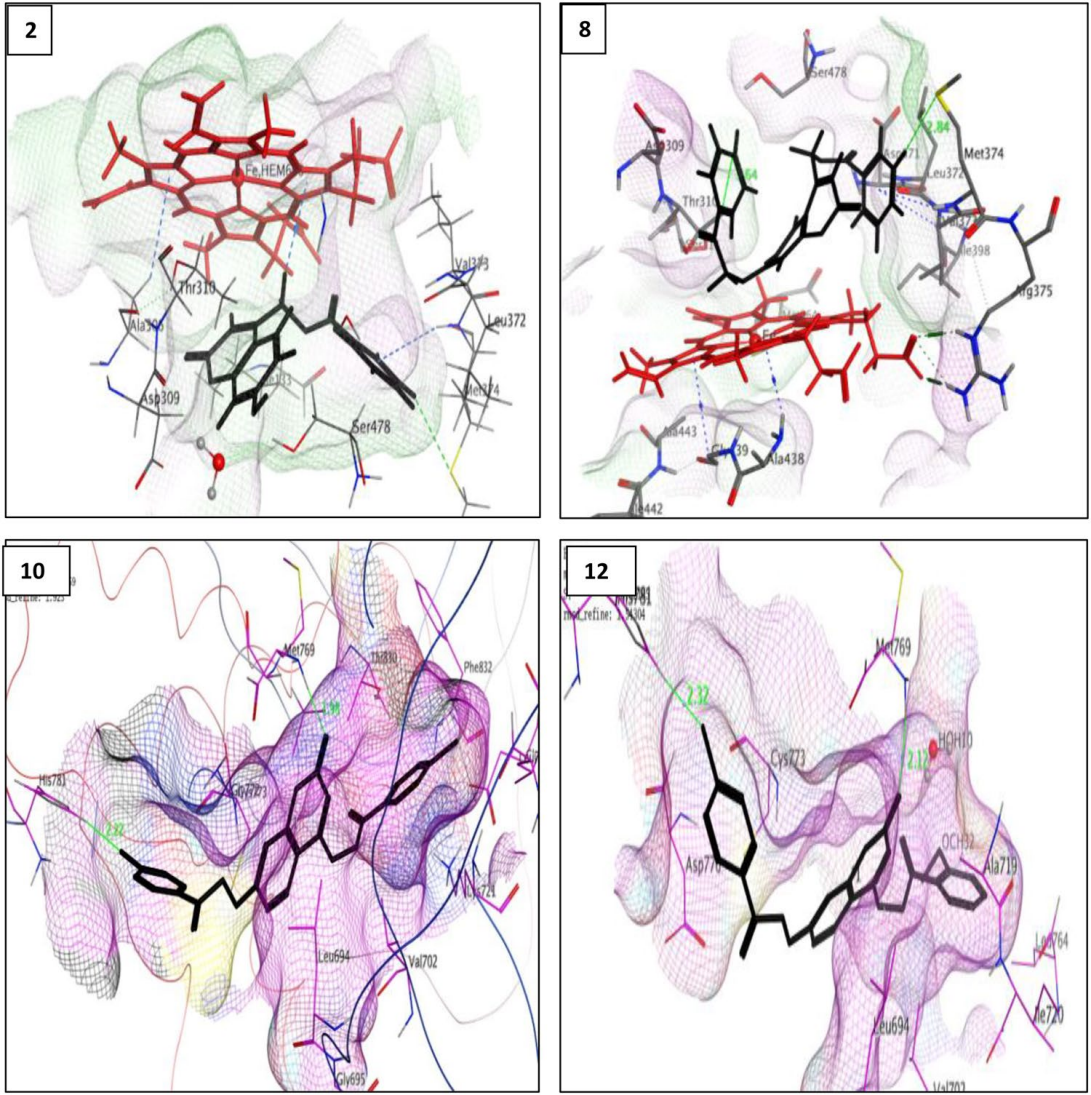
3D binding modes of compounds **2** and **8** inside ARO binding pocket (PDB ID: 3EQM) and compounds **10** and **12** into the active site of EGFR (PDB ID: 1 M17).

Regarding compound **8**, the most potent AI showed an acceptable binding mode (Table 6, Fig. 5). When compared to the binding mode of ligands, it was found that the coumarin scaffold was stabilized by arene-arene interaction with the 5-membered ring of the heme moiety, which is considered the binding driving interaction.

Unsubstituted benzoate at position-4 of coumarin is essential for activity, binding with Met 374 by H-bond (2.84 Å) and providing a convenient allocation in the hydrophobic pocket, constituted of Val 373, Met 374, Leu 372, Ile 398 residues.

Introducing a substituent on the phenyl moiety (*p*-chloro or *o*-methoxy) resulted in a dramatic decrease in activity due to either a steric effect or a change in lipophilicity.

In particular, the distance between the benzene ring placed in the hydrophobic pocket and the coumarin moiety is likely the same as the entire length of ASD (6.97 Å, 6.69 Å, respectively) and plays a significant role in the control of affinity. The substitution at position-4 on the methylene bridge could be responsible for the activity.

In addition, the phenyl ethoxy side chain at position-7 slightly twisted to be embedded into the binding site and seemed to induce additional weak *H*-bond with Thr 310 (3.64 Å) to close the entrance cavity between Asp 309 and Ser 478^20^. The longer the substituent at position-7 could justify the better affinity to the binding site than the free OH moiety. These three binding site interactions were fulfilled when the coumarin ring carried benzyl carboxylate methyl group at position-4 and *p*-chlorophenyl ethoxy substituent at position-7. Therefore, the better binding mode of target compound **8** when compared to compound **2** can be explained, in which it showed the same binding manner with the backbone amide group of Met 374 (3.42 Å) and the heme moiety through arene–*H* interaction. Although, the free hydroxyl group at position-7 exhibited a decrease in activity due to the inability to close the entrance side of the pocket (Fig. 5). The 2D binding poses of both compounds are shown in (Supplementary Fig. S16).

On the other hand, because of the correlation between the bulkiness of the position-4 substituent and the activity, it may be presumed that the *o*-methoxy substituent in compound **11** (IC_50_ = 5.98 µM) generated steric hindrance in the rear side of the pocket and unstable binding mode with score (S) = − 0.1435 kcal/mol. In addition, compound **6** (IC_50_ = 3.93 µM) did not illustrate the three-site binding hypothesis since its molecular volume is smaller than the pocket site. They both shared the lack of essential interaction in either the rear side or the entrance side and unacceptable values of RMSD of more than 2 (Supplementary Fig. S17).

#### Bioavailability and ADMET properties screening

To understand the pharmacokinetic behavior of the most active compounds **8**, **10, 12** and **14,** their physicochemical characters were calculated with the aid of the online application ADMETSAR server http://lmmd.ecust.edu.cn/admetsar1 by applying various qualitative ADMET models as described in (Table 7). The results revealed that all compounds possessed good intestinal absorption and could penetrate the blood–brain barrier. Besides, the candidate compounds illustrated good oral bioavailability, making them promising candidates as anti-BC agents.

**Table 7:** Some ADMET properties of the target compounds using admet SAR server.

Models	Comp. 8	Comp. 10	Comp. 12	Comp.14
Human intestinal absorption	+	+	+	+
Human oral bioavailability	+	+	+	+
Blood brain barrier	BBB +	BBB +	BBB +	BBB +
CYP2D6 inhibition	Non inhibitor	Non inhibitor	Non inhibitor	Non inhibitor
CYP3A4 inhibition	Non inhibitor	Non inhibitor	Non inhibitor	Non inhibitor
Estrogen receptor binding	+	+	+	+
Aromatase binding	+	+	−	+
Ames mutagenesis	−	−	−	−
Carcinogenicity	Non required	Non required	Non required	Non required
Biodegradation	Not biodegradable	Not biodegradable	Not biodegradable	Not biodegradable
Honey bee toxicity	−	−	−	+
Acute oral toxicity (c)	III	III	III	III
Water solubility (Log S)	− 4.16834	− 4.09608	− 4.27475	− 4.22896

#### QSAR study

The QSAR analysis was carried out on the synthesized compounds against MDA-MB-231 cancer cells, where most compounds showed good selectivity using MOE software. The synthesized compounds were used as a training set with their experimental pIC_50_ (- log IC_50_). Various physicochemical descriptors were calculated.

Vsurf descriptors explained the hydrophilic and hydrophobic interactions, while the hydrophobic bindings were described by the vsurf_D descriptors (vsurf_D5 and vsurf_D3)^44,45^.

The following equation represents the best QSAR model is: pIC_50_ = 7.49008 + 0.41537 log *P* (o/w)—0.01752 E_vdw + 0.26664 opr_nrot—0.02428 vsurf_D3—0.00142 Weight + 0.01549 vsurf_D5—0.26814 logS (with root mean square error (RMSE) = 0.39205 and squared correlation coefficient (r^2^) = 0.21403. According to the previous equation, the anticancer activity was enhanced by log P (lipophilicity), vsurf D5 (hydrophobic bindings), and Opr nrot (number of rotatable bonds), while the activity is negatively correlated with the following: van der Waal energy, polar interactions, molecular weight, and log S (aqueous solubility).

**Figure 6:**
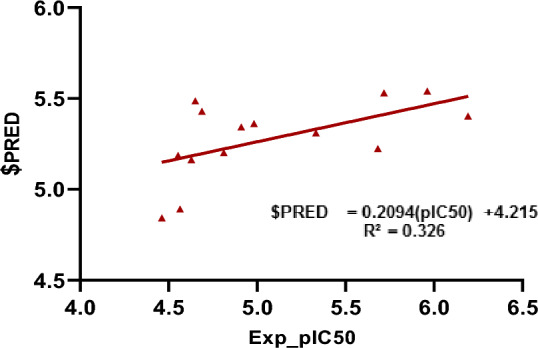
Experimental versus predicted pIC_50_ of the tested compounds against MCF-7 human cancer cell line.

The reliability of the built model was confirmed by excellent linearity [$PRED = 0.2094 (pIC_50_) + 4.215], with R^2^ = 0.326 (Fig. 6) and the relative values between the predicted and experimental activities (Table 8). It was found that the predicted values are close to those experimentally investigated, indicating that the QSAR model is reliable and can be safely applied to predict more effective compounds.

**Table 8:** The experimental, predicted pIC_50_ of the synthesized compounds and the residual.

Comp. No.	Exp_pIC50	$PRED	Residual
2	4.812	5.200	− 0.388
3	4.651	5.486	− 0.835
4	5.682	5.223	0.459
5	5.718	5.529	0.189
6	4.688	5.428	− 0.74
7	4.628	5.162	− 0.534
8	4.91	5.342	− 0.432
9	5.332	5.310	0.022
10	5.962	5.539	0.423
11	4.565	4.891	− 0.326
12	4.462	4.842	− 0.38
13	4.982	5.361	− 0.379
14	6.193	5.402	0.791
15	4.554	5.186	− 0.632

## Discussion

In the current study, a new series of nonsteroidal 4,7-disubstituted coumarin (1,2-benzopyrone) derivatives bearing methyl ester moiety at the C^4^-position were designed as anti-BC agents. Easy and good-yielding synthetic pathways were adopted to prepare the designed compounds. MTT assay method was used to evaluate their in vitro anti-BC activity against MCF-7, MDA-MB-231, and MCF-10A cell lines using Doxorubicin (DOX) as a reference drug. Generally, most of the tested compounds revealed MDA-MB-231 selectivity more than MCF-7. Particularly, compounds **2** and **8** exhibited obvious selectivity against MCF-7, while compounds **4**, **5**, **10**, **12,** and **14** exhibited considerable selectivity against the MDA-MB-231 cell line.

In conclusion, it might be emphasized that "mono substitution with electron-withdrawing groups as chlorine on phenyl moiety is crucial for the anti-BC activity against both cell lines as it could be involved in electrostatic interactions.

The cytotoxic activity of compounds **10** and **12** against MDA-MB-231 could be due to its potent EGFR inhibitory activity, while compound **8** against MCF-7 might be attributed to its ARO inhibitory activity. Regarding compound **14**, it possessed weak inhibitory activity toward EGFR despite its potent anticancer activity in MDA-MB-231 cell lines. Consequently, further investigations for the most active compounds **8**, **10, 12** and **14** were done to discover more mechanistic pathways for their antiproliferative activity, such as cell cycle analysis and apoptosis assay using flow cytometry. It was found that they were able to induce cell cycle arrest at G0-G1 and S phases. Their apoptotic mechanism was proved by a significant increase in both Bax/Bcl-2 ratio and caspase-9. The inhibitory activities of compounds **2** and **8** on ARO enzymes and **10** and **12** on EGFR were confirmed by docking studies, highlighting significant binding interactions***.*** ADMET study was also carried out to further describe the synthesized drugs' pharmacokinetics. The findings of the QSAR model revealed that there should be a balance between the compounds' hydrophilic and hydrophobic substituents, which was confirmed by the docking results. Therefore, the observed results showed that these compounds were susceptible to being modified further to serve as promising multitargeted anti-BC agents (Supplementary Fig. S18).

## Methods

### Chemistry

Melting points were determined on Stuart melting point apparatus and are uncorrected. Microanalyses were performed at Cairo University and performed on a Perkin-Elmer 240 elemental analyzer for C, H, and N elements, and the results were within the acceptable range of the theoretical values. ^1^H and ^13^C NMR were performed at Mansoura University and recorded on Brucker 400 MHz spectrometer and 100 MHz spectrometer, respectively. Chemical shifts are expressed in δ ppm with reference to TMS. Using a Nicolet iS10 infrared spectrometer, IR spectra were recorded at Mansoura University. Mass spectral analyses were performed on Thermo SCIENTIFIC DCQII at Azhar University. All the used chemicals and reagents were purchased from Aldrich Chemicals Co, USA.

#### General procedure for the preparation of (7-hydroxy-2-oxo-2H-chromen-4-yl) methyl benzoate derivatives (2–4)

To a solution of **1** (0.45 g, 2 mmol) in DMF (8 mL), the appropriate sodium salt of (un)substituted benzoic acid (2 mmol) was added. The reaction mixture was heated at 90 °C overnight, then cooled and poured over ice-water. The separated solid products were filtered off, dried and then recrystallized from EtOAc/MeOH (4:1).

#### (7-Hydroxy-2-oxo-2H-chromen-4-yl)methyl benzoate (2).

White solid; Yield 72%; mp 248–250 °C. IR (KBr) υ_max_ (cm^−1^): 3154 (OH); 2943 and 2840 (CH aliphatic); 1720 and 1689 (C=O); 1614 and 1568 (C=C); 1136 (C–O–C). ^**1**^**H NMR** (400 MHz, DMSO-*d*_*6*_): δ 10.72 (s, 1H, OH, D_2_O exchangeable), 8.08 (d, 2H, *J* = 7.5 Hz, Phenyl-C_2_-H and C_6_-H), 7.73 (t, *J* = 7.2 Hz, 1H, Phenyl-C_4_-H), 7.68 (d, *J* = 8.6 Hz, 1H, C_5_-H), 7.59 (t, 2H, *J* = 7.6 Hz, Phenyl-C_3_-H and C_5_-H), 6.85 (dd, 1H, *J*_1_ = 8.6, *J*_2_ = 1.5 Hz, C_6_-H), 6.79 (d, 1H, *J* = 1.5Hz, C_8_-H), 6.29 (s, 1H, C_3_-H), 5.60 (s, 2H, OCH_2_). ^**13**^**C NMR** (100 Hz, DMSO-*d*_*6*_), δ (ppm): δ 165.6 (C=O), 162.0 (C_2_=O), 160.6 (C_7_), 155.6 (C_4_), 150.9 (C_8a_), 134.3 (Phenyl-C_4_), 130.0 (two Phenyl-C_2_ and C_6_), 129.5 (two Phenyl-C_3_ and -C_5_), 129.4 (Phenyl-C_1_), 126.6 (C_5_), 113.7 (C_6_), 110.0 (C_4a_), 108.8 (C_3_), 103.0 (C_8_), 62.5 (CH_2_). **MS** (m/z %): 296.22 (1.39, M^+^), 294.85 (M-H^+^), 105.18 (100). **Elemental analysis** for C_17_H_12_O_5_, calcd.: C, 68.92; H, 4.08; Found: C, 68.78; H, 4.26.

#### (7-Hydroxy-2-oxo-2H-chromen-4-yl)methyl 4-chlorobenzoate (3).

White solid; Yield 80%; mp 173–175 °C. IR (KBr) υ_max_ (cm^−1^): 3174 (OH); 2930 and 2846 (CH aliphatic); 1721 and 1690 (C=O); 1599 (C=C); 1130 (C–O–C). ^**1**^**H NMR** (400 MHz, DMSO-*d*_*6*_): δ 10.69 (s, 1H, OH, D_2_O exchangeable), 7.95 (d, 2H, *J* = 8.3 Hz, Phenyl-C_2_-H and C_6_-H), 7.58 (d, 2H, *J* = 8.3 Hz, Phenyl-C_3_-H and C_5_-H), 7.69 (d, 1H, *J* = 8.7 Hz, C_5_-H), 6.86 (dd, 1H, *J*_1_ = 8.7, *J*_*2*_ = 1.3 Hz, C_6_-H), 6.77 (d, 1H, *J* = 1.3 Hz, C_8_-H), 6.43 (s, 1H, C_3_–H), 5.50 (s, 2H, OCH_2_). ^**13**^**C NMR** (100 Hz, DMSO-*d*_*6*_), δ (ppm) 166.9 (C=O), 161.7 (C_2_=O), 160.8 (C_7_), 155.7 (C_4_), 151.5 (C_8a_), 138.4 (Phenyl-C_4_), 131.6 (Phenyl-C_2_), 131.6 (Phenyl-C_6_), 129.9 (Phenyl-C_1_), 129.3 (two Phenyl-C_3_ and C_5_), 127.1 (C_5_), 113.6 (C_6_), 111.5 (C_4a_), 109.9 (C_3_), 102.9 (C_8_), 62.5 (CH_2_). **MS** (m/z %): 332.03 (22.66, M + 2), 330.50 (24.62, M^+^), 252.22 (100). **Elemental analysis** for C_17_H_11_ClO_5_, calcd.: C, 61.74; H, 3.35; Found: C, 61.97; H, 3.51.

#### (7-Hydroxy-2-oxo-2H-chromen-4-yl)methyl 2-methoxybenzoate (4).

White solid; Yield 64%; mp 268–270 °C. IR (KBr) υ_max_ (cm^−1^): 3260 (OH); 3097 (CH Aromatic), 2999, 2941 and 2836 (CH aliphatic); 1721 and 1693 (C=O); 1612 and 1567 (C=C); 1130 (C–O–C). ^**1**^**H NMR** (400 MHz, DMSO-*d*_*6*_): δ 10.69 (s, 1H, C_7_–OH, D_2_O exchangeable), 7.77 (d, 1H, *J* = 7.5 Hz, Phenyl-C_6_-H), 7.69 (d, 1H *J* = 8.7 Hz, C_5_–H), 7.62 (t, 1H, *J* = 7.9 Hz, Phenyl-C_4-_H),7.22 (d, 1H, *J* = 8.4 Hz, Phenyl-C_3_-H), 7.08 (t, 1H, *J* = 7.5 Hz, Phenyl-C_5_-H), 6.85 (dd, 1H, *J*_*1*_ = 8.7, *J*_*2*_ = 1.6 Hz, C_6_-H), 6.78 (d, 1H, *J* = 1.6 Hz, C_8_-H), 6.37 (s, 1H, C_3_-H), 5.55 (s, 2H, OCH_2_), 3.88 (s, 3H, CH_3_). ^**13**^**C NMR** (100 Hz, DMSO-*d*_*6*_), δ (ppm) δ 165.6 (C=O), 162.7 (C_2_=O), 161.8 (C_7_), 160.6 (Phenyl-C_2_), 158.8 (C_4_), 155.5 (C_8a_), 151.1 (Phenyl-C_4_), 134.7 (Phenyl-C_6_), 131.6 (C_5_), 126.5 (Phenyl-C_1_), 120.7 (Phenyl-C_5_), 119.3 (C_6_), 113.5 ((Phenyl-C_3_), 113.1 (C_4a_), 109.6 (C_3_), 108.6 (C_8_), 62.3 (CH_2_), 56.2 (OCH_3_). **MS** (m/z %): 326.24 (4.06, M^+^), 325.61 (10.6, M-H^+^), 44.22 (100). **Elemental analysis** for C_18_H_14_O_6_, calcd.: C, 66.26; H, 4.32; Found: C, 66.45; H, 4.59.

#### General procedure for the preparation of 4-(chloromethyl)-7-(2-oxo-2-phenylethoxy)-2H-chromen-2-one derivatives (5, 6)

To a solution of **1** (0.32 g, 1.5 mmol) in acetone (10 mL), anhydrous K_2_CO_3_ (0.29 g, 3 mmol) and the appropriate phenacyl bromide (1.5 mmol) were added. The reaction mixture was kept under stirring at room temperature for about 4 h and was monitored by TLC (petroleum ether/ethyl acetate, 7:3 v/v). After filtration, the solution was concentrated under vacuum. The obtained residue was recrystallized from a mixture of EtOAc/acetone (4:1).

#### 4-(Chloromethyl)-7-(2-oxo-2-phenylethoxy)-2H-chromen-2-one (5).

White solid; Yield 63%; mp 180–182 °C. ^**1**^**H NMR** (400 MHz, DMSO-*d*_*6*_): δ 8.06 (d, 2H, *J* = 7.2 Hz, Phenyl-C_2_-H and C_6_-H), 7.79 (d, 1H, *J* = 8.9 Hz, C_5_-H), 7.73 (t, 1H, *J* = 7.4 Hz, Phenyl-C_4_-H), 7.60 (t, 2H, *J* = 7.6 Hz, Phenyl-C_3_-H and C_5_-H), 7.16 (d, 1H, *J* = 2.5 Hz, C_8_-H), 7.10 (dd, 1H, *J*_*1*_ = 8.9, *J*_*2* =_ 2.5 Hz, C_6_-H), 6.52 (s, 1H, C_3_-H), 5.79 (s, 2H, OCH_2_), 5.02 (s, 2H, CH_2_Cl). ^**13**^**C NMR** (100 Hz, DMSO-*d*_*6*_), δ (ppm) 194.8 (C=O), 161.8 (C_2_=O), 160.4 (C_7_), 155.5(C_8a_), 151.2 (C_4_), 134.6 (Phenyl-C_4_), 134.4 (Phenyl-C_1_), 129.3 (two Phenyl-C_2_ and-C_6_), 128.3 (two Phenyl-C_3_ and -C_5_), 126.8 (C_5_), 113.2 (C_6_), 112.6 (C_3_), 111.2 (C_4a_), 102.45 (C_8_), 71.07 (CH_2_), 41.8 (CH_2_). **MS** (m/z %): 328.88 (19.45, M^+^), 330.20 (15.51, M^+^ + 2), 329.80 (10.36, M^+^ + 1), 125.26 (100). **Elemental analysis** for C_18_H_13_ClO_4_, calcd.: C, 65.76; H, 3.99; Found: C, 66.02; H, 4.13.

#### 4-(Chloromethyl)-7-(2-(4-chlorophenyl)-2-oxoethoxy)-2H-chromen-2-one (6)

Buff solid; Yield 66%; mp 195–197 °C. ^**1**^**H NMR** (400 MHz, DMSO-*d*_*6*_): δ 8.07 (d, 2H, *J* = 8.4 Hz, Phenyl-C_2_-H and C_6_–H), 7.79 (d, 1H, *J* = 8.8 Hz, C_5_–H), 7.69 (d, 2H, *J* = 8.4 Hz, Phenyl-C_3_-H and C_5_-H), 7.17 (d, 1H,* J* = 2.2 Hz, C_8_–H), 7.11 (dd, 1H, *J*_*1*_ = 8.8 Hz, *J*_*2*_ = 2.2 Hz, C_6_-H), 6.53 (s, 1H, C_3_-H), 5.77 (s, 2H, OCH_2_), 5.02 (s, 2H, CH_2_Cl). ^**13**^**C NMR** (100 Hz, DMSO-*d*_*6*_): δ (ppm) 193.3 (C=O), 161.7 (C_2_=O), 160.4 (C_7_), 155.5 (C_8a_), 151.2 (C_4_), 139.2 (Phenyl-C_4_), 133.3 (Phenyl-C_1_), 130.3 (two phenyl-C_2_ and-C_6_), 129.4 (two phenyl-C_3_ and -C_5_), 126.8 (C_5_), 113.2 (C_6_), 112.6 (C_3_), 111.2 (C_4a_), 102.4 (C_8_), 71.1 (CH_2_), 41.8 (CH_2_). **MS** (m/z %): 362.08 (2.37, M^+^), 363.81 (4.51, M^+^ + 2), 139.18 (100). **Elemental analysis** for C_18_H_12_Cl_2_O_4_, calcd.: C, 59.53; H, 3.33; Found: C, 59.74.; H, 3.58.

#### General Procedure for the Preparation of 7-[(2-oxo-2-phenylethoxy)-2H-chromen-4-yl] methyl benzoate derivatives (7–12)

##### Pathway (A)

The same procedure used for the synthesis of **5** and **6** was adopted, except that 7-hydroxycoumarin derivatives **2–4** were used instead of **1**.

##### Pathway (B)

The same procedure used for the synthesis of derivatives **2**–**4** was adopted, except that coumarin derivatives **5** and **6** were used instead of **1**.

##### Pathway (C)

To a solution of **1** (0.32 g, 1.5 mmol) in DMF (10 mL), the appropriate sodium salt of (un)substituted benzoic acid (1.5 mmol), anhydrous K_2_CO_3_ (0.29 g, 3 mmol) and the appropriate phenacyl bromide (1.5 mmol) were added. The reaction mixture was kept under stirring at room temperature for about 4 h, heated at 90 °C overnight, then cooled and poured over ice-water. The separated solid products were found to be an impure mixture of different products with very low yields.

#### [2-Oxo-7-(2-oxo-2-phenylethoxy)-2H-chromen-4-yl]methyl benzoate (7)

Buff solid; Yield 66%; mp 242–244 °C. ^**1**^**H NMR** (400 MHz, DMSO-*d*_*6*_): δ 8.07 (d, 2H, *J* = 7.3 Hz, Phenyl C_2_–H and C_6_–H), 7.96 (s, 2H, Phenyl C_2`_-H and C_6’_–H), 7.73 (t, 2H, *J* = 7.4 Hz, Phenyl C_4_ -H and C_4’_-H) 7.68 (d, 1H, *J* = 8.7 Hz, C_5_-H), 7.59–7.57 (m, 4H, Phenyl C_3_–H, C_5_–H, C_3’_–H and C_5’_–H), 6.85 (dd, 1H, *J*_*1*_ = 8.7 Hz, *J*_*2*_ = 2.0 Hz, C_6_-H), 6.78 (d, 1H, *J* = 2.0 Hz, C_8_–H), 6.28 (s, 1H, C_3_–H), 5.79 (s, 2H, COCH_2_), 5.60 (s, 2H, OCH_2_). ^**13**^**C NMR** (100 Hz, DMSO-*d*_*6*_), δ (ppm) 196.4 (C=O), 165.6 (C=O), 162.9 (C_2_=O), 160,6 (C_7_), 155.6 (C_4_), 150.9 (C_8a_), 134.3 (two Phenyl-C_4’_ and Phenyl-C_1’_), 133.10 (two Phenyl-C_1_ and Phenyl-C_4_), 129.9 (two Phenyl-C_2_ and C_6_), 129.5 (two Phenyl-C_2`_ and C_6`_), 129.3 (two Phenyl-C_3’_ and C_5’_), 128.4 (two Phenyl-C_3_ and C_5_), 126.6 (C_5_), 113.7 (C_6_), 109.7 (C_4a_), 108.8 (C_3_), 102.9 (C_8_), 70.0 (COCH_2_), 62.5 (OCH_2_). **MS** (m/z %): 414.14 (23.61, M^+^), 413.24 (6.94, M-H^+^), 105.12 (100). **Elemental analysis** for C_25_H_18_O_6_, calcd.: C, 72.46; H, 4.38; Found: C, 72.23; H, 4.57.

#### {7-[2-(4-Chlorophenyl)-2-oxoethoxy]-2-oxo-2H-chromen-4-yl}methyl benzoate (8)

White solid; Yield 51%; mp 245–247 °C. ^**1**^**H NMR** (400 MHz, DMSO-*d*_*6*_): δ 8.08 (d, 2H, *J* = 8.7 Hz, Phenyl C_2_–H and C_6_–H), 8.05 (d, 2H,* J* = 8.9 Hz, Phenyl C_2’_–H and C_6’_–H), 7.78 (d, 1H, *J* = 8.9 Hz, C_5_–H), 7.73 (t, 1H, *J* = 7.4 Hz, Phenyl C_4_–H), 7.66 (d, 2H, *J* = 8.6 Hz, Phenyl C_3’_–H, C_5’_–H), 7.61 (t, 2H, *J* = 7.7 Hz, Phenyl C_3_–H, C_5_–H,), 7.18 (d, 1H, *J* = 2.3 Hz, C_8_–H), 7.09 (dd, 1H, *J*_*1*_ = 8.6 Hz, *J*_*2*_ = 2.3 Hz, C_6_-H), 6.41 (s, 1H, C_3_-H), 5.81 (s, 2H, COCH_2_), 5.64 (s, 2H, OCH_2_). ^**13**^**C NMR** (100 Hz, DMSO-*d*_*6*_): δ (ppm): δ 194.2 (C=O), 164.8 (C=O), 161.8 (C_2_=O), 160.4 (C_7_), 155.4 (C_4_), 150.5 (C_8a_), 136.3 (Phenyl-C_4`_), 134.7 (Phenyl-C_1`_), 134.4 (Phenyl-C_1_), 131.8 (Phenyl-C_4_), 129.7 (two Phenyl-C_2`_and C_6`_), 129.3 (two Phenyl-C_2_ and C_6_), 128.4 (two Phenyl-C_3`_and C_5`_), 128.3 (two Phenyl-C_3_ and C_5_), 126.4 (C_5_), 113.4 (C_6_), 111.1 (C_4a_), 110.1 (C_3_), 102.4 (C_8_), 71.1 (COCH_2_), 62.8 (OCH_2_). **MS** (m/z %): 447.97 (12.78, M^+^), 450.63 (32.38, M^+^ + 2), 43.19 (100). **Elemental analysis** for C_25_H_17_ClO_6_, calcd.: C, 66.90; H, 3.82; Found: C, 67.12; H, 3.97.

#### [2-Oxo-7-(2-oxo-2-phenylethoxy)-2H-chromen-4-yl]methyl 4-chlorobenzoate (9)

White crystals; Yield 77%; mp 217–219 °C. ^**1**^**H NMR** (400 MHz, DMSO-*d*_*6*_): δ 8.10–8.07 (m, 4H, Phenyl C_2_–H, C_6_–H, C_2’_–H and C_6’_–H), 7.78 (d, 1H, *J* = 8.9 Hz, C_5_–H), 7.73 (t, 1H, *J* = 7.4 Hz, Phenyl C_4’_–H), 7.66 (d, 2H, *J* = 8.5 Hz, Phenyl C_3_–H and C_5_–H), 7.61 (t, 2H, *J* = 7.6 Hz, Phenyl C_3’_–H and C_5’_–H), 7.18 (d, 1H, *J* = 2.4 Hz, C_8_-H), 7.09 (dd, 1H, *J*_*1*_ = 8.9 Hz, *J*_*2*_ = 2.4 Hz, C_6_-H), 6.41 (s, 1H, C_3_–H), 5.81 (s, 2H, COCH_2_), 5.64 (s, 2H, OCH_2_). ^**13**^**C NMR** (100 Hz, DMSO-*d*_*6*_): δ 194.2 (C=O), 164.8 (C=O), 161.8 (C_2_=O), 160.4 (C_7_), 155.3 (C_4_), 150.5 (C_8a_), 139.2 (Phenyl-C_4_), 134.6 (Phenyl-C_4’_), 134.5 (Phenyl-C_1’_), 131.8 (Phenyl-C_2_), 131.8 (Phenyl-C_6_), 129.7 (Phenyl-C_2’_), 129.7 (Phenyl-C_6’_), 129.4 (Phenyl-C_3’_), 129.4 (Phenyl-C_5’_), 128.4 (Phenyl-C_3_), 128.4 (Phenyl-C_5_), 128.2 (Phenyl-C_1_), 126.4 (C_5_), 113.4 (C_6_), 111.1 (C_4a_), 110.1 (C_3_), 102.4 (C_8_), 71.1 (CH_2_), 62.8 (CH_2_). **MS** (m/z %): 448.41 (38.92, M^+^), 450.14 (22.31, M^+^ + 2), 447.68 (28.44, M^+^-1), 384.02 (100). **Elemental analysis** for C_25_H_17_ClO_6_, calcd.: C, 66.90; H, 3.82; Found: C, 66.83; H, 4.05.

#### {7-[2-(4-Chlorophenyl)-2-oxoethoxy]-2-oxo-2H-chromen-4-yl}methyl 4-chlorobenzoate (10)

Buff crystals*;* Yield 51%; mp 228–230 °C. ^**1**^**H NMR** (400 MHz, DMSO-*d*_*6*_): δ 8.09–8.07 (m, 4H, Phenyl C_2_–H, C_6_–H, C_2`_-H and C_6`_-H), 7.78 (d, 1H, *J* = 8.9 Hz, C_5_–H), 7.68–7.65 (m, 4H, Phenyl C_3_–H, C_5_–H, C_3’_–H and C_5’_–H), 7.20 (d, 1H, *J* = 2 Hz, C_8_–H), 7.09 (dd, 1H, *J*_1_ = 8.9, *J*_2_ = 2.0 Hz, C_6_–H), 6.41 (s, 1H, C_3_–H), 5.78 (s, 2H, COCH_2_), 5.64 (s, 2H, OCH_2_). ^**13**^**C NMR** (100 Hz, DMSO-*d*_*6*_): δ 193.3 (C=O), 164.8 (C=O), 161.8 (C_2_=O), 160.4 (C_7_), 155.6 (C_4_), 150.7 (C_8a_), 139.2 (two Phenyl-C_4_ and C_4’_), 131.8 (two Phenyl-C_2_ and C_6_), 130.4 (two Phenyl- C_2’_ and C_6’_), 129.6 (two Phenyl-C_3’_ and C_5’_), 129.4 (two Phenyl-C_3_ and C_5_), 128.3 (two Phenyl-C_1_ and C_1’_), 126.5 (C_5_), 113.9 (C_6_), 113.4 (C_4a_), 103.1 (C_8_), 102.4 (C_3_), 71.56 (CH_2_), 62.8 (CH_2_). **MS** (m/z %): 483.13 (35.54, M^+^), 485.01 (15.28, M^+^ + 2), 141.69 (100). **Elemental analysis** for C_25_H_16_Cl_2_O_6_, calcd.: C, 62.13; H, 3.34; Found: C, 61.96; H, 3.56.

#### [2-Oxo-7-(2-oxo-2-phenylethoxy)-2H-chromen-4-yl]methyl 2-methoxybenzoate (11)

White solid; Yield 45%; mp 220–222 °C. ^**1**^**H NMR** (400 MHz, DMSO-*d*_*6*_): δ 8.06 (d, 2H, *J* = 7.7 Hz, Phenyl C_2’_–H and C_6’_–H), 7.78 (d, 1H, *J* = 6.4 Hz, C_5_–H), 7.76 (d, 1H, *J* = 8.7 Hz, Phenyl C_6_–H), 7.73 (m, 1H, Phenyl C_4’_–H), 7.64–7.62 (m, 3H, Phenyl C_3’_–H and C_5’_–H and C_4’_H), 7.23 (d, 1H, *J* = 7.2 Hz, C_6_–H), 7.18 (s, 1H, C_8_-H), 7.09 (dd, 2H, *J*_*1*_ = 13.7, *J*_*2*_ = 6.5 Hz, Phenyl C_3_-H and C_5_-H), 6.46 (s, 1H, C_3_-H), 5.81 (s, 2H, COCH_2_), 5.60 (s, 2H, OCH_2_), 3.88 (s, 3H, OCH_3_). ^**13**^**C NMR** (100 Hz, DMSO-*d*_*6*_): δ (ppm) 194.2 (C=O), 165.6 (C=O), 161.7 (C_2_=O), 160.4 (C_7_), 158.9 (Phenyl-C_2_), 155.3 (C_4_), 151.0 (C_8a_), 134.7 (Phenyl-C_4_), 134.6 (Phenyl-C_4`_), 134.4 (phenyl-C_1`_), 131.6 (phenyl-C_6_), 129.3 (Phenyl-C_2`_), 129.3 (Phenyl-C_6`_), 128.4 (Phenyl-C_3`_), 128.4 (Phenyl-C_5`_), 126.4 (C_5_), 120.8 (Phenyl-C_1_), 119.4 (Phenyl-C_5_), 113.2 (Phenyl-C_3_), 113.1 (C_6_), 111.1 (C_4a_), 109.8 (C_3_), 102.4 (C_8_), 71 (COCH_2_), 62.4 (OCH_2_), 56.28 (OCH_3_). **MS** (m/z %): 444.46 (9.60, M^+^), 77.32 (100). **Elemental analysis** for C_26_H_20_O_7_, calcd.: C, 70.27; H, 4.54; Found: C, 70.44; H, 4.63.

#### {7-[2-(4-Chlorophenyl)-2-oxoethoxy]-2-oxo-2H-chromen-4-yl}methyl 2-methoxybenzoate (12)

White solid; Yield 51%; mp 218–220 °C. ^**1**^**H NMR** (400 MHz, DMSO-*d*_*6*_): δ 8.07 (d, 2H, *J* = 8.2 Hz, Phenyl C_2’_–H and C_6’_–H), 7.78 (d, 1H, *J* = 6.8 Hz, C_5_–H), 7.74 (d, 1H, *J* = 7.7 Hz, Phenyl C_6_–H), 7.69 (d, 2H, *J* = 8.3 Hz, Phenyl C_3’_–H and C_5’_–H), 7.62 (t, 1H, *J* = 8.0 Hz, Phenyl C_4_–H), 7.22 (d, 1H, *J* = 7.7 Hz, C_6_–H), 7.19 (s, 1H, C_8_–H), 7.09 (dd, 2H, *J*_1_ = 11.3 Hz, *J*_2_ = 7.7 Hz, Phenyl C_3_–H and C_5_–H), 6.46 (s, 1H, C_3_–H), 5.78 (s, 2H, COCH_2_), 5.60 (s, 2H, OCH_2_), 3.87 (s, 3H, OCH_3_). ^**13**^**C NMR** (100 Hz, DMSO-*d*_*6*_): δ (ppm) 193.3 (C=O), 165.6 (C=O), 161.6 (C_2_=O), 160.4 (C_7_), 158.9 (Phenyl-C_2`_), 155.3 (C_4_), 150.9 (C_8a_), 139.2 (Phenyl-C_4`_), 134.7 (Phenyl-C_4_), 133.3 (Phenyl-C_1`_), 131.7 (Phenyl-C_6_), 131.6 (Phenyl-C_2`_), 130.3 (Phenyl-C_6`_), 129.4 (Phenyl-C_3_), 126.5 (Phenyl-C_3`_), 126.4 (Phenyl-C_5`_), 120.8 (C_5_), 119.4 (Phenyl-C_1_), 113.2 (Phenyl-C_5_), 113.1 (C_6_), 111.1 (C_4a_), 109.8 (C_3_), 102.3 (C_8_), 71.1 (COCH_2_), 62.3 (OCH_2_), 56.2 (OCH_3_). **MS** (m/z %): 478.24 (8.62, M^+^), 479.23 (18.46, M^+^ + 1), 480.20 (12.17, M^+^ + 2), 235.15 (100). **Elemental analysis** for C_26_H_19_ClO_7_, calcd.: C, 65.21; H, 4.00; Found: C, 65.03; H, 4.19.

#### General procedure for the preparation of (7-acetoxy-2-oxo-2H-chromen-4-yl)methyl benzoate derivatives (13–15)

The appropriate **2–4** derivative (1.5 mmol) was added to a mixture of acetic anhydride and acetic acid (1:1, 10 mL). The reaction mixture was refluxed for 12 h, cooled and poured onto ice. The crude off-white solid was then filtered, washed with water, and dried. The obtained solid was recrystallized from ethanol.

#### (7-Acetoxy-2-oxo-2H-chromen-4-yl)methyl benzoate (13)

White solid; Yield 71%; mp 192–194 °C. ^**1**^**H NMR** (400 MHz, DMSO-*d*_*6*_): δ 8.09 (d, 2H, *J* = 7.3 Hz, Phenyl C_2_–H and C_6_–H), 7.92 (d, 1H, *J* = 8.6 Hz, C_5_–H), 7.73 (t, 1H, *J* = 7.1 Hz, Phenyl C_4_–H), 7.59 (t, 2H, *J* = 7.4 Hz, Phenyl C_3_–H and C_5_–H), 7.37 (s, 1H, C_8_–H), 7.25 (d, 1H, *J* = 8.5 Hz, C_6_–H), 6.56 (s, 1H, C_3_–H), 5.66 (s, 2H, OCH_2_), 2.33 (s, 3H, COCH_3_). ^**13**^**C NMR** (100 Hz, DMSO-*d*_*6*_): δ (ppm) 169.2 (C=O), 165.5 (C=O), 159.8 (C_2_=O), 154.2 (C_7_), 153.6 (C_4_), 150.2 (C_8a_), 134.3 (phenyl-C_4_), 129.9 (two Phenyl-C_2_ and C_6_), 129.4 (two Phenyl-C_3_ and C_5_), 129.3 (phenyl-C_1_), 126.4 (C_5_), 119.2 (C_6_), 115.3 (C_4a_), 112.7 (C_8_), 110.9 (C_3_), 62.5 (OCH_2_), 21.3 (COCH_3_). **MS** (m/z %): 338.55 (24.52, M^+^), 337.37 (18.27, M^+^-1), 280.45 (100). **Elemental analysis** for C_19_H_14_O_6_, calcd.: C, 67.45; H, 4.17; Found: C, 67.63; H, 4.39.

#### (7-Acetoxy-2-oxo-2H-chromen-4-yl)methyl 4-chlorobenzoate (14).

Off-White solid; Yield 88%; mp 216–218 °C. ^**1**^**H NMR** (400 MHz, DMSO-*d*_*6*_): δ 8.09 (d, 2H, *J* = 8.2 Hz, Phenyl C_2_–H and C_6_-H), 7.92 (d, 1H, *J* = 7.0 Hz, C_5_–H), 7.67 (d, 2H, *J* = 6.6 Hz, Phenyl C_3_–H and C_5_–H), 7.37 (s, 1H, C_8_–H), 7.25 (d, 1H, *J* = 5.2 Hz, C_6_–H), 6.60 (s, 1H, C_3_–H), 5.67 (s, 2H, OCH_2_), 2.34 (s, 3H, COCH_3_). ^**13**^**C NMR** (100 Hz, DMSO-*d*_*6*_): δ (ppm) 169.4 (C=O), 165.4 (C=O), 159.4 (C_2_=O), 154.3 (C_7_), 153.6 (C_4_), 149.9 (C_8a_), 134.6 (phenyl-C_4_), 131.8 (two Phenyl-C_2_ and C_6_), 129.6 (two Phenyl-C_3_ and C_5_), 128.2 (phenyl-C_1_), 126.4 (C_5_), 119.2 (C_6_), 115.5 (C_4a_), 112.8 (C_8_), 110.9 (C_3_), 62.7 (OCH_2_), 21.4 (COCH_3_). **MS** (m/z %): 372.14 (43.76, M^+^), 374.07 (63.76, M^+^ + 2), 312.09 (100). **Elemental analysis** for C_19_H_13_ClO_6_, calcd C, 61.22; H, 3.52; Found C, 61.39; H, 3.68.

#### (7-Acetoxy-2-oxo-2H-chromen-4-yl)methyl 2-methoxybenzoate (15).

White solid; Yield 74%. mp 186–188 °C. ^**1**^**H NMR** (400 MHz, DMSO-*d*_*6*_): δ 7.89 (d, 1H, *J* = 8.5 Hz, Phenyl C_6_–H), 7.79 (d, 1H, *J* = 6.7 Hz, C_5_–H), 7.62 (t, 1H, *J* = 7.5 Hz, Phenyl C_4_–H), 7.35 (s, 1H, C_8_–H), 7.25 (d, 1H, *J* = 7.9 Hz, C_6_–H), 7.22 (d, 1H, *J* = 8.8 Hz, Phenyl C_3_–H), 7.08 (t, 1H, *J* = 7.4 Hz, Phenyl C_5_–H), 6.63 (s, 1H, C_3_–H), 5.62 (s, 2H, OCH_2_), 3.88 (s, 3H, OCH_3_), 2.33 (s, 3H, COCH_3_). ^**13**^**C NMR** (100 Hz, DMSO-*d*_*6*_): δ (ppm) 169.3 (C=O), 165.6 (C=O), 158.9 (C_2_=O), 155.5 (phenyl-C_2_), 154.2 (C_7_), 153.5 (C_4_), 150.2 (C_8a_), 134.7 (phenyl-C_4_), 131.6 (Phenyl C_6_), 126.5 (Phenyl-C_1_), 126.4 (phenyl-C_5_), 120.7 (C_5_), 119.3 (C_6_), 115.2 (C_4a_), 113.5 (phenyl-C_3_), 113.1 (C_8_), 109.6 (C_3_), 62.5 (OCH_2_), 56.2 (OCH_3_), 21.3 (COCH_3_). **MS** (m/z %): 368.36 (6.80, M^+^), 44.13 (100). **Elemental analysis** for C_20_H_16_O_7_, calcd.: C, 65.22; H, 4.38; Found C, 65.41; H, 4.45.

### Biological tests

#### In vitro* cytotoxic activity using MTT assay*

In this study, all synthesized compounds were initially screened for their in *vitro* antitumor activities against two human BC cell lines, namely, MCF-7 (estrogen positive) and MDA-MB-231(triple negative), to select the most promising ones for further screenings via MTT assay using Doxorubicin, as a reference drug as described in the reported method^46,47^.

#### In vitro* (EGFR) enzyme inhibition assay*

Fourteen compounds that showed good cytotoxic activity against the TNBC cell line (MDA-MB-231) were examined for in vitro EGFR inhibition assay relative to erlotinib as a reference drug according to the reported protocol^48^.

#### In vitro* (ARO) enzyme inhibition assay*

Eleven compounds that showed good cytotoxic activity against the ER + cell line (MCF-7) were examined for in vitro ARO inhibition assay relative to EXM as a reference drug according to the reported method^49^.

#### Cell cycle arrest

DNA flow cytometric cell cycle analysis was performed on treated MCF-7 cells with IC_50_ concentration of compound **8** and treated MDA-MB-231 cells with IC_50_ concentration of compounds **10**, **12** and **14** according to the reported procedure^50^.

#### Apoptosis assay

Annexin V-FITC/PI dual staining assay was performed according to the reported method^51^ to further study the ability of compounds **8**, **10**, **12** and **14** to induce apoptosis by introducing flow cytometry-based analysis.

#### Measurement of the level of Bax Bcl-2 and Caspase-9

MCF-7 cells were treated with compound **8,** while MDA-MB-231 cells were exposed to compounds **10**, **12** and **14** according to the manufacturer's instructions^52^.

### Molecular docking study with both EGFR and human ARO enzymes

Crystal structures of EGFR (PDB ID: 1M17)^18^ and ARO (PDB ID: 3EQM)^53^ were obtained from the PDB. Regarding enzyme inhibition assay, the most potent compounds were selected for docking study using Molecular Operating Environment (MOE) software.

### Supplementary Information

Supplementary Information 1.

Supplementary Information 2.

## Data Availability

All study data are presented in this article and its supplementary information files.
