# Supplementary material for: Developing multitarget coumarin based anti-breast cancer agents: synthesis and molecular modeling study
Source: Sci Rep. 2023 Aug 17;13:13370. doi: 10.1038/s41598-023-40232-3 (PMC10435442; doi:10.1038/s41598-023-40232-3)
Supplement: Supplementary file 1 — Supplementary Information 1. [file 41598_2023_40232_MOESM1_ESM.docx]

**Developing Multitarget Coumarin Based Anti-Breast Cancer Agents: Synthesis, Molecular Modeling Study**

## Fiby N. Takla,^1,2^ Waleed A. Bayoumi,^1^ Shahenda M. El-Messery,^1^*^*^* Magda N.

**A. Nasr ^1^**

*^1^Department of Pharmaceutical Organic Chemistry, Faculty of Pharmacy, Mansoura University, Mansoura 35516, Egypt*

*^2^Department of Pharmaceutical Chemistry, Faculty of Pharmacy, Delta University for Science and Technology, International Coastal Road, Gamasa City, 35712, Egypt*

**Corresponding author:* Prof. Shahenda M. El-Messery Department of Pharmaceutical

Organic Chemistry, Faculty of Pharmacy, Mansoura University, P.O.Box 35516, Mansoura,

Egypt.

E- mail , [habib2001@mans.edu.eg](mailto:habib2001@mans.edu.eg) Tel, Fax: +20-50-220024

## Supplementary figures and tables:

**S1.** Bar chart Representation of EGFR inhibition IC_50_ of the tested Compound

**S2.** Bar chart Representation of ARO inhibition IC_50_ of the tested Compounds .

##### **S3**. Bar chart representation of cell cycle arrest of MCF-7 cells treated with compound **8** and MDA-MB-231 cells treated with compounds 10, 12 and 14 expressed as % of cells in each phase of the cell cycle.

##### **S4.** Bar chart showing % of cells in early, late apoptotic, and necrotic stages for compound **8** in MCF-7 cells and compounds **10**, **12** and **14** in MDA-MB-231 cells compared to control untreated cells


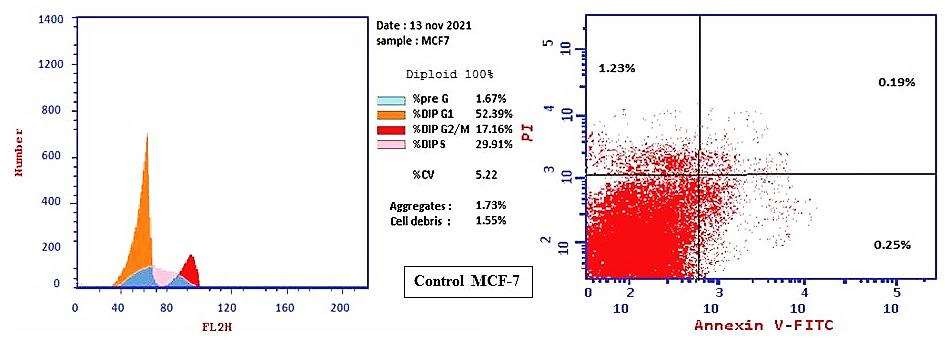


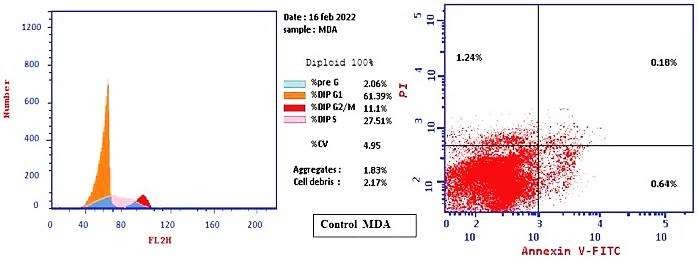


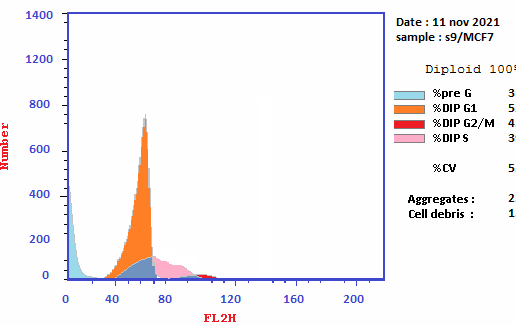


**8 / MCF-7**


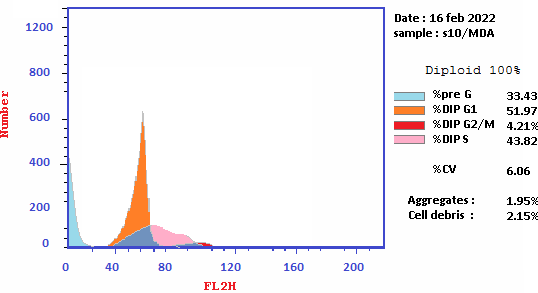


**10 / MDA**


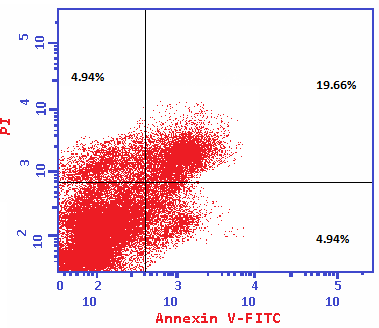


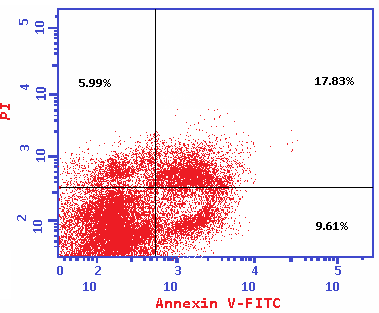


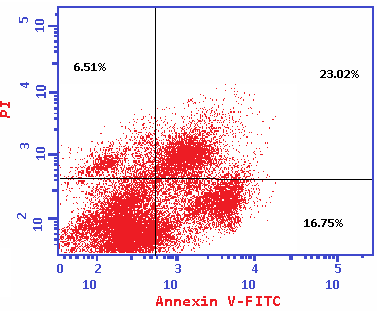


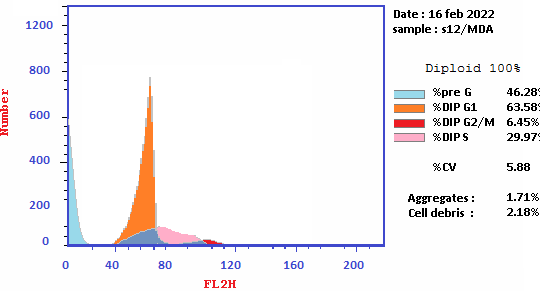


**12 / MDA**

**
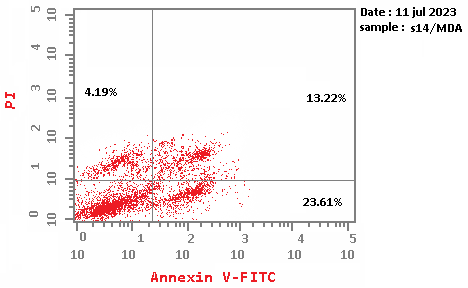

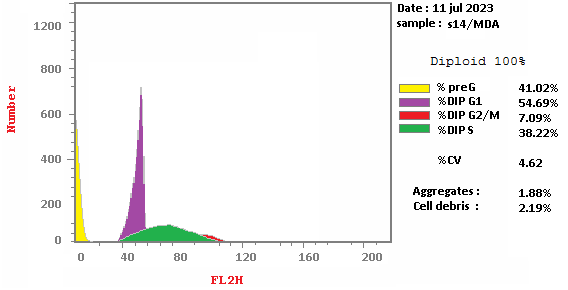
**

#####

**14/MDA**

##### **S5**. Results of cell cycle analysis & annexin V-FTIC / PI dual staining assay for MCF-7 and MDA-MB-231 shows annexin V-FITC staining on the x-axis and PI staining on the Y-axis

| Compound No. | Bax  Conc (ng/ml) | FLD | Bcl-2  Conc (ng/ml) | FLD |
| --- | --- | --- | --- | --- |
| **8/MCF7** | 0.164±0.0056^acd^ | 5.27 | 3.291±0.36^a^ | 0.44 |
| **Control MCF7** | 0.031±0.0014^abe^ | 1 | 7.438±0.08^bcd^ | 1 |
| **10/MDA-MB-231** | 0.249±0.0079^be^ | 3.6 | 4.088±0.058^cf^ | 0.377 |
| **12/MDA-MB-231** | 0.36±0.0118^cef^ | 5.22 | 2.934±0.154^be^ | 0.271 |
| **14/MDA-MB-231** | 0.292±0.0106^ghi^ | 4.9 | 2.984±0.12^gh^ | 0.329 |
| **Control MDA-MB-231** | 0.069±0.0032^dfe^ | 1 | 10.83±0.046^adef^ | 1 |
| **p value** | <0.001* |  | <0.001* |  |

Similar superscripted small letters in same column denote significant difference between groups by post HOC Tukey test

**S6.** Bax/Bcl-2 levels in (ng/ml) in MCF-7 cells treated with compound **8** and MDA-MB-231 cells treated with compounds **10** ,**12, 14** and control untreated cells.

##### **S7**. Bar chart representations of Bax (A) /Bcl-2 (B) levels in MCF-7 cells treated with compound **8** and MDA-MB-231 cells treated with compounds **10**, **12, 14** and control untreated cells.

| Compound No. | Caspase-9 Conc. (ng/ml) | FLD |
| --- | --- | --- |
| **8/MCF7** | 30.39±2.56^abc^ | 6.71 |
| **Control MCF7** | 4.528±0.61^ad^ | 1 |
| **10/MDA-MB-231** | 16.77±0.37^be^ | 6.42 |
| **12/MDA-MB-231** | 19.16±0.41^df^ | 7.34 |
| **14/MDA-MB-231** | 18.97±0.62^ghi^ | 4.37 |
| **Control MDA-MB-231** | 2.611±0.19^cef^ | 1 |
| **Pvalue** | <0.001* |  |

Similar superscripted small letters in same column denote significant difference between groups by post HOC Tukey test

**S8**. Caspase-9 protein levels in (ng/ml) in MCF-7 cells treated with compound **8** and MDA- MB-231 cells treated with compounds **10** , **12, 14** and control untreated cells.

##### **S9.** Bar chart representation of Caspase-9 protein levels in (ng/ml) in MCF-7 cells treated with compound **8** and MDA-MB-231 cells treated with compounds **10, 12** , **14** and control untreated cells.


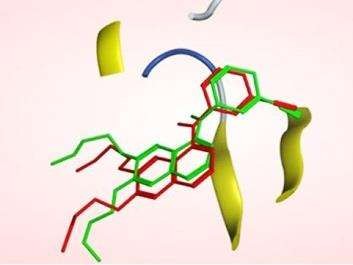


**S10**. Binding mode of the re-docked ligand (red) per imposed on the same position as the native ligand (green), showing the same orientation


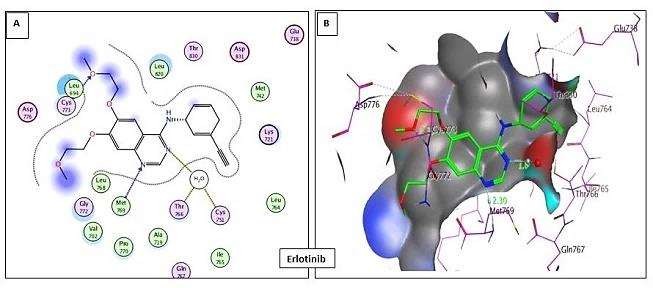


##### **S11**. 2D and 3D binding modes of erlotininb into the active site of EGFR (PDB ID: 1 M17).


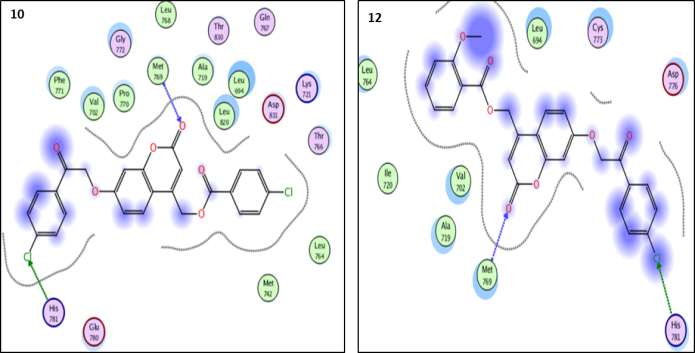


**S12**. 2D binding modes of compounds **10 and 12** into the active site of EGFR (PDB ID: 1M17).


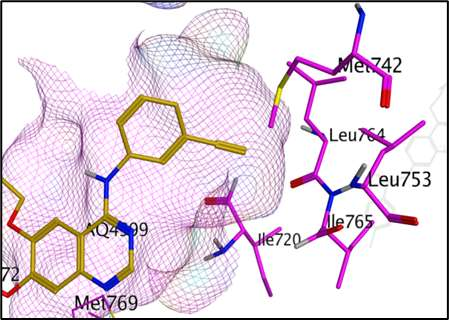


##### **S13.** Ethynyl phenyl moiety of Erlotinib (yellow) showed hydrophobic interactions with Leu 764, Leu 753, Met 742, Ile 720 and Ile 765 (part of the hydrophobic pocket) presented as (pink, stick).


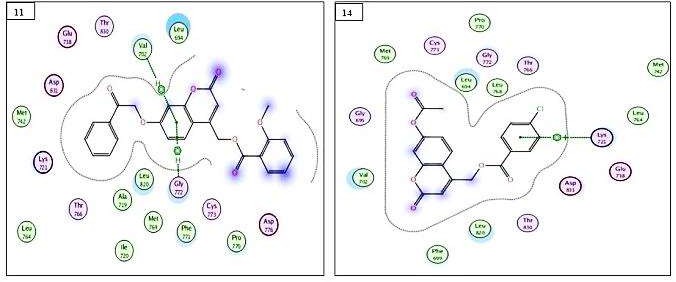


**S14.** Compounds **11** and **14** with poor binding with EGFR active site (PDB: ID: 1M17


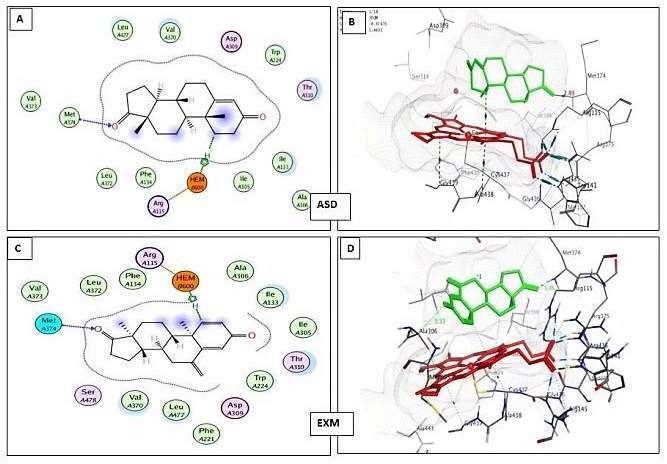


##### **S15.** . 2D and 3D binding modes of ASD, EXM inside ARO (PDB ID: 3EQM) binding pocket


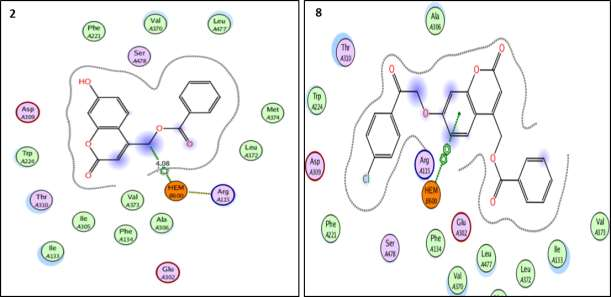


**S16.** 2D binding modes of compounds **2** and **8** inside ARO (PDB ID: 3EQM) binding pocket


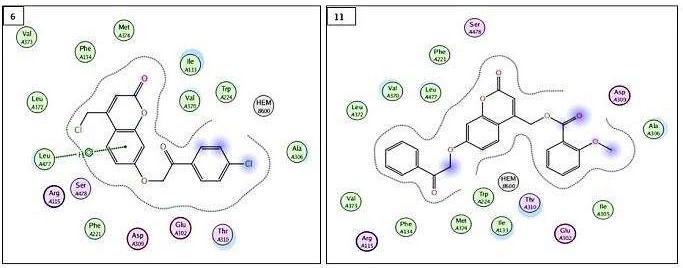


##### **S17.** Compounds **6** and **11** with poor binding with ARO active site (PDB ID: 3EQM)


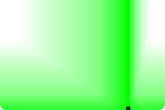

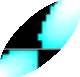

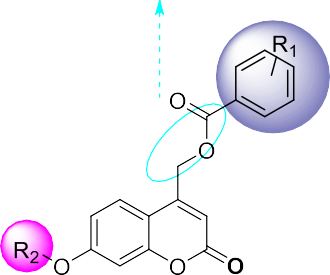

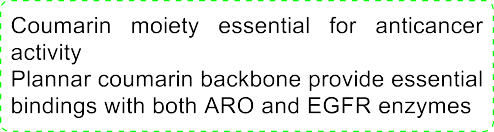

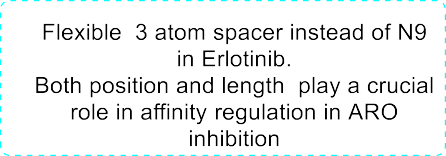

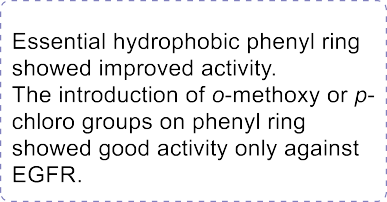

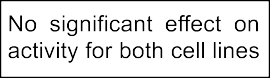

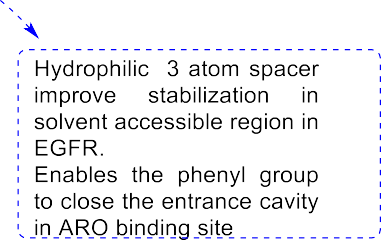

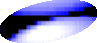

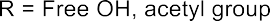

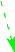

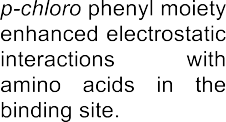

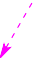

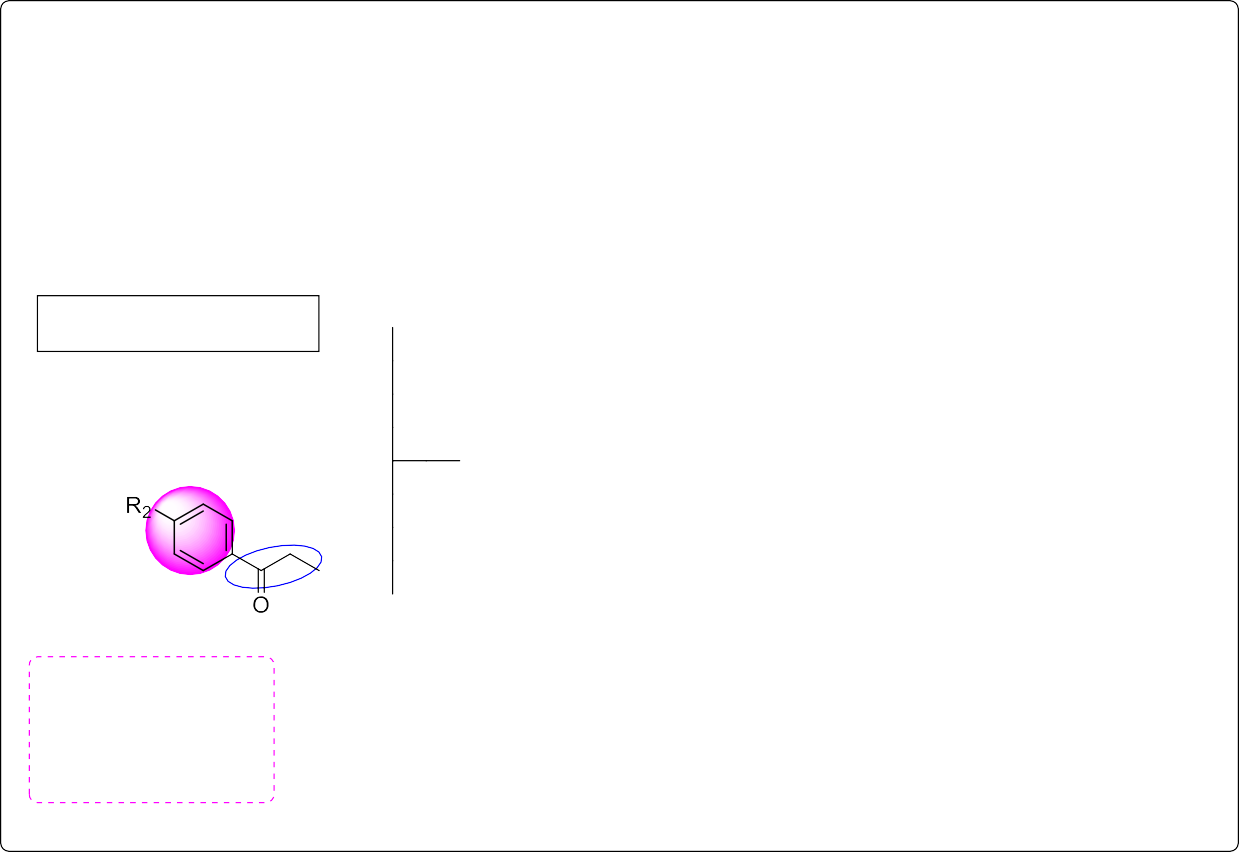


**S18.** SAR study of the newly designed 4,7-disubstituted coumarin derivatives as a potent anti- breast cancer agent

## 1. Supplementary biology

Researcher

Assay Samples Cell lines Ref.

Date Reader Kit used

Solvent : DMSO

: Dr.Feby Nabil

: MTT cytotoxicity assay

: 16 samples.

: ----

: ----

: 14-08-2021

: BIOLINE ELIZA READER

: SIGMA chemicals

email:

mob. 01224037280

wl 450 nm

*Cytotoxicity results

# Lab Report

| Ser | Sample | | cytotoxicity_IC50 ug/ml | | | SD  ± |
| --- | --- | --- | --- | --- | --- | --- |
|  | code | M.W  g/mol | **mcf7** | **MDA** | **MCF10a** |  |
| 1 | **1** |  | 24.3±1.47 | 74.5±4.06 | 93±4.53 |  |
| 2 | **2** |  | 1.78±0.11 | 15.4±0.84 | 55±2.68 |  |
| 3 | **3** |  | 12.1±0.74 | 22.3±1.22 | 122±5.93 |  |
| 4 | **4** |  | 5.6±0.34 | 2.08±0.11 | 41.6±2.03 |  |
| 5 | **5** |  | 13±0.79 | 1.91±0.1 | 27.6±1.35 |  |
| 6 | **6** |  | 78.7±4.77 | 20.5±1.12 | 56.6±2.76 |  |
| 7 | **7** |  | 32.1±1.94 | 23.5±1.28 | 41.9±2.04 |  |
| 8 | **8** |  | 2.62±0.16 | 12.3±0.67 | 35.8±1.74 |  |
| 9 | **9** |  | 45±2.73 | 4.65±0.25 | 135±6.56 |  |
| 10 | **10** |  | 46.3±2.81 | 1.09±0.06 | 37.1±1.81 |  |
| 11 | **11** |  | 58.1±3.52 | 27.2±1.48 | 79.2±3.86 |  |
| 12 | **12** |  | 6.33±0.38 | 1.71±0.09 | 34.5±1.68 |  |
| 13 | **13** |  | 37.6±2.28 | 10.4±0.56 | 166±8.1 |  |
| 14 | **14** |  | 106±6.41 | 0.64±0.03 | 97.9±4.77 |  |
| 15 | **15** |  | 6.3±0.38 | 27.9±1.52 | 115±5.62 |  |
| *** | **Doxorubicin** | --- | 3.04±0.18 | 3.98±0.22 | 12.4±0.6 |  |

## Cell culture Protocol

Cell Line cells were obtained from American Type Culture Collection , cells were cultured using DMEM (Invitrogen/Life Technologies) supplemented with 10% FBS (Hyclone,), 10 ug/ml of insulin (Sigma), and 1% penicillin- streptomycin. All of the other chemicals and reagents were from Sigma, or Invitrogen.

Plate cells (cells density 1.2 – 1.8 × 10,000 cells/well) in a volume of 100µl complete growth medium + 100 ul of the tested compound per well in a 96-well plate for 24 hours before the MTT assay .

Cell cuture protocol

1. Remove culture medium to a centrifuge tube.
2. Briefly rinse the cell layer with 0.25% (w/v) Trypsin 0.53 mM EDTA solution to remove all traces of serum which contains Trypsin inhibitor.
3. Add 2.0 to 3.0 ml of Trypsin EDTA solution to flask and observe cells under an inverted microscope until cell layer is dispersed (usually within 5 to 15 minutes).

Note: To avoid clumping do not agitate the cells by hitting or shaking the flask while waiting for the cells to detach. Cells that are difficult to detach may be placed at 37°C to facilitate dispersal.

1. Add 6.0 to 8.0 mL of complete growth medium and aspirate cells by gently pipetting.
2. Transfer the cell suspension to the centrifuge tube with the medium and cells from step 1, and centrifuge at approximately 125 xg for 5 to 10 minutes. Discard the supernatant.
3. Resuspend the cell pellet in fresh growth medium. Add appropriate aliquots of the cell suspension to new culture vessels.
4. Incubate cultures at 37°C for 24 hrs.

8-After treatment of cells with the serial concentrations of the compound to be tested incubation is carried out for 48 h at 37ºC ,then the plates are to be examined under the inverted microscope and proceed for the MTT assay

MTT – Cytotoxicity assay protocol The MTT method of monitoring in vitro cytotoxicity is

well suited for use with multiwell plates. For best results, cells in the

log phase of growth should be employed and final cell number should not exceed 106 cells/cm2. Each test should include a blank containing complete medium without cells.

1. Remove cultures from incubator into laminar flow hood or other sterile work area.
2. Reconstitute each vial of MTT [M-5655] to be used with 3 ml of medium or balanced salt solution without phenol red and serum. Add reconstituted MTT in an amount equal to 10% of the culture medium volume.
3. Return cultures to incubator for 2-4 hours depending on cell type and maximum cell density. (An incubation period of 2 hours is generally adequate but may be lengthened for low cell densities or cells with lower metabolic activity.) Incubation times should beconsistent when making comparisons.
4. After the incubation period, remove cultures from incubator and dissolve the resulting formazan crystals by adding an amount of MTT Solubilization Solution [M-8910] equal to the original culture medium volume.
5. Gentle mixing in a gyratory shaker will enhance dissolution. Occasionally, especially in dense cultures, pipetting up and down [trituration] may be required to completely dissolve the MTT formazan crystals.
6. Spectrophotometrically measure absorbance

at a wavelength of 570 nm. Measure the background absorbance of multiwell plates at 690 nm and subtract from the 450 nm measurement. Tests performed in multiwell plates can be read using the appropriate type of plate

reader or the contents of individual wells may be transferred to appropriate size cuvets for spectrophotometric measurement


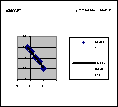

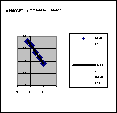


researcher assay Date cells

Dr.Feby Nabil MTT 15/08/2021

|  | **Blank** | **CC** | **Sample No. 1/MCF7** | | | | |
| --- | --- | --- | --- | --- | --- | --- | --- |
|  | **1** | **2** | **3** | **4** | **5** | **6** | **7** |
| A | B | C | 100ug | 25ug | 6.3ug | 1.6ug | 0.4ug |
| B | B | C | 100ug | 25ug | 6.25ug | 1.6ug | 0.4ug |
| C | B | C | 100ug | 25ug | 6.25ug | 1.6ug | 0.4ug |

ROBONIK P2000 Eia reader Wave

length: 450 nm

Reference: 630 nm

|  | **1** | **2** | **3** | **4** | **5** | **6** | **7** |
| --- | --- | --- | --- | --- | --- | --- | --- |

| A | 0.001 | 0.541 | 0.221 | 0.264 | 0.316 | 0.364 | 0.418 |
| --- | --- | --- | --- | --- | --- | --- | --- |
| B | 0.003 | 0.529 | 0.208 | 0.255 | 0.323 | 0.353 | 0.404 |
| C | 0.001 | 0.534 | 0.231 | 0.267 | 0.318 | 0.362 | 0.425 |
| mean | 0.002 | 0.535 | 0.22 | 0.262 | 0.319 | 0.3597 | 0.4157 |
| %  viability |  |  | 41.1471 | 49.002 | 59.663 | 67.269 | 77.743 |

1/MCF7


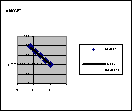


| 2 | 41.15 |
| --- | --- |
| 1.4 | 49 |
| 0.8 | 59.66 |
| 0.19 | 67.27 |
| -0.41 | 77.74 |
| IC50= | |

|  | **Blank** | **CC** | **Sample No. 2/MCF7** | | | | | **Sample No. 3/MCF7** | | | | |
| --- | --- | --- | --- | --- | --- | --- | --- | --- | --- | --- | --- | --- |
|  | **1** | **2** | **3** | **4** | **5** | **6** | **7** | **8** | **9** | **10** | **11** | **12** |
| A | B | C | 100ug | 25ug | 6.3ug | 1.6ug | 0.4ug | 100ug | 25ug | 6.3ug | 1.6ug | 0.4ug |
| B | B | C | 100ug | 25ug | 6.25ug | 1.6ug | 0.4ug | 100ug | 25ug | 6.25ug | 1.6ug | 0.4ug |
| C | B | C | 100ug | 25ug | 6.25ug | 1.6ug | 0.4ug | 100ug | 25ug | 6.25ug | 1.6ug | 0.4ug |

ROBONIK P2000 Eia reader Wave

length: 450 nm

Reference: 630 nm

|  | **1** | **2** | **3** | **4** | **5** | **6** | **7** | **8** | **9** | **10** | **11** | **12** |
| --- | --- | --- | --- | --- | --- | --- | --- | --- | --- | --- | --- | --- |

| A | 0.001 | 0.541 | 0.082 | 0.175 | 0.242 | 0.279 | 0.311 | 0.216 | 0.242 | 0.291 | 0.346 | 0.402 |
| --- | --- | --- | --- | --- | --- | --- | --- | --- | --- | --- | --- | --- |
| B | 0.003 | 0.528 | 0.141 | 0.183 | 0.208 | 0.283 | 0.328 | 0.185 | 0.257 | 0.276 | 0.358 | 0.386 |
| C | 0.001 | 0.551 | 0.118 | 0.176 | 0.227 | 0.288 | 0.313 | 0.177 | 0.239 | 0.303 | 0.351 | 0.379 |
| mean | 0.002 | 0.54 | 0.11367 | 0.178 | 0.2257 | 0.2833 | 0.3173 | 0.1927 | 0.246 | 0.29 | 0.352 | 0.389 |
| %  viability |  |  | 21.0494 | 32.963 | 41.79 | 52.469 | 58.765 | 35.679 | 45.5556 | 53.704 | 65.12 | 72.04 |

2/MCF7 3/MCF7

| 2 | 21.05 |  |
| --- | --- | --- |
| 1.4 | 32.96 |  |
| 0.8 | 41.79 |  |
| 0.19 | 52.47 |  |
| -0.41 | 58.77 |  |
| IC50= | |  |

| 2 | 35.679 |  |
| --- | --- | --- |
| 1.3979 | 45.5556 |  |
| 0.7959 | 53.7037 |  |
| 0.1931 | 65.1235 |  |
| -  0.4089 | 72.037 |  |
| IC50= | |  |

|  | **Blank** | **CC** | **Sample No. 4/MCF7** | | | | | **Sample No. 6/MCF7** | | | | |
| --- | --- | --- | --- | --- | --- | --- | --- | --- | --- | --- | --- | --- |
|  | **1** | **2** | **3** | **4** | **5** | **6** | **7** | **8** | **9** | **10** | **11** | **12** |
| A | B | C | 100ug | 25ug | 6.3ug | 1.6ug | 0.4ug | 100ug | 25ug | 6.3ug | 1.6ug | 0.4ug |
| B | B | C | 100ug | 25ug | 6.25ug | 1.6ug | 0.4ug | 100ug | 25ug | 6.25ug | 1.6ug | 0.4ug |
| C | B | C | 100ug | 25ug | 6.25ug | 1.6ug | 0.4ug | 100ug | 25ug | 6.25ug | 1.6ug | 0.4ug |

ROBONIK P2000 Eia reader Wave

length: 450 nm

Reference: 630 nm

|  | **1** | **2** | **3** | **4** | **5** | **6** | **7** | **8** | **9** | **10** | **11** | **12** |
| --- | --- | --- | --- | --- | --- | --- | --- | --- | --- | --- | --- | --- |

| A | 0.001 | 0.495 | 0.153 | 0.201 | 0.247 | 0.293 | 0.351 | 0.241 | 0.289 | 0.354 | 0.411 | 0.454 |
| --- | --- | --- | --- | --- | --- | --- | --- | --- | --- | --- | --- | --- |
| B | 0.003 | 0.525 | 0.161 | 0.194 | 0.253 | 0.297 | 0.346 | 0.233 | 0.303 | 0.369 | 0.393 | 0.444 |
| C | 0.001 | 0.507 | 0.158 | 0.199 | 0.256 | 0.305 | 0.347 | 0.228 | 0.316 | 0.375 | 0.388 | 0.439 |
| mean | 0.002 | 0.509 | 0.15733 | 0.198 | 0.252 | 0.2983 | 0.348 | 0.234 | 0.30267 | 0.366 | 0.397 | 0.446 |
| %  viability |  |  | 30.9103 | 38.9 | 49.509 | 58.612 | 68.369 | 45.972 | 59.463 | 71.906 | 78.06 | 87.56 |

4/MCF7 6/MCF7


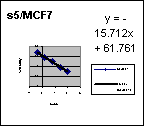


IC50=


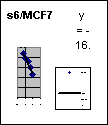


| 2 | 30.91 |
| --- | --- |
| 1.4 | 38.9 |
| 0.8 | 49.51 |
| 0.19 | 58.61 |
| -0.41 | 68.37 |

| 2 | 45.9725 |
| --- | --- |
| 1.3979 | 59.463 |
| 0.7959 | 71.9057 |
| 0.1931 | 78.0616 |
| -  0.4089 | 87.5573 |

IC50=

|  | **Blank** | **CC** | **Sample No. 5/MCF7** | | | | | **Sample No. 7/MCF7** | | | | |
| --- | --- | --- | --- | --- | --- | --- | --- | --- | --- | --- | --- | --- |
|  | **1** | **2** | **3** | **4** | **5** | **6** | **7** | **8** | **9** | **10** | **11** | **12** |
| A | B | C | 100ug | 25ug | 6.3ug | 1.6ug | 0.4ug | 100ug | 25ug | 6.3ug | 1.6ug | 0.4ug |
| B | B | C | 100ug | 25ug | 6.25ug | 1.6ug | 0.4ug | 100ug | 25ug | 6.25ug | 1.6ug | 0.4ug |
| C | B | C | 100ug | 25ug | 6.25ug | 1.6ug | 0.4ug | 100ug | 25ug | 6.25ug | 1.6ug | 0.4ug |

ROBONIK P2000 Eia reader Wave

length: 450 nm

Reference: 630 nm

|  | **1** | **2** | **3** | **4** | **5** | **6** | **7** | **8** | **9** | **10** | **11** | **12** |
| --- | --- | --- | --- | --- | --- | --- | --- | --- | --- | --- | --- | --- |

| A | 0.001 | 0.551 | 0.208 | 0.261 | 0.297 | 0.352 | 0.372 | 0.231 | 0.288 | 0.333 | 0.401 | 0.447 |
| --- | --- | --- | --- | --- | --- | --- | --- | --- | --- | --- | --- | --- |
| B | 0.001 | 0.539 | 0.197 | 0.257 | 0.282 | 0.353 | 0.369 | 0.228 | 0.292 | 0.342 | 0.391 | 0.455 |
| C | 0.001 | 0.545 | 0.218 | 0.254 | 0.285 | 0.346 | 0.363 | 0.209 | 0.283 | 0.347 | 0.384 | 0.462 |

| mean | 0.001 | 0.545 | 0.20767 | 0.2573 | 0.288 | 0.3503 | 0.368 | 0.2227 | 0.28767 | 0.3407 | 0.392 | 0.455 |
| --- | --- | --- | --- | --- | --- | --- | --- | --- | --- | --- | --- | --- |
| %  viability |  |  | 38.104 | 47.217 | 52.844 | 64.281 | 67.523 | 40.856 | 52.7829 | 62.508 | 71.93 | 83.43 |


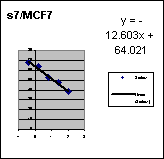

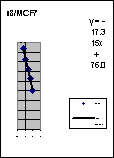
5/MCF7 7/MCF7

| log conc. | %  viability |
| --- | --- |
| 2 | 38.1 |
| 1.4 | 47.22 |
| 0.8 | 52.84 |
| 0.19 | 64.28 |
| -0.41 | 67.52 |

| log conc. | %  viability |
| --- | --- |
| 2 | 40.8563 |
| 1.3979 | 52.7829 |
| 0.7959 | 62.5076 |
| 0.1931 | 71.9266 |
| -  0.4089 | 83.4251 |

IC50=

IC50=

|  | **Blank** | **CC** | **Sample No. 8/MCF7** | | | | | **Sample No. 9/MCF7** | | | | |
| --- | --- | --- | --- | --- | --- | --- | --- | --- | --- | --- | --- | --- |
|  | **1** | **2** | **3** | **4** | **5** | **6** | **7** | **8** | **9** | **10** | **11** | **12** |
| A | B | C | 100ug | 25ug | 6.3ug | 1.6ug | 0.4ug | 100ug | 25ug | 6.3ug | 1.6ug | 0.4ug |
| B | B | C | 100ug | 25ug | 6.25ug | 1.6ug | 0.4ug | 100ug | 25ug | 6.25ug | 1.6ug | 0.4ug |
| C | B | C | 100ug | 25ug | 6.25ug | 1.6ug | 0.4ug | 100ug | 25ug | 6.25ug | 1.6ug | 0.4ug |

ROBONIK P2000 Eia reader Wave

length: 450 nm

Reference: 630 nm

|  | **1** | **2** | **3** | **4** | **5** | **6** | **7** | **8** | **9** | **10** | **11** | **12** |
| --- | --- | --- | --- | --- | --- | --- | --- | --- | --- | --- | --- | --- |

| A | 0.001 | 0.577 | 0.164 | 0.212 | 0.264 | 0.314 | 0.357 | 0.261 | 0.321 | 0.367 | 0.414 | 0.462 |
| --- | --- | --- | --- | --- | --- | --- | --- | --- | --- | --- | --- | --- |
| B | 0.003 | 0.591 | 0.155 | 0.227 | 0.255 | 0.326 | 0.366 | 0.258 | 0.322 | 0.366 | 0.422 | 0.449 |
| C | 0.001 | 0.606 | 0.167 | 0.203 | 0.252 | 0.323 | 0.369 | 0.266 | 0.319 | 0.371 | 0.419 | 0.458 |
| mean | 0.002 | 0.591 | 0.162 | 0.214 | 0.257 | 0.321 | 0.364 | 0.2617 | 0.32067 | 0.368 | 0.418 | 0.456 |
| %  viability |  |  | 27.3957 | 36.189 | 43.461 | 54.284 | 61.556 | 44.25 | 54.2277 | 62.232 | 70.74 | 77.17 |


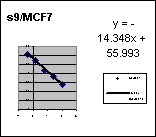

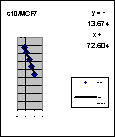
8/MCF7 9/MCF7

| 2 | 27.4 |
| --- | --- |
| 1.4 | 36.19 |
| 0.8 | 43.46 |
| 0.19 | 54.28 |
| -0.41 | 61.56 |

| 2 | 44.2503 |
| --- | --- |
| 1.3979 | 54.2277 |
| 0.7959 | 62.2322 |
| 0.1931 | 70.7441 |
| -  0.4089 | 77.1702 |

IC50=

IC50=

|  | **Blank** | **CC** | **Sample No. 10/MCF7** | | | | | **Sample No. 11/MCF7** | | | | |
| --- | --- | --- | --- | --- | --- | --- | --- | --- | --- | --- | --- | --- |
|  | **1** | **2** | **3** | **4** | **5** | **6** | **7** | **8** | **9** | **10** | **11** | **12** |
| A | B | C | 100ug | 25ug | 6.3ug | 1.6ug | 0.4ug | 100ug | 25ug | 6.3ug | 1.6ug | 0.4ug |
| B | B | C | 100ug | 25ug | 6.25ug | 1.6ug | 0.4ug | 100ug | 25ug | 6.25ug | 1.6ug | 0.4ug |
| C | B | C | 100ug | 25ug | 6.25ug | 1.6ug | 0.4ug | 100ug | 25ug | 6.25ug | 1.6ug | 0.4ug |

ROBONIK P2000 Eia reader Wave

length: 450 nm

Reference: 630 nm

|  | **1** | **2** | **3** | **4** | **5** | **6** | **7** | **8** | **9** | **10** | **11** | **12** |
| --- | --- | --- | --- | --- | --- | --- | --- | --- | --- | --- | --- | --- |

| A | 0.001 | 0.488 | 0.225 | 0.264 | 0.313 | 0.359 | 0.417 | 0.222 | 0.275 | 0.324 | 0.369 | 0.419 |
| --- | --- | --- | --- | --- | --- | --- | --- | --- | --- | --- | --- | --- |
| B | 0.001 | 0.469 | 0.236 | 0.258 | 0.326 | 0.364 | 0.408 | 0.219 | 0.283 | 0.309 | 0.367 | 0.433 |
| C | 0.001 | 0.503 | 0.194 | 0.271 | 0.303 | 0.347 | 0.425 | 0.228 | 0.275 | 0.318 | 0.363 | 0.404 |
| mean | 0.001 | 0.487 | 0.21833 | 0.2643 | 0.314 | 0.3567 | 0.4167 | 0.223 | 0.27767 | 0.317 | 0.366 | 0.419 |
| %  viability |  |  | 44.863 | 54.315 | 64.521 | 73.288 | 85.616 | 45.822 | 57.0548 | 65.137 | 75.27 | 86.03 |


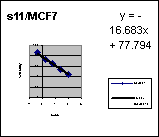

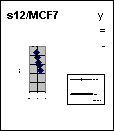
10/MCF7 11/MCF7

| 2 | 44.86 |
| --- | --- |
| 1.4 | 54.32 |
| 0.8 | 64.52 |
| 0.19 | 73.29 |
| -0.41 | 85.62 |

| 2 | 45.8219 |
| --- | --- |
| 1.3979 | 57.0548 |
| 0.7959 | 65.137 |
| 0.1931 | 75.274 |
| -  0.4089 | 86.0274 |

IC50=

IC50=

|  | **Blank** | **CC** | **Sample No. 12/MCF7** | | | | | **Sample No. 13/MCF7** | | | | |
| --- | --- | --- | --- | --- | --- | --- | --- | --- | --- | --- | --- | --- |
|  | **1** | **2** | **3** | **4** | **5** | **6** | **7** | **8** | **9** | **10** | **11** | **12** |
| A | B | C | 100ug | 25ug | 6.3ug | 1.6ug | 0.4ug | 100ug | 25ug | 6.3ug | 1.6ug | 0.4ug |
| B | B | C | 100ug | 25ug | 6.25ug | 1.6ug | 0.4ug | 100ug | 25ug | 6.25ug | 1.6ug | 0.4ug |
| C | B | C | 100ug | 25ug | 6.25ug | 1.6ug | 0.4ug | 100ug | 25ug | 6.25ug | 1.6ug | 0.4ug |

ROBONIK P2000 Eia reader Wave

length: 450 nm

Reference: 630 nm

|  | **1** | **2** | **3** | **4** | **5** | **6** | **7** | **8** | **9** | **10** | **11** | **12** |
| --- | --- | --- | --- | --- | --- | --- | --- | --- | --- | --- | --- | --- |

| A | 0.001 | 0.565 | 0.181 | 0.255 | 0.288 | 0.324 | 0.371 | 0.239 | 0.316 | 0.373 | 0.405 | 0.461 |
| --- | --- | --- | --- | --- | --- | --- | --- | --- | --- | --- | --- | --- |
| B | 0.002 | 0.581 | 0.179 | 0.257 | 0.285 | 0.326 | 0.372 | 0.225 | 0.311 | 0.364 | 0.404 | 0.448 |
| C | 0.001 | 0.559 | 0.203 | 0.246 | 0.282 | 0.331 | 0.369 | 0.233 | 0.312 | 0.359 | 0.401 | 0.469 |
| mean | 0.001 | 0.568 | 0.18767 | 0.2527 | 0.285 | 0.327 | 0.3707 | 0.2323 | 0.313 | 0.3653 | 0.403 | 0.459 |
| %  viability |  |  | 33.0205 | 44.457 | 50.147 | 57.537 | 65.22 | 40.88 | 55.0733 | 64.282 | 70.97 | 80.82 |


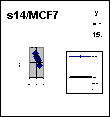
12/MCF7 13/MCF7


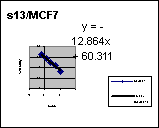


IC50=

| 2 | 33.02 |
| --- | --- |
| 1.4 | 44.46 |
| 0.8 | 50.15 |
| 0.19 | 57.54 |
| -0.41 | 65.22 |

| 2 | 40.8798 |
| --- | --- |
| 1.3979 | 55.0733 |
| 0.7959 | 64.2815 |
| 0.1931 | 70.9677 |
| -  0.4089 | 80.8211 |

IC50=

|  | **Blank** | **CC** | **Sample No. 14/MCF7** | | | | | **Sample No. 15/MCF7** | | | | |
| --- | --- | --- | --- | --- | --- | --- | --- | --- | --- | --- | --- | --- |
|  | **1** | **2** | **3** | **4** | **5** | **6** | **7** | **8** | **9** | **10** | **11** | **12** |
| A | B | C | 100ug | 25ug | 6.3ug | 1.6ug | 0.4ug | 100ug | 25ug | 6.3ug | 1.6ug | 0.4ug |
| B | B | C | 100ug | 25ug | 6.25ug | 1.6ug | 0.4ug | 100ug | 25ug | 6.25ug | 1.6ug | 0.4ug |
| C | B | C | 100ug | 25ug | 6.25ug | 1.6ug | 0.4ug | 100ug | 25ug | 6.25ug | 1.6ug | 0.4ug |

ROBONIK P2000 Eia reader Wave

length: 450 nm

Reference: 630 nm

|  | **1** | **2** | **3** | **4** | **5** | **6** | **7** | **8** | **9** | **10** | **11** | **12** |
| --- | --- | --- | --- | --- | --- | --- | --- | --- | --- | --- | --- | --- |

| A | 0.003 | 0.533 | 0.264 | 0.319 | 0.357 | 0.409 | 0.466 | 0.163 | 0.224 | 0.264 | 0.313 | 0.357 |
| --- | --- | --- | --- | --- | --- | --- | --- | --- | --- | --- | --- | --- |
| B | 0.001 | 0.526 | 0.272 | 0.322 | 0.352 | 0.412 | 0.462 | 0.169 | 0.221 | 0.266 | 0.303 | 0.362 |
| C | 0.001 | 0.527 | 0.269 | 0.316 | 0.353 | 0.404 | 0.469 | 0.165 | 0.231 | 0.264 | 0.308 | 0.359 |
| mean | 0.002 | 0.529 | 0.26833 | 0.319 | 0.354 | 0.4083 | 0.4657 | 0.1657 | 0.22533 | 0.2647 | 0.308 | 0.359 |
| %  viability |  |  | 50.7566 | 60.34 | 66.961 | 77.238 | 88.083 | 31.337 | 42.623 | 50.063 | 58.26 | 67.97 |


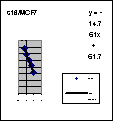
14/MCF7 15/MCF7


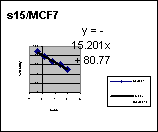


IC50=

| 2 | 50.76 |
| --- | --- |
| 1.4 | 60.34 |
| 0.8 | 66.96 |
| 0.19 | 77.24 |
| -0.41 | 88.08 |

| 2 | 31.3367 |
| --- | --- |
| 1.3979 | 42.623 |
| 0.7959 | 50.0631 |
| 0.1931 | 58.2598 |
| -  0.4089 | 67.9697 |

IC50=

|  | **Blank** | **CC** | **Sample No. Dox/MCF7** | | | | |
| --- | --- | --- | --- | --- | --- | --- | --- |
|  | **1** | **2** | **3** | **4** | **5** | **6** | **7** |
| A | B | C | 100ug | 25ug | 6.3ug | 1.6ug | 0.4ug |
| B | B | C | 100ug | 25ug | 6.25ug | 1.6ug | 0.4ug |
| C | B | C | 100ug | 25ug | 6.25ug | 1.6ug | 0.4ug |

ROBONIK P2000 Eia reader Wave

length: 450 nm

Reference: 630 nm

|  | **1** | **2** | **3** | **4** | **5** | **6** | **7** |
| --- | --- | --- | --- | --- | --- | --- | --- |

| A | 0.001 | 0.552 | 0.155 | 0.211 | 0.249 | 0.288 | 0.344 |
| --- | --- | --- | --- | --- | --- | --- | --- |
| B | 0.002 | 0.543 | 0.159 | 0.206 | 0.261 | 0.285 | 0.343 |
| C | 0.001 | 0.546 | 0.161 | 0.211 | 0.252 | 0.283 | 0.346 |
| mean | 0.001 | 0.547 | 0.1583 | 0.20933 | 0.254 | 0.285 | 0.344 |
| %  viability |  |  | 28.946 | 38.2693 | 46.435 | 52.16 | 62.95 |


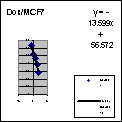
Dox/MCF7

| 2 | 28.9458 |
| --- | --- |
| 1.3979 | 38.2693 |
| 0.7959 | 46.4351 |

| 0.1931 | 52.1633 |
| --- | --- |
| -  0.4089 | 62.9494 |

IC50=

|  | **Blank** | **CC** | **Sample No. 1/MDA** | | | | |
| --- | --- | --- | --- | --- | --- | --- | --- |
|  | **1** | **2** | **3** | **4** | **5** | **6** | **7** |
| A | B | C | 100ug | 25ug | 6.3ug | 1.6ug | 0.4ug |
| B | B | C | 100ug | 25ug | 6.25ug | 1.6ug | 0.4ug |
| C | B | C | 100ug | 25ug | 6.25ug | 1.6ug | 0.4ug |

ROBONIK P2000 Eia reader Wave

length: 450 nm

Reference: 630 nm

|  | **1** | **2** | **3** | **4** | **5** | **6** | **7** |
| --- | --- | --- | --- | --- | --- | --- | --- |

| A | 0.003 | 0.505 | 0.236 | 0.287 | 0.341 | 0.388 | 0.436 |
| --- | --- | --- | --- | --- | --- | --- | --- |
| B | 0.001 | 0.477 | 0.233 | 0.272 | 0.334 | 0.373 | 0.431 |
| C | 0.001 | 0.483 | 0.241 | 0.271 | 0.336 | 0.369 | 0.428 |
| mean | 0.002 | 0.488 | 0.23667 | 0.2767 | 0.337 | 0.3767 | 0.4317 |
| %  viability |  |  | 48.4642 | 56.655 | 69.01 | 77.133 | 88.396 |

1/MDA


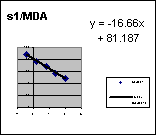


| 2 | 48.46 |
| --- | --- |
| 1.4 | 56.66 |
| 0.8 | 69.01 |
| 0.19 | 77.13 |
| -0.41 | 88.4 |

IC50=

|  | **Blank** | **CC** | **Sample No. 2/MDA** | | | | | **Sample No. 3/MDA** | | | | |
| --- | --- | --- | --- | --- | --- | --- | --- | --- | --- | --- | --- | --- |
|  | **1** | **2** | **3** | **4** | **5** | **6** | **7** | **8** | **9** | **10** | **11** | **12** |
| A | B | C | 100ug | 25ug | 6.3ug | 1.6ug | 0.4ug | 100ug | 25ug | 6.3ug | 1.6ug | 0.4ug |
| B | B | C | 100ug | 25ug | 6.25ug | 1.6ug | 0.4ug | 100ug | 25ug | 6.25ug | 1.6ug | 0.4ug |
| C | B | C | 100ug | 25ug | 6.25ug | 1.6ug | 0.4ug | 100ug | 25ug | 6.25ug | 1.6ug | 0.4ug |

ROBONIK P2000 Eia reader Wave

length: 450 nm

Reference: 630 nm

|  | **1** | **2** | **3** | **4** | **5** | **6** | **7** | **8** | **9** | **10** | **11** | **12** |
| --- | --- | --- | --- | --- | --- | --- | --- | --- | --- | --- | --- | --- |
|  |  | | | | | | | | | | | |
| A | 0.001 | 0.554 | 0.198 | 0.262 | 0.306 | 0.354 | 0.404 | 0.221 | 0.267 | 0.313 | 0.354 | 0.414 |
| B | 0.003 | 0.549 | 0.212 | 0.255 | 0.328 | 0.362 | 0.418 | 0.241 | 0.281 | 0.329 | 0.362 | 0.419 |

| C | 0.001 | 0.555 | 0.196 | 0.271 | 0.317 | 0.371 | 0.436 | 0.208 | 0.269 | 0.331 | 0.378 | 0.431 |
| --- | --- | --- | --- | --- | --- | --- | --- | --- | --- | --- | --- | --- |
| mean | 0.002 | 0.553 | 0.202 | 0.2627 | 0.317 | 0.3623 | 0.4193 | 0.2233 | 0.27233 | 0.3243 | 0.365 | 0.421 |
| %  2/MDA |  | ` | 36.5501 | 47.527 | 57.358 | 65.561 | 75.875 | 40.41 | 49.2762 | 58.685 | 65.98 | 76.24 |
|  |  | 3/MDA | | | | | | | | | | |
| log conc. | %  viability |  |  |  |  |  |  |  |  |  |  |  |
| 2 | 36.55 |  |  |  |  |  |  |  |  |  |  |  |
| 1.4 | 47.53 |  |  |  |  |  |  |  |  |  |  |  |
| 0.8 | 57.36 |  |  |  |  |  |  |  |  |  |  |  |
| 0.19 | 65.56 |  |  |  |  |  |  |  |  |  |  |  |
| -0.41 | 75.87 |  |  |  |  |  |  |  |  |  |  |  |


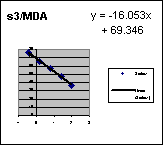

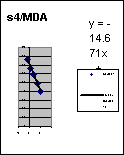


IC50=

IC50=

| log conc. | %  viability |
| --- | --- |
| 2 | 40.4101 |
| 1.3979 | 49.2762 |
| 0.7959 | 58.6852 |
| 0.1931 | 65.9831 |
| -0.4089 | 76.2364 |

|  | **Blank** | **CC** | **Sample No. 4/MDA** | | | | | **Sample No. 6/MDA** | | | | |
| --- | --- | --- | --- | --- | --- | --- | --- | --- | --- | --- | --- | --- |
|  | **1** | **2** | **3** | **4** | **5** | **6** | **7** | **8** | **9** | **10** | **11** | **12** |
| A | B | C | 100ug | 25ug | 6.3ug | 1.6ug | 0.4ug | 100ug | 25ug | 6.3ug | 1.6ug | 0.4ug |
| B | B | C | 100ug | 25ug | 6.25ug | 1.6ug | 0.4ug | 100ug | 25ug | 6.25ug | 1.6ug | 0.4ug |
| C | B | C | 100ug | 25ug | 6.25ug | 1.6ug | 0.4ug | 100ug | 25ug | 6.25ug | 1.6ug | 0.4ug |

ROBONIK P2000 Eia reader Wave

length: 450 nm

Reference: 630 nm

|  | **1** | **2** | **3** | **4** | **5** | **6** | **7** | **8** | **9** | **10** | **11** | **12** |
| --- | --- | --- | --- | --- | --- | --- | --- | --- | --- | --- | --- | --- |

| A | 0.001 | 0.565 | 0.133 | 0.191 | 0.242 | 0.291 | 0.345 | 0.232 | 0.274 | 0.313 | 0.356 | 0.418 |
| --- | --- | --- | --- | --- | --- | --- | --- | --- | --- | --- | --- | --- |
| B | 0.001 | 0.559 | 0.147 | 0.195 | 0.243 | 0.292 | 0.344 | 0.235 | 0.279 | 0.319 | 0.358 | 0.424 |
| C | 0.001 | 0.566 | 0.146 | 0.191 | 0.246 | 0.288 | 0.338 | 0.228 | 0.282 | 0.323 | 0.352 | 0.403 |
| mean | 0.001 | 0.563 | 0.142 | 0.1923 | 0.2437 | 0.2903 | 0.3423 | 0.2317 | 0.27833 | 0.3183 | 0.355 | 0.415 |
| %  viability |  |  | 25.2071 | 34.142 | 43.254 | 51.538 | 60.769 | 41.124 | 49.4083 | 56.509 | 63.08 | 73.67 |


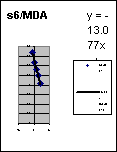
4/MDA 6/MDA


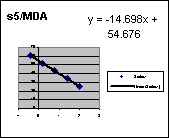


| log conc. | %  viability |
| --- | --- |
| 2 | 25.21 |
| 1.4 | 34.14 |
| 0.8 | 43.25 |
| 0.19 | 51.54 |
| -0.41 | 60.77 |

| log conc. | %  viability |
| --- | --- |
| 2 | 41.1243 |
| 1.3979 | 49.4083 |
| 0.7959 | 56.5089 |
| 0.1931 | 63.0769 |
| -0.4089 | 73.6686 |

IC50=

IC50=

|  | **Blank** | **CC** | **Sample No. 5/MDA** | | | | | **Sample No. 7/MDA** | | | | |
| --- | --- | --- | --- | --- | --- | --- | --- | --- | --- | --- | --- | --- |
|  | **1** | **2** | **3** | **4** | **5** | **6** | **7** | **8** | **9** | **10** | **11** | **12** |

| A | B | C | 100ug | 25ug | 6.3ug | 1.6ug | 0.4ug | 100ug | 25ug | 6.3ug | 1.6ug | 0.4ug |
| --- | --- | --- | --- | --- | --- | --- | --- | --- | --- | --- | --- | --- |
| B | B | C | 100ug | 25ug | 6.25ug | 1.6ug | 0.4ug | 100ug | 25ug | 6.25ug | 1.6ug | 0.4ug |
| C | B | C | 100ug | 25ug | 6.25ug | 1.6ug | 0.4ug | 100ug | 25ug | 6.25ug | 1.6ug | 0.4ug |


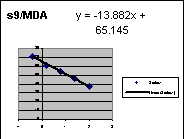

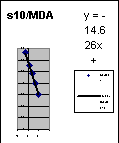
ROBONIK P2000 Eia reader Wave

length: 450 nm

Reference: 630 nm


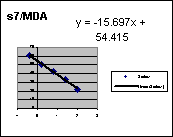


5/MDA

IC50=


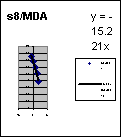


7/MDA

IC50=

|  | **1** | **2** | **3** | **4** | **5** | **6** | **7** | **8** | **9** | **10** | **11** | **12** |
| --- | --- | --- | --- | --- | --- | --- | --- | --- | --- | --- | --- | --- |

| A | 0.001 | 0.532 | 0.124 | 0.181 | 0.226 | 0.264 | 0.319 | 0.201 | 0.251 | 0.306 | 0.355 | 0.388 |
| --- | --- | --- | --- | --- | --- | --- | --- | --- | --- | --- | --- | --- |
| B | 0.001 | 0.529 | 0.119 | 0.177 | 0.221 | 0.271 | 0.319 | 0.209 | 0.258 | 0.314 | 0.367 | 0.404 |
| C | 0.001 | 0.524 | 0.111 | 0.175 | 0.219 | 0.265 | 0.3317 | 0.208 | 0.263 | 0.319 | 0.366 | 0.412 |
| mean | 0.001 | 0.528 | 0.118 | 0.1777 | 0.222 | 0.2667 | 0.3232 | 0.206 | 0.269 | 0.313 | 0.363 | 0.401 |
| %  viability |  |  | 22.3344 | 33.628 | 42.019 | 50.473 | 61.18 | 38.991 | 50.9148 | 59.243 | 68.64 | 75.96 |

| 2 | 22.33 |
| --- | --- |
| 1.4 | 33.63 |
| 0.8 | 42.02 |
| 0.19 | 50.47 |
| -0.41 | 61.18 |

| 2 | 38.9905 |
| --- | --- |
| 1.3979 | 50.9148 |
| 0.7959 | 59.2429 |
| 0.1931 | 68.6435 |
| -0.4089 | 75.9621 |

|  | **Blank** | **CC** | **Sample No. 8/MDA** | | | | | **Sample No. 9/MDA** | | | | |
| --- | --- | --- | --- | --- | --- | --- | --- | --- | --- | --- | --- | --- |
|  | **1** | **2** | **3** | **4** | **5** | **6** | **7** | **8** | **9** | **10** | **11** | **12** |
| A | B | C | 100ug | 25ug | 6.3ug | 1.6ug | 0.4ug | 100ug | 25ug | 6.3ug | 1.6ug | 0.4ug |
| B | B | C | 100ug | 25ug | 6.25ug | 1.6ug | 0.4ug | 100ug | 25ug | 6.25ug | 1.6ug | 0.4ug |
| C | B | C | 100ug | 25ug | 6.25ug | 1.6ug | 0.4ug | 100ug | 25ug | 6.25ug | 1.6ug | 0.4ug |

ROBONIK P2000 Eia reader Wave

length: 450 nm

Reference: 630 nm

|  | **1** | **2** | **3** | **4** | **5** | **6** | **7** | **8** | **9** | **10** | **11** | **12** |
| --- | --- | --- | --- | --- | --- | --- | --- | --- | --- | --- | --- | --- |

A B

| 0.001 | 0.606 | 0.224 | 0.272 | 0.323 | 0.361 | 0.411 | 0.175 | 0.231 | 0.282 | 0.331 | 0.393 |
| --- | --- | --- | --- | --- | --- | --- | --- | --- | --- | --- | --- |
| 0.001 | 0.572 | 0.218 | 0.269 | 0.321 | 0.362 | 0.424 | 0.177 | 0.235 | 0.285 | 0.324 | 0.395 |
| 0.001 | 0.585 | 0.216 0.267 | | 0.319 | 0.359 | 0.423 | 0.181 | 0.236 | 0.322  0.291 | | 0.384 |
| 0.001 | 0.588 | 0.21933 | 0.2693 | 0.321 | 0.3607 | 0.4193 | 0.1777 | 0.234 | 0.286 | 0.326 | 0.391 |
|  | ` | 37.3227 | 45.831 | 54.623 | 61.373 | 71.356 | 30.233 | 39.8185 | 48.667 | 55.42 | 66.48 |

C

mean

%

8/MDA 9/MDA

| log  conc. | %  viability |  |
| --- | --- | --- |
| 2 | 37.32 |  |
| 1.4 | 45.83 |  |
| 0.8 | 54.62 |  |
| 0.19 | 61.37 |  |
| -0.41 | 71.36 |  |

| log  conc. | %  viability |
| --- | --- |
| 2 | 30.2326 |
| 1.3979 | 39.8185 |
| 0.7959 | 48.667 |
| 0.1931 | 55.4169 |
| -0.4089 | 66.4776 |

IC50=

IC50=

|  | **Blank** | **CC** | **Sample No. 10/MDA** | | | | | **Sample No. 11/MDA** | | | | |
| --- | --- | --- | --- | --- | --- | --- | --- | --- | --- | --- | --- | --- |
|  | **1** | **2** | **3** | **4** | **5** | **6** | **7** | **8** | **9** | **10** | **11** | **12** |
| A | B | C | 100ug | 25ug | 6.3ug | 1.6ug | 0.4ug | 100ug | 25ug | 6.3ug | 1.6ug | 0.4ug |
| B | B | C | 100ug | 25ug | 6.25ug | 1.6ug | 0.4ug | 100ug | 25ug | 6.25ug | 1.6ug | 0.4ug |
| C | B | C | 100ug | 25ug | 6.25ug | 1.6ug | 0.4ug | 100ug | 25ug | 6.25ug | 1.6ug | 0.4ug |

ROBONIK P2000 Eia reader Wave

length: 450 nm

Reference: 630 nm

|  | **1** | **2** | **3** | **4** | **5** | **6** | **7** | **8** | **9** | **10** | **11** | **12** |
| --- | --- | --- | --- | --- | --- | --- | --- | --- | --- | --- | --- | --- |

| A | 0.001 | 0.572 | 0.128 | 0.171 | 0.223 | 0.265 | 0.313 | 0.231 | 0.289 | 0.324 | 0.375 | 0.415 |
| --- | --- | --- | --- | --- | --- | --- | --- | --- | --- | --- | --- | --- |
| B | 0.001 | 0.549 | 0.137 | 0.174 | 0.227 | 0.268 | 0.317 | 0.235 | 0.295 | 0.326 | 0.376 | 0.414 |
| C | 0.001 | 0.555 | 0.129 | 0.168 | 0.225 | 0.261 | 0.315 | 0.226 | 0.292 | 0.323 | 0.367 | 0.406 |
| mean | 0.001 | 0.559 | 0.13133 | 0.171 | 0.225 | 0.2647 | 0.315 | 0.2307 | 0.292 | 0.3243 | 0.373 | 0.412 |
| %  viability |  |  | 23.5084 | 30.609 | 40.274 | 47.375 | 56.384 | 41.289 | 52.2673 | 58.055 | 66.71 | 73.69 |


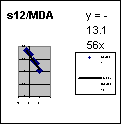
10/MDA 11/MDA


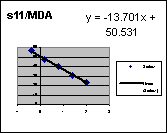


| log conc. | %  viability |
| --- | --- |
| 2 | 23.51 |
| 1.4 | 30.61 |
| 0.8 | 40.27 |
| 0.19 | 47.37 |
| -0.41 | 56.38 |
| IC50= | |

| log conc. | %  viability |
| --- | --- |
| 2 | 41.2888 |
| 1.3979 | 52.2673 |
| 0.7959 | 58.0549 |
| 0.1931 | 66.7064 |
| -0.4089 | 73.6874 |
| IC50= | |

|  | **Blank** | **CC** | **Sample No. 12/MDA** | | | | | **Sample No. 13/MDA** | | | | |
| --- | --- | --- | --- | --- | --- | --- | --- | --- | --- | --- | --- | --- |
|  | **1** | **2** | **3** | **4** | **5** | **6** | **7** | **8** | **9** | **10** | **11** | **12** |
| A | B | C | 100ug | 25ug | 6.3ug | 1.6ug | 0.4ug | 100ug | 25ug | 6.3ug | 1.6ug | 0.4ug |
| B | B | C | 100ug | 25ug | 6.25ug | 1.6ug | 0.4ug | 100ug | 25ug | 6.25ug | 1.6ug | 0.4ug |
| C | B | C | 100ug | 25ug | 6.25ug | 1.6ug | 0.4ug | 100ug | 25ug | 6.25ug | 1.6ug | 0.4ug |

ROBONIK P2000 Eia reader Wave

length: 450 nm

Reference: 630 nm


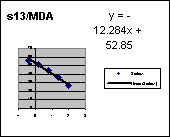


12/MDA

IC50=


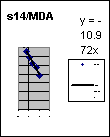


13/MDA

IC50=

|  | **1** | **2** | **3** | **4** | **5** | **6** | **7** | **8** | **9** | **10** | **11** | **12** |
| --- | --- | --- | --- | --- | --- | --- | --- | --- | --- | --- | --- | --- |

| A | 0.001 | 0.611 | 0.171 | 0.225 | 0.278 | 0.313 | 0.352 | 0.244 | 0.289 | 0.321 | 0.365 | 0.409 |
| --- | --- | --- | --- | --- | --- | --- | --- | --- | --- | --- | --- | --- |
| B | 0.003 | 0.642 | 0.169 | 0.231 | 0.274 | 0.324 | 0.353 | 0.241 | 0.296 | 0.325 | 0.362 | 0.414 |
| C | 0.001 | 0.619 | 0.163 | 0.228 | 0.276 | 0.322 | 0.353 | 0.236 | 0.294 | 0.333 | 0.369 | 0.408 |
| mean | 0.002 | 0.624 | 0.16767 | 0.228 | 0.276 | 0.3197 | 0.3527 | 0.2403 | 0.293 | 0.3263 | 0.365 | 0.41 |
| %  viability |  |  | 26.8697 | 36.538 | 44.231 | 51.229 | 56.517 | 38.515 | 46.9551 | 52.297 | 58.55 | 65.76 |

| 2 | 26.87 |
| --- | --- |
| 1.4 | 36.54 |
| 0.8 | 44.23 |
| 0.19 | 51.23 |
| -0.41 | 56.52 |

| 2 | 38.515 |
| --- | --- |
| 1.3979 | 46.9551 |
| 0.7959 | 52.297 |
| 0.1931 | 58.547 |
| -0.4089 | 65.7585 |

|  | **Blank** | **CC** | **Sample No. 14/MDA** | | | | | **Sample No. 15/MDA** | | | | |
| --- | --- | --- | --- | --- | --- | --- | --- | --- | --- | --- | --- | --- |
|  | **1** | **2** | **3** | **4** | **5** | **6** | **7** | **8** | **9** | **10** | **11** | **12** |
| A | B | C | 100ug | 25ug | 6.3ug | 1.6ug | 0.4ug | 100ug | 25ug | 6.3ug | 1.6ug | 0.4ug |
| B | B | C | 100ug | 25ug | 6.25ug | 1.6ug | 0.4ug | 100ug | 25ug | 6.25ug | 1.6ug | 0.4ug |
| C | B | C | 100ug | 25ug | 6.25ug | 1.6ug | 0.4ug | 100ug | 25ug | 6.25ug | 1.6ug | 0.4ug |

ROBONIK P2000 Eia reader Wave

length: 450 nm

Reference: 630 nm

|  | **1** | **2** | **3** | **4** | **5** | **6** | **7** | **8** | **9** | **10** | **11** | **12** |
| --- | --- | --- | --- | --- | --- | --- | --- | --- | --- | --- | --- | --- |

| A | 0.001 | 0.581 | 0.077 | 0.154 | 0.198 | 0.254 | 0.308 | 0.227 | 0.282 | 0.342 | 0.391 | 0.448 |
| --- | --- | --- | --- | --- | --- | --- | --- | --- | --- | --- | --- | --- |
| B | 0.001 | 0.603 | 0.107 | 0.163 | 0.205 | 0.262 | 0.317 | 0.221 | 0.275 | 0.339 | 0.389 | 0.463 |
| C | 0.001 | 0.599 | 0.092 | 0.149 | 0.217 | 0.257 | 0.329 | 0.225 | 0.279 | 0.334 | 0.395 | 0.457 |
| mean | 0.001 | 0.594 | 0.092 | 0.1553 | 0.2067 | 0.2577 | 0.318 | 0.2243 | 0.27867 | 0.3383 | 0.392 | 0.456 |
| %  viability |  |  | 15.4795 | 26.136 | 34.773 | 43.354 | 53.505 | 37.745 | 46.8873 | 56.927 | 65.9 | 76.72 |


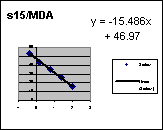

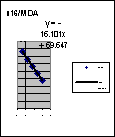
14/MDA 15/MDA

| 2 | 15.48 |
| --- | --- |
| 1.4 | 26.14 |
| 0.8 | 34.77 |
| 0.19 | 43.35 |
| -0.41 | 53.51 |

| 2 | 37.7454 |
| --- | --- |
| 1.3979 | 46.8873 |
| 0.7959 | 56.9265 |
| 0.1931 | 65.9002 |
| -0.4089 | 76.7246 |

IC50= IC50=

|  | **Blank** | **CC** | **Sample No. Dox/MDA** | | | | |
| --- | --- | --- | --- | --- | --- | --- | --- |
|  | **1** | **2** | **3** | **4** | **5** | **6** | **7** |
| A | B | C | 100ug | 25ug | 6.3ug | 1.6ug | 0.4ug |
| B | B | C | 100ug | 25ug | 6.25ug | 1.6ug | 0.4ug |
| C | MZ47 | C | 100ug | 25ug | 6.25ug | 1.6ug | 0.4ug |

ROBONIK P2000 Eia reader Wave

length: 450 nm

Reference: 630 nm

|  | **1** | **2** | **3** | **4** | **5** | **6** | **7** |
| --- | --- | --- | --- | --- | --- | --- | --- |

| A | 0.001 | 0.557 | 0.171 | 0.228 | 0.264 | 0.314 | 0.365 |
| --- | --- | --- | --- | --- | --- | --- | --- |
| B | 0.002 | 0.568 | 0.159 | 0.231 | 0.267 | 0.318 | 0.344 |
| C | 0.001 | 0.571 | 0.172 | 0.236 | 0.259 | 0.315 | 0.369 |
| mean | 0.001 | 0.565 | 0.1673 | 0.23167 | 0.2633 | 0.316 | 0.359 |
| %  viability |  |  | 29.599 | 40.9788 | 46.58 | 55.84 | 63.56 |


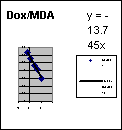
Dox/MDA

| 2 | 29.5991 |
| --- | --- |
| 1.3979 | 40.9788 |
| 0.7959 | 46.5802 |
| 0.1931 | 55.8373 |
| -0.4089 | 63.5613 |

IC50=

|  | **Blank** | **CC** | **Sample No. 1/MCF10a** | | | | |
| --- | --- | --- | --- | --- | --- | --- | --- |
|  | **1** | **2** | **3** | **4** | **5** | **6** | **7** |
| A | B | C | 100ug | 25ug | 6.3ug | 1.6ug | 0.4ug |
| B | B | C | 100ug | 25ug | 6.25ug | 1.6ug | 0.4ug |
| C | B | C | 100ug | 25ug | 6.25ug | 1.6ug | 0.4ug |

ROBONIK P2000 Eia reader Wave

length: 450 nm

Reference: 630 nm

|  | **1** | **2** | **3** | **4** | **5** | **6** | **7** |
| --- | --- | --- | --- | --- | --- | --- | --- |

| A | 0.002 | 0.575 | 0.275 | 0.324 | 0.382 | 0.424 | 0.467 |
| --- | --- | --- | --- | --- | --- | --- | --- |
| B | 0.001 | 0.562 | 0.289 | 0.323 | 0.384 | 0.431 | 0.478 |
| C | 0.001 | 0.558 | 0.285 | 0.319 | 0.378 | 0.426 | 0.479 |
| mean | 0.001 | 0.565 | 0.283 | 0.322 | 0.3813 | 0.427 | 0.4747 |
| %  viability |  |  | 50.0885 | 56.991 | 67.493 | 75.575 | 84.012 |

1/MCF10a


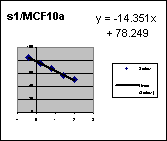


IC50=

| 2 | 50.09 |
| --- | --- |
| 1.4 | 56.99 |
| 0.8 | 67.49 |
| 0.19 | 75.58 |
| -0.41 | 84.01 |

|  | **Blank** | **CC** | **Sample No. 2/MCF10a** | | | | | **Sample No. 3/MCF10a** | | | | |
| --- | --- | --- | --- | --- | --- | --- | --- | --- | --- | --- | --- | --- |
|  | **1** | **2** | **3** | **4** | **5** | **6** | **7** | **8** | **9** | **10** | **11** | **12** |
| A | B | C | 100ug | 25ug | 6.3ug | 1.6ug | 0.4ug | 100ug | 25ug | 6.3ug | 1.6ug | 0.4ug |
| B | B | C | 100ug | 25ug | 6.25ug | 1.6ug | 0.4ug | 100ug | 25ug | 6.25ug | 1.6ug | 0.4ug |
| C | B | C | 100ug | 25ug | 6.25ug | 1.6ug | 0.4ug | 100ug | 25ug | 6.25ug | 1.6ug | 0.4ug |

ROBONIK P2000 Eia reader Wave

length: 450 nm

Reference: 630 nm

|  | **1** | **2** | **3** | **4** | **5** | **6** | **7** | **8** | **9** | **10** | **11** | **12** |
| --- | --- | --- | --- | --- | --- | --- | --- | --- | --- | --- | --- | --- |

| A | 0.001 | 0.485 | 0.221 | 0.288 | 0.331 | 0.377 | 0.424 | 0.257 | 0.303 | 0.354 | 0.41 | 0.466 |
| --- | --- | --- | --- | --- | --- | --- | --- | --- | --- | --- | --- | --- |
| B | 0.002 | 0.479 | 0.209 | 0.275 | 0.316 | 0.379 | 0.427 | 0.262 | 0.296 | 0.358 | 0.405 | 0.465 |
| C | 0.001 | 0.492 | 0.217 | 0.276 | 0.321 | 0.368 | 0.431 | 0.249 | 0.292 | 0.349 | 0.406 | 0.464 |

| mean | 0.001 | 0.485 | 0.21567 | 0.2797 | 0.3227 | 0.3747 | 0.4273 | 0.256 | 0.297 | 0.3537 | 0.407 | 0.465 |
| --- | --- | --- | --- | --- | --- | --- | --- | --- | --- | --- | --- | --- |
| %  viability |  |  | 44.4368 | 57.624 | 66.484 | 77.198 | 88.049 | 52.747 | 61.1951 | 72.871 | 83.86 | 95.81 |


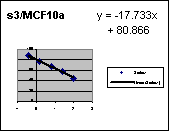

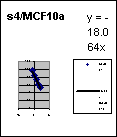
2/MCF10a 3/MCF10a

| 2 | 44.44 |
| --- | --- |
| 1.4 | 57.62 |
| 0.8 | 66.48 |
| 0.19 | 77.2 |
| -0.41 | 88.05 |

| 2 | 52.7473 |
| --- | --- |
| 1.3979 | 61.1951 |
| 0.7959 | 72.8709 |
| 0.1931 | 83.8599 |
| -  0.4089 | 95.8104 |

IC50=

IC50=

|  | **Blank** | **CC** | **Sample No. 4/MCF10a** | | | | | **Sample No. 6/MCF10a** | | | | |
| --- | --- | --- | --- | --- | --- | --- | --- | --- | --- | --- | --- | --- |
|  | **1** | **2** | **3** | **4** | **5** | **6** | **7** | **8** | **9** | **10** | **11** | **12** |
| A | B | C | 100ug | 25ug | 6.3ug | 1.6ug | 0.4ug | 100ug | 25ug | 6.3ug | 1.6ug | 0.4ug |
| B | B | C | 100ug | 25ug | 6.25ug | 1.6ug | 0.4ug | 100ug | 25ug | 6.25ug | 1.6ug | 0.4ug |
| C | B | C | 100ug | 25ug | 6.25ug | 1.6ug | 0.4ug | 100ug | 25ug | 6.25ug | 1.6ug | 0.4ug |

ROBONIK P2000 Eia reader Wave

length: 450 nm

Reference: 630 nm

|  | **1** | **2** | **3** | **4** | **5** | **6** | **7** | **8** | **9** | **10** | **11** | **12** |
| --- | --- | --- | --- | --- | --- | --- | --- | --- | --- | --- | --- | --- |

| A | 0.001 | 0.609 | 0.276 | 0.324 | 0.374 | 0.424 | 0.467 | 0.289 | 0.334 | 0.385 | 0.434 | 0.474 |
| --- | --- | --- | --- | --- | --- | --- | --- | --- | --- | --- | --- | --- |
| B | 0.001 | 0.614 | 0.271 | 0.326 | 0.371 | 0.425 | 0.469 | 0.285 | 0.336 | 0.378 | 0.428 | 0.481 |
| C | 0.001 | 0.608 | 0.265 | 0.331 | 0.373 | 0.423 | 0.471 | 0.282 | 0.333 | 0.376 | 0.427 | 0.475 |
| mean | 0.001 | 0.61 | 0.27067 | 0.327 | 0.3727 | 0.424 | 0.469 | 0.2853 | 0.33433 | 0.3797 | 0.43 | 0.477 |
| %  viability |  |  | 44.3474 | 53.577 | 61.06 | 69.47 | 76.843 | 46.75 | 54.7788 | 62.206 | 70.4 | 78.1 |


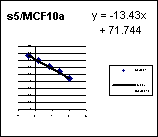

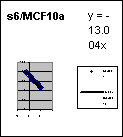
4/MCF10a 6/MCF10a

| 2 | 44.35 |
| --- | --- |
| 1.4 | 53.58 |
| 0.8 | 61.06 |
| 0.19 | 69.47 |
| -0.41 | 76.84 |

| 2 | 46.7504 |
| --- | --- |
| 1.3979 | 54.7788 |
| 0.7959 | 62.2064 |
| 0.1931 | 70.3987 |
| -  0.4089 | 78.0994 |

IC50=

IC50=

|  | **Blank** | **CC** | **Sample No. 5/MCF10a** | | | | | **Sample No. 7/MCF10a** | | | | |
| --- | --- | --- | --- | --- | --- | --- | --- | --- | --- | --- | --- | --- |
|  | **1** | **2** | **3** | **4** | **5** | **6** | **7** | **8** | **9** | **10** | **11** | **12** |
| A | B | C | 100ug | 25ug | 6.3ug | 1.6ug | 0.4ug | 100ug | 25ug | 6.3ug | 1.6ug | 0.4ug |

| B | B | C | 100ug | 25ug | 6.25ug | 1.6ug | 0.4ug | 100ug | 25ug | 6.25ug | 1.6ug | 0.4ug |
| --- | --- | --- | --- | --- | --- | --- | --- | --- | --- | --- | --- | --- |
| C | B | C | 100ug | 25ug | 6.25ug | 1.6ug | 0.4ug | 100ug | 25ug | 6.25ug | 1.6ug | 0.4ug |


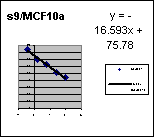

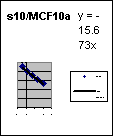
ROBONIK P2000 Eia reader Wave

length: 450 nm

Reference: 630 nm

|  | **1** | **2** | **3** | **4** | **5** | **6** | **7** | **8** | **9** | **10** | **11** | **12** |
| --- | --- | --- | --- | --- | --- | --- | --- | --- | --- | --- | --- | --- |

| A | 0.001 | 0.592 | 0.252 | 0.289 | 0.334 | 0.388 | 0.431 | 0.266 | 0.308 | 0.351 | 0.418 | 0.464 |
| --- | --- | --- | --- | --- | --- | --- | --- | --- | --- | --- | --- | --- |
| B | 0.001 | 0.583 | 0.257 | 0.294 | 0.341 | 0.401 | 0.441 | 0.267 | 0.321 | 0.347 | 0.419 | 0.456 |
| C | 0.001 | 0.585 | 0.256 | 0.296 | 0.339 | 0.385 | 0.438 | 0.261 | 0.316 | 0.344 | 0.422 | 0.459 |
| mean | 0.001 | 0.587 | 0.255 | 0.293 | 0.338 | 0.3913 | 0.4367 | 0.2647 | 0.315 | 0.3473 | 0.42 | 0.46 |
| %  viability |  |  | 43.4659 | 49.943 | 57.614 | 66.705 | 74.432 | 45.114 | 53.6932 | 59.205 | 71.53 | 78.35 |


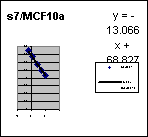
5/MCF10a 7MCF10a


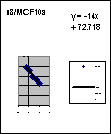


IC50=

| 2 | 43.47 |
| --- | --- |
| 1.4 | 49.94 |
| 0.8 | 57.61 |
| 0.19 | 66.7 |
| -0.41 | 74.43 |

| 2 | 45.1136 |
| --- | --- |
| 1.3979 | 53.6932 |
| 0.7959 | 59.2045 |
| 0.1931 | 71.5341 |
| -  0.4089 | 78.3523 |

IC50=

|  | **Blank** | **CC** | **Sample No. 8/MCF10a** | | | | | **Sample No. 9/MCF10a** | | | | |
| --- | --- | --- | --- | --- | --- | --- | --- | --- | --- | --- | --- | --- |
|  | **1** | **2** | **3** | **4** | **5** | **6** | **7** | **8** | **9** | **10** | **11** | **12** |
| A | B | C | 100ug | 25ug | 6.3ug | 1.6ug | 0.4ug | 100ug | 25ug | 6.3ug | 1.6ug | 0.4ug |
| B | B | C | 100ug | 25ug | 6.25ug | 1.6ug | 0.4ug | 100ug | 25ug | 6.25ug | 1.6ug | 0.4ug |
| C | B | C | 100ug | 25ug | 6.25ug | 1.6ug | 0.4ug | 100ug | 25ug | 6.25ug | 1.6ug | 0.4ug |

ROBONIK P2000 Eia reader Wave

length: 450 nm

Reference: 630 nm

|  | **1** | **2** | **3** | **4** | **5** | **6** | **7** | **8** | **9** | **10** | **11** | **12** |
| --- | --- | --- | --- | --- | --- | --- | --- | --- | --- | --- | --- | --- |

| A | 0.001 | 0.525 | 0.228 | 0.272 | 0.323 | 0.366 | 0.441 | 0.276 | 0.323 | 0.364 | 0.408 | 0.482 |
| --- | --- | --- | --- | --- | --- | --- | --- | --- | --- | --- | --- | --- |
| B | 0.002 | 0.507 | 0.225 | 0.267 | 0.331 | 0.359 | 0.428 | 0.263 | 0.316 | 0.362 | 0.414 | 0.459 |
| C | 0.001 | 0.519 | 0.223 | 0.265 | 0.327 | 0.353 | 0.445 | 0.272 | 0.319 | 0.364 | 0.412 | 0.464 |
| mean | 0.001 | 0.517 | 0.22533 | 0.268 | 0.327 | 0.3593 | 0.438 | 0.2703 | 0.31933 | 0.3633 | 0.411 | 0.468 |
| %  viability |  |  | 43.5848 | 51.838 | 63.25 | 69.504 | 84.72 | 52.289 | 61.7666 | 70.277 | 79.56 | 90.59 |

8/MCF10a 9/MCF10a

| 2 | 43.58 |
| --- | --- |
| 1.4 | 51.84 |

| 2 | 52.2888 |
| --- | --- |
| 1.3979 | 61.7666 |


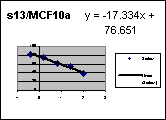

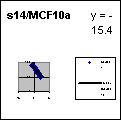


| 0.8 | 63.25 |
| --- | --- |
| 0.19 | 69.5 |
| -0.41 | 84.72 |

| 0.7959 | 70.2772 |
| --- | --- |
| 0.1931 | 79.5616 |
| -  0.4089 | 90.5867 |

IC50=

IC50=

|  | **Blank** | **CC** | **Sample No. 10/MCF10a** | | | | | **Sample No. 11/MCF10a** | | | | |
| --- | --- | --- | --- | --- | --- | --- | --- | --- | --- | --- | --- | --- |
|  | **1** | **2** | **3** | **4** | **5** | **6** | **7** | **8** | **9** | **10** | **11** | **12** |
| A | B | C | 100ug | 25ug | 6.3ug | 1.6ug | 0.4ug | 100ug | 25ug | 6.3ug | 1.6ug | 0.4ug |
| B | B | C | 100ug | 25ug | 6.25ug | 1.6ug | 0.4ug | 100ug | 25ug | 6.25ug | 1.6ug | 0.4ug |
| C | B | C | 100ug | 25ug | 6.25ug | 1.6ug | 0.4ug | 100ug | 25ug | 6.25ug | 1.6ug | 0.4ug |

ROBONIK P2000 Eia reader Wave

length: 450 nm

Reference: 630 nm

|  | **1** | **2** | **3** | **4** | **5** | **6** | **7** | **8** | **9** | **10** | **11** | **12** |
| --- | --- | --- | --- | --- | --- | --- | --- | --- | --- | --- | --- | --- |
| A | 0.001 | 0.532 | 0.245 | 0.286 | 0.331 | 0.382 | 0.438 | 0.265 | 0.314 | 0.361 | 0.414 | 0.454 |
| B | 0.001 | 0.548 | 0.239 | 0.285 | 0.335 | 0.377 | 0.452 | 0.258 | 0.325 | 0.364 | 0.418 | 0.461 |
| C | 0.001 | 0.561 | 0.244 | 0.292 | 0.337 | 0.375 | 0.466 | 0.261 | 0.319 | 0.366 | 0.425 | 0.467 |
| mean | 0.001 | 0.547 | 0.24267 | 0.2877 | 0.3343 | 0.378 | 0.452 | 0.2613 | 0.31933 | 0.3637 | 0.419 | 0.461 |
| %via |  |  | 44.3632 | 52.59 | 61.121 | 69.104 | 82.633 | 47.776 | 58.379 | 66.484 | 76.6 | 84.22 |


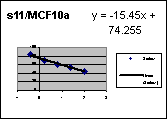

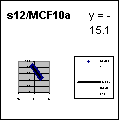
10/MCF10a 11/MCF10a

| 2 | 44.36  52.59  61.12  69.1  82.63 |
| --- | --- |
| 1.4 |  |
| 0.8 |  |
| 0.19 |  |
| -0.41 |  |
| IC50= | |
|  | |

| 2 | 47.7757 |
| --- | --- |
| 1.3979 | 58.379 |
| 0.7959 | 66.4839 |
| 0.1931 | 76.5996 |
| - |  |
| 0.4089 | 84.2169 |
| IC50= | |

|  | **Blank** | **CC** | **Sample No. 12/MCF10a** | | | | | **Sample No. 13/MCF10a** | | | | |
| --- | --- | --- | --- | --- | --- | --- | --- | --- | --- | --- | --- | --- |
|  | **1** | **2** | **3** | **4** | **5** | **6** | **7** | **8** | **9** | **10** | **11** | **12** |
| A | B | C | 100ug | 25ug | 6.3ug | 1.6ug | 0.4ug | 100ug | 25ug | 6.3ug | 1.6ug | 0.4ug |
| B | B | C | 100ug | 25ug | 6.25ug | 1.6ug | 0.4ug | 100ug | 25ug | 6.25ug | 1.6ug | 0.4ug |
| C | B | C | 100ug | 25ug | 6.25ug | 1.6ug | 0.4ug | 100ug | 25ug | 6.25ug | 1.6ug | 0.4ug |

ROBONIK P2000 Eia reader Wave

length: 450 nm

Reference: 630 nm

|  | **1** | **2** | **3** | **4** | **5** | **6** | **7** | **8** | **9** | **10** | **11** | **12** |
| --- | --- | --- | --- | --- | --- | --- | --- | --- | --- | --- | --- | --- |
| A | 0.001 | 0.567 | 0.231 | 0.309 | 0.353 | 0.432 | 0.467 | 0.302 | 0.357 | 0.412 | 0.464 | 0.509 |
| B | 0.001 | 0.571 | 0.219 | 0.314 | 0.358 | 0.431 | 0.462 | 0.292 | 0.362 | 0.424 | 0.461 | 0.511 |
| C | 0.001 | 0.568 | 0.225 | 0.316 | 0.355 | 0.431 | 0.459 | 0.296 | 0.361 | 0.419 | 0.458 | 0.51 |
| mean |  | 0.569 | 0.225 | 0.313 | 0.3553 | 0.4313 | 0.4627 | 0.2967 | 0.36 | 0.4183 | 0.461 | 0.51 |
| %via |  |  | 39.5662 | 55.041 | 62.485 | 75.85 | 81.36 | 52.169 | 63.306 | 73.564 | 81.07 | 89.68 |

12/MCF10a 13/MCF10a

39.57 52.1688

2

2


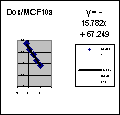


| 1.4 | 55.04  62.49  75.85  81.36 |
| --- | --- |
| 0.8 |  |
| 0.19 |  |
| -0.41 |  |
| IC50= | |
|  | |

| 1.3979 | 63.306 |
| --- | --- |
| 0.7959 | 73.5639 |
| 0.1931 | 81.0668 |
| - |  |
| 0.4089 | 89.6835 |
| IC50= | |

|  | **Blank** | **CC** | **Sample No. 14/MCF10a** | | | | | **Sample No. 15/MCF10a** | | | | |
| --- | --- | --- | --- | --- | --- | --- | --- | --- | --- | --- | --- | --- |
|  | **1** | **2** | **3** | **4** | **5** | **6** | **7** | **3** | **4** | **5** | **6** | **7** |
| A | B | C | 100ug | 25ug | 6.3ug | 1.6ug | 0.4ug | 100ug | 25ug | 6.3ug | 1.6ug | 0.4ug |
| B | B | C | 100ug | 25ug | 6.25ug | 1.6ug | 0.4ug | 100ug | 25ug | 6.25ug | 1.6ug | 0.4ug |
| C | B | C | 100ug | 25ug | 6.25ug | 1.6ug | 0.4ug | 100ug | 25ug | 6.25ug | 1.6ug | 0.4ug |

ROBONIK P2000 Eia reader Wave

length: 450 nm

Reference: 630 nm

|  | **1** | **2** | **3** | **4** | **5** | **6** | **7** | **3** | **4** | **5** | **6** | **7** |
| --- | --- | --- | --- | --- | --- | --- | --- | --- | --- | --- | --- | --- |
| A | 0.001 | 0.544 | 0.264 | 0.313 | 0.354 | 0.408 | 0.449 | 0.282 | 0.315 | 0.359 | 0.414 | 0.461 |
| B | 0.001 | 0.529 | 0.259 | 0.324 | 0.359 | 0.412 | 0.457 | 0.267 | 0.321 | 0.364 | 0.411 | 0.449 |
| C | 0.001 | 0.517 | 0.265 | 0.309 | 0.356 | 0.414 | 0.455 | 0.258 | 0.319 | 0.361 | 0.418 | 0.458 |
| mean |  | 0.53 | 0.26267 | 0.3153 | 0.3563 | 0.4113 | 0.4537 | 0.269 | 0.31833 | 0.3613 | 0.414 | 0.456 |
| %via |  |  | 49.5597 | 59.497 | 67.233 | 77.61 | 85.597 | 50.755 | 60.0629 | 68.176 | 78.18 | 86.04 |


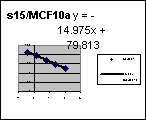

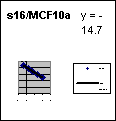
14/MCF10a 15/MCF10a

| 2 | 49.56  59.5  67.23  77.61  85.6 |
| --- | --- |
| 1.4 |  |
| 0.8 |  |
| 0.19 |  |
| -0.41 |  |
| IC50= | |
|  | |

| 2 | 50.7547 |
| --- | --- |
| 1.3979 | 60.0629 |
| 0.7959 | 68.1761 |
| 0.1931 | 78.1761 |
| - |  |
| 0.4089 | 86.0377 |
| IC50= | |

|  | **Blank** | **CC** | **Sample No. Dox/MCF10a** | | | | |
| --- | --- | --- | --- | --- | --- | --- | --- |
|  | **1** | **2** | **3** | **4** | **5** | **6** | **7** |
| A | B | C | 100ug | 25ug | 6.3ug | 1.6ug | 0.4ug |
| B | B | C | 100ug | 25ug | 6.25ug | 1.6ug | 0.4ug |
| C | B | C | 100ug | 25ug | 6.25ug | 1.6ug | 0.4ug |

ROBONIK P2000 Eia reader Wave

length: 450 nm

Reference: 630 nm

Dox/MCF10a

|  | **1** | **2** | **3** | **4** | **5** | **6** | **12** |
| --- | --- | --- | --- | --- | --- | --- | --- |
| A | 0.001 | 0.494 | 0.182 | 0.223 | 0.277 | 0.321 | 0.377 |
| B | 0.001 | 0.506 | 0.179 | 0.228 | 0.281 | 0.319 | 0.373 |
| C | 0.001 | 0.515 | 0.183 | 0.225 | 0.282 | 0.322 | 0.371 |
| mean |  | 0.505 | 0.1813 | 0.22533 | 0.28 | 0.321 | 0.374 |
| %via |  |  | 35.908 | 44.6205 | 55.446 | 63.5 | 73.99 |

| 2 | 35.9076 |
| --- | --- |
| 1.3979 | 44.6205 |
| 0.7959 | 55.4455 |
| 0.1931 | 63.4983 |
| - |  |
| 0.4089 | 73.9934 |
| IC50= | |

Researcher

Assay Samples Cell lines Ref.

Date Reader Kit used Solvent

: Dr.Feby Nabil email: [Fibytakla@gmail.com](mailto:Fibytakla@gmail.com) mob. 01224037280

: EGFR inh.assay

: 04 compounds

: ---

: ---

: 01-09-2021

: Tecan Spark Reader

: ---.

: DMSO

# Lab Report

| **ser** | **Compound** | | | **EGFR** | | Difference from Reference |
| --- | --- | --- | --- | --- | --- | --- |
|  | **code** | **MW** | **conc. ug/ml** | **IC50 ug/ml** | **Reference Erlotinib** |  |
| 1 | **5** |  |  | 0.104±0.0022 | 0.057±  0.0012 |  |
| 2 | **11** |  |  | 0.815±0.017 |  |  |
| 3 | **12** |  |  | 0.085±0.0018 |  |  |
| 4 | **14** |  |  | 0.908±0.019 |  |  |
| 5 |  |  |  |  | |  |


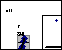

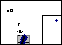

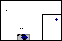

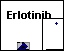


####
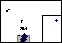
 Detailed results

**EGFR**

| code IC50 conc log | %inh | T2 | T1 | ∆T | RFU2 | RFU1 | ∆RFU | slope | K.Activity | EC |
| --- | --- | --- | --- | --- | --- | --- | --- | --- | --- | --- |

| 5 |  | 10 | 1 | 90 | 30 | 0 | 30 | 9.88 | 0 | 9.88 | 3.33333 | 11.85601 | 120 |
| --- | --- | --- | --- | --- | --- | --- | --- | --- | --- | --- | --- | --- | --- |
|  |  | 1 | 0 | 70 | 30 | 0 | 30 | 29.61 | 0 | 29.61 | 3.33333 | 35.53204 | 120 |
|  |  | 0.1 | -1 | 50 | 30 | 0 | 30 | 49.76 | 0 | 49.76 | 3.33333 | 59.71206 | 120 |
|  |  | 0.01 | -2 | 29 | 30 | 0 | 30 | 71.32 | 0 | 71.32 | 3.33333 | 85.58409 | 120 |
| EC |  |  |  | 0 | 30 | 0 | 30 | 100 | 0 | 100 | 3.33333 | 120 | 120 |
|  |  |  |  |  |  |  |  |  |  |  |  |  |  |
| code | IC50 | conc.ng/ml | log  conc | %inh | T2 | T1 | ∆T | RFU2 | RFU1 | ∆RFU | slope | K.Activity | EC |
| 11 |  | 10 | 1 | 76 | 30 | 0 | 30 | 23.72 | 0 | 23.72 | 3.33333 | 28.46403 | 120 |
|  |  | 1 | 0 | 54 | 30 | 0 | 30 | 45.99 | 0 | 45.99 | 3.33333 | 55.18806 | 120 |
|  |  | 0.1 | -1 | 23 | 30 | 0 | 30 | 77.03 | 0 | 77.03 | 3.33333 | 92.43609 | 120 |
|  |  | 0.01 | -2 | 7.8 | 30 | 0 | 30 | 92.15 | 0 | 92.15 | 3.33333 | 110.5801 | 120 |
| EC |  |  |  | 0 | 30 | 0 | 30 | 100 | 0 | 100 | 3.33333 | 120 | 120 |
|  |  |  |  |  |  |  |  |  |  |  |  |  |  |
| code | IC50 | conc.ng/ml | log  conc | %inh | T2 | T1 | ∆T | RFU2 | RFU1 | ∆RFU | slope | K.Activity | EC |
| 12 |  | 10 | 1 | 88 | 30 | 0 | 30 | 11.74 | 0 | 11.74 | 3.33333 | 14.08801 | 120 |
|  |  | 1 | 0 | 73 | 30 | 0 | 30 | 27.09 | 0 | 27.09 | 3.33333 | 32.50803 | 120 |
|  |  | 0.1 | -1 | 50 | 30 | 0 | 30 | 49.61 | 0 | 49.61 | 3.33333 | 59.53206 | 120 |
|  |  | 0.01 | -2 | 32 | 30 | 0 | 30 | 67.95 | 0 | 67.95 | 3.33333 | 81.54008 | 120 |
| EC |  |  |  | 0 | 30 | 0 | 30 | 100 | 0 | 100 | 3.33333 | 120 | 120 |
|  |  |  |  |  |  |  |  |  |  |  |  |  |  |
| code | IC50 | conc.ng/ml | log  conc | %inh | T2 | T1 | ∆T | RFU2 | RFU1 | ∆RFU | slope | K.Activity | EC |
| 14 |  | 10 | 1 | 78 | 30 | 0 | 30 | 21.82 | 0 | 21.82 | 3.33333 | 26.18403 | 120 |
|  |  | 1 | 0 | 44 | 30 | 0 | 30 | 56.39 | 0 | 56.39 | 3.33333 | 67.66807 | 120 |
|  |  | 0.1 | -1 | 24 | 30 | 0 | 30 | 76.46 | 0 | 76.46 | 3.33333 | 91.75209 | 120 |
|  |  | 0.01 | -2 | 18 | 30 | 0 | 30 | 82.23 | 0 | 82.23 | 3.33333 | 98.6761 | 120 |
| EC |  |  |  | 0 | 30 | 0 | 30 | 100 | 0 | 100 | 3.33333 | 120 | 120 |
|  |  |  |  |  |  |  |  |  |  |  |  |  |  |
|  |  |  | log |  |  |  |  |  |  |  |  |  |  |
| code | IC50 | conc.ng/ml | conc | %inh | T2 | T1 | ∆T | RFU2 | RFU1 | ∆RFU | slope | K.Activity | EC |
| **Erlotinib** |  | 10 | 1 | 94 | 30 | 0 | 30 | 6.46 | 0 | 6.46 | 3.33333 | 7.752008 | 120 |
|  |  | 1 | 0 | 81 | 30 | 0 | 30 | 19.42 | 0 | 19.42 | 3.33333 | 23.30402 | 120 |
|  |  | 0.1 | -1 | 58 | 30 | 0 | 30 | 41.76 | 0 | 41.76 | 3.33333 | 50.11205 | 120 |
|  |  | 0.01 | -2 | 30 | 30 | 0 | 30 | 69.61 | 0 | 69.61 | 3.33333 | 83.53208 | 120 |
| EC |  |  |  | 0 | 30 | 0 | 30 | 100 | 0 | 100 | 3.33333 | 120 | 120 |
|  |  |  |  |  |  |  |  |  |  |  |  |  |  |


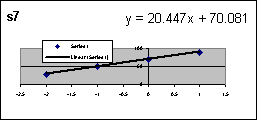


**5**


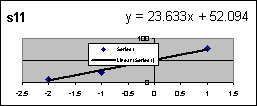


**11**


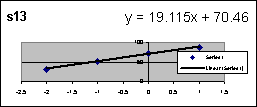


**12**


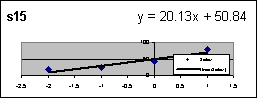


**14**


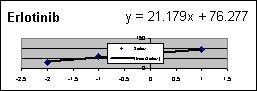


Researcher

Assay Samples Cell lines Ref.

Date Reader Kit used Solvent

: Dr.Feby Nabil email: [Fibytakla@gmail.com](mailto:Fibytakla@gmail.com) mob. 01224037280

: EGFR inh.assay

: 10 compounds

: ---

: ---

: 01-11-2021

: Tecan Spark Reader

: ---.

: DMSO

# Lab Report

| **ser** | **Compound** | | **EGFR** | **SD**  ± |
| --- | --- | --- | --- | --- |
|  | **code** | **MW**  **g/mol** | **IC50**  **ug/ml** |  |
| 1 | **2** |  | **0.256** | 0.016 |
| 2 | **3** |  | **0.322** | 0.02 |
| 3 | **4** |  | **0.143** | 0.009 |
| 4 | **6** |  | **0.056** | 0.003 |
| 5 | **7** |  | **0.138** | 0.008 |
| 6 | **8** |  | **0.493** | 0.03 |
| 7 | **9** |  | **0.059** | 0.004 |
| 8 | **10** |  | **0.042** | 0.003 |
| 9 | **13** |  | **0.123** | 0.007 |
| *** | **Erlotinib** | 393.436 | **0.033** | 0.002 |

#### Detailed Results

|  | EGFR |  |  |  |  |  |  |  |  |  |  | | | |
| --- | --- | --- | --- | --- | --- | --- | --- | --- | --- | --- | --- | --- | --- | --- |
|  |  |  |  |  |  |  |  |  |  |  |  |  |  |  |
|  | code |  | IC50 | conc | log | %inh | T2 | T1 | ∆T | RFU2 | RFU1 | ∆RFU | slope | K.Activity |
|  | 2 |  |  | 10 | 1 | 84 | 30 | 0 | 30 | 16.03 | 0 | 16.03 | 3.33333 | 19.23602 |
|  |  |  |  | 1 | 0 | 63 | 30 | 0 | 30 | 37.21 | 0 | 37.21 | 3.33333 | 44.65204 |
|  |  |  |  | 0.1 | -1 | 41 | 30 | 0 | 30 | 59.37 | 0 | 59.37 | 3.33333 | 71.24407 |
|  |  |  |  | 0.01 | -2 | 20 | 30 | 0 | 30 | 79.62 | 0 | 79.62 | 3.33333 | 95.5441 |
|  | EC |  |  |  |  | 0 | 30 | 0 | 30 | 100 | 0 | 100 | 3.33333 | 120 |
|  |  |  |  |  |  |  |  |  |  |  |  |  |  |  |
|  | code |  | IC50 | conc | log | %inh | T2 | T1 | ∆T | RFU2 | RFU1 | ∆RFU | slope | K.Activity |
|  | 3 |  |  | 10 | 1 | 85 | 30 | 0 | 30 | 14.53 | 0 | 14.53 | 3.33333 | 17.43602 |
|  |  |  |  | 1 | 0 | 60 | 30 | 0 | 30 | 39.51 | 0 | 39.51 | 3.33333 | 47.41205 |
|  |  |  |  | 0.1 | -1 | 36 | 30 | 0 | 30 | 64.09 | 0 | 64.09 | 3.33333 | 76.90808 |
|  |  |  |  | 0.01 | -2 | 17 | 30 | 0 | 30 | 82.54 | 0 | 82.54 | 3.33333 | 99.0481 |
|  | EC |  |  |  |  | 0 | 30 | 0 | 30 | 100 | 0 | 100 | 3.33333 | 120 |
|  |  |  |  |  |  |  |  |  |  |  |  |  |  |  |
|  | code |  | IC50 | conc | log | %inh | T2 | T1 | ∆T | RFU2 | RFU1 | ∆RFU | slope | K.Activity |
|  | 4 |  |  | 10 | 1 | 87 | 30 | 0 | 30 | 12.55 | 0 | 12.55 | 3.33333 | 15.06002 |
|  |  |  |  | 1 | 0 | 70 | 30 | 0 | 30 | 29.84 | 0 | 29.84 | 3.33333 | 35.80804 |
|  |  |  |  | 0.1 | -1 | 46 | 30 | 0 | 30 | 53.85 | 0 | 53.85 | 3.33333 | 64.62006 |
|  |  |  |  | 0.01 | -2 | 25 | 30 | 0 | 30 | 74.69 | 0 | 74.69 | 3.33333 | 89.62809 |
|  | EC |  |  |  |  | 0 | 30 | 0 | 30 | 100 | 0 | 100 | 3.33333 | 120 |
|  |  |  |  |  |  |  |  |  |  |  |  |  |  |  |
|  | code |  | IC50 | conc | log | %inh | T2 | T1 | ∆T | RFU2 | RFU1 | ∆RFU | slope | K.Activity |
|  | 6 |  |  | 10 | 1 | 91 | 30 | 0 | 30 | 8.91 | 0 | 8.91 | 3.33333 | 10.69201 |
|  |  |  |  | 1 | 0 | 74 | 30 | 0 | 30 | 25.71 | 0 | 25.71 | 3.33333 | 30.85203 |
|  |  |  |  | 0.1 | -1 | 57 | 30 | 0 | 30 | 42.95 | 0 | 42.95 | 3.33333 | 51.54005 |
|  |  |  |  | 0.01 | -2 | 34 | 30 | 0 | 30 | 65.72 | 0 | 65.72 | 3.33333 | 78.86408 |
|  | EC |  |  |  |  | 0 | 30 | 0 | 30 | 100 | 0 | 100 | 3.33333 | 120 |
|  |  |  |  |  |  |  |  |  |  |  |  |  |  |  |
|  | code |  | IC50 | conc | log | %inh | T2 | T1 | ∆T | RFU2 | RFU1 | ∆RFU | slope | K.Activity |
|  | **7** |  |  |  |  |  |  |  |  |  |  |  |  |  |
|  |  |  |  | 10 | 1 | 89 | 30 | 0 | 30 | 11.04 | 0 | 11.04 | 3.33333 | 13.24801 |
|  |  |  |  | 1 | 0 | 67 | 30 | 0 | 30 | 32.52 | 0 | 32.52 | 3.33333 | 39.02404 |
|  |  |  |  | 0.1 | -1 | 42 | 30 | 0 | 30 | 57.64 | 0 | 57.64 | 3.33333 | 69.16807 |
|  |  |  |  | 0.01 | -2 | 30 | 30 | 0 | 30 | 69.82 | 0 | 69.82 | 3.33333 | 83.78408 |
|  | EC |  |  |  |  | 0 | 30 | 0 | 30 | 100 | 0 | 100 | 3.33333 | 120 |
|  |  |  |  |  |  |  |  |  |  |  |  |  |  |  |
|  | code |  | IC50 | conc | log | %inh | T2 | T1 | ∆T | RFU2 | RFU1 | ∆RFU | slope | K.Activity |
|  | **8** |  |  | 10 | 1 | 83 | 30 | 0 | 30 | 17.13 | 0 | 17.13 | 3.33333 | 20.55602 |

EC

|  | 1 | 0 | 58 | 30 | 0 | 30 | 41.85 | 0 | 41.85 | 3.33333 | 50.22005 |
| --- | --- | --- | --- | --- | --- | --- | --- | --- | --- | --- | --- |
|  | 0.1 | -1 | 28 | 30 | 0 | 30 | 72.02 | 0 | 72.02 | 3.33333 | 86.42409 |
|  | 0.01 | -2 | 12 | 30 | 0 | 30 | 87.69 | 0 | 87.69 | 3.33333 | 105.2281 |
|  |  |  | 0 | 30 | 0 | 30 | 100 | 0 | 100 | 3.33333 | 120 |
|  |  |  |  |  |  |  |  |  |  |  |  |
| IC50 | conc | log | %inh | T2 | T1 | ∆T | RFU2 | RFU1 | ∆RFU | slope | K.Activity |
|  | 10 | 1 | 90 | 30 | 0 | 30 | 9.93 | 0 | 9.93 | 3.33333 | 11.91601 |
|  | 1 | 0 | 76 | 30 | 0 | 30 | 24.08 | 0 | 24.08 | 3.33333 | 28.89603 |
|  | 0.1 | -1 | 54 | 30 | 0 | 30 | 46.31 | 0 | 46.31 | 3.33333 | 55.57206 |
|  | 0.01 | -2 | 35 | 30 | 0 | 30 | 64.97 | 0 | 64.97 | 3.33333 | 77.96408 |
|  |  |  | 0 | 30 | 0 | 30 | 100 | 0 | 100 | 3.33333 | 120 |
|  |  |  |  |  |  |  |  |  |  |  |  |
| IC50 | conc | log | %inh | T2 | T1 | ∆T | RFU2 | RFU1 | ∆RFU | slope | K.Activity |
|  | 10 | 1 | 92 | 30 | 0 | 30 | 7.88 | 0 | 7.88 | 3.33333 | 9.456009 |
|  | 1 | 0 | 81 | 30 | 0 | 30 | 18.62 | 0 | 18.62 | 3.33333 | 22.34402 |
|  | 0.1 | -1 | 59 | 30 | 0 | 30 | 41.06 | 0 | 41.06 | 3.33333 | 49.27205 |
|  | 0.01 | -2 | 35 | 30 | 0 | 30 | 64.92 | 0 | 64.92 | 3.33333 | 77.90408 |
|  |  |  | 0 | 30 | 0 | 30 | 100 | 0 | 100 | 3.33333 | 120 |
|  |  |  |  |  |  |  |  |  |  |  |  |
| IC50 | conc | log | %inh | T2 | T1 | ∆T | RFU2 | RFU1 | ∆RFU | slope | K.Activity |
|  | 10 | 1 | 88 | 30 | 0 | 30 | 12.31 | 0 | 12.31 | 3.33333 | 14.77201 |
|  | 1 | 0 | 71 | 30 | 0 | 30 | 29.31 | 0 | 29.31 | 3.33333 | 35.17204 |
|  | 0.1 | -1 | 48 | 30 | 0 | 30 | 52.43 | 0 | 52.43 | 3.33333 | 62.91606 |
|  | 0.01 | -2 | 27 | 30 | 0 | 30 | 72.51 | 0 | 72.51 | 3.33333 | 87.01209 |
|  |  |  | 0 | 30 | 0 | 30 | 100 | 0 | 100 | 3.33333 | 120 |
|  |  |  |  |  |  |  |  |  |  |  |  |
| IC50 | conc | log | %inh | T2 | T1 | ∆T | RFU2 | RFU1 | ∆RFU | slope | K.Activity |
|  | 10 | 1 | 93 | 30 | 0 | 30 | 7.47 | 0 | 7.47 | 3.33333 | 8.964009 |
|  | 1 | 0 | 82 | 30 | 0 | 30 | 18.39 | 0 | 18.39 | 3.33333 | 22.06802 |
|  | 0.1 | -1 | 61 | 30 | 0 | 30 | 38.51 | 0 | 38.51 | 3.33333 | 46.21205 |
|  | 0.01 | -2 | 37 | 30 | 0 | 30 | 62.83 | 0 | 62.83 | 3.33333 | 75.39608 |
|  |  |  | 0 | 30 | 0 | 30 | 100 | 0 | 100 | 3.33333 | 120 |
|  |  |  |  |  |  |  |  |  |  |  |  |

code

**9**

EC

code

**10**

EC

code

**13**

EC

code

**Erlotinib**

EC


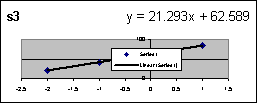


**2**


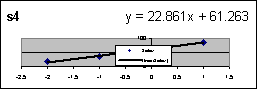


**3**


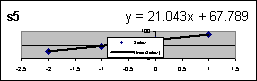


**4**


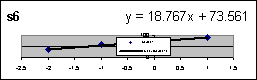


**6**


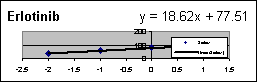


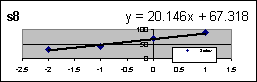


**7**


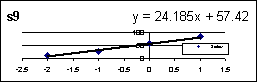


**8**


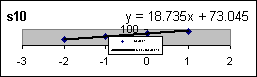


**9**


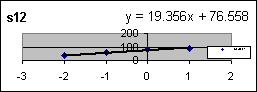


**10**


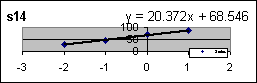


**13**

Researcher

Assay Samples Ref.

Date Reader Kit used Solvent

Assay samples

: Dr.Feby Nabil email: [Fibytakla@gmail.com](mailto:Fibytakla@gmail.com) mob. 01224037280

: Aromatase assay

: 11 compounds

: ---

: 14-12-2020

: ROBONIK P2000 EIA READER

: --

: DMSO

:---

wl 450 nm

# Lab Report

| **s** | Compound | | | **Aromatase** | SD  ± |
| --- | --- | --- | --- | --- | --- |
|  | **code** | **M.W**  **g/mol** | **conc uM** | **IC50**  **ug/ml** |  |
| 1 | **2** |  |  | 0.064 | 0.003 |
| **2** | **3** |  |  | 0.704 | 0.038 |
| **3** | **4** |  |  | 0.508 | 0.028 |
| 4 | **5** |  |  | 0.351 | 0.019 |
| 5 | **6** |  |  | 1.425 | 0.078 |
| 6 | **7** |  |  | 0.759 | 0.041 |
| 7 | **8** |  |  | 0.051 | 0.003 |
| 8 | **9** |  |  | 1.346 | 0.073 |
| 9 | **10** |  |  | 1.452 | 0.079 |
| 10 | **11** |  |  | 2.658 | 0.145 |
| 11 | **12** |  |  | 0.184 | 0.01 |
| 13 | **Exemestane** | 296.403 |  | 0.032 | 0.002 |

Detailed results

Aromatase

| code IC50 conc log | %inh | T2 | T1 | ∆T | RFU2 | RFU1 | ∆RFU | slope | K.Activity |
| --- | --- | --- | --- | --- | --- | --- | --- | --- | --- |

| **2** |  | 10 | 1 | 89 | 30 | 0 | 30 | 11.43 | 0 | 11.43 | 3.33333 | 13.71601 |
| --- | --- | --- | --- | --- | --- | --- | --- | --- | --- | --- | --- | --- |
|  |  | 1 | 0 | 68 | 30 | 0 | 30 | 31.51 | 0 | 31.51 | 3.33333 | 37.81204 |
|  |  | 0.1 | -1 | 54 | 30 | 0 | 30 | 46.38 | 0 | 46.38 | 3.33333 | 55.65606 |
|  |  | 0.01 | -2 | 37 | 30 | 0 | 30 | 63.27 | 0 | 63.27 | 3.33333 | 75.92408 |
| EC |  |  |  | 0 | 30 | 0 | 30 | 100 | 0 | 100 | 3.33333 | 120 |
|  |  |  |  |  |  |  |  |  |  |  |  |  |
|  |  |  | log |  |  |  |  |  |  |  |  |  |
| code | IC50 | conc.ng/ml | conc | %inh | T2 | T1 | ∆T | RFU2 | RFU1 | ∆RFU | slope | K.Activity |
| **3** |  | 10 | 1 | 75 | 30 | 0 | 30 | 24.92 | 0 | 24.92 | 3.33333 | 29.90403 |
|  |  | 1 | 0 | 50 | 30 | 0 | 30 | 49.69 | 0 | 49.69 | 3.33333 | 59.62806 |
|  |  | 0.1 | -1 | 31 | 30 | 0 | 30 | 68.54 | 0 | 68.54 | 3.33333 | 82.24808 |
|  |  | 0.01 | -2 | 16 | 30 | 0 | 30 | 84.16 | 0 | 84.16 | 3.33333 | 100.9921 |
| EC |  |  |  | 0 | 30 | 0 | 30 | 100 | 0 | 100 | 3.33333 | 120 |
|  |  |  |  |  |  |  |  |  |  |  |  |  |
|  |  |  | log |  |  |  |  |  |  |  |  |  |
| code | IC50 | conc.ng/ml | conc | %inh | T2 | T1 | ∆T | RFU2 | RFU1 | ∆RFU | slope | K.Activity |
| **4** |  | 10 | 1 | 81 | 30 | 0 | 30 | 19.13 | 0 | 19.13 | 3.33333 | 22.95602 |
|  |  | 1 | 0 | 55 | 30 | 0 | 30 | 44.62 | 0 | 44.62 | 3.33333 | 53.54405 |
|  |  | 0.1 | -1 | 30 | 30 | 0 | 30 | 69.88 | 0 | 69.88 | 3.33333 | 83.85608 |
|  |  | 0.01 | -2 | 15 | 30 | 0 | 30 | 84.62 | 0 | 84.62 | 3.33333 | 101.5441 |
| EC |  |  |  | 0 | 30 | 0 | 30 | 100 | 0 | 100 | 3.33333 | 120 |
|  |  |  |  |  |  |  |  |  |  |  |  |  |
|  |  |  | log |  |  |  |  |  |  |  |  |  |
| code | IC50 | conc.ng/ml | conc | %inh | T2 | T1 | ∆T | RFU2 | RFU1 | ∆RFU | slope | K.Activity |
| **6** |  | 10 | 1 | 77 | 30 | 0 | 30 | 22.61 | 0 | 22.61 | 3.33333 | 27.13203 |
|  |  | 1 | 0 | 42 | 30 | 0 | 30 | 57.67 | 0 | 57.67 | 3.33333 | 69.20407 |
|  |  | 0.1 | -1 | 10 | 30 | 0 | 30 | 89.66 | 0 | 89.66 | 3.33333 | 107.5921 |
|  |  | 0.01 | -2 | 3.9 | 30 | 0 | 30 | 96.07 | 0 | 96.07 | 3.33333 | 115.2841 |
| EC |  |  |  | 0 | 30 | 0 | 30 | 100 | 0 | 100 | 3.33333 | 120 |
|  |  |  |  |  |  |  |  |  |  |  |  |  |
| code | IC50 | conc.ng/ml | log conc | %inh | T2 | T1 | ∆T | RFU2 | RFU1 | ∆RFU | slope | K.Activity |
| ***5*** |  | 10 | 1 | 84 | 30 | 0 | 30 | 16.25 | 0 | 16.25 | 3.33333 | 19.50002 |
|  |  | 1 | 0 | 61 | 30 | 0 | 30 | 39.39 | 0 | 39.39 | 3.33333 | 47.26805 |
|  |  | 0.1 | -1 | 38 | 30 | 0 | 30 | 62.07 | 0 | 62.07 | 3.33333 | 74.48407 |
|  |  | 0.01 | -2 | 13 | 30 | 0 | 30 | 86.51 | 0 | 86.51 | 3.33333 | 103.8121 |
| EC |  |  |  | 0 | 30 | 0 | 30 | 100 | 0 | 100 | 3.33333 | 120 |
|  |  |  |  |  |  |  |  |  |  |  |  |  |
| code | IC50 | conc.ng/ml | log  conc | %inh | T2 | T1 | ∆T | RFU2 | RFU1 | ∆RFU | slope | K.Activity |
| **7** |  | 10 | 1 | 75 | 30 | 0 | 30 | 25.02 | 0 | 25.02 | 3.33333 | 30.02403 |
|  |  | 1 | 0 | 50 | 30 | 0 | 30 | 49.51 | 0 | 49.51 | 3.33333 | 59.41206 |

|  |  | 0.1 | -1 | 33 | 30 | 0 | 30 | 66.85 | 0 | 66.85 | 3.33333 | 80.22008 |
| --- | --- | --- | --- | --- | --- | --- | --- | --- | --- | --- | --- | --- |
|  |  | 0.01 | -2 | 8.3 | 30 | 0 | 30 | 91.69 | 0 | 91.69 | 3.33333 | 110.0281 |
| EC |  |  |  | 0 | 30 | 0 | 30 | 100 | 0 | 100 | 3.33333 | 120 |
|  |  |  |  |  |  |  |  |  |  |  |  |  |
| code | IC50 | conc.ng/ml | log  conc | %inh | T2 | T1 | ∆T | RFU2 | RFU1 | ∆RFU | slope | K.Activity |
| **8** |  | 10 | 1 | 89 | 30 | 0 | 30 | 11.05 | 0 | 11.05 | 3.33333 | 13.26001 |
|  |  | 1 | 0 | 73 | 30 | 0 | 30 | 26.61 | 0 | 26.61 | 3.33333 | 31.93203 |
|  |  | 0.1 | -1 | 56 | 30 | 0 | 30 | 43.88 | 0 | 43.88 | 3.33333 | 52.65605 |
|  |  | 0.01 | -2 | 36 | 30 | 0 | 30 | 64.12 | 0 | 64.12 | 3.33333 | 76.94408 |
| EC |  |  |  | 0 | 30 | 0 | 30 | 100 | 0 | 100 | 3.33333 | 120 |
|  |  |  |  |  |  |  |  |  |  |  |  |  |
|  |  |  | log |  |  |  |  |  |  |  |  |  |
| code | IC50 | conc.ng/ml | conc | %inh | T2 | T1 | ∆T | RFU2 | RFU1 | ∆RFU | slope | K.Activity |
| **9** |  | 10 | 1 | 72 | 30 | 0 | 30 | 28.01 | 0 | 28.01 | 3.33333 | 33.61203 |
|  |  | 1 | 0 | 45 | 30 | 0 | 30 | 54.63 | 0 | 54.63 | 3.33333 | 65.55607 |
|  |  | 0.1 | -1 | 21 | 30 | 0 | 30 | 79.46 | 0 | 79.46 | 3.33333 | 95.3521 |
|  |  | 0.01 | -2 | 6.2 | 30 | 0 | 30 | 93.82 | 0 | 93.82 | 3.33333 | 112.5841 |
| EC |  |  |  | 0 | 30 | 0 | 30 | 100 | 0 | 100 | 3.33333 | 120 |
|  |  |  |  |  |  |  |  |  |  |  |  |  |
|  |  |  | log |  |  |  |  |  |  |  |  |  |
| code | IC50 | conc.ng/ml | conc | %inh | T2 | T1 | ∆T | RFU2 | RFU1 | ∆RFU | slope | K.Activity |
| **10** |  | 10 | 1 | 76 | 30 | 0 | 30 | 23.96 | 0 | 23.96 | 3.33333 | 28.75203 |
|  |  | 1 | 0 | 38 | 30 | 0 | 30 | 62.11 | 0 | 62.11 | 3.33333 | 74.53207 |
|  |  | 0.1 | -1 | 20 | 30 | 0 | 30 | 79.93 | 0 | 79.93 | 3.33333 | 95.9161 |
|  |  | 0.01 | -2 | 4.3 | 30 | 0 | 30 | 95.74 | 0 | 95.74 | 3.33333 | 114.8881 |
| EC |  |  |  | 0 | 30 | 0 | 30 | 100 | 0 | 100 | 3.33333 | 120 |
|  |  |  |  |  |  |  |  |  |  |  |  |  |
|  |  |  | log |  |  |  |  |  |  |  |  |  |
| code | IC50 | conc.ng/ml | conc | %inh | T2 | T1 | ∆T | RFU2 | RFU1 | ∆RFU | slope | K.Activity |
| **11** |  | 10 | 1 | 71 | 30 | 0 | 30 | 28.79 | 0 | 28.79 | 3.33333 | 34.54803 |
|  |  | 1 | 0 | 32 | 30 | 0 | 30 | 67.54 | 0 | 67.54 | 3.33333 | 81.04808 |
|  |  | 0.1 | -1 | 8.9 | 30 | 0 | 30 | 91.11 | 0 | 91.11 | 3.33333 | 109.3321 |
|  |  | 0.01 | -2 | 2.6 | 30 | 0 | 30 | 97.42 | 0 | 97.42 | 3.33333 | 116.9041 |
| EC |  |  |  | 0 | 30 | 0 | 30 | 100 | 0 | 100 | 3.33333 | 120 |
|  |  |  |  |  |  |  |  |  |  |  |  |  |
|  |  |  | log |  |  |  |  |  |  |  |  |  |
| code | IC50 | conc.ng/ml | conc | %inh | T2 | T1 | ∆T | RFU2 | RFU1 | ∆RFU | slope | K.Activity |
| **12** |  | 10 | 1 | 87 | 30 | 0 | 30 | 13.06 | 0 | 13.06 | 3.33333 | 15.67202 |
|  |  | 1 | 0 | 61 | 30 | 0 | 30 | 38.72 | 0 | 38.72 | 3.33333 | 46.46405 |
|  |  | 0.1 | -1 | 43 | 30 | 0 | 30 | 56.79 | 0 | 56.79 | 3.33333 | 68.14807 |
|  |  | 0.01 | -2 | 27 | 30 | 0 | 30 | 72.83 | 0 | 72.83 | 3.33333 | 87.39609 |
| EC |  |  |  | 0 | 30 | 0 | 30 | 100 | 0 | 100 | 3.33333 | 120 |
|  |  |  |  |  |  |  |  |  |  |  |  |  |
| code | IC50 | conc.ng/ml | log  conc | %inh | T2 | T1 | ∆T | RFU2 | RFU1 | ∆RFU | slope | K.Activity |
| **Exemestane** |  | 10 | 1 | 92 | 30 | 0 | 30 | 8.41 | 0 | 8.41 | 3.33333 | 10.09201 |
|  |  | 1 | 0 | 78 | 30 | 0 | 30 | 21.59 | 0 | 21.59 | 3.33333 | 25.90803 |
|  |  | 0.1 | -1 | 58 | 30 | 0 | 30 | 42.06 | 0 | 42.06 | 3.33333 | 50.47205 |

EC

| 0.01 | -2 | 41 | 30 | 0 | 30 | 58.72 | 0 | 58.72 | 3.33333 | 70.46407 |
| --- | --- | --- | --- | --- | --- | --- | --- | --- | --- | --- |
|  |  | 0 | 30 | 0 | 30 | 100 | 0 | 100 | 3.33333 | 120 |
|  |  |  |  |  |  |  |  |  |  |  |
|  |  |  |  |  |  |  |  |  |  |  |

2

3

4

6

5

7

8

9

10

11

**Researcher**

Assay Samples cell line Ref.

Date Reader Kit used Solvent

Assay samples

: Dr.Feby Nabil

: Cell Cycle Analysis

: 01 samples

: ---

: ---

: 10-10-2021

: BD FACSCalibur

email: [Fibytakla@gmail.com](mailto:Fibytakla@gmail.com) mob. 01224037280

: ab139418_Propidium Iodide Flow Cytometry Kit/BD

: DMSO

: Cell culture

# Lab Report

| **s e r** | Sample data | | Results DNA content | | | | |  |
| --- | --- | --- | --- | --- | --- | --- | --- | --- |
|  | **code** | **IC50**  **uM** | **%G0-G1** | **%S** | **%G2/M** | **%Pre-G1** | **Comment** |  |
| 1 | **8/MCF7** |  | 55.83 | 39.85 | 4.32 | 31.19 | cell growth arrest@ G1/S |  |
| 3 | **cont.MCF7** |  | 52.93 | 29.91 | 17.16 | 1.67 | --- |  |

|  |  |  | **Apoptosis** | | |  |
| --- | --- | --- | --- | --- | --- | --- |
| **s** | **code** | **conc** | Total | Early | Late | **Necrosis** |
| 1 | **8/MCF7** |  | 31.19 | 4.94 | 19.66 | 4.94 |
| 3 | **cont.MCF7** |  | 1.67 | 0.25 | 0.19 | 1.23 |

**Detailed results**

8

**Researcher**

Assay Samples cell line Ref.

Date Reader Kit used Solvent

Assay samples

: Dr.Feby Nabil

: Cell Cycle Analysis

: 02 samples

: ---

: ---

: 18-01-2022

: BD FACSCalibur

email: [Fibytakla@gmail.com](mailto:Fibytakla@gmail.com) mob. 01224037280

: ab139418_Propidium Iodide Flow Cytometry Kit/BD

: DMSO

: Cell culture

# Lab Report

| **s e r** | Sample data | | Results DNA content | | | | |  |
| --- | --- | --- | --- | --- | --- | --- | --- | --- |
|  | **code** | **IC50**  **uM** | **%G0-G1** | **%S** | **%G2/M** | **%Pre-G1** | **Comment** |  |
| 1 | **10/MDA** |  | 51.97 | 43.82 | 4.21 | 33.43 | cell growth arrest@ S |  |
| 2 | **12/MDA** |  | 63.58 | 29.97 | 6.45 | 46.28 | cell growth arrest@ G1/S |  |
| 3 | **cont.MDA** |  | 61.39 | 27.51 | 11.1 | 2.06 | --- |  |

|  |  |  | **Apoptosis** | | |  |
| --- | --- | --- | --- | --- | --- | --- |
| **s** | **code** | **conc** | Total | Early | Late | **Necrosis** |
| 1 | **10/MDA** |  | 33.43 | 9.61 | 17.83 | 5.99 |
| 2 | **12/MDA** |  | 46.28 | 16.75 | 23.02 | 6.51 |
| 3 | **cont.MDA** |  | 2.06 | 0.64 | 0.18 | 1.24 |

**Detailed results**

Researcher

Assay Samples Cells Ref.

Date Reader Kit used Solvent

Assay samples

: Dr.Feby Nabil

: BAX enzyme assay

: 01 sample

: ---

: --

: 10-10-2021

email: [Fibytakla@gmail.com](mailto:Fibytakla@gmail.com) mob. 01224037280

: ROBONIK P2000 ELISA READER

wl 450 nm

: DRG® Human Bax ELISA (EIA-4487) (96 tests)

: DMSO

: Cell culture supernatant

# Lab Report

| **ser** | **Compound** | | | **BAX** | **FLD** |
| --- | --- | --- | --- | --- | --- |
|  | **code** | **M.W**  **g/mol** | **IC50**  **ug/ml** | **Pg/**ml |  |
| 1 | **S8/MCF7** |  |  | **164.5**±5.58 | **5.27** |
| 3 | **cont.MCF7** |  |  | **31.19**±1.39 | **1** |

Detailed results

| **STANDARDS** | Pg/ml |
| --- | --- |
| St.1 | 2000 |
| St.2 | 1000 |
| St.3 | 500 |
| St.4 | 250 |
| St.5 | 125 |
| St.6 | 62.5 |

Plate map

3

2.5

**Measurement**

2

1.5

1

0.5

0

**Bax**

4PL Standard

10 100 1000 10000

**Concentration**

|  | **1** | **2** | 3 | 4 | 5 | 6 | 7 | 8 | 9 | 10 | 11 | 12 |
| --- | --- | --- | --- | --- | --- | --- | --- | --- | --- | --- | --- | --- |
| A | St.1 | **S8/MCF7** | **cont.MCF7** | -- | -- | -- | -- | -- | -- | -- | -- | -- |
| B | St.2 | **S8/MCF7** |  | -- | -- | -- | -- | -- | -- | -- | -- | -- |
| C | St.3 | **S8/MCF7** |  | -- | -- | -- | -- | -- | -- | -- | -- | -- |
| G | St.0 | **cont.MCF7** | -- | -- | -- | -- | -- | -- | -- | -- | -- | -- |
| H | B | **cont.MCF7** | -- | -- | -- | -- | -- | -- | -- | -- | -- | -- |

Samples OD results

|  | **1** | **2** | 3 | 4 | 5 | 6 | 7 | 8 | 9 | 10 | 11 | 12 |
| --- | --- | --- | --- | --- | --- | --- | --- | --- | --- | --- | --- | --- |
| A | 2.481 | 0.297 | 0.088 | 0 | 0 | 0 | 0 | 0 | 0 | 0 | 0 | 0 |
| B | 1.795 | 0.309 | 0.131 | 0 | 0 | 0 | 0 | 0 | 0 | 0 | 0 | 0 |
| C | 1.045 | 0.317 | 0.119 | 0 | 0 | 0 | 0 | 0 | 0 | 0 | 0 | 0 |
| D | 0.438 | 0.449 | 0.143 | 0 | 0 | 0 | 0 | 0 | 0 | 0 | 0 | 0 |
| E | 0.255 | 0.466 | 0 | 0 | 0 | 0 | 0 | 0 | 0 | 0 | 0 | 0 |
| F | 0.167 | 0.481 | 0 | 0 | 0 | 0 | 0 | 0 | 0 | 0 | 0 | 0 |
| G | 0.122 | 0.091 | 0 | 0 | 0 | 0 | 0 | 0 | 0 | 0 | 0 | 0 |
| H | 0.007 | 0.125 | 0 | 0 | 0 | 0 | 0 | 0 | 0 | 0 | 0 | 0 |

| **Calibrator** | **Wells** | **Conc.** | **Raw (Corrected)** | **Backfit** | **Recovery**  **%** |
| --- | --- | --- | --- | --- | --- |
| Standard1 | A1 | 2000 | 2.47 | 2014 | 100.7 |
| Standard2 | B1 | 1000 | 1.79 | 985.4 | 98.54 |
| Standard3 | C1 | 500 | 1.04 | 515.6 | 103.1 |
| Standard4 | D1 | 250 | 0.431 | 231.7 | 92.66 |
| Standard5 | E1 | 125 | 0.248 | 134.1 | 107.3 |
| Standard6 | F1 | 62.5 | 0.16 | 71.97 | 115.2 |
| Standard7 | G1 | 31.25 | 0.115 | 19.65 | 62.88 |

| **Sample** | **Wells** | **Raw** | **Background Corrected** | **Conc.** | **Conc. (Average)** | **%CV** | **SD** | **SEM** |
| --- | --- | --- | --- | --- | --- | --- | --- | --- |
| Control1 | A3 | 0.088 | 0.0943 | 28.41 | 31.19 | - | 1.39 | 0 |
|  | G2 | 0.091 |  | 30.04 |  |  |  |  |
|  | H2 | 0.125 |  | 35.11 |  |  |  |  |
| S8/MCF7 | A2 | 0.297 | 0.301 | 158.6 | 164.5 | 3.39 | 5.58 | 3.22 |
|  | B2 | 0.309 |  | 165.3 |  |  |  |  |
|  | C2 | 0.317 |  | 169.7 |  |  |  |  |
| Blank | H1 | 0.007 | 0 | <  Curve | - | - | - | - |

**Bax**

**400.0**

**300.0**

**pg/ml 200.0**

**100.0**

**0.0**

**Ser**

Researcher

Assay Samples Cells Ref.

Date Reader Kit used Solvent

Assay samples

: Dr.Feby Nabil

email: [Fibytakla@gmail.com](mailto:Fibytakla@gmail.com) mob. 01224037280

: BAX enzyme assay

: 03 samples

: MDA

: --

: 18-01-2022

: ROBONIK P2000 ELISA READER

wl 450 nm

: DRG® Human Bax ELISA (EIA-4487) (96 tests)

: DMSO

: Cell culture supernatant

# Lab Report

| **ser** | **Compound** | | | **BAX** | **FLD** |
| --- | --- | --- | --- | --- | --- |
|  | **code** | **M.W**  **g/mol** | **cells** | **Pg/ml** |  |
| 1 | **s10/MDA** |  |  | **248.9**±7.88 | **3.6** |
| 2 | **s12/MDA** |  |  | **360.4**±11.8 | **5.22** |
| 3 | **cont.MDA** | --- |  | **69.05**±3.22 | **1** |

Detailed results

2.5

2

**BAX**

4PL Standard

Plate map

1.5

1

**Measurement**

| **STANDARDS** | Conc. Pg/ml |
| --- | --- |
| St.1 | 2000 |
| St.2 | 1000 |
| St.3 | 500 |
| St.4 | 250 |
| St.5 | 125 |
| St.6 | 62.5 |

0.5

0

10

100

**Concentration**

1000

10000

|  | **1** | **2** | 3 | 4 | 5 | 6 | 7 | 8 | 9 | 10 | 11 | 12 |
| --- | --- | --- | --- | --- | --- | --- | --- | --- | --- | --- | --- | --- |
| A | St.1 | s10 | -- | -- | -- | -- | -- | -- | -- | -- | -- | -- |
| B | St.2 | s10 | -- | -- | -- | -- | -- | -- | -- | -- | -- | -- |
| C | St.3 | s12 | -- | -- | -- | -- | -- | -- | -- | -- | -- | -- |
| D | St.4 | s12 | -- | -- | -- | -- | -- | -- | -- | -- | -- | -- |
| E | St.5 | cont | -- | -- | -- | -- | -- | -- | -- | -- | -- | -- |
| F | St.6 | cont | -- | -- | -- | -- | -- | -- | -- | -- | -- | -- |
| G | St.0 | -- | -- | -- | -- | -- | -- | -- | -- | -- | -- | -- |
| H | B | -- | -- | -- | -- | -- | -- | -- | -- | -- | -- | -- |

Samples OD results

|  | **1** | **2** | 3 | 4 | 5 | 6 | 7 | 8 | 9 | 10 | 11 | 12 |
| --- | --- | --- | --- | --- | --- | --- | --- | --- | --- | --- | --- | --- |
| A | 2.361 | 0.392 | 0 | 0 | 0 | 0 | 0 | 0 | 0 | 0 | 0 | 0 |
| B | 1.627 | 0.376 | 0 | 0 | 0 | 0 | 0 | 0 | 0 | 0 | 0 | 0 |
| C | 0.806 | 0.577 | 0 | 0 | 0 | 0 | 0 | 0 | 0 | 0 | 0 | 0 |
| D | 0.391 | 0.548 | 0 | 0 | 0 | 0 | 0 | 0 | 0 | 0 | 0 | 0 |
| E | 0.255 | 0.139 | 0 | 0 | 0 | 0 | 0 | 0 | 0 | 0 | 0 | 0 |
| F | 0.181 | 0.114 | 0 | 0 | 0 | 0 | 0 | 0 | 0 | 0 | 0 | 0 |
| G | 0.007 | 0 | 0 | 0 | 0 | 0 | 0 | 0 | 0 | 0 | 0 | 0 |
| H | 0.016 | 0 | 0 | 0 | 0 | 0 | 0 | 0 | 0 | 0 | 0 | 0 |

| **Calibrator** | **Wells** | **Conc.** | **Raw (Corrected)** | **Backfit** | **Recovery**  **%** |
| --- | --- | --- | --- | --- | --- |
| Standard1 | A1 | 2000 | 2.35 | 1996 | 99.82 |
| Standard2 | B1 | 1000 | 1.62 | 1004 | 100.4 |
| Standard3 | C1 | 500 | 0.795 | 495.1 | 99.03 |
| Standard4 | D1 | 250 | 0.38 | 253.7 | 101.5 |
| Standard5 | E1 | 125 | 0.244 | 142.6 | 114.1 |
| Standard6 | F1 | 62.5 | 0.169 | <  Curve | - |

| **Sample** | **Wells** | **Raw** | **Background Corrected** | **Conc.** | **Conc. (Average)** | **%CV** | **SD** | **SEM** |
| --- | --- | --- | --- | --- | --- | --- | --- | --- |
| Control | E2  F2 | 0.139  0.114 | 0.115 | 72.18  65.92 | 69.05 | 1.94 | 3.22 | 2.27 |
| s10 | A2  B2 | 0.392  0.376 | 0.373 | 254.4  243.3 | 248.9 | 3.17 | 7.88 | 5.57 |
| s12 | C2  D2 | 0.577  0.548 | 0.551 | 368.8  352 | 360.4 | 3.29 | 11.8 | 8.38 |
| Blank | G1  H1 | 0.007  0.016 | 0 | <  Curve  <  Curve | - | - | - | - |

Researcher

Assay Samples Cell line Ref.

Date Reader Kit used Solvent

Assay samples

: Dr.Feby Nabil

email: [Fibytakla@gmail.com](mailto:Fibytakla@gmail.com) mob. 01224037280

: BCL2 enzyme assay

: 01 sample

: --

: ---

: 10-10-2021

: ROBONIK P2000ELISA READER

wl 450 nm

: Invitrogen Zymed® Bcl-2 ELISA Kit (96 tests)

: DMSO

: Cell culture

# Lab Report

| **ser** | **Compound** | | | **BCL2** | **FLD** |
| --- | --- | --- | --- | --- | --- |
|  | **code** | **M.Wt** | **IC50**  **ug/ml** | **conc. ng/ml** |  |
| 1 | **S8/MCF7** |  |  | **3.291**±0.36 | **0.44** |
| 3 | **cont.MCF7** |  |  | **7.438**±0.08 | **1** |

Detailed results

###### Bcl2

4PL Standard

| **STANDARDS** | Conc. ng/ml |
| --- | --- |
| St.1 | 32.0 |
| St.2 | 16.0 |
| St.3 | 8.0 |
| St.4 | 4.0 |
| St.5 | 2.0 |
| St.6 | 1.0 |

2.5

2

**Measurement**

1.5

1

Plate map

0.5

0

0.1

1 10

**Concentration**

100

|  | **1** | **2** | 3 | 4 | 5 | 6 | 7 | 8 | 9 | 10 | 11 | 12 |
| --- | --- | --- | --- | --- | --- | --- | --- | --- | --- | --- | --- | --- |
| A | St.1 | **S8/MCF7** | **cont.MCF7** | -- | -- | -- | -- | -- | -- | -- | -- | -- |
| B | St.2 | **S8/MCF7** |  | -- | -- | -- | -- | -- | -- | -- | -- | -- |
| C | St.3 | **S8/MCF7** |  | -- | -- | -- | -- | -- | -- | -- | -- | -- |
| G | St.0 | **cont.MCF7** | -- | -- | -- | -- | -- | -- | -- | -- | -- | -- |
| H | B | **cont.MCF7** | -- | -- | -- | -- | -- | -- | -- | -- | -- | -- |

Samples OD results

|  | 1 | 2 | 3 | 4 | 5 | 6 | 7 | 8 | 9 | 10 | 11 | 12 |
| --- | --- | --- | --- | --- | --- | --- | --- | --- | --- | --- | --- | --- |
| A | 2.272 | 0.331 | 0.659 | 0 | 0 | 0 | 0 | 0 | 0 | 0 | 0 | 0 |
| B | 1.407 | 0.279 | 0.834 | 0 | 0 | 0 | 0 | 0 | 0 | 0 | 0 | 0 |
| C | 0.669 | 0.291 | 0.828 | 0 | 0 | 0 | 0 | 0 | 0 | 0 | 0 | 0 |
| D | 0.374 | 0.513 | 0.851 | 0 | 0 | 0 | 0 | 0 | 0 | 0 | 0 | 0 |
| E | 0.239 | 0.487 | 0 | 0 | 0 | 0 | 0 | 0 | 0 | 0 | 0 | 0 |
| F | 0.177 | 0.528 | 0 | 0 | 0 | 0 | 0 | 0 | 0 | 0 | 0 | 0 |
| G | 0.091 | 0.648 | 0 | 0 | 0 | 0 | 0 | 0 | 0 | 0 | 0 | 0 |
| H | 0.012 | 0.661 | 0 | 0 | 0 | 0 | 0 | 0 | 0 | 0 | 0 | 0 |

| **Calibrator** | **Wells** | **Conc.** | **Raw (Corrected)** | **Backfit** | **Recovery**  **%** |
| --- | --- | --- | --- | --- | --- |
| Standard1 | A1 | 32 | 2.26 | 31.9 | 99.68 |
| Standard2 | B1 | 16 | 1.4 | 16.25 | 101.6 |

| Standard3 | C1 | 8 | 0.657 | 7.581 | 94.76 |
| --- | --- | --- | --- | --- | --- |
| Standard4 | D1 | 4 | 0.362 | 4.227 | 105.7 |
| Standard5 | E1 | 2 | 0.227 | 2.443 | 122.1 |
| Standard6 | F1 | 1 | 0.165 | 1.427 | 142.7 |
| Standard7 | G1 | 0.5 | 0.079 | <  Curve | - |

| **Sample** | **Wells** | **Raw** | **Background Corrected** | **Conc.** | **Conc. (Average)** | **%CV** | **SD** | **SEM** |
| --- | --- | --- | --- | --- | --- | --- | --- | --- |
| Control1 | A3 | 0.659 | 0.644 | 7.471 | 7.438 | 1.04 | 0.077 | 0.0445 |
|  | G2 | 0.648 |  | 7.35 |  |  |  |  |
|  | H2 | 0.661 |  | 7.493 |  |  |  |  |
| S8/MCF7 | A2 | 0.331 | 0.288 | 3.693 | 3.291 | 10.8 | 0.357 | 0.206 |
|  | B2 | 0.279 |  | 3.01 |  |  |  |  |
|  | C2 | 0.291 |  | 3.172 |  |  |  |  |
| Blank | H1 | 0.012 | 0 | <  Curve | - | - | - | - |

**Bcl2**

**12.000**

**10.000**

**8.000**

**ng/ml 6.000**

**4.000**

**2.000**

**0.000**

**Ser**

Researcher

Assay Samples Cell line Ref.

Date Reader Kit used Solvent

Assay samples

: Dr.Feby Nabil

email: [Fibytakla@gmail.com](mailto:Fibytakla@gmail.com) mob. 01224037280

: BCL2 enzyme assay

: 02 samples

: --

: ---

: 18-01-2022

: ROBONIK P2000ELISA READER

wl 450 nm

: Invitrogen Zymed® Bcl-2 ELISA Kit (96 tests)

: DMSO

: Cell culture

# Lab Report

| **ser** | **Compound** | | | **BCL2** | **FLD** |
| --- | --- | --- | --- | --- | --- |
|  | **code** | **M.Wt** | **IC50**  **ug/ml** | **conc. ng/ml** |  |
| 1 | **s10/MDA** |  |  | **4.088**±0.058 | **0.377** |
| 2 | **s12/MDA** |  |  | **2.934**±0.154 | **0.271** |
| 3 | **cont.MDA** |  |  | **10.83**±0.046 | **1** |

Detailed results

###### Bcl2

4PL Standard

| **STANDARDS** | Conc. ng/ml |
| --- | --- |
| St.1 | 32.0 |
| St.2 | 16.0 |
| St.3 | 8.0 |
| St.4 | 4.0 |
| St.5 | 2.0 |
| St.6 | 1.0 |

2.5

2

**Measurement**

1.5

1

Plate map

0.5

0

0.1

1 10

**Concentration**

100

|  | **1** | **2** | 3 | 4 | 5 | 6 | 7 | 8 | 9 | 10 | 11 | 12 |
| --- | --- | --- | --- | --- | --- | --- | --- | --- | --- | --- | --- | --- |
| A | St.1 | s10 | -- | -- | -- | -- | -- | -- | -- | -- | -- | -- |
| B | St.2 | s10 | -- | -- | -- | -- | -- | -- | -- | -- | -- | -- |
| C | St.3 | s12 | -- | -- | -- | -- | -- | -- | -- | -- | -- | -- |
| D | St.4 | s12 | -- | -- | -- | -- | -- | -- | -- | -- | -- | -- |
| E | St.5 | cont | -- | -- | -- | -- | -- | -- | -- | -- | -- | -- |
| F | St.6 | cont | -- | -- | -- | -- | -- | -- | -- | -- | -- | -- |
| G | St.0 | -- | -- | -- | -- | -- | -- | -- | -- | -- | -- | -- |
| H | B | -- | -- | -- | -- | -- | -- | -- | -- | -- | -- | -- |

Samples OD results

|  | 1 | 2 | 3 | 4 | 5 | 6 | 7 | 8 | 9 | 10 | 11 | 12 |
| --- | --- | --- | --- | --- | --- | --- | --- | --- | --- | --- | --- | --- |
| A | 2.272 | 0.355 | 0 | 0 | 0 | 0 | 0 | 0 | 0 | 0 | 0 | 0 |
| B | 1.407 | 0.361 | 0 | 0 | 0 | 0 | 0 | 0 | 0 | 0 | 0 | 0 |
| C | 0.669 | 0.275 | 0 | 0 | 0 | 0 | 0 | 0 | 0 | 0 | 0 | 0 |
| D | 0.374 | 0.288 | 0 | 0 | 0 | 0 | 0 | 0 | 0 | 0 | 0 | 0 |
| E | 0.239 | 0.955 | 0 | 0 | 0 | 0 | 0 | 0 | 0 | 0 | 0 | 0 |
| F | 0.177 | 0.949 | 0 | 0 | 0 | 0 | 0 | 0 | 0 | 0 | 0 | 0 |
| G | 0.091 | 0 | 0 | 0 | 0 | 0 | 0 | 0 | 0 | 0 | 0 | 0 |
| H | 0.012 | 0 | 0 | 0 | 0 | 0 | 0 | 0 | 0 | 0 | 0 | 0 |

| **Calibrator** | **Wells** | **Conc.** | **Raw (Corrected)** | **Backfit** | **Recovery**  **%** |
| --- | --- | --- | --- | --- | --- |
| Standard1 | A1 | 32 | 2.26 | 31.9 | 99.68 |
| Standard2 | B1 | 16 | 1.4 | 16.25 | 101.6 |
| Standard3 | C1 | 8 | 0.657 | 7.581 | 94.76 |
| Standard4 | D1 | 4 | 0.362 | 4.227 | 105.7 |
| Standard5 | E1 | 2 | 0.227 | 2.443 | 122.1 |
| Standard6 | F1 | 1 | 0.165 | 1.427 | 142.7 |
| Standard7 | G1 | 0.5 | 0.079 | < Curve | - |

| **Sample** | **Wells** | **Raw** | **Background Corrected** | **Conc.** | **Conc. (Average)** | **%CV** | **SD** | **SEM** |
| --- | --- | --- | --- | --- | --- | --- | --- | --- |
| Control | E2  F2 | 0.955  0.949 | 0.901 | 10.86  10.8 | 10.83 | 0.425 | 0.046 | 0.0325 |
| s10 | A2  B2 | 0.355  0.361 | 0.307 | 4.046  4.129 | 4.088 | 1.44 | 0.0587 | 0.0415 |
| s12 | C2  D2 | 0.275  0.288 | 0.23 | 2.826  3.043 | 2.934 | 5.23 | 0.154 | 0.109 |
| Blank | G1  H1 | 0.091  0.012 | 0 | <  Curve  <  Curve | - | - | - | - |

Researcher

Assay Samples cell line Ref.

Date Reader Kit used Solvent

Assay samples

: Dr.Feby Nabil

email: [Fibytakla@gmail.com](mailto:Fibytakla@gmail.com) mob. 01224037280

: Caspase 9 enzyme assay

: 01 sample

: ---

: ---

: 10-10-2021

: ROBONIK P2000 Eliza Reader wl 450 nm

: Invitrogen EIA kit Human caspase9 (active) KHO1091 (96 tests)

: DMSO

: Cell culture supernatant

# Lab Report

| **Ser** | **Compound** | | | **Casp9** | **FLD** |
| --- | --- | --- | --- | --- | --- |
|  | **code** | **Cells** | **IC50**  **ug/ml** | **ng/ml** |  |
| 1 | **S8/MCF7** |  |  | 30.39±2.56 | 6.71 |
| 3 | **cont.MCF7** |  |  | 4.528±0.61 | 1 |

Detailed results

**Casp9**

4PL Standard

| **STANDARDS**  **Ca9** | Conc. ng/ml |
| --- | --- |
| St.1 | 100 |
| St.2 | 50 |
| St.3 | 25 |
| St.4 | 12.5 |
| St.5 | 6.3 |
| St.6 | 3.1 |
| St.7 | 1.6 |

Plate map

2.5

2

**Measurement**

1.5

1

0.5

0

1

10

**Concentration**

100

|  | 1 | 2 | 3 | 4 | 5 | 6 | 7 | 8 | 9 | 10 | 11 | 12 |
| --- | --- | --- | --- | --- | --- | --- | --- | --- | --- | --- | --- | --- |
| A | st1 | **S8/MCF7** | **cont.MCF7** | - | - | - | - | - | - | - | - | - |
| B | st2 | **S8/MCF7** |  | - | - | - | - | - | - | - | - | - |
| C | st3 | **S8/MCF7** |  | - | - | - | - | - | - | - | - | - |
| G | st7 | **cont.MCF7** | - | - | - | - | - | - | - | - | - | - |
| H | B | **cont.MCF7** | - | - | - | - | - | - | - | - | - | - |

ODs

|  | 1 | 2 | 3 | 4 | 5 | 6 | 7 | 8 | 9 | 10 | 11 | 12 |
| --- | --- | --- | --- | --- | --- | --- | --- | --- | --- | --- | --- | --- |
| A | 2.086 | 0.654 | 0.142 | 0 | 0 | 0 | 0 | 0 | 0 | 0 | 0 | 0 |
| B | 1.244 | 0.772 | 0.128 | 0 | 0 | 0 | 0 | 0 | 0 | 0 | 0 | 0 |
| C | 0.585 | 0.777 | 0.097 | 0 | 0 | 0 | 0 | 0 | 0 | 0 | 0 | 0 |
| D | 0.263 | 0.476 | 0.114 | 0 | 0 | 0 | 0 | 0 | 0 | 0 | 0 | 0 |
| E | 0.206 | 0.487 | 0 | 0 | 0 | 0 | 0 | 0 | 0 | 0 | 0 | 0 |
| F | 0.152 | 0.521 | 0 | 0 | 0 | 0 | 0 | 0 | 0 | 0 | 0 | 0 |
| G | 0.081 | 0.138 | 0 | 0 | 0 | 0 | 0 | 0 | 0 | 0 | 0 | 0 |
| H | 0.016 | 0.161 | 0 | 0 | 0 | 0 | 0 | 0 | 0 | 0 | 0 | 0 |

| **Calibrator** | **Wells** | **Conc.** | **Raw (Corrected)** | **Backfit** | **Recovery**  **%** |
| --- | --- | --- | --- | --- | --- |
| Standard1 | A1 | 100 | 2.07 | 99.92 | 99.92 |
| Standard2 | B1 | 50 | 1.23 | 50.16 | 100.3 |
| Standard3 | C1 | 25 | 0.569 | 24.87 | 99.5 |
| Standard4 | D1 | 12.5 | 0.247 | 11.65 | 93.19 |
| Standard5 | E1 | 6.25 | 0.19 | 8.622 | 137.9 |
| Standard6 | F1 | 3.125 | 0.136 | 4.995 | 159.8 |
| Standard7 | G1 | 1.563 | 0.065 | <  Curve | - |

| **Sample** | **Wells** | **Raw** | **Background Corrected** | **Conc.** | **Conc. (Average)** | **%CV** | **SD** | **SEM** |
| --- | --- | --- | --- | --- | --- | --- | --- | --- |
| Control1 | A3 | 0.142 | 0.131 | 4.133 | 4.528 | 22.7 | 1.03 | 0.594 |
|  | G2 | 0.138 |  | 3.755 |  |  |  |  |
|  | H2 | 0.161 |  | 5.695 |  |  |  |  |
| S8/MCF7 | A2 | 0.654 | 0.718 | 27.43 | 30.39 | 8.44 | 2.56 | 1.48 |
|  | B2 | 0.772 |  | 31.78 |  |  |  |  |
|  | C2 | 0.777 |  | 31.97 |  |  |  |  |
|  |  |  |  |  |  |  |  |  |
| Blank | H1 | 0.016 | 0 | <  Curve | - | - | - | - |

Researcher

Assay Samples cell line Ref.

Date Reader Kit used Solvent

Assay samples

: Dr.Feby Nabil

email: [Fibytakla@gmail.com](mailto:Fibytakla@gmail.com) mob. 01224037280

: Caspase 9 enzyme assay

: 02 samples

: ---

: ---

: 18-01-2022

: ROBONIK P2000 Eliza Reader wl 450 nm

: Invitrogen EIA kit Human caspase9 (active) KHO1091 (96 tests)

: DMSO

: Cell culture supernatant

# Lab Report

| **Ser** | **Compound** | | | | **Casp9** | **FLD** |
| --- | --- | --- | --- | --- | --- | --- |
|  | **code** | **mw** | **Cells** | **IC50**  **ug/ml** | **ng/ml** |  |
| **1** | **s10/MDA** |  |  |  | **16.77±0.37** | **6.42** |
| **2** | **s12/MDA** |  |  |  | **19.16±0.41** | **7.34** |
| **3** | **cont.MDA** |  |  |  | **2.611±0.19** | **1** |

**Casp9**

**25.00**

**20.00**

**15.00**

**ng/ml**

**10.00**

**5.00**

**0.00**

**S**

Detailed results

**Casp9**

4PL Standard

| **STANDARDS**  **Ca9** | Conc. ng/ml |
| --- | --- |
| St.1 | 100 |
| St.2 | 50 |
| St.3 | 25 |
| St.4 | 12.5 |
| St.5 | 6.3 |
| St.6 | 3.1 |
| St.7 | 1.6 |

Plate map

2.5

2

**Measurement**

1.5

1

0.5

0

1

10

**Concentration**

100

|  | 1 | 2 | 3 | 4 | 5 | 6 | 7 | 8 | 9 | 10 | 11 | 12 |
| --- | --- | --- | --- | --- | --- | --- | --- | --- | --- | --- | --- | --- |
| A | st1 | s10 | - | - | - | - | - | - | - | - | - |  |
| B | st2 | s10 | - | - | - | - | - | - | - | - | - |  |
| C | st3 | s12 | - | - | - | - | - | - | - | - | - |  |
| D | st4 | s12 | - | - | - | - | - | - | - | - | - |  |
| E | st5 | cont | - | - | - | - | - | - | - | - | - | - |
| F | st6 | cont | - | - | - | - | - | - | - | - | - | - |
| G | st7 | -- | - | - | - | - | - | - | - | - | - | - |
| H | B | -- | - | - | - | - | - | - | - | - | - | - |

ODs

|  | 1 | 2 | 3 | 4 | 5 | 6 | 7 | 8 | 9 | 10 | 11 | 12 |
| --- | --- | --- | --- | --- | --- | --- | --- | --- | --- | --- | --- | --- |
| A | 2.143 | 0.431 | 0 | 0 | 0 | 0 | 0 | 0 | 0 | 0 | 0 | 0 |
| B | 1.401 | 0.407 | 0 | 0 | 0 | 0 | 0 | 0 | 0 | 0 | 0 | 0 |
| C | 0.613 | 0.518 | 0 | 0 | 0 | 0 | 0 | 0 | 0 | 0 | 0 | 0 |
| D | 0.288 | 0.522 | 0 | 0 | 0 | 0 | 0 | 0 | 0 | 0 | 0 | 0 |
| E | 0.217 | 0.161 | 0 | 0 | 0 | 0 | 0 | 0 | 0 | 0 | 0 | 0 |
| F | 0.165 | 0.149 | 0 | 0 | 0 | 0 | 0 | 0 | 0 | 0 | 0 | 0 |
| G | 0.096 | 0 | 0 | 0 | 0 | 0 | 0 | 0 | 0 | 0 | 0 | 0 |
| H | 0.012 | 0 | 0 | 0 | 0 | 0 | 0 | 0 | 0 | 0 | 0 | 0 |

| **Calibrator** | **Wells** | **Conc.** | **Raw (Corrected)** | **Backfit** | **Recovery**  **%** |
| --- | --- | --- | --- | --- | --- |
| Standard1 | A1 | 100 | 2.13 | 99.69 | 99.69 |
| Standard2 | B1 | 50 | 1.39 | 50.34 | 100.7 |
| Standard3 | C1 | 25 | 0.601 | 24.46 | 97.84 |
| Standard4 | D1 | 12.5 | 0.276 | 12.83 | 102.6 |
| Standard5 | E1 | 6.25 | 0.205 | 9.115 | 145.8 |
| Standard6 | F1 | 3.125 | 0.153 | 4.996 | 159.9 |
| Standard7 | G1 | 1.563 | 0.084 | <  Curve | - |

| **Sample** | **Wells** | **Raw** | **Background Corrected** | **Conc.** | **Conc. (Average)** | **%CV** | **SD** | **SEM** |
| --- | --- | --- | --- | --- | --- | --- | --- | --- |
| Control | E2  F2 | 0.161  0.149 | 0.143 | 4.527  3.64 | 4.084 | 37.2 | 1.33 | 0.943 |
| s10 | A2  B2 | 0.431  0.407 | 0.407 | 18.48  17.62 | 18.05 | 3.37 | 0.608 | 0.43 |
| s12 | C2  D2 | 0.518  0.522 | 0.508 | 21.43  21.56 | 21.49 | 0.431 | 0.0925 | 0.0654 |
| Blank | H1 | 0.012 | 0 | <  Curve | - | - | - | - |
