# Supplementary material for: Developing multitarget coumarin based anti-breast cancer agents: synthesis and molecular modeling study
Source: Sci Rep. 2023 Aug 17;13:13370. doi: 10.1038/s41598-023-40232-3 (PMC10435442; doi:10.1038/s41598-023-40232-3)

Developing Multitarget Coumarin Based Anti-Breast Cancer Agents: Synthesis and Molecular Modeling Study

Fiby N. Takla,[1] Waleed A. Bayoumi,[2] Shahenda M. El-Messery,[2*] Magda N. A. Nasr [2]

[1] *Department of Pharmaceutical Chemistry, Faculty of Pharmacy, Delta University for Science and Technology, International Coastal Road, Gamasa City, 35712, Egypt.*

[2]*Department of Pharmaceutical Organic Chemistry, Faculty of Pharmacy, Mansoura University, Mansoura 35516, Egypt.*

**Corresponding author:* Prof. Shahenda M. El-Messery Department of Pharmaceutical Organic Chemistry, Faculty of Pharmacy, Mansoura University, P.O.Box 35516, Mansoura, Egypt.

E- mail , habib2001@mans.edu.eg Tel, Fax: +20-50-2200242

Supplementary file (chemistry)

Compound 2

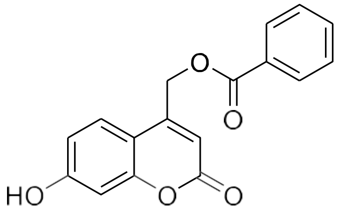

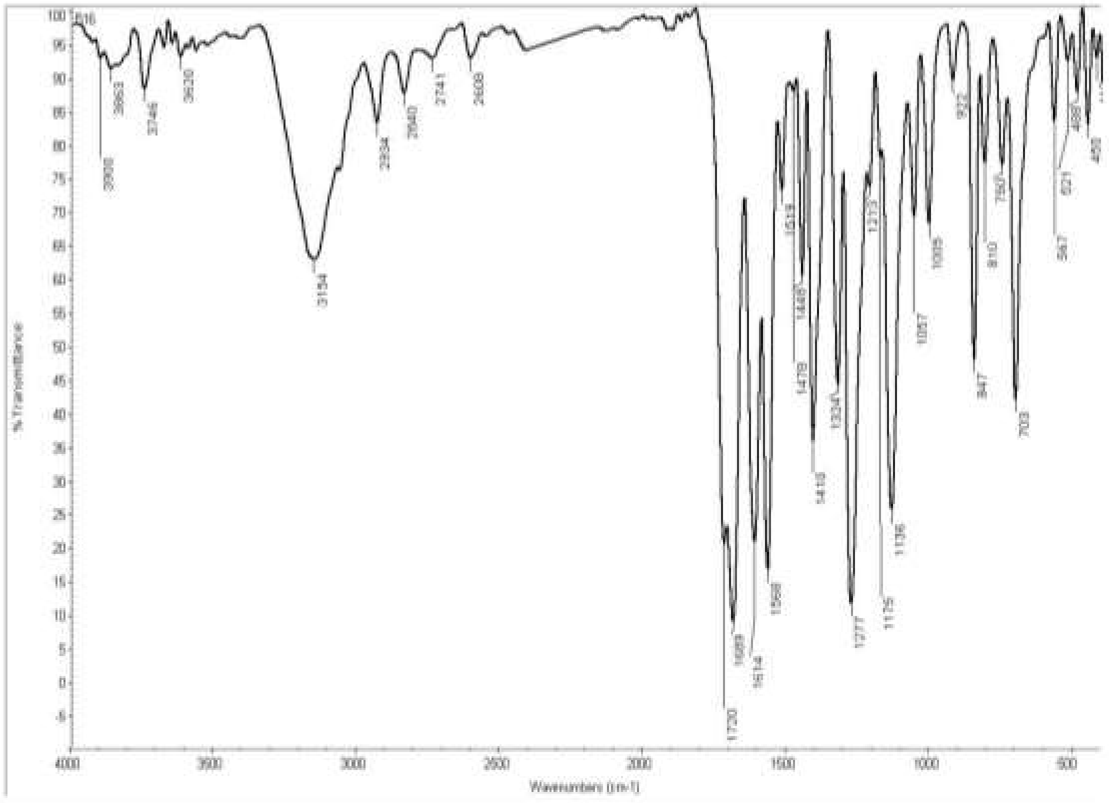

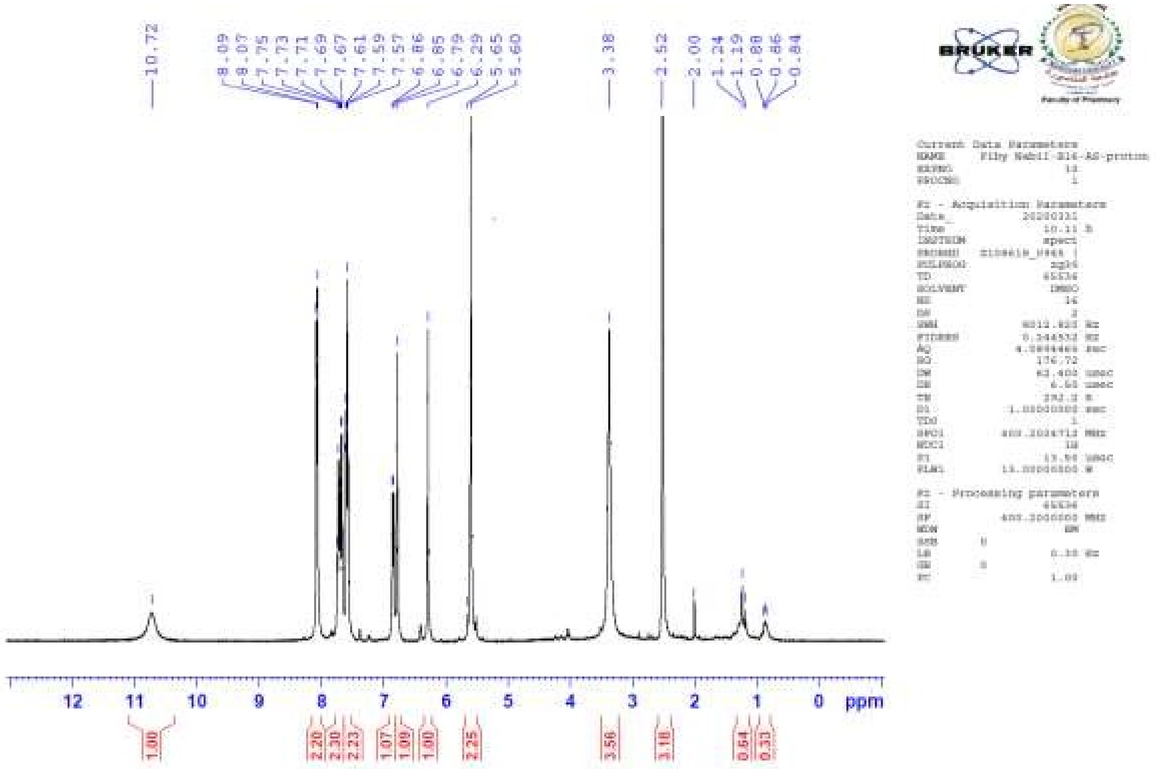
10.72
8.09
8.07
7.75
7.73
7.71
7.69
7.67
7.61
7.59
7.57
6.86
6.85
6.79
6.29
5.65
5.60
3.38
2.52
2.00
1.24
1.19
0.88
0.86
0.84
12 11 10 9 8 7 6 5 4 3 2 1 0 ppm
1.00
2.20
2.30
2.23
1.07
1.09
1.00
2.25
3.56
3.18
0.64
0.33
BRUKER
Current Data Parameters
Faculty of Pharmacy

Fiby Nabil-B16-AS-proton

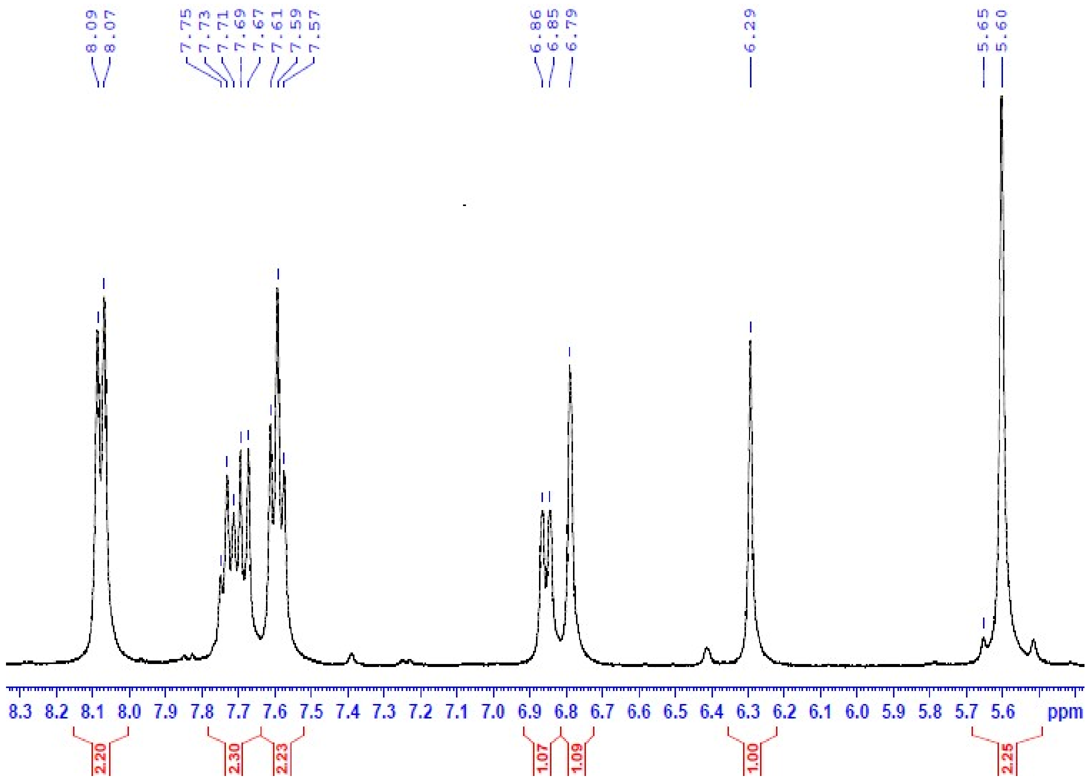

Fiby Nabil-B16-AS-proton

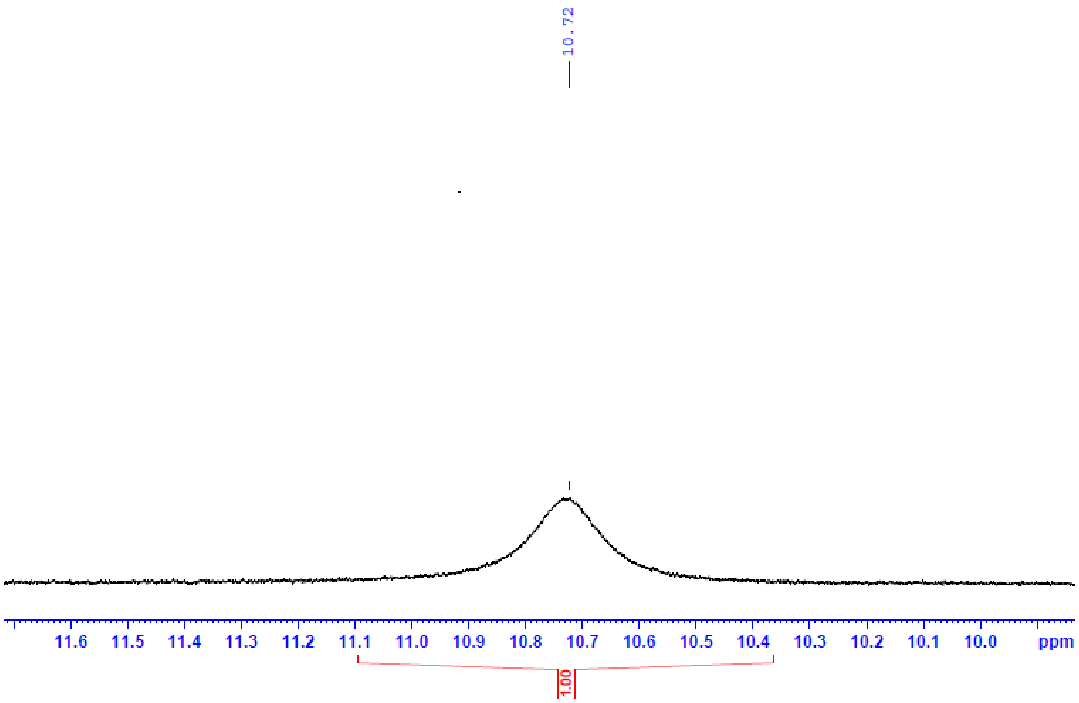

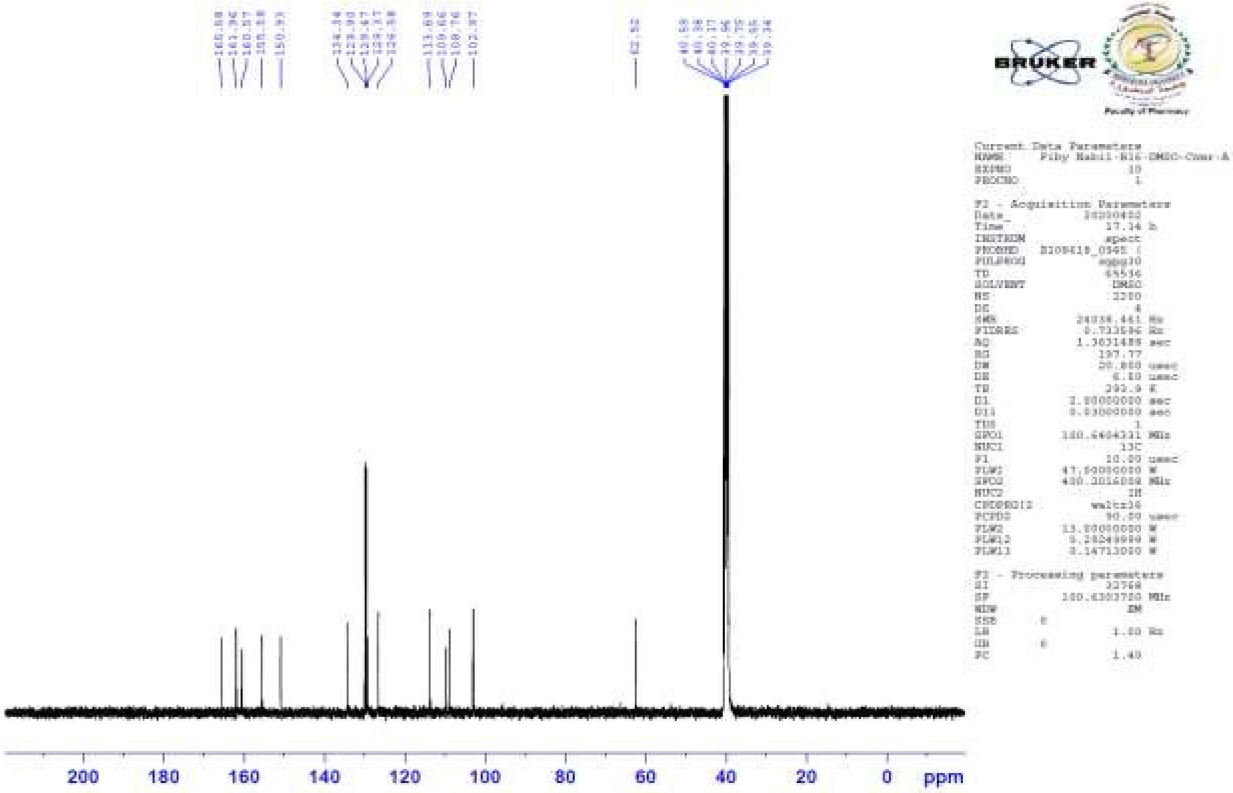
165.58
161.96
160.57
155.58
150.93
134.34
129.90
129.47
129.37
126.58
113.69
109.66
108.76
102.97
62.52
40.58
40.38
40.17
39.96
39.75
39.55
39.34
BRUKER
Current Data Parameters
F2 - Acquisition Parameters
F2 - Processing parameters
200
180
160
140
120
100
80
60
40
20
0
ppm

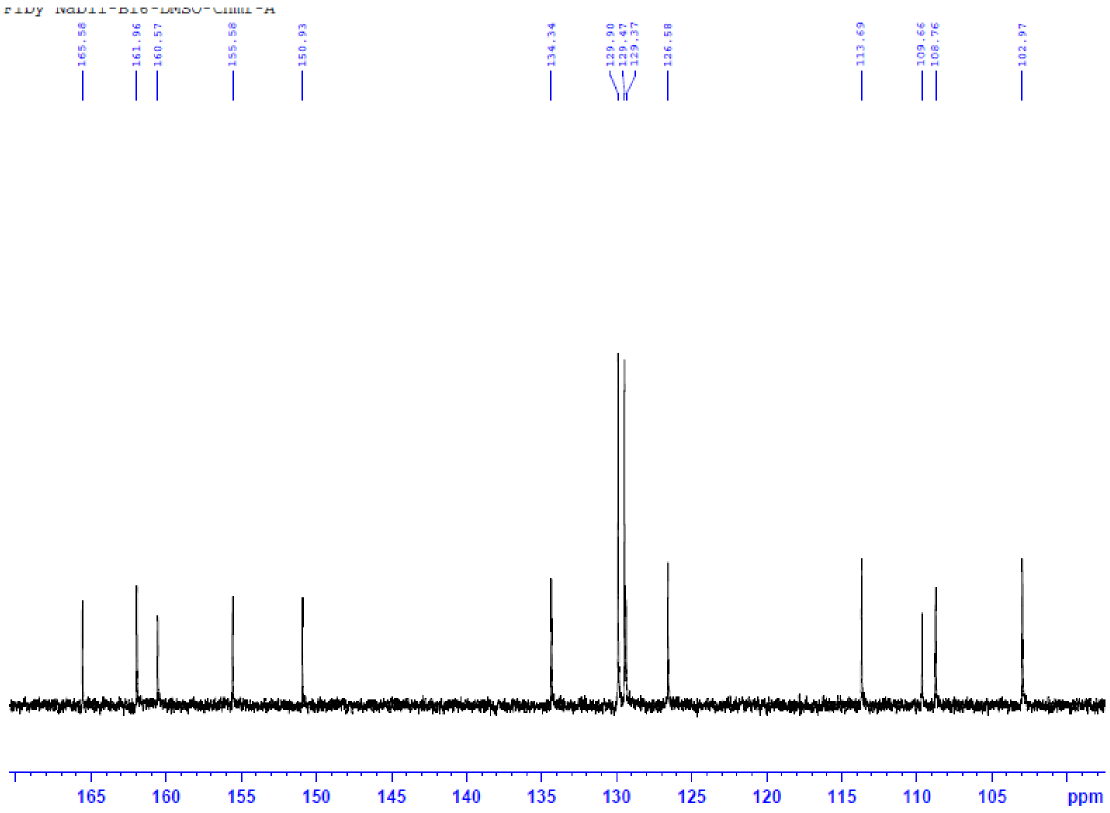
165.58
161.96
160.57
155.58
150.93
134.34
129.90
129.47
129.37
126.58
113.69
109.66
108.76
102.97
165
160
155
150
145
140
135
130
125
120
115
110
105
ppm

RT: 0.00 - 5.47 SM: 15G

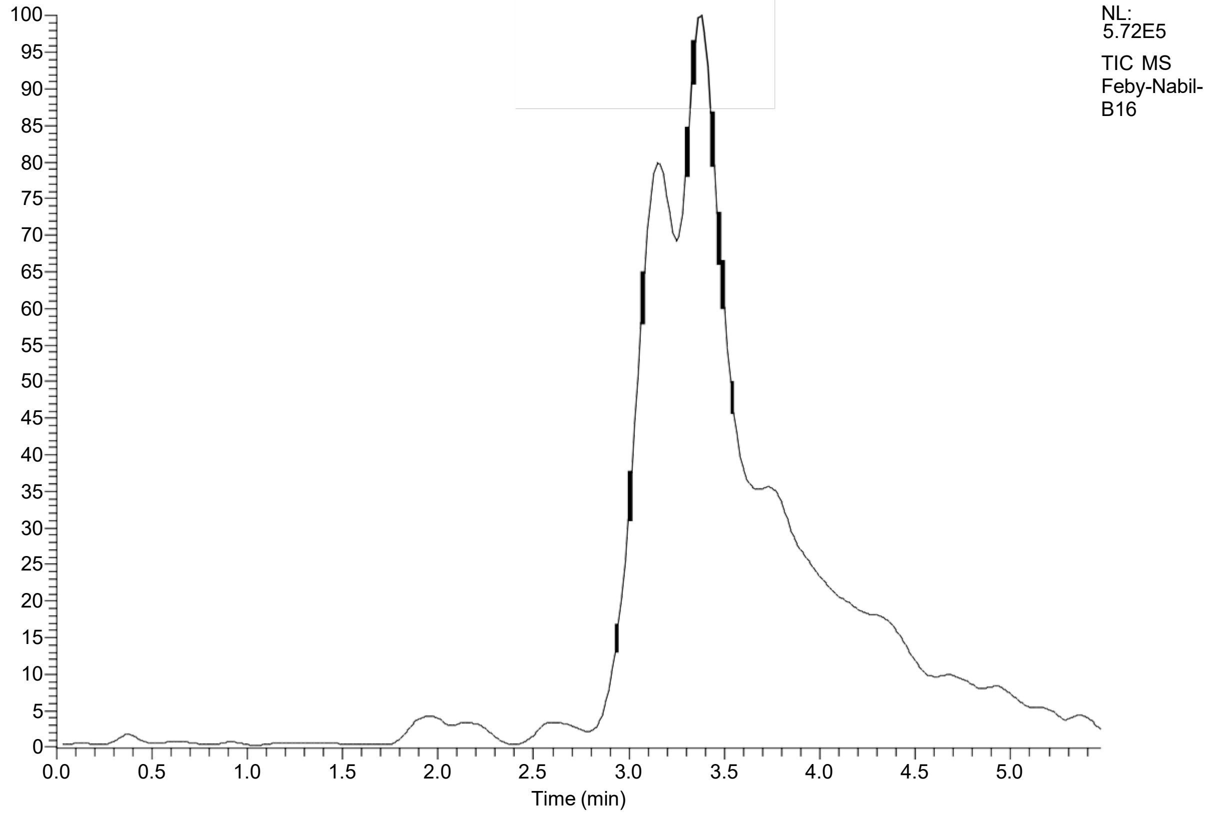

Feby-Nabil-B16 #214 RT: 3.60 AV: 1 SB: 2 4.45 , 4.45 NL: 2.72E4
T: {0,0} + c EI Full ms [40.00-1000.00]

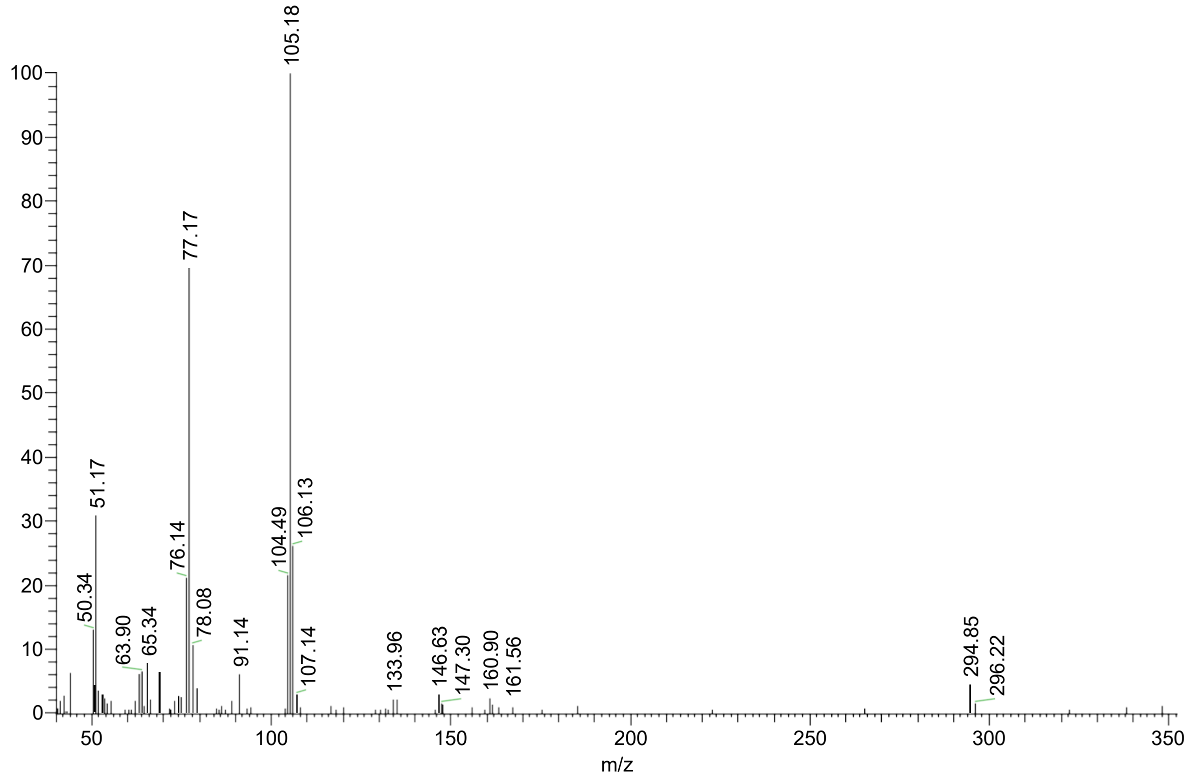

Compound 3

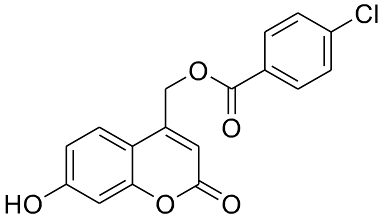

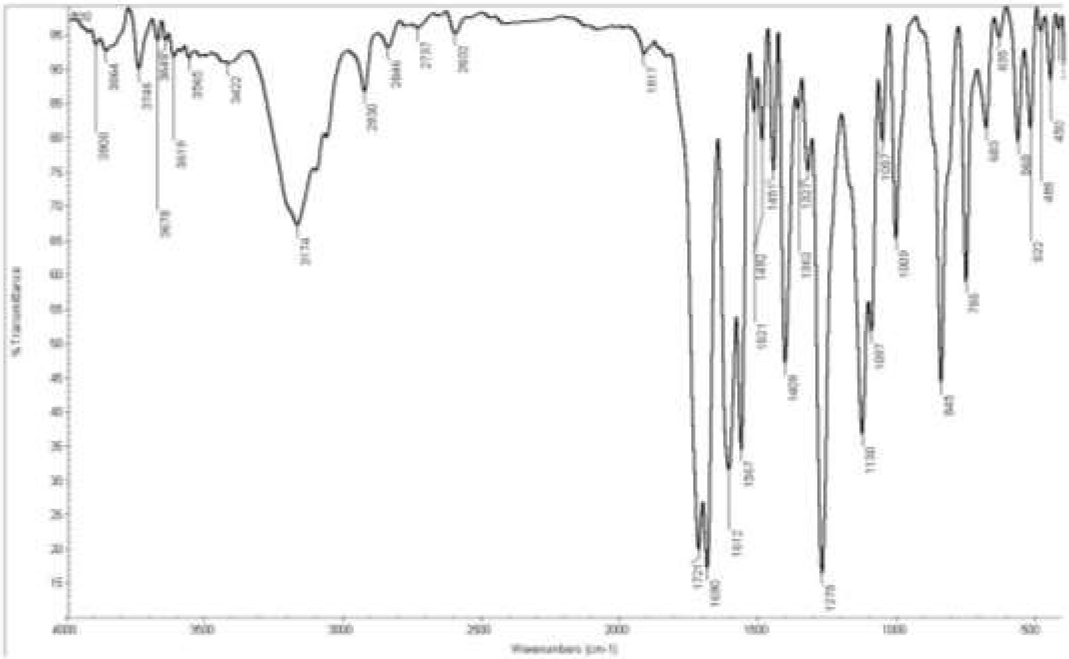

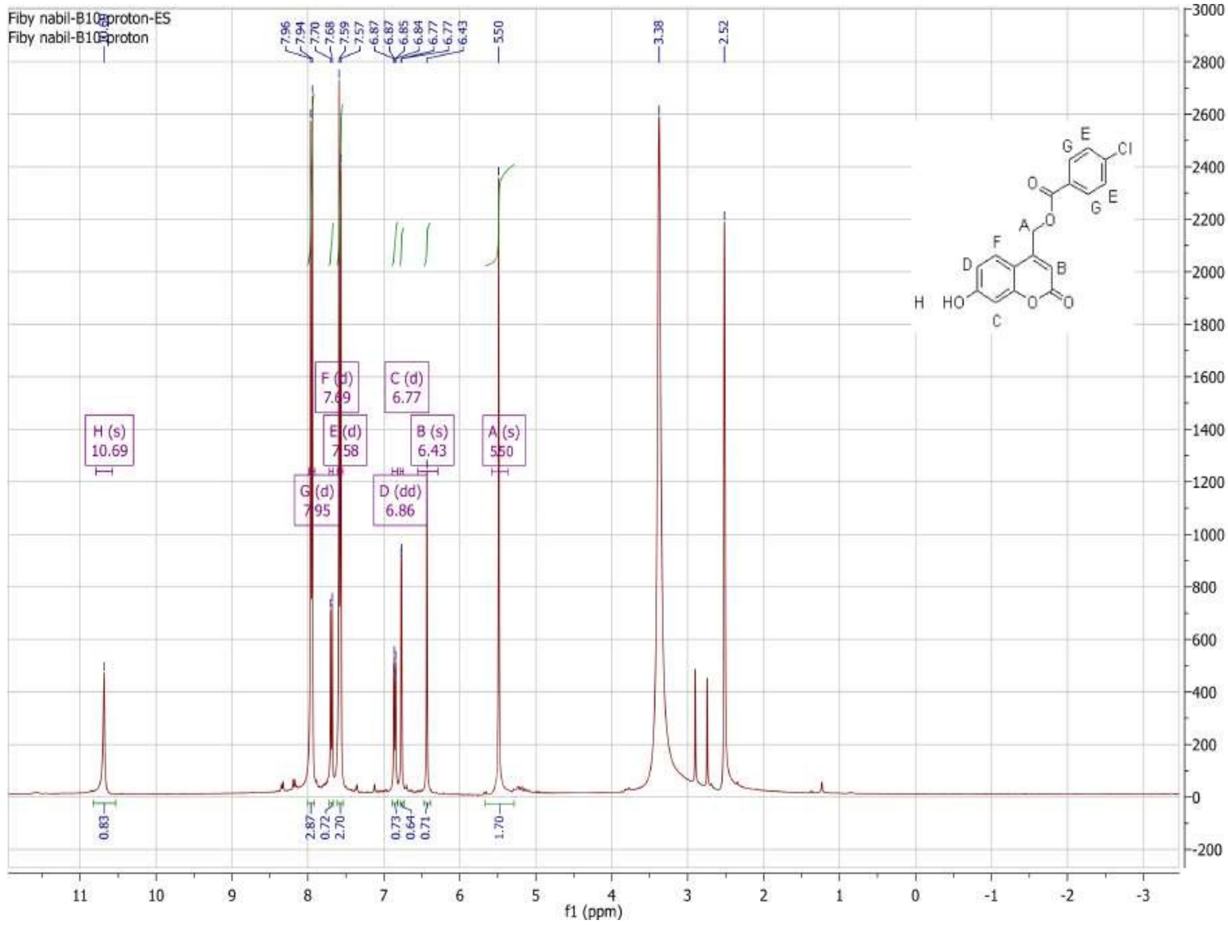

Fiby nabil-B10-proton-ES
Fiby nabil-B10-proton
f1 (ppm)

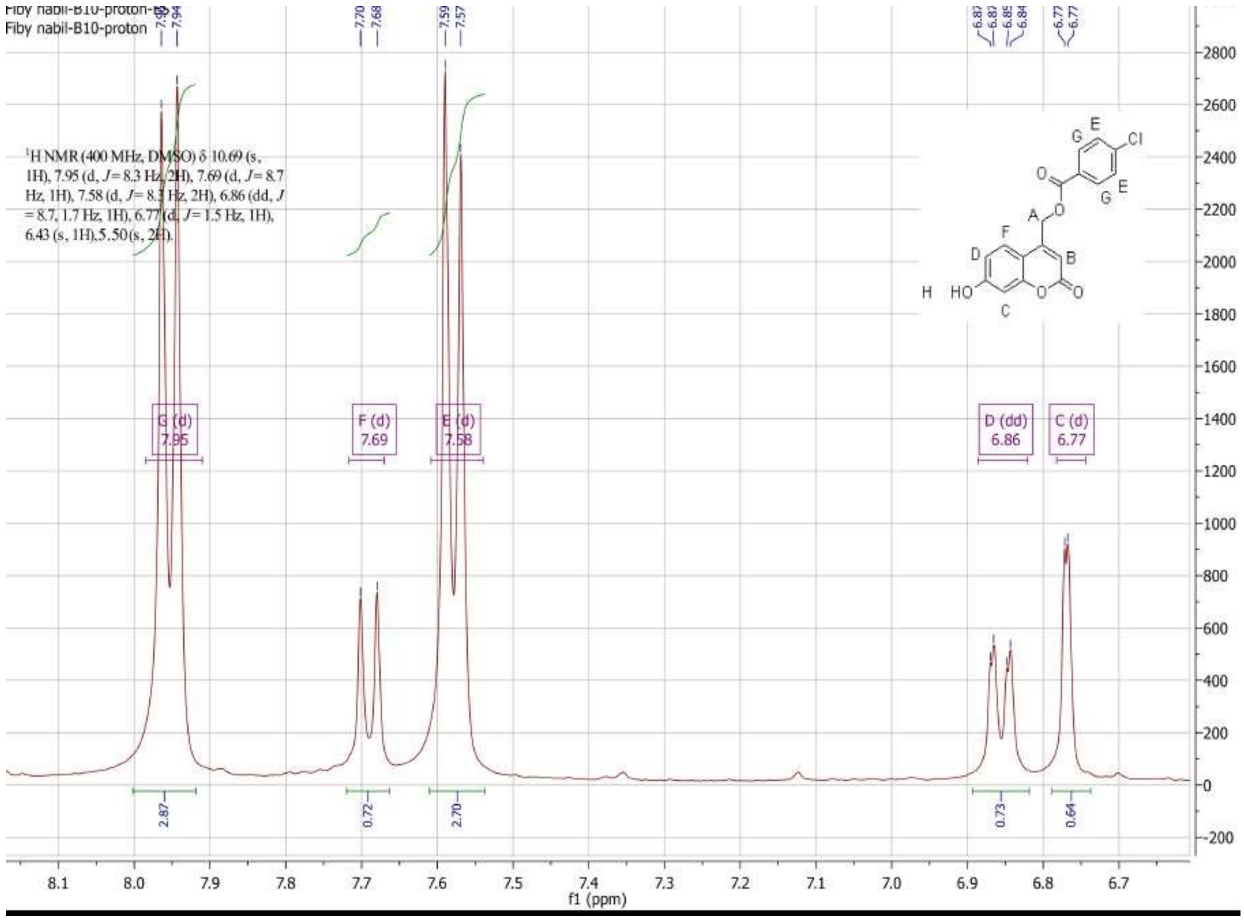

Fiby nabil-B10-proton-ES
Fiby nabil-B10-proton
^{1}H NMR (400 MHz, DMSO) δ 10.69 (s, 1H), 7.95 (d, J = 8.3 Hz, 2H), 7.69 (d, J = 8.7 Hz, 1H), 7.58 (d, J = 8.3 Hz, 2H), 6.86 (dd, J = 8.7, 1.7 Hz, 1H), 6.77 (d, J = 1.5 Hz, 1H), 6.43 (s, 1H),5.50 (s, 2H).
f1 (ppm)

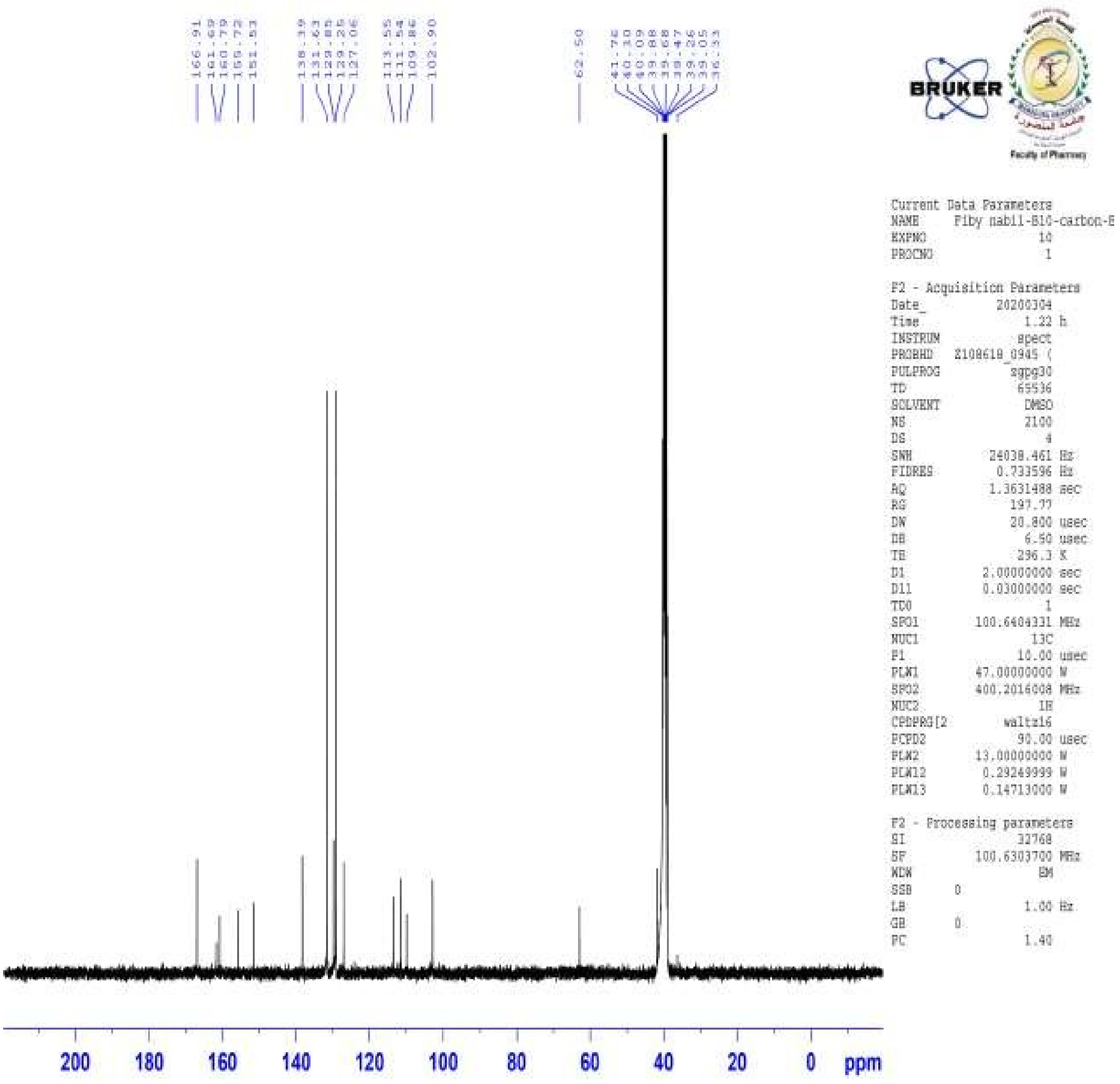

166.91
161.69
160.79
155.72
151.53
138.39
131.63
129.85
129.26
127.06
113.55
111.54
109.86
102.90
62.50
41.76
40.30
40.09
39.88
39.68
39.47
39.26
39.05
36.33
BRUKER
Faculty of Pharmacy
Current Data Parameters
NAME Fiby nabil-B10-carbon-E
EXPNO 10
PROCNO 1
F2 - Acquisition Parameters
Date_ 20200304
Time 1.22 h
INSTRUM spect
PROBHD Z108618_0945 (
PULPROG zgpg30
TD 65536
SOLVENT DMSO
NS 2100
DS 4
SWH 24038.461 Hz
FIDRES 0.733596 Hz
AQ 1.3631488 sec
RG 197.77
DW 20.800 usec
DE 6.50 usec
TE 296.3 K
D1 2.00000000 sec
D11 0.03000000 sec
TD0 1
SFO1 100.6404331 MHz
NUC1 13C
P1 10.00 usec
PLW1 47.00000000 W
SFO2 400.2016008 MHz
NUC2 1H
CPDPRG[2 waltz16
PCPD2 90.00 usec
PLW2 13.00000000 W
PLW12 0.29249999 W
PLW13 0.14713000 W
F2 - Processing parameters
SI 32768
SF 100.6303700 MHz
WDW EM
SSB 0
LB 1.00 Hz
GB 0
PC 1.40
200
180
160
140
120
100
80
60
40
20
0
ppm

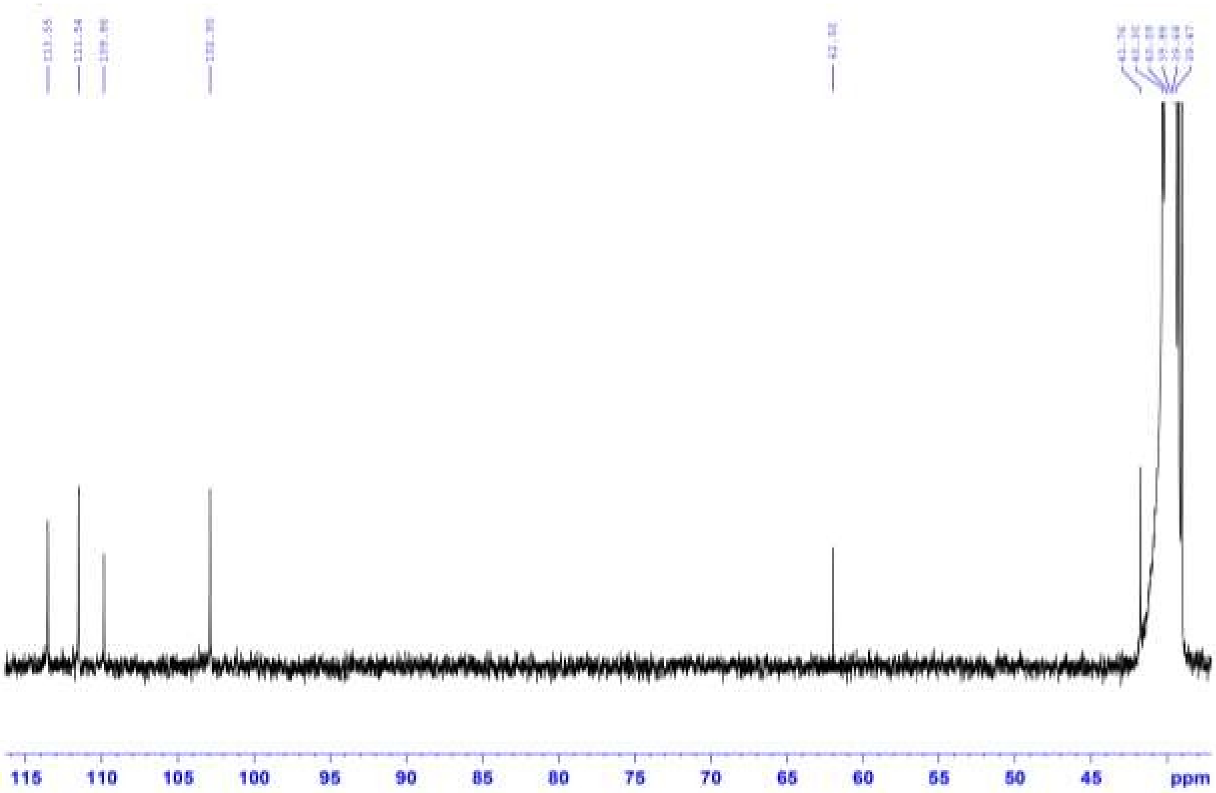

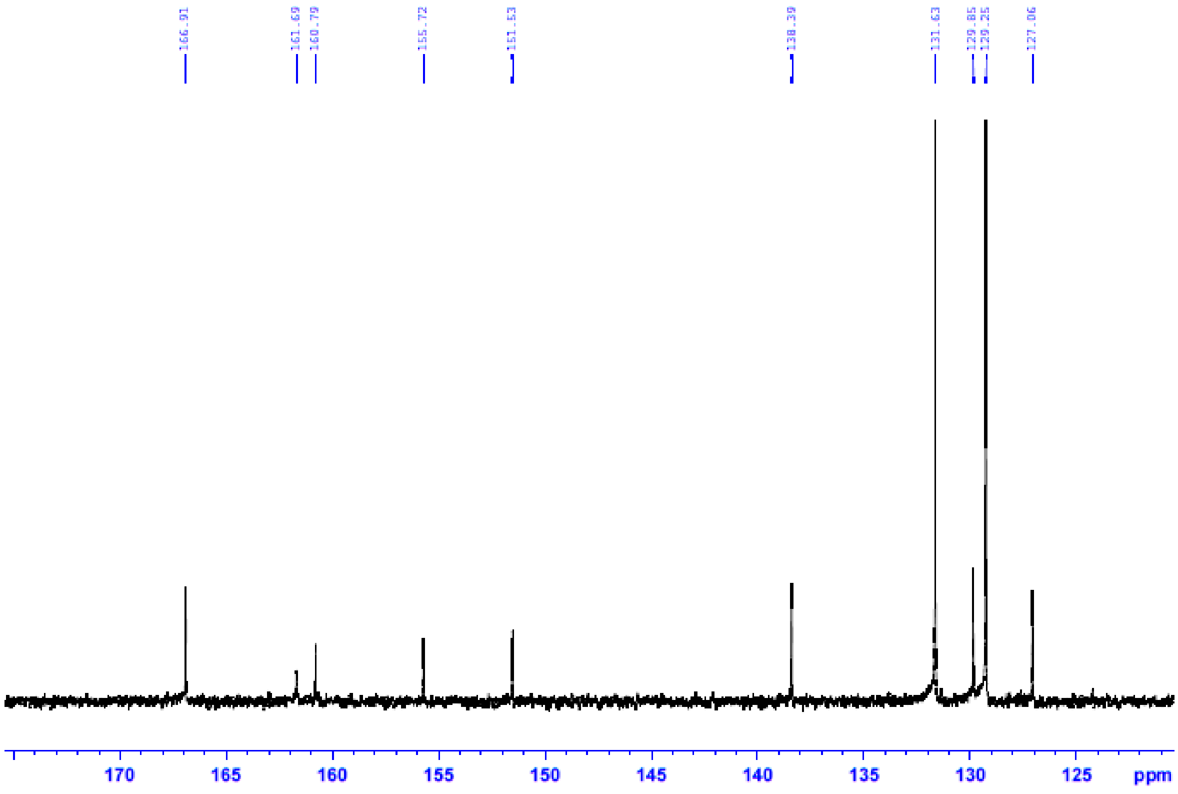
166.91
161.69
160.79
155.72
151.53
138.39
131.63
129.85
129.25
127.06
170
165
160
155
150
145
140
135
130
125
ppm

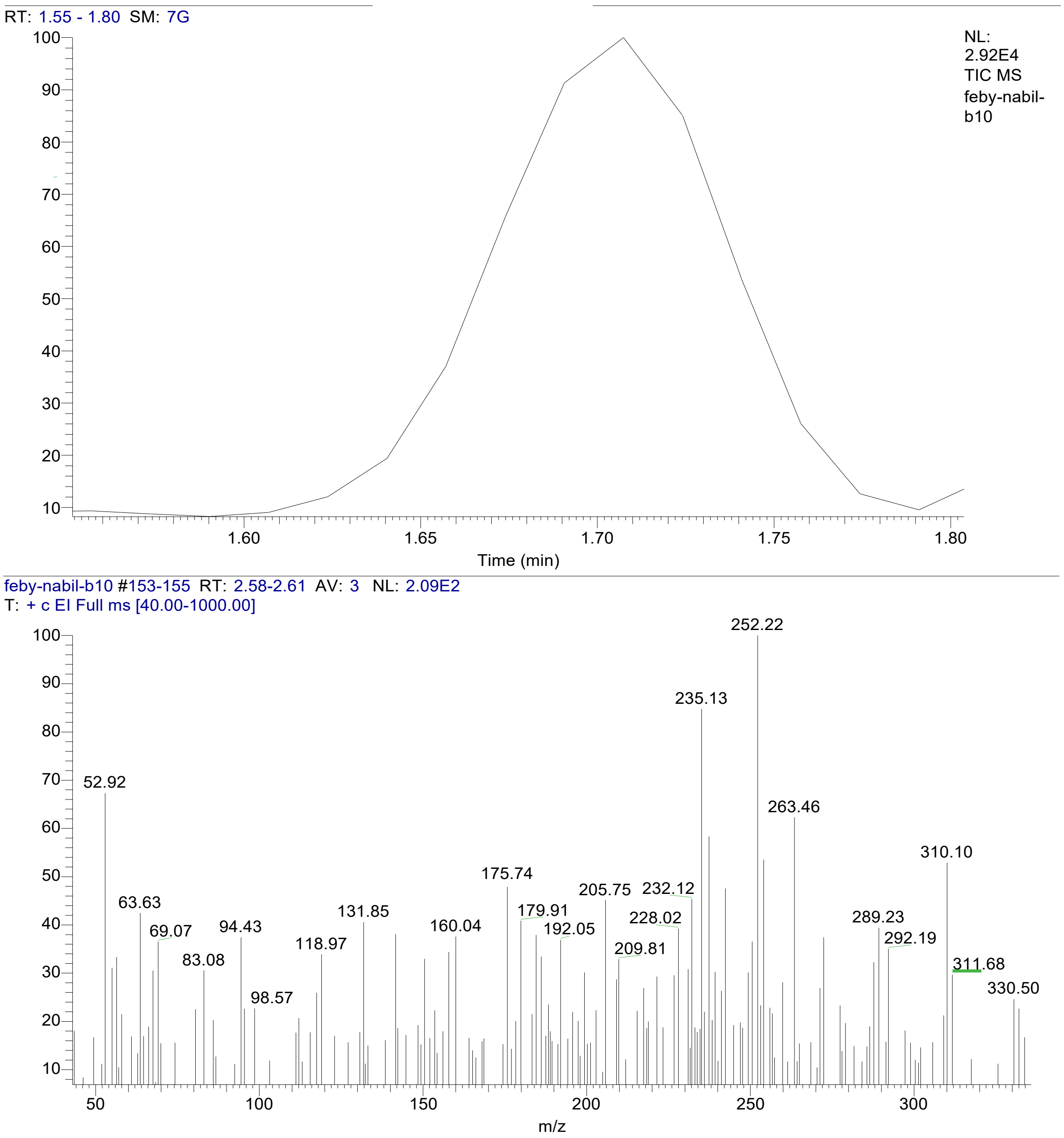
RT: 1.55 - 1.80 SM: 7G
NL:
2.92E4
TIC MS
feby-nabil-
b10
Time (min)
feby-nabil-b10 #153-155 RT: 2.58-2.61 AV: 3 NL: 2.09E2
T: + c EI Full ms [40.00-1000.00]
252.22
235.13
52.92
263.46
310.10
175.74
205.75
232.12
63.63
131.85
179.91
289.23
69.07
94.43
160.04
192.05
228.02
292.19
118.97
209.81
83.08
311.68
98.57
330.50
m/z

Compound 4

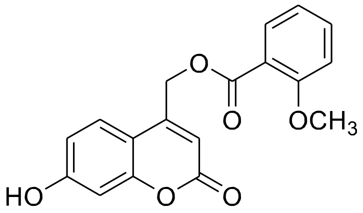

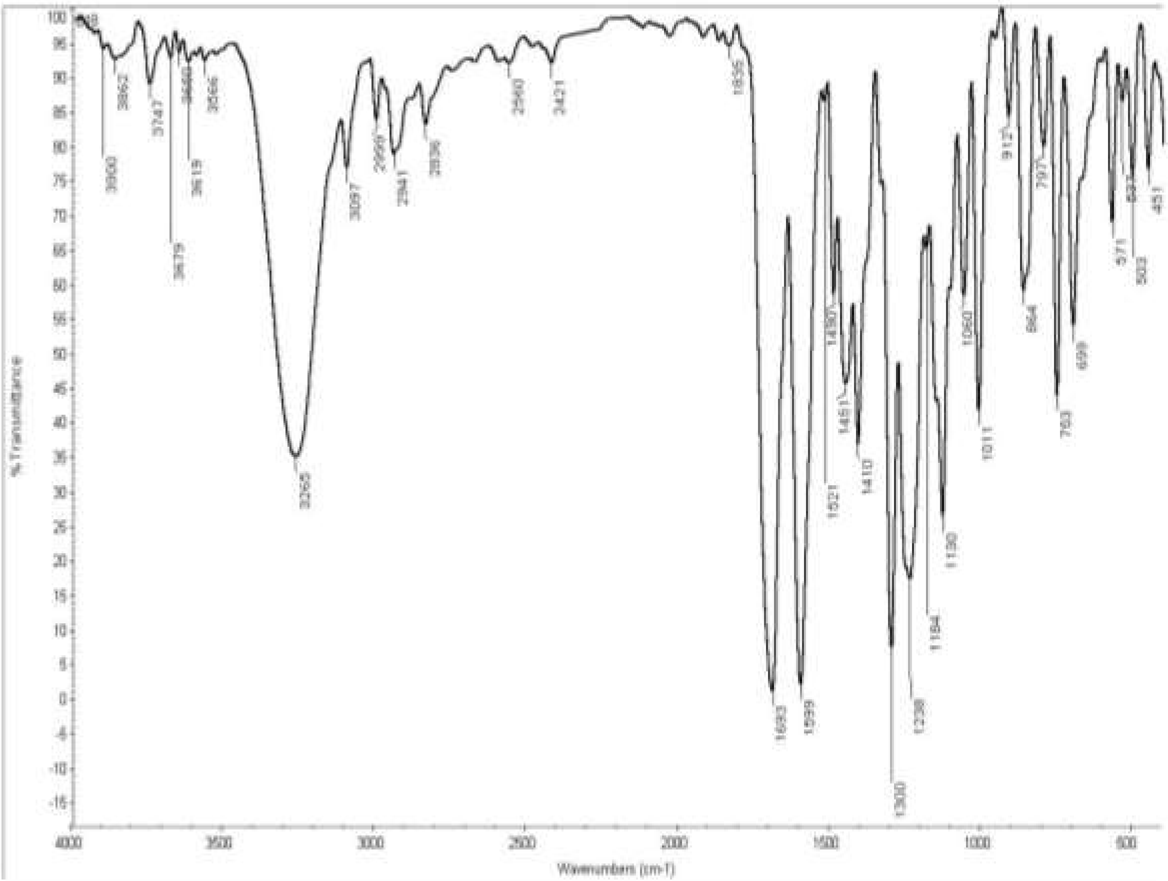
% Transmittance
Wavenumbers (cm-1)
3268
1693
1599
1300
1238
1184
1130
1011
763

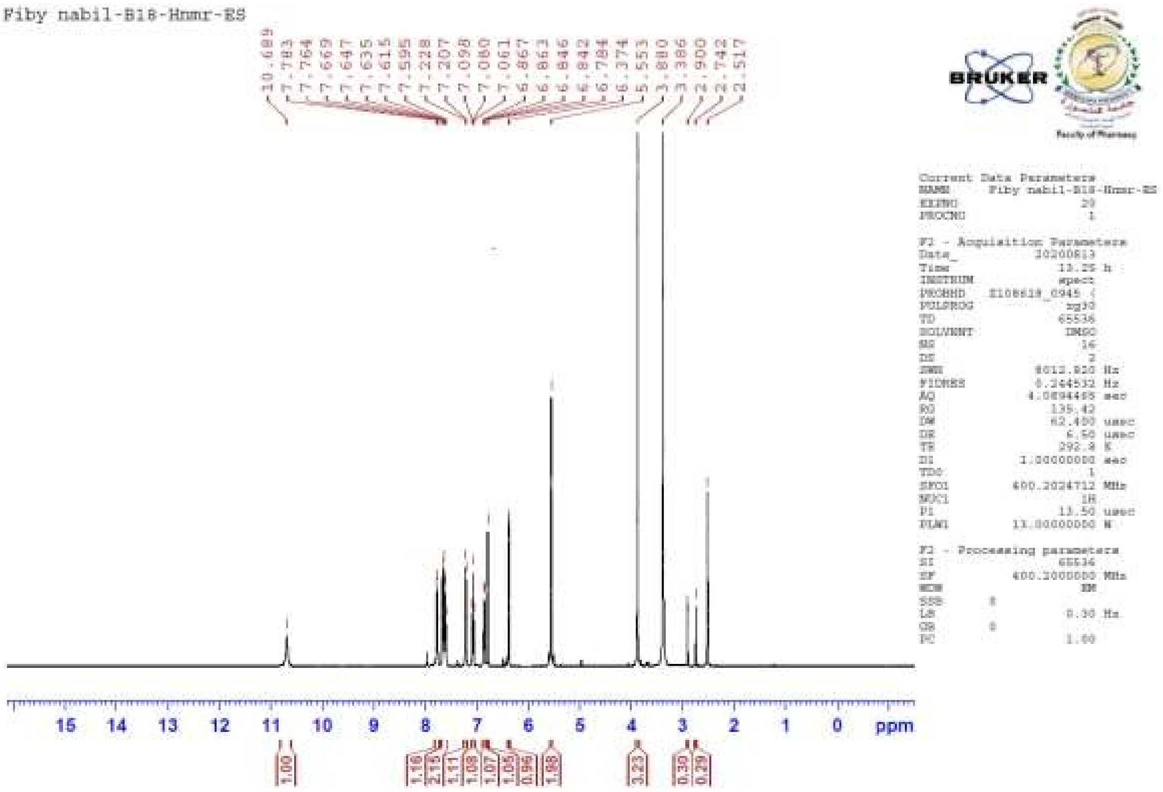
Fiby nabil-B18-Hnmr-ES
10.689
7.783
7.764
7.669
7.647
7.635
7.615
7.595
7.228
7.207
7.098
7.080
7.061
6.867
6.863
6.846
6.842
6.784
6.374
5.553
3.880
3.386
2.900
2.742
2.517
BRUKER
ppm
1.00
1.10
2.15
1.11
1.08
1.07
1.05
0.96
1.98
3.23
0.30
0.29

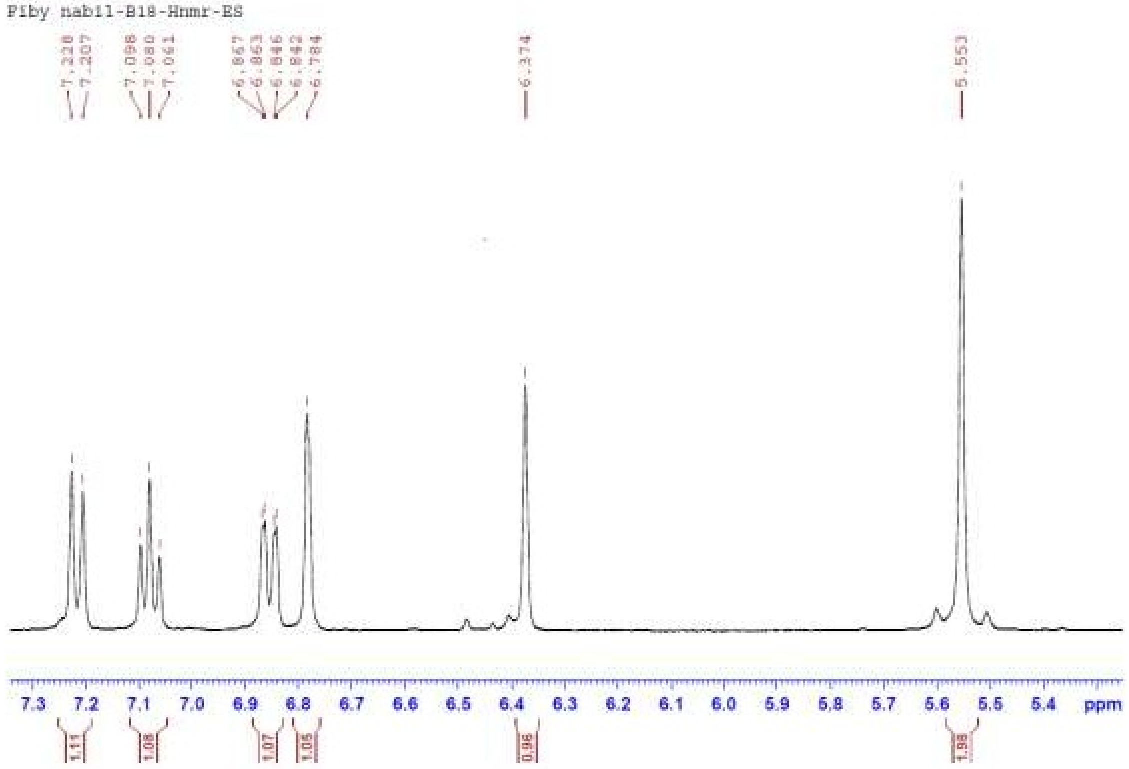
Fiby nabil-B18-Hnmr-ES
7.228
7.207
7.098
7.080
7.061
6.867
6.863
6.846
6.842
6.784
6.374
5.553
ppm
1.11
1.08
1.07
1.05
0.96
1.98

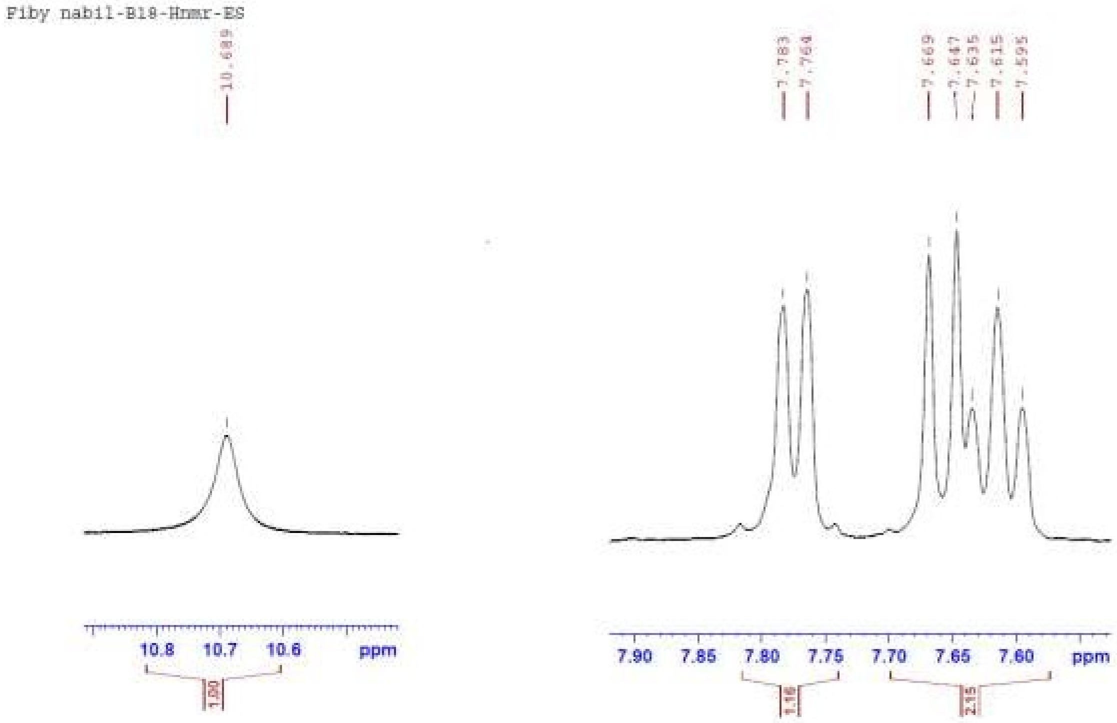
Fiby nabil-B18-Hnmr-ES
10.689
7.783
7.764
7.669
7.647
7.635
7.615
7.595
10.8 10.7 10.6 ppm
1.00
7.90 7.85 7.80 7.75 7.70 7.65 7.60 ppm
1.16
2.15

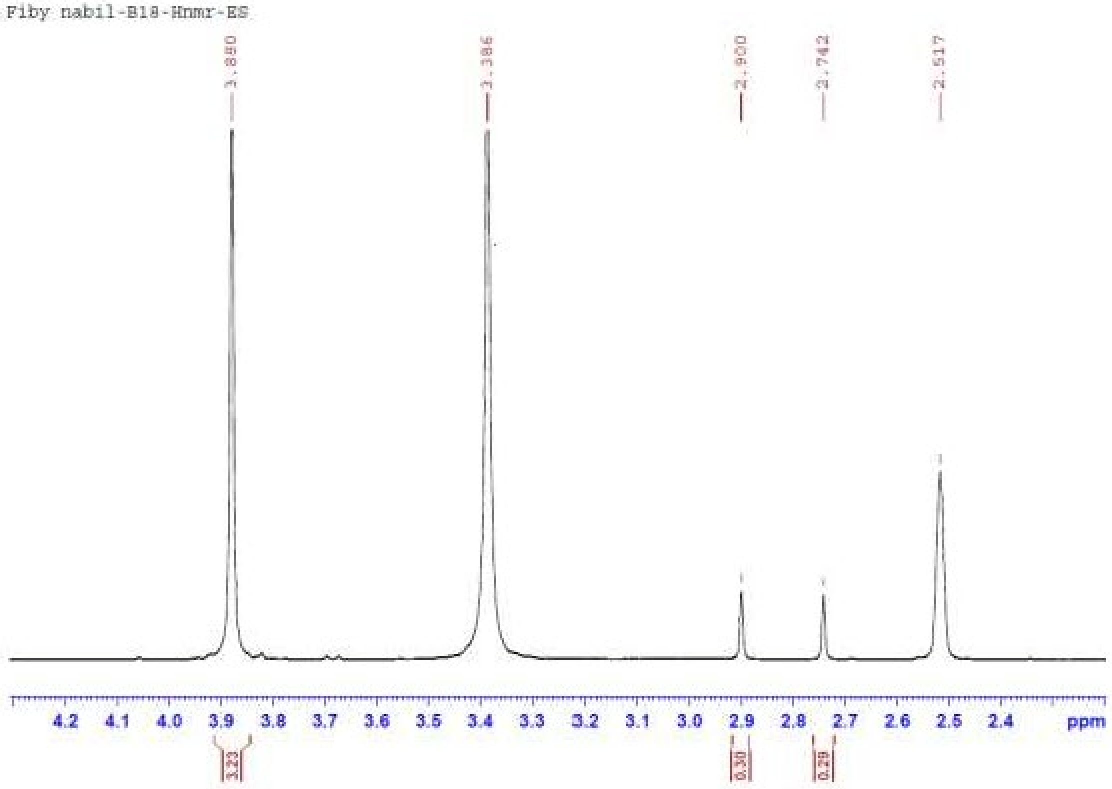
Fiby nabil-B18-Hnmr-ES
3.880
3.386
2.900
2.742
2.517
4.2 4.1 4.0 3.9 3.8 3.7 3.6 3.5 3.4 3.3 3.2 3.1 3.0 2.9 2.8 2.7 2.6 2.5 2.4 ppm
3.23
0.30
0.29

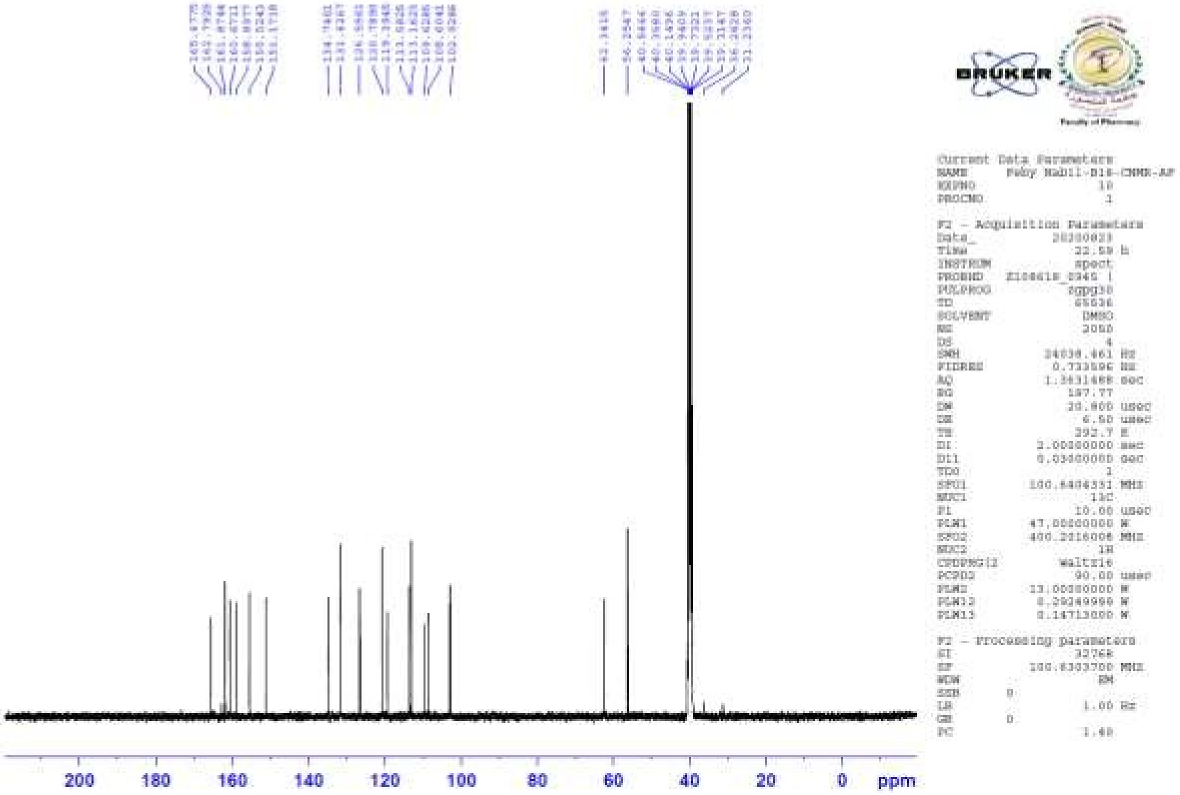
Current Data Parameters
F2 - Acquisition Parameters
SOLVENT DMSO
F2 - Processing parameters
200
180
160
140
120
100
80
60
40
20
0
ppm

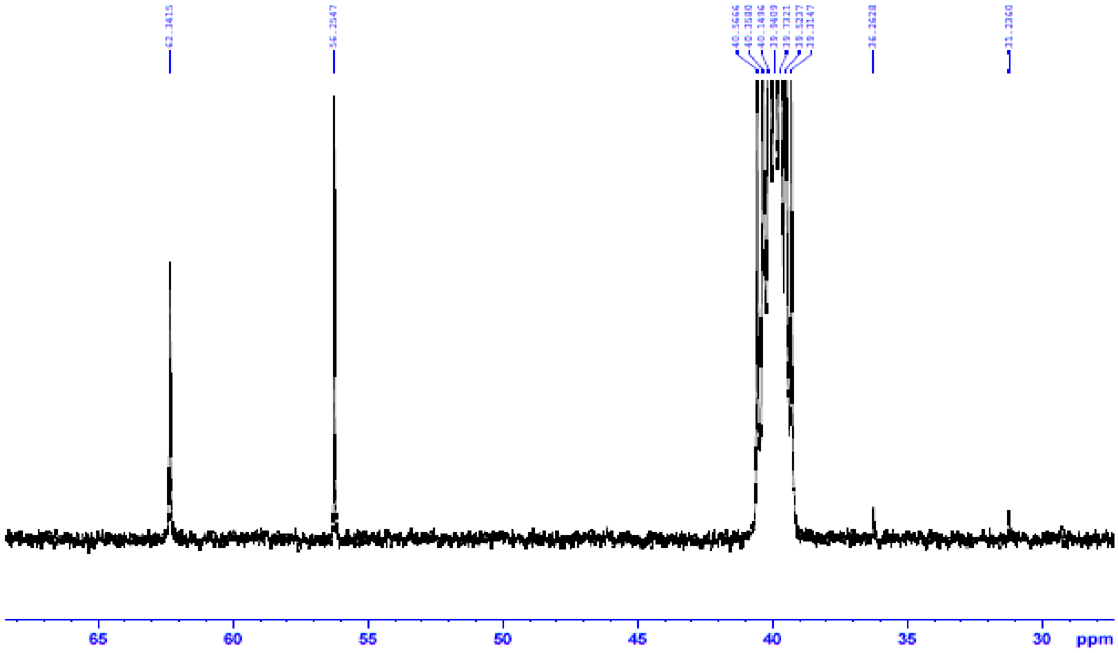
62.3415
56.2547
65
60
55
50
45
40
35
30
ppm

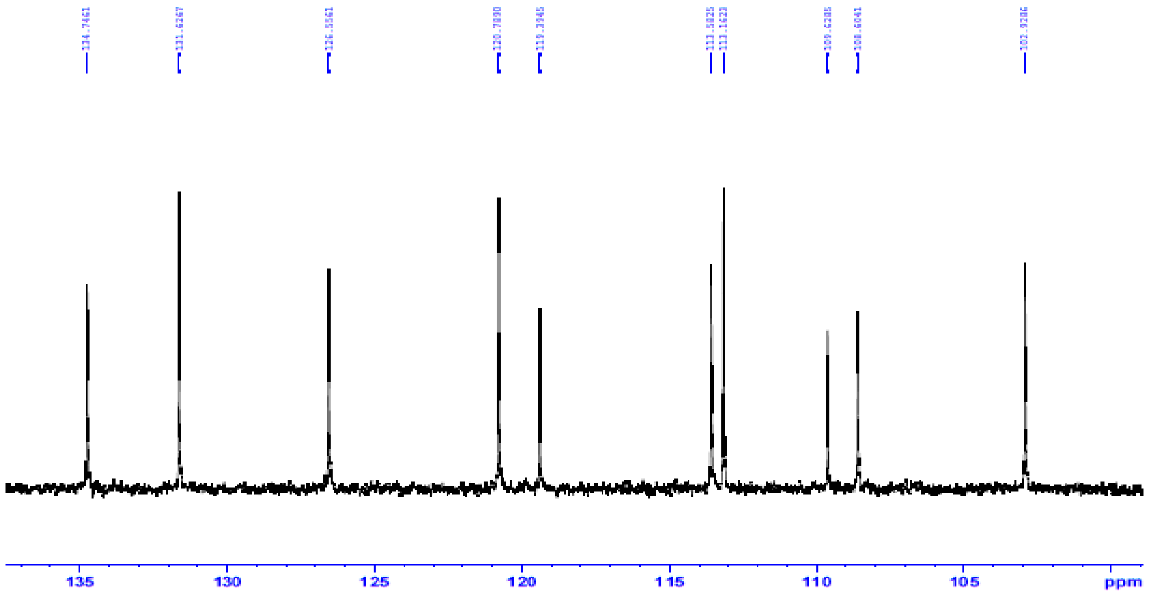

134.7461
131.6267
126.5561
120.7830
119.3945
113.5825
113.1623
109.6285
108.6041
102.9286
135
130
125
120
115
110
105
ppm

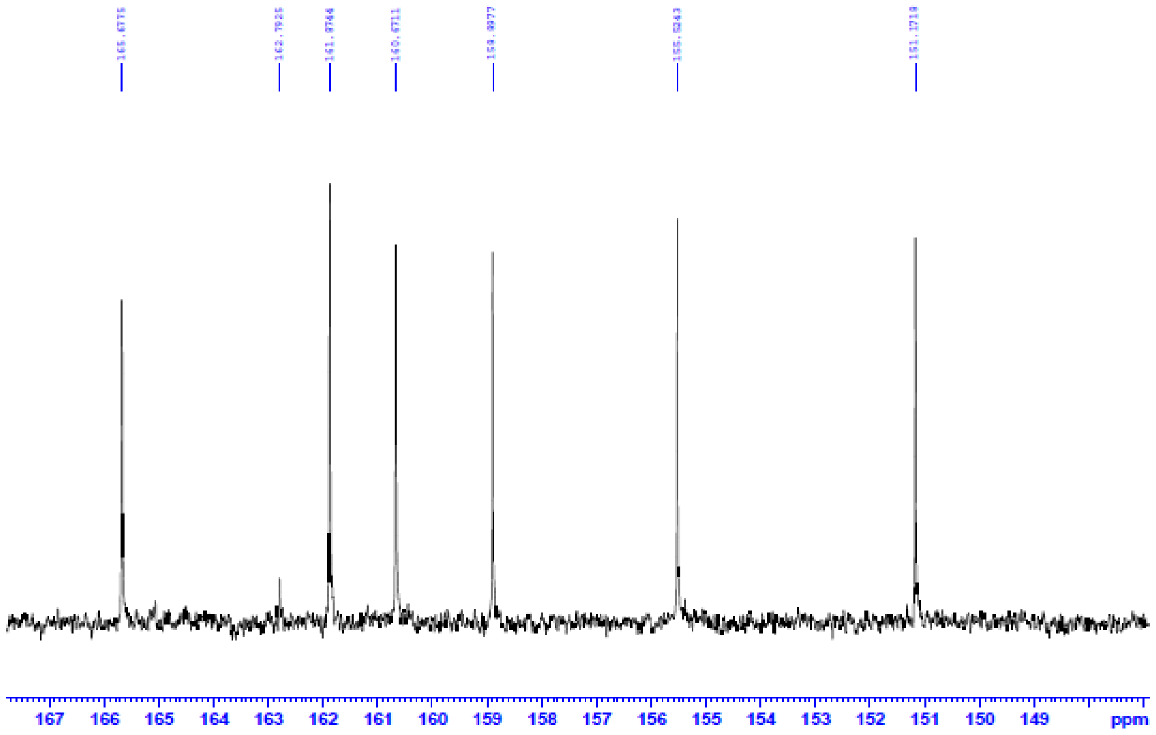

165.6776
162.7925
161.8744
160.6711
158.9977
155.5243
151.1719
167
166
165
164
163
162
161
160
159
158
157
156
155
154
153
152
151
150
149
ppm

C:\Xcalibur\data\S\Feby-Nabil-B18 20-Oct-20 3:03:14 PM
THE [illegible]OGY AND BIOTECHNOLOGY
AZH[illegible]

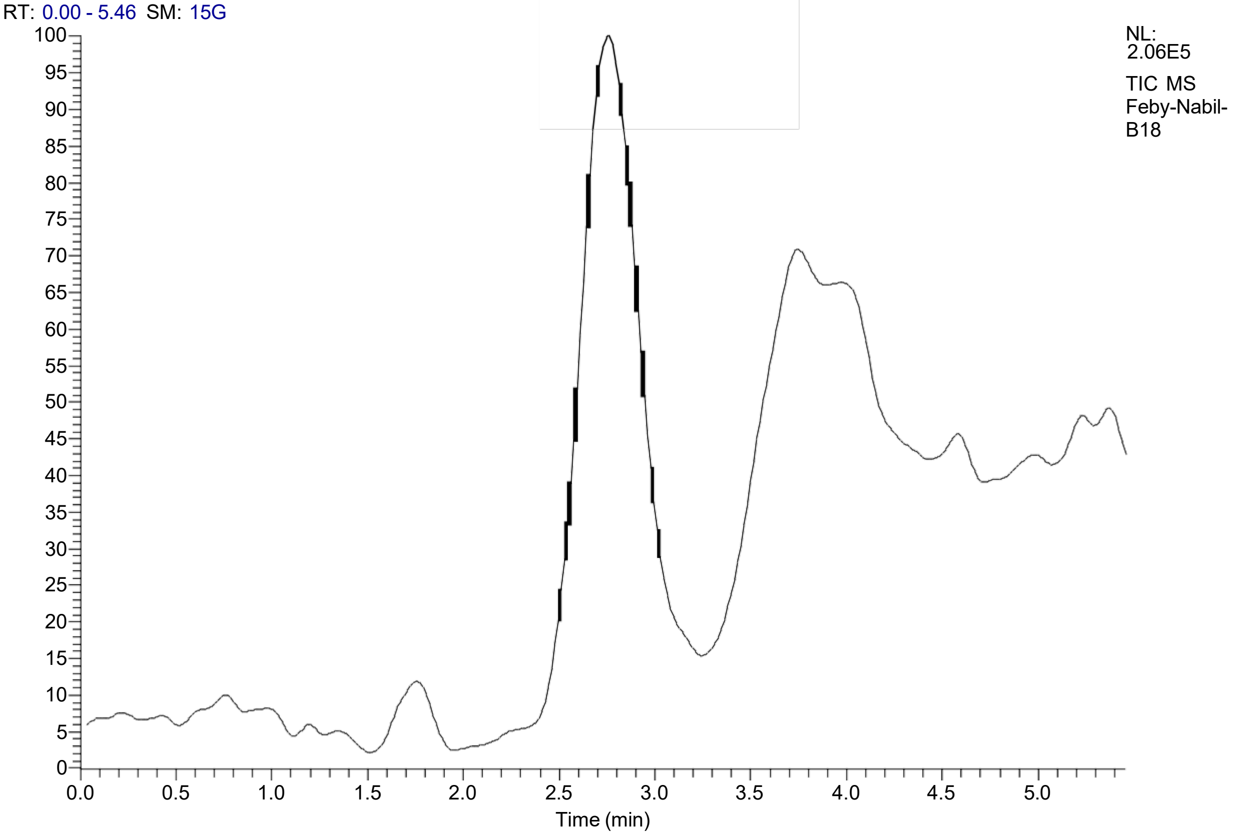

Feby-Nabil-B18 #222 RT: 3.73 AV: 1 SB: 2 4.45 , 4.45 NL: 5.76E3
T: {0,0} + c EI Full ms [40.00-1000.00]

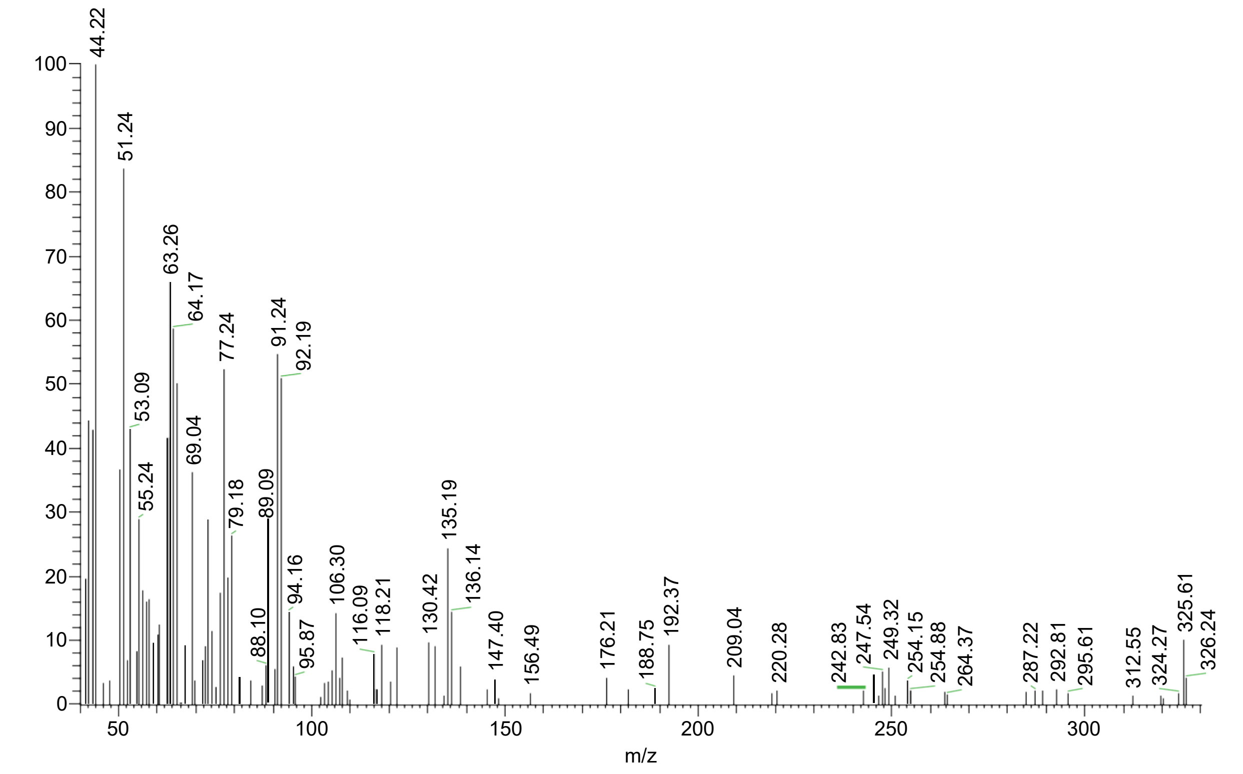

Compound 5

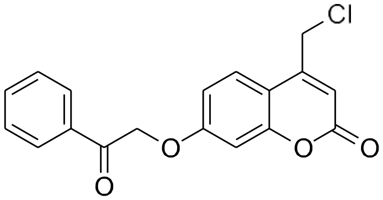
Cl
O
O
O
O

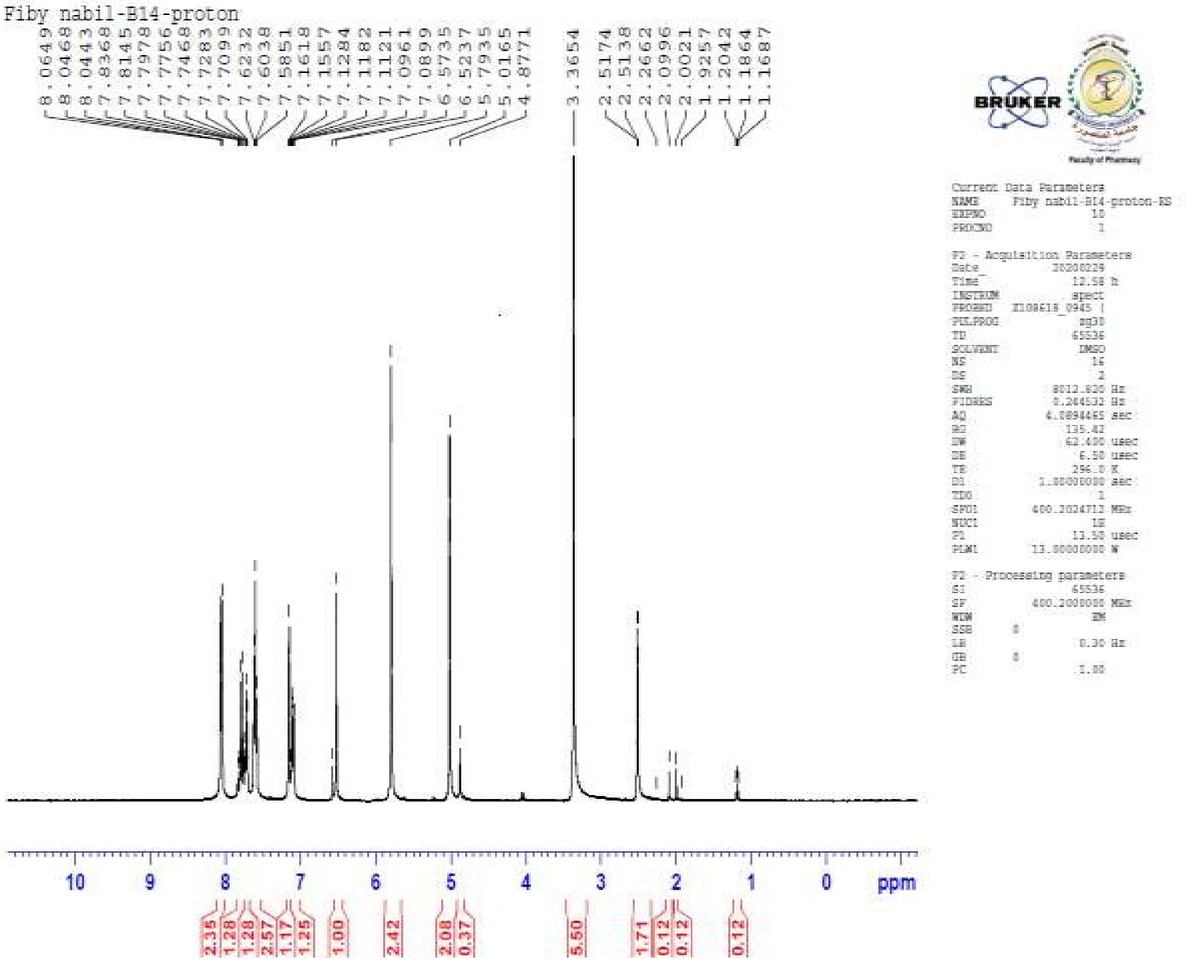
Fiby nabil-B14-proton
8.0649
8.0468
8.0443
7.8368
7.8145
7.7978
7.7756
7.7468
7.7283
7.7099
7.6232
7.6038
7.5851
7.1618
7.1557
7.1284
7.1182
7.1121
7.0961
7.0899
6.5735
6.5237
5.7935
5.0165
4.8771
3.3654
2.5174
2.5138
2.2662
2.0996
2.0021
1.9257
1.2042
1.1864
1.1687
10
9
8
7
6
5
4
3
2
1
0
ppm
2.35
1.28
1.28
2.57
1.17
1.25
1.00
2.42
2.08
0.37
5.50
1.71
0.12
0.12
0.12
BRUKER
Current Data Parameters
F2 - Acquisition Parameters
TD 65536
SOLVENT DMSO
NS 16
SFO1 400.2024712 MHz
F2 - Processing parameters
SI 65536
SF 400.2000000 MHz

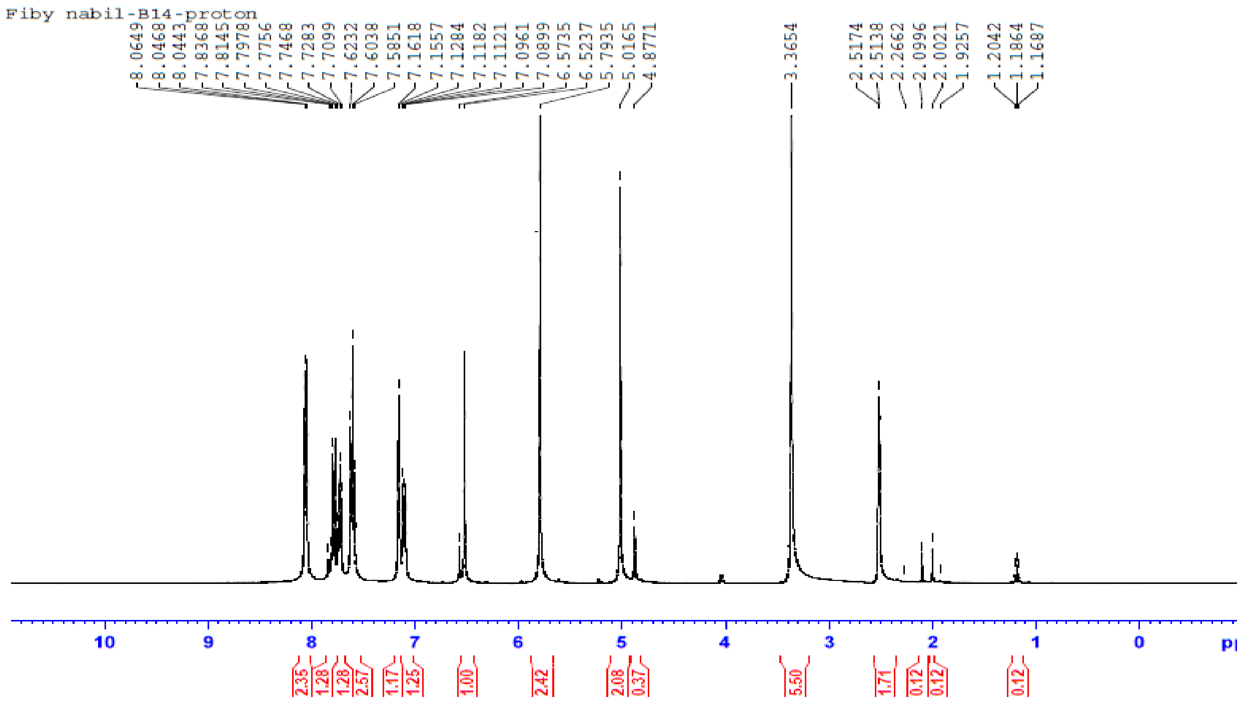
Fiby nabil-B14-proton
8.0649
8.0468
8.0443
7.8368
7.8145
7.7978
7.7756
7.7468
7.7283
7.7099
7.6232
7.6038
7.5851
7.1618
7.1557
7.1284
7.1182
7.1121
7.0961
7.0899
6.5735
6.5237
5.7935
5.0165
4.8771
3.3654
2.5174
2.5138
2.2662
2.0996
2.0021
1.9257
1.2042
1.1864
1.1687
10 9 8 7 6 5 4 3 2 1 0
2.35 1.28 1.28 2.57 1.17 1.25 1.00 2.42 2.08 0.37 5.50 1.71 0.12 0.12 0.12

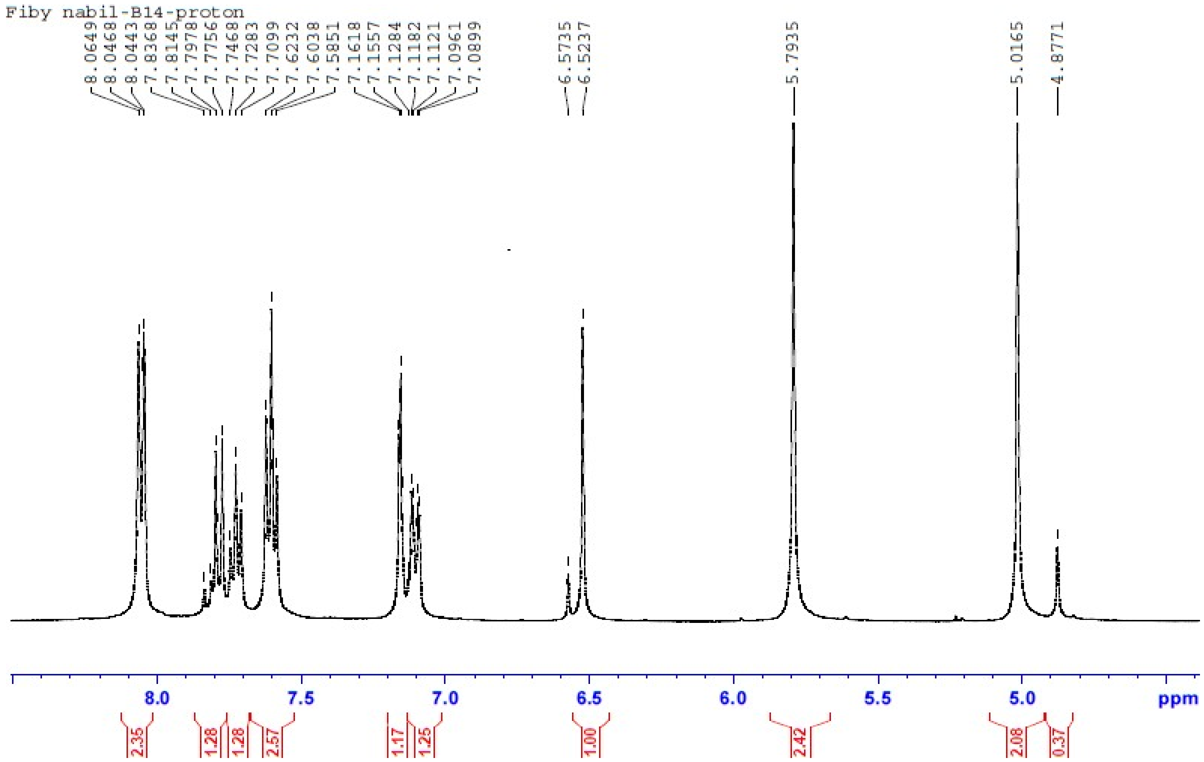
Fiby nabil-B14-proton
8.0649
8.0468
8.0443
7.8368
7.8145
7.7978
7.7756
7.7468
7.7283
7.7099
7.6232
7.6038
7.5851
7.1618
7.1557
7.1284
7.1182
7.1121
7.0961
7.0899
6.5735
6.5237
5.7935
5.0165
4.8771
8.0 7.5 7.0 6.5 6.0 5.5 5.0 ppm
2.35 1.28 1.28 2.57 1.17 1.25 1.00 2.42 2.08 0.37

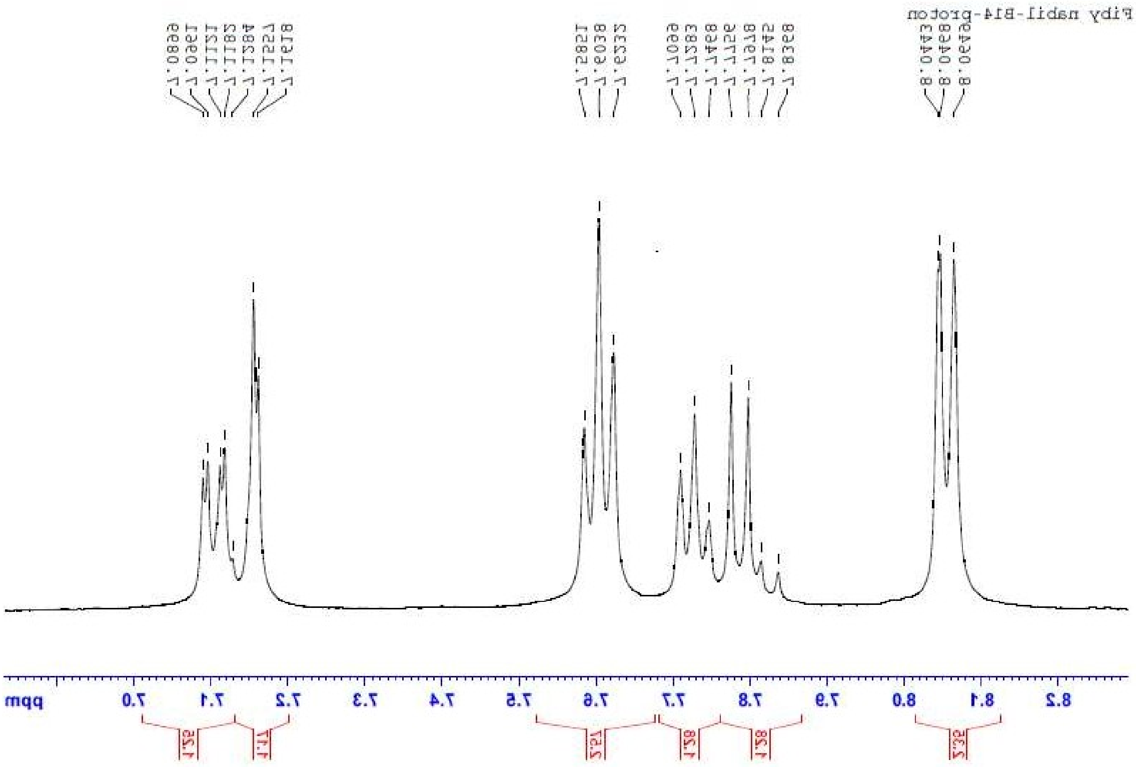
Fiby nabil-B14-proton
ppm

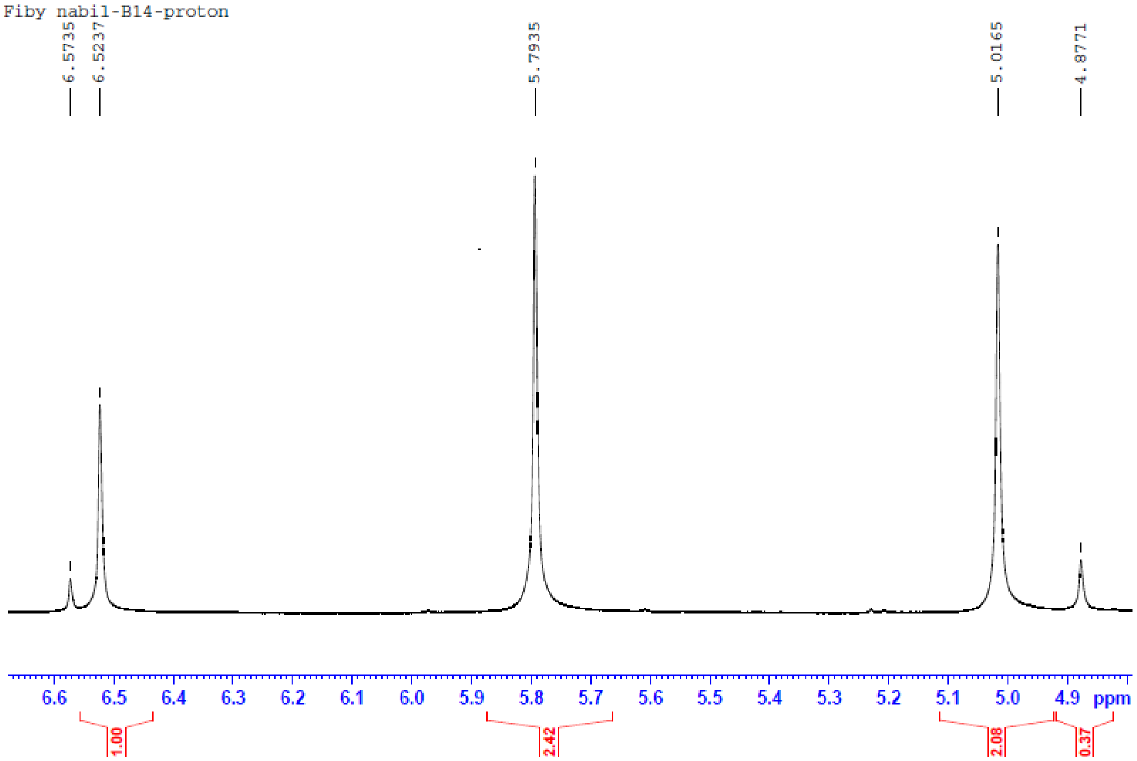

Fiby nabil-B14-proton
6.5735
6.5237
5.7935
5.0165
4.8771
6.6 6.5 6.4 6.3 6.2 6.1 6.0 5.9 5.8 5.7 5.6 5.5 5.4 5.3 5.2 5.1 5.0 4.9 ppm
1.00
2.42
2.08
0.37

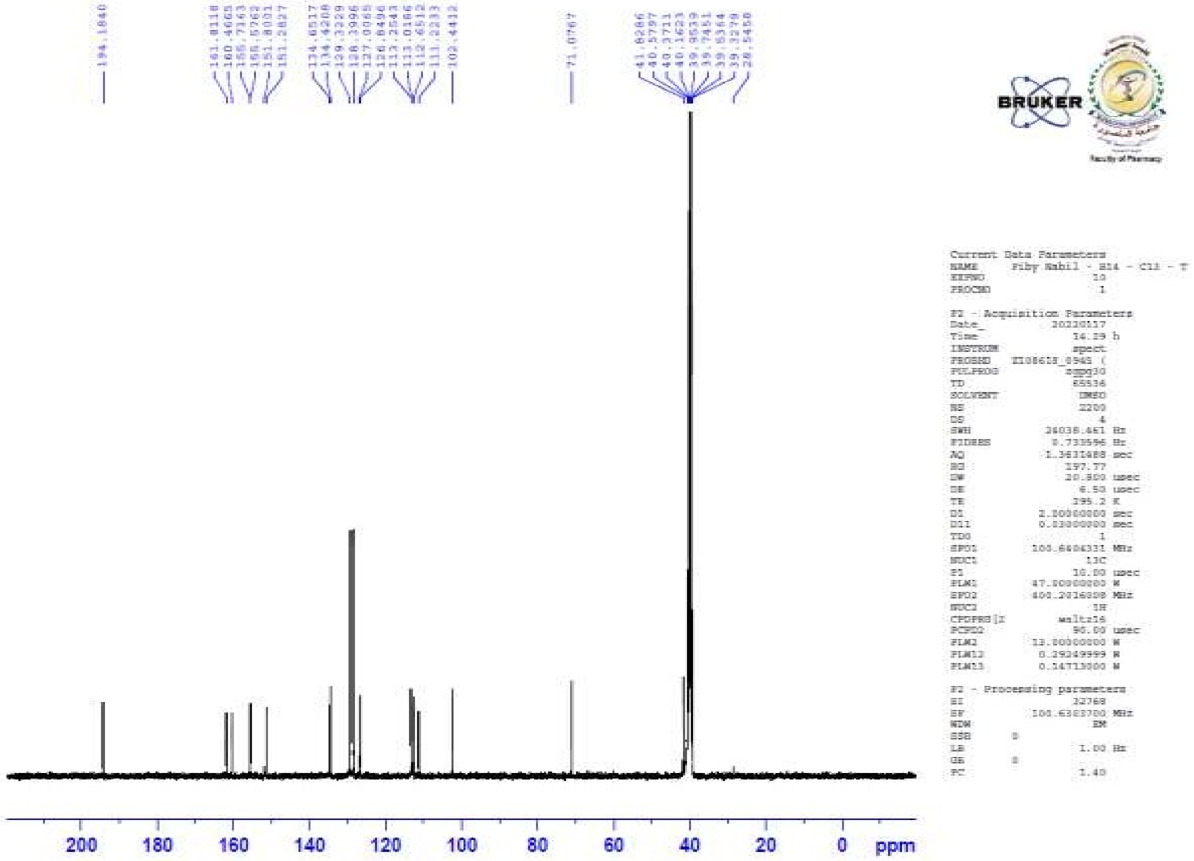
194.1840
161.8118
160.4665
155.7163
155.5762
151.8801
151.2827
134.6517
134.4208
129.3229
128.3996
127.0065
126.8496
113.2543
113.0186
112.6512
111.2233
102.4412
71.0767
41.0286
40.5797
40.3711
40.1623
39.9539
39.7451
39.5364
39.3279
28.5458
BRUKER
Faculty of Pharmacy
Current Data Parameters
NAME Fiby Nabil - H14 - C13 - T
EXPNO 10
PROCNO 1
F2 - Acquisition Parameters
Date_ 20220117
Time 14.29 h
INSTRUM spect
PULPROG zgpg30
TD 65536
SOLVENT DMSO
NS 2200
DS 4
SWH 24038.461 Hz
FIDRES 0.733596 Hz
AQ 1.3631488 sec
RG 197.77
DW 20.800 usec
DE 6.50 usec
TE 295.2 K
D1 2.00000000 sec
D11 0.03000000 sec
TD0 1
SFO1 100.6604331 MHz
NUC1 13C
P1 10.00 usec
PLW1 47.00000000 W
SFO2 400.2016008 MHz
NUC2 1H
CPDPRG[2 waltz16
PCPD2 90.00 usec
PLW2 13.00000000 W
PLW12 0.29249999 W
PLW13 0.14713000 W
F2 - Processing parameters
SI 32768
SF 100.6303700 MHz
WDW EM
SSB 0
LB 1.00 Hz
GB 0
PC 1.40
200
180
160
140
120
100
80
60
40
20
0
ppm

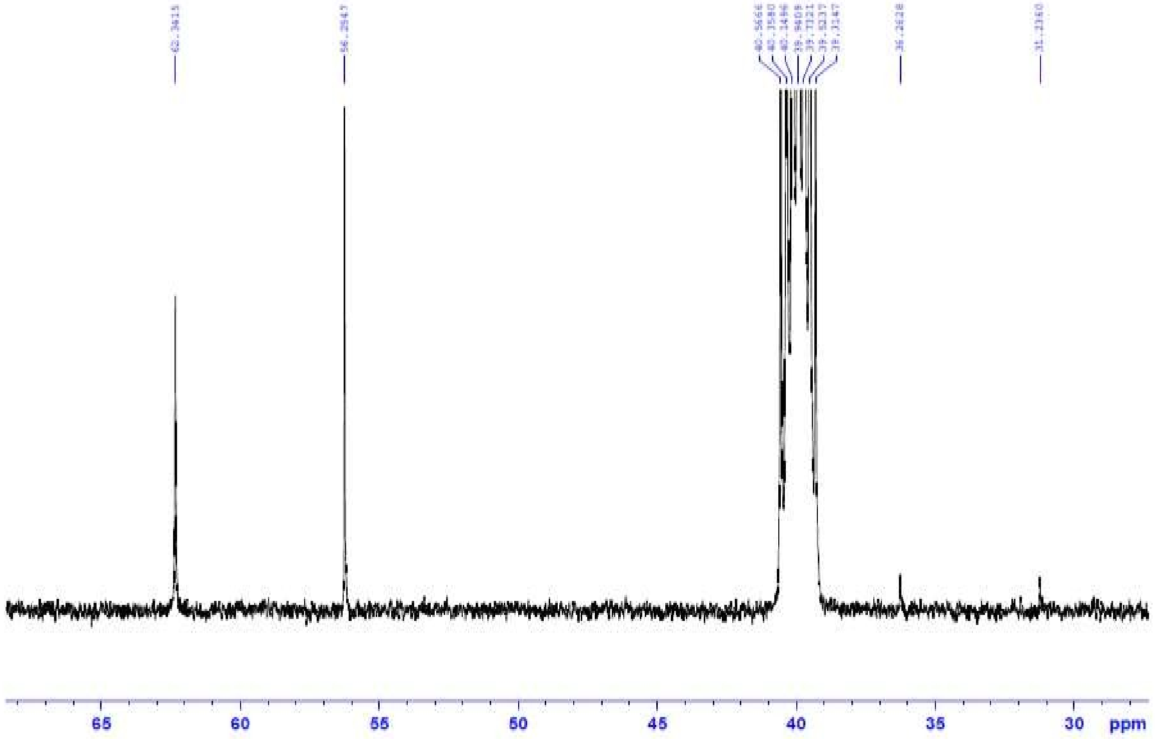
62.3415
56.2547
40.5666
40.3580
40.1486
39.9409
39.7321
39.5237
39.3147
36.2628
31.2360
65
60
55
50
45
40
35
30
ppm

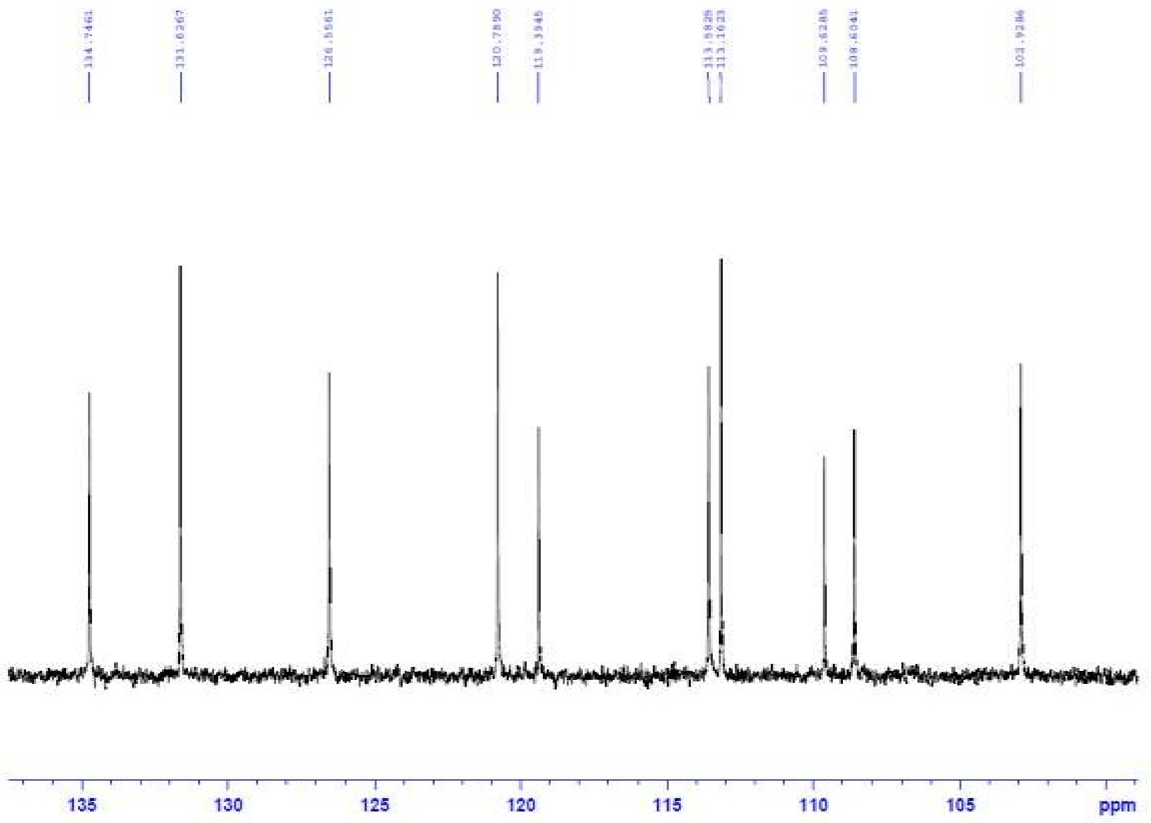
134.7461
131.6267
126.5561
120.7890
119.3945
113.5929
113.1623
109.6288
108.6041
103.9286
135
130
125
120
115
110
105
ppm

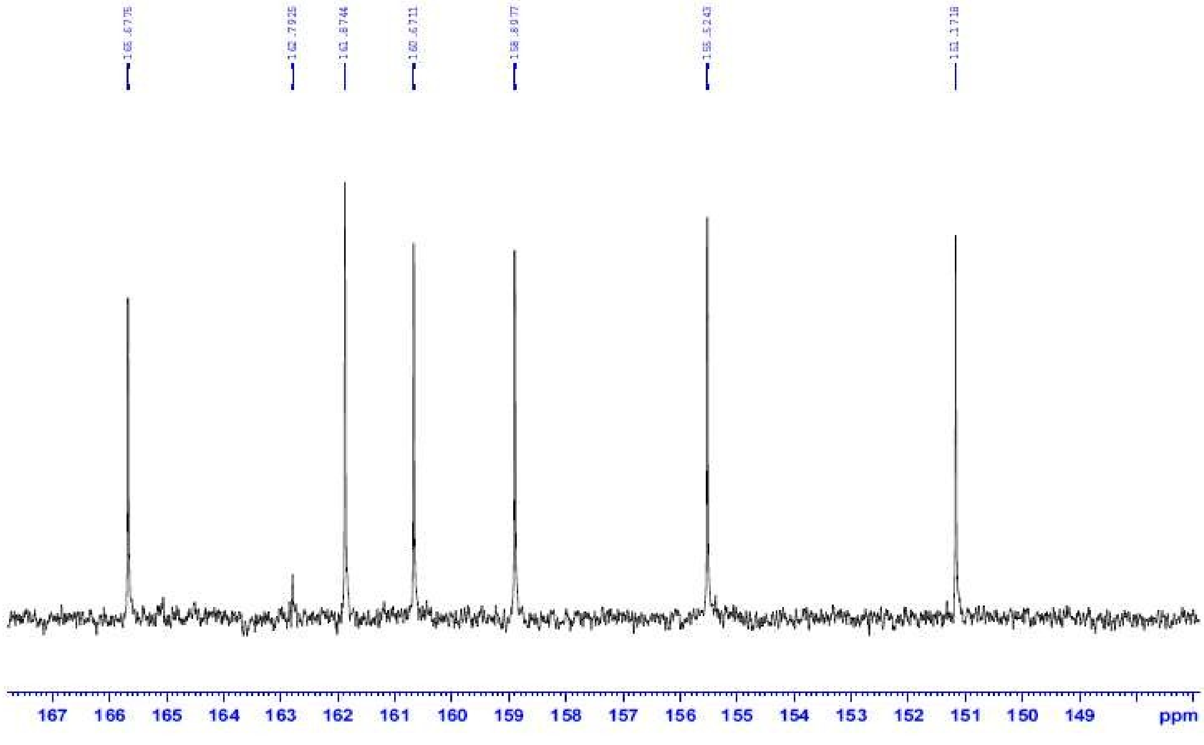
165.6775
162.7925
161.8744
160.6711
158.8977
155.5243
151.1718
167
166
165
164
163
162
161
160
159
158
157
156
155
154
153
152
151
150
149
ppm

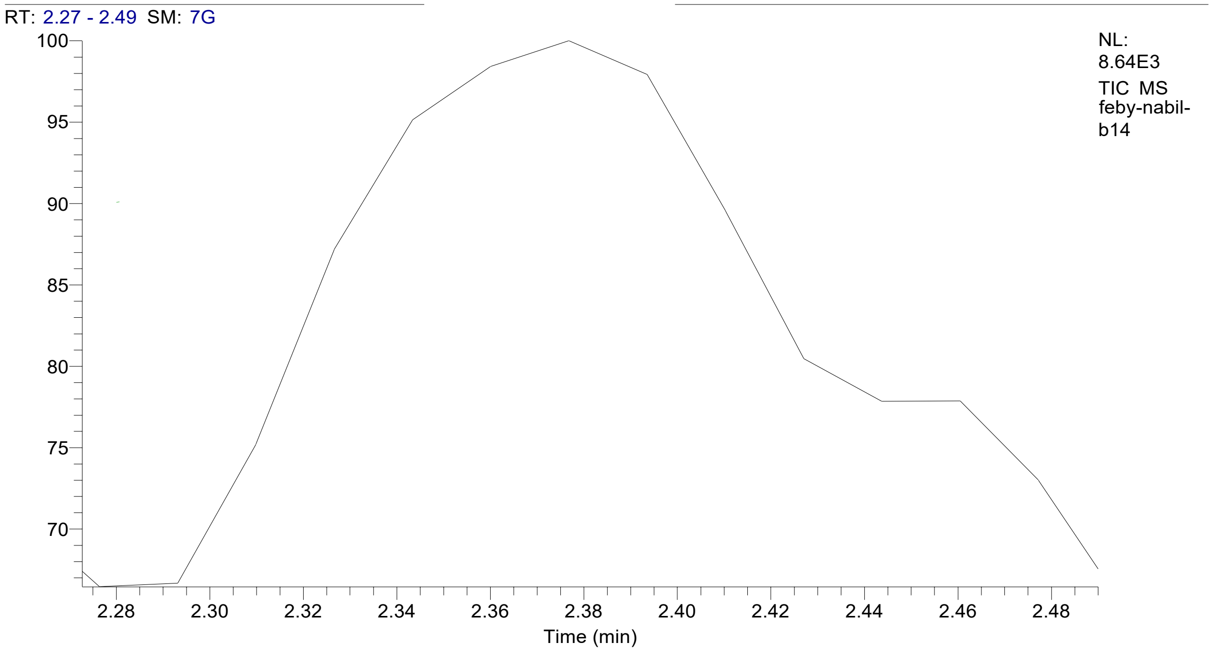

RT: 2.27 - 2.49 SM: 7G
NL:
8.64E3
TIC MS
feby-nabil-
b14
Time (min)

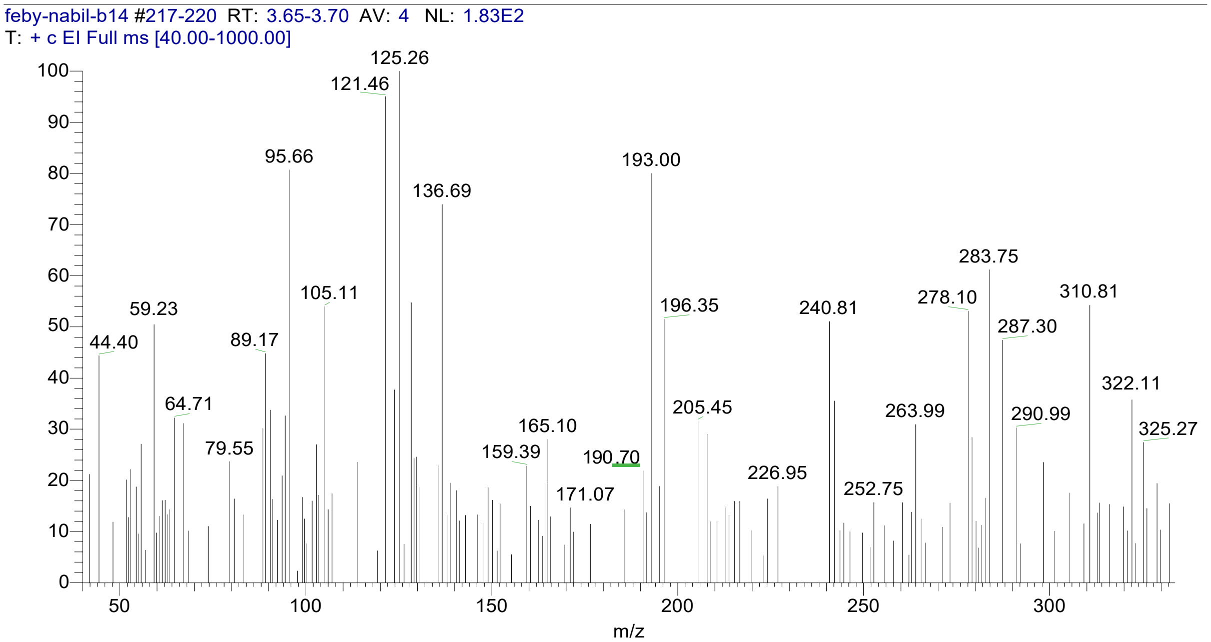

feby-nabil-b14 #217-220 RT: 3.65-3.70 AV: 4 NL: 1.83E2
T: + c EI Full ms [40.00-1000.00]
125.26
121.46
95.66
193.00
136.69
283.75
310.81
59.23
105.11
196.35
240.81
278.10
44.40
89.17
287.30
322.11
64.71
205.45
263.99
290.99
325.27
79.55
165.10
159.39
190.70
226.95
171.07
252.75
m/z

Compound 6

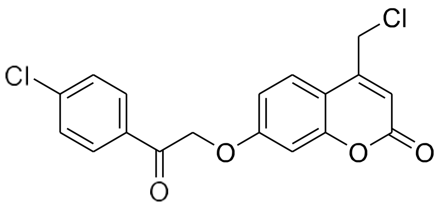

Cl
Cl
O
O
O
O

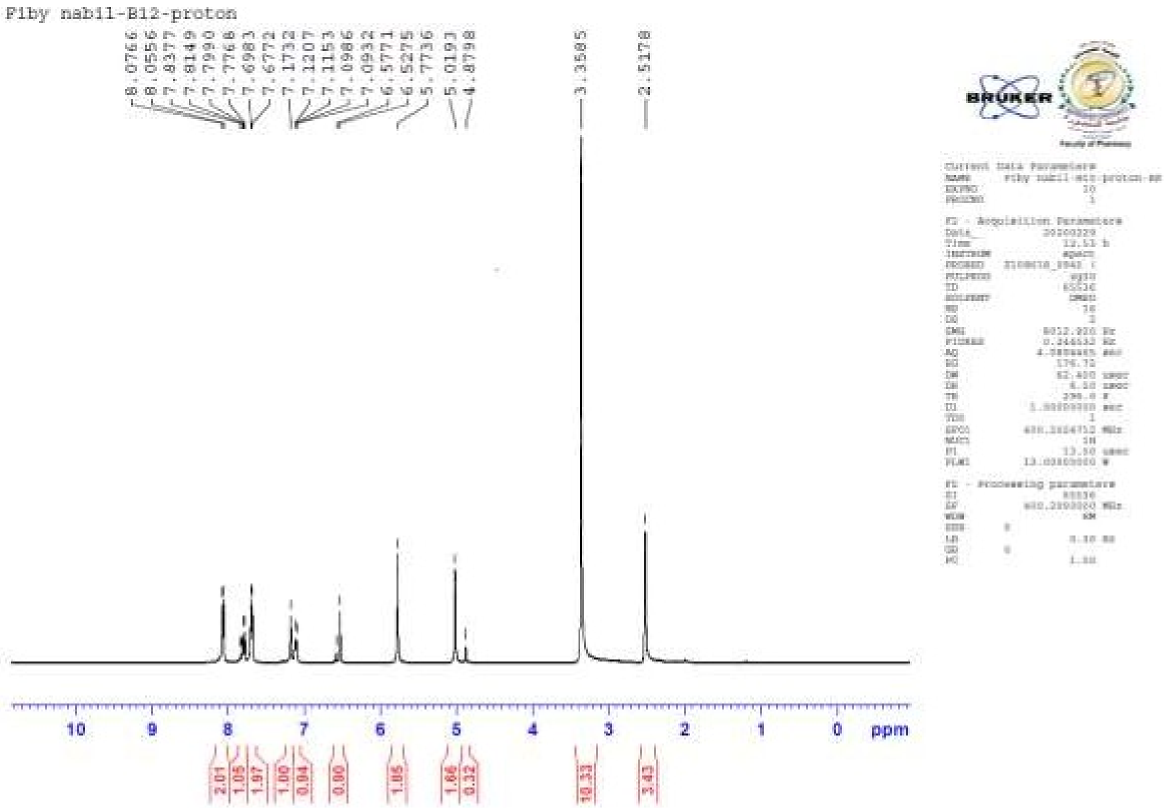

Fiby nabil-B12-proton
8.0766
8.0556
7.8377
7.8149
7.7990
7.7768
7.6983
7.6772
7.1732
7.1207
7.1153
7.0986
7.0932
6.5771
6.5275
5.7736
5.0193
4.8798
3.3505
2.5178
BRUKER
10
9
8
7
6
5
4
3
2
1
0
ppm
2.01
1.05
1.97
1.00
0.94
0.90
1.95
1.86
0.32
10.33
3.43

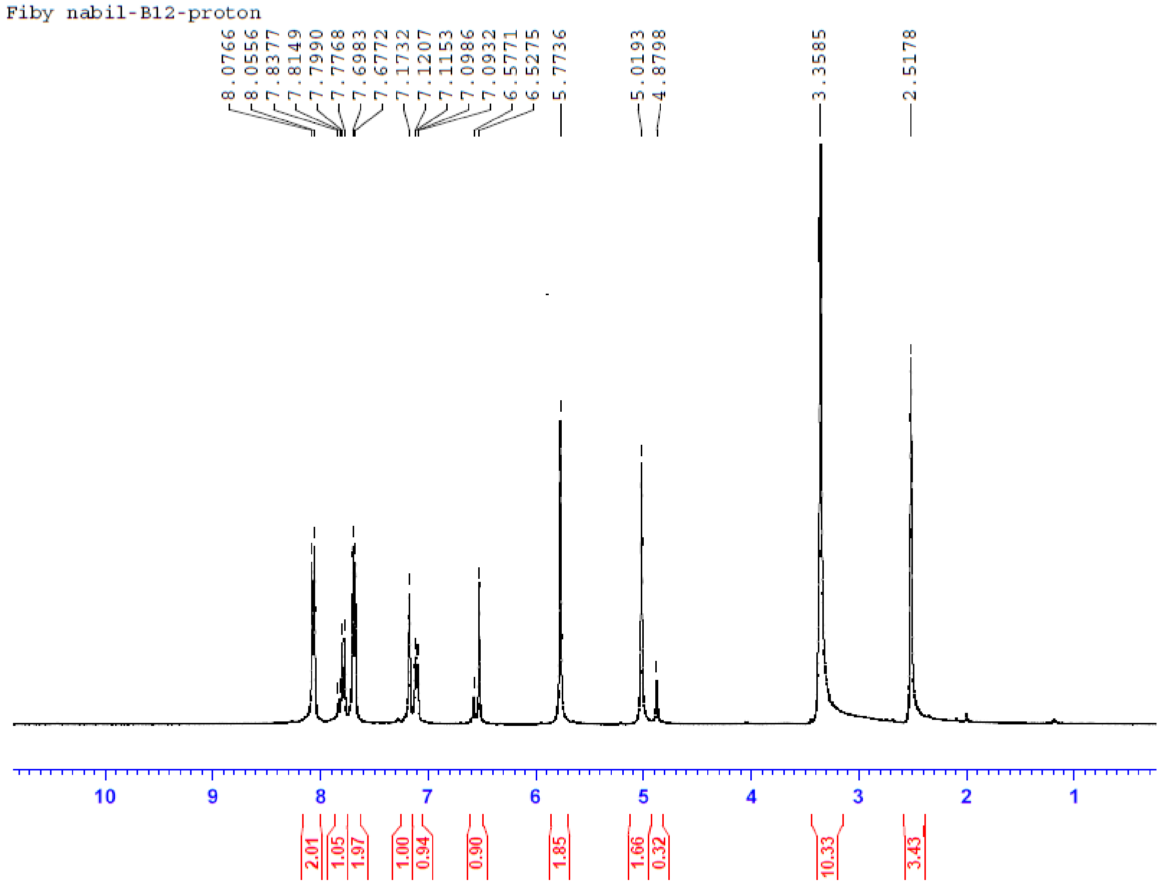
Fiby nabil-B12-proton
8.0766
8.0556
7.8377
7.8149
7.7990
7.7768
7.6983
7.6772
7.1732
7.1207
7.1153
7.0986
7.0932
6.5771
6.5275
5.7736
5.0193
4.8798
3.3585
2.5178
10
9
8
7
6
5
4
3
2
1
2.01
1.05
1.97
1.00
0.94
0.90
1.85
1.66
0.32
10.33
3.43

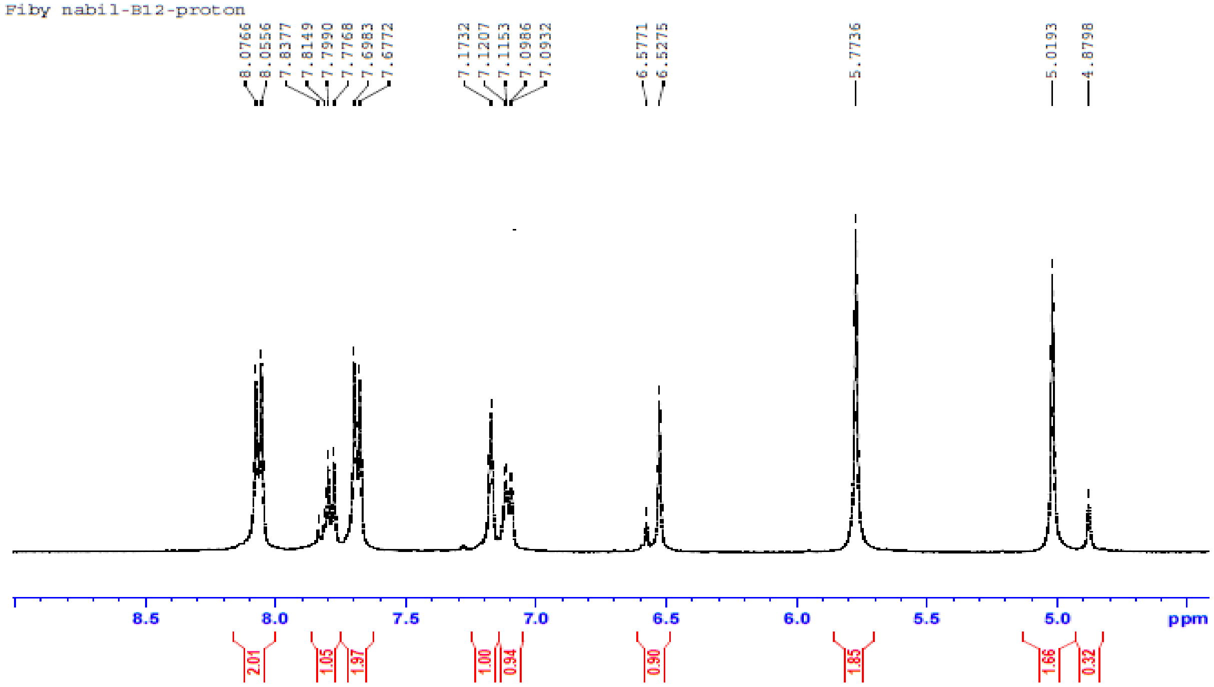
Fiby nabil-B12-proton
8.0766
8.0556
7.8377
7.8149
7.7990
7.7768
7.6983
7.6772
7.1732
7.1207
7.1153
7.0986
7.0932
6.5771
6.5275
5.7736
5.0193
4.8798
8.5
8.0
7.5
7.0
6.5
6.0
5.5
5.0
ppm
2.01
1.05
1.97
1.00
0.94
0.90
1.85
1.66
0.32

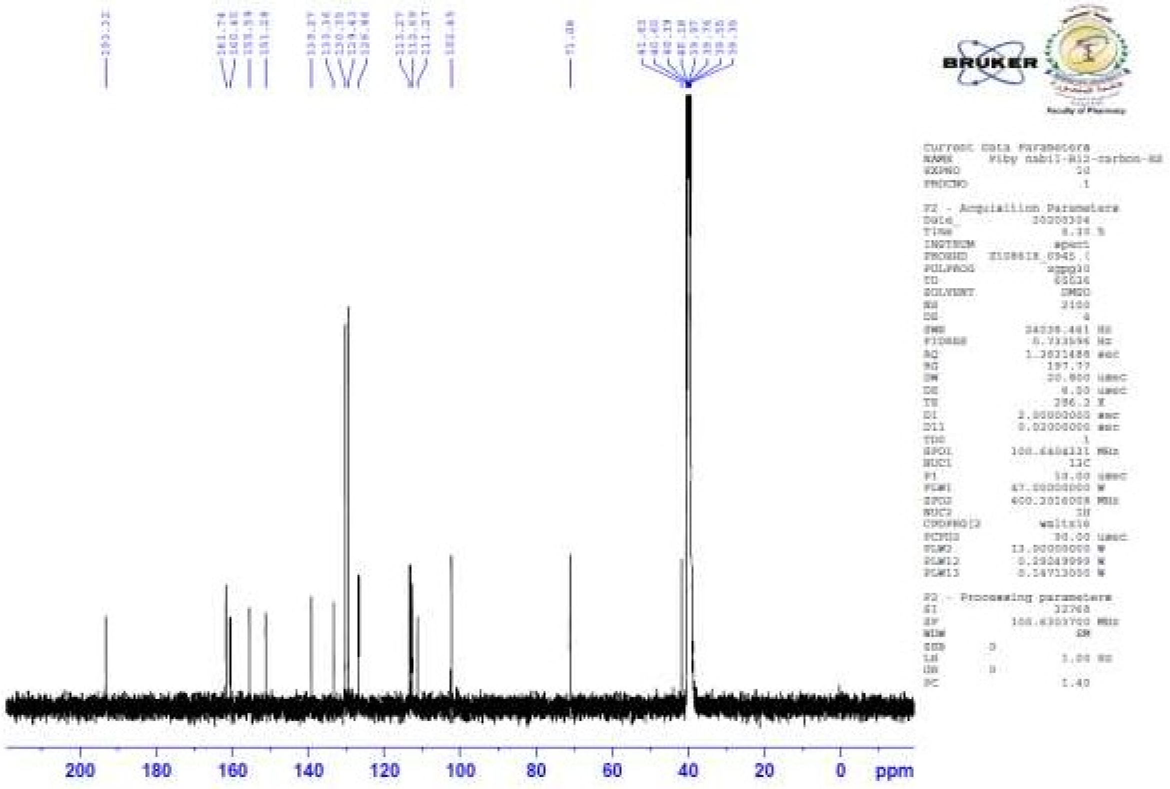
BRUKER
Faculty of Pharmacy
200
180
160
140
120
100
80
60
40
20
0
ppm

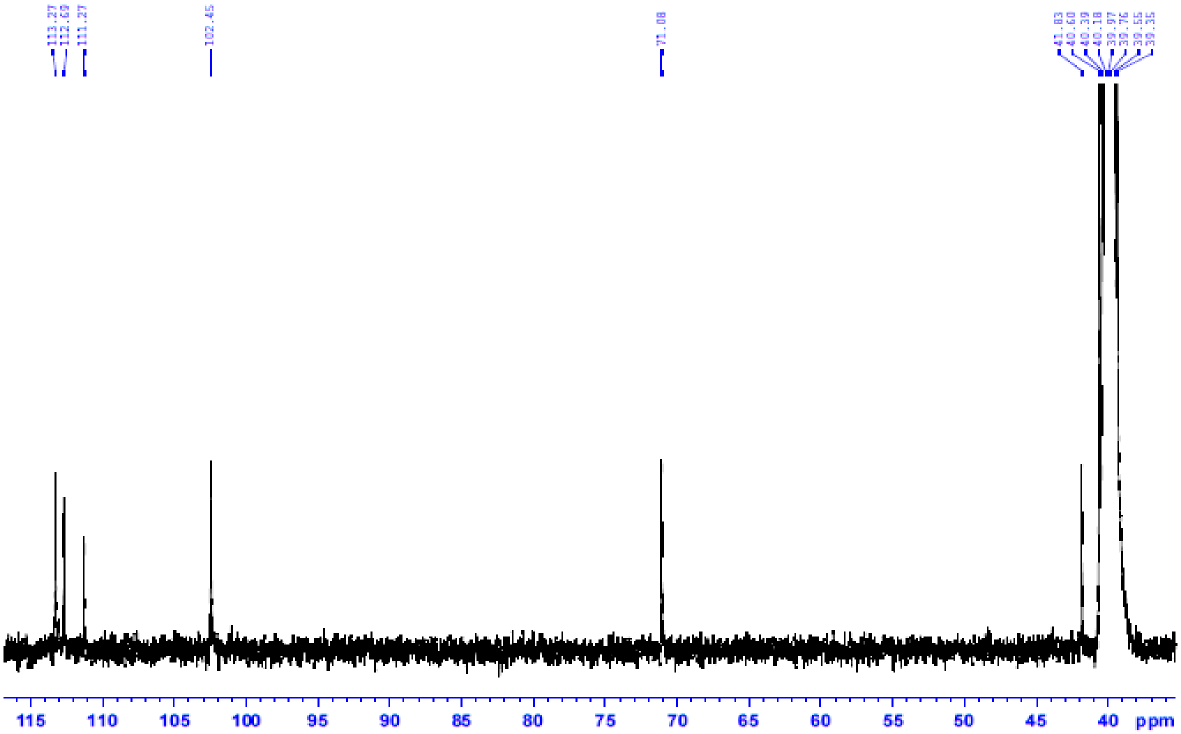
113.27
112.69
111.27
102.45
71.08
41.83
40.60
40.39
40.18
39.97
39.76
39.55
39.35
115 110 105 100 95 90 85 80 75 70 65 60 55 50 45 40 ppm

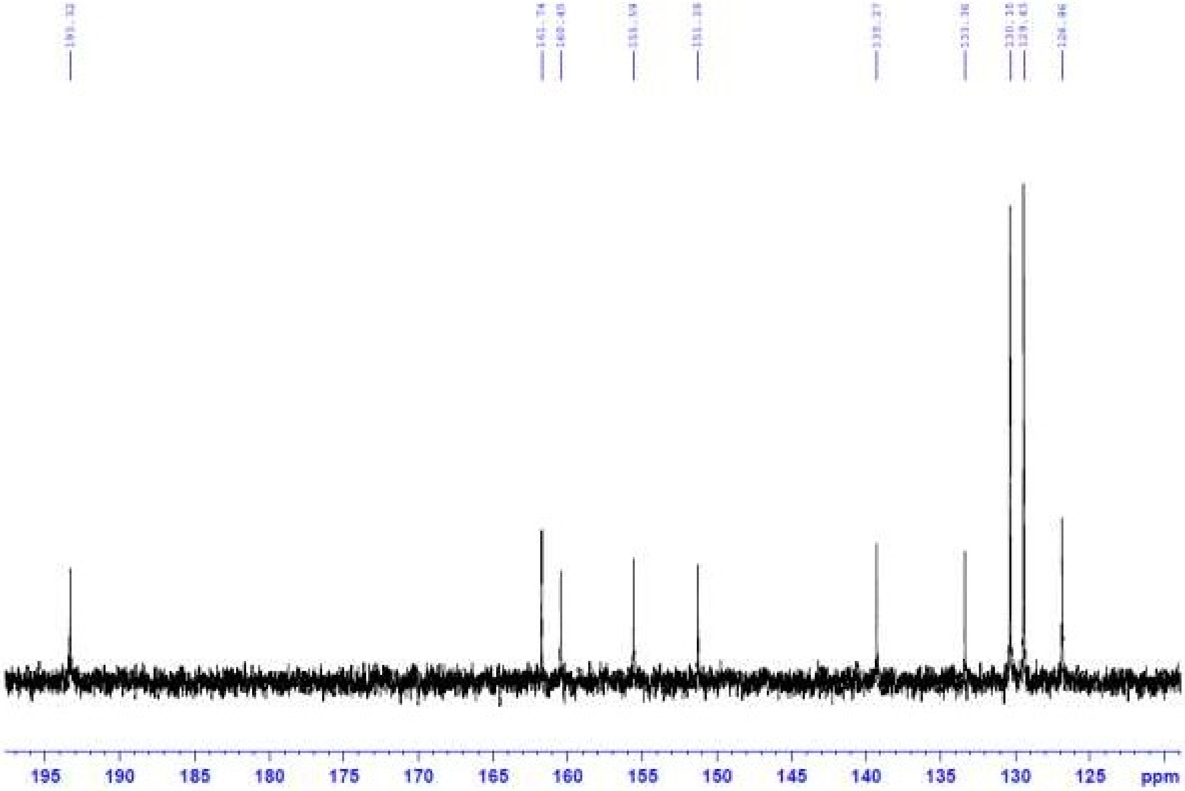
195 190 185 180 175 170 165 160 155 150 145 140 135 130 125 ppm

C:\Xcalibur\data\S\Feby-Nabil-B12 20-Oct-20 3:58:47 PM
THE [illegible]OGY AND BIOTECHNOLOGY
AZH[illegible]

RT: 0.00 - 5.42 SM: 15G

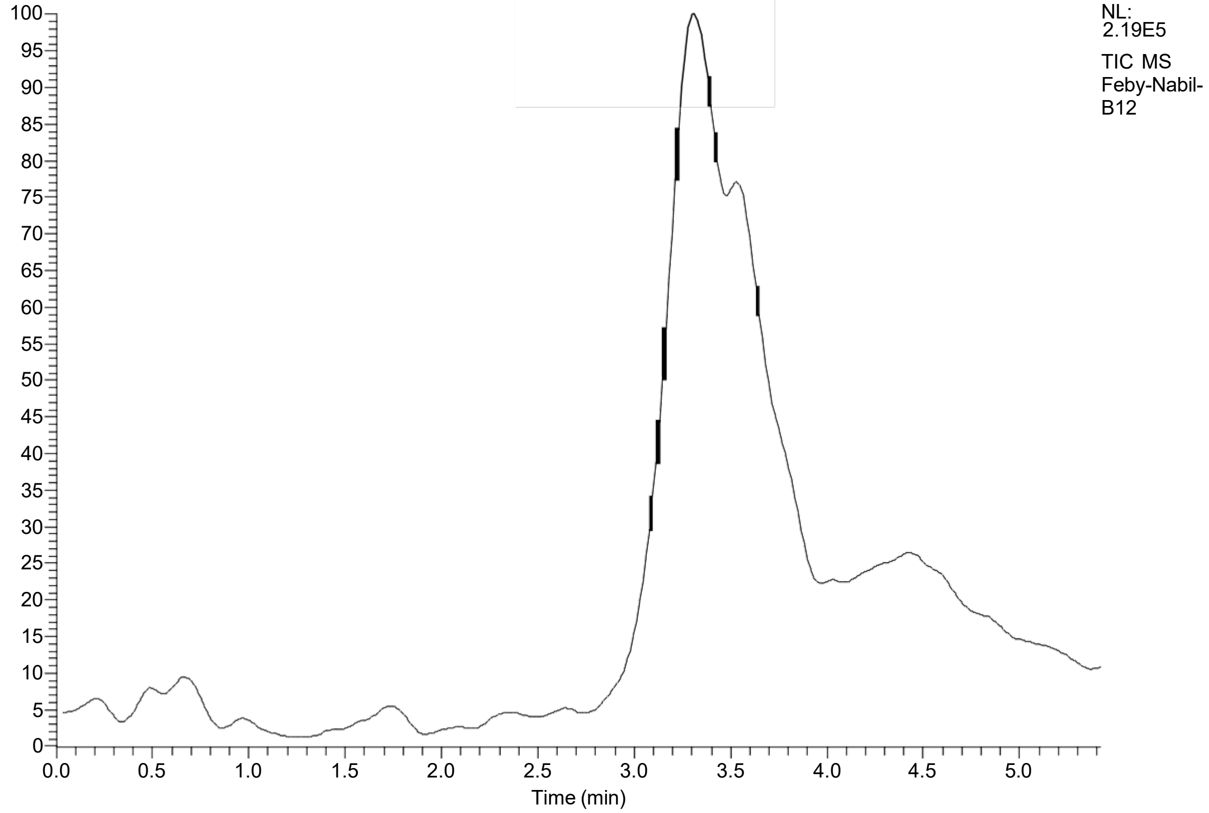

Feby-Nabil-B12 #199 RT: 3.35 AV: 1 SB: 2 4.45 , 4.45 NL: 2.79E4
T: {0,0} + c EI Full ms [40.00-1000.00]

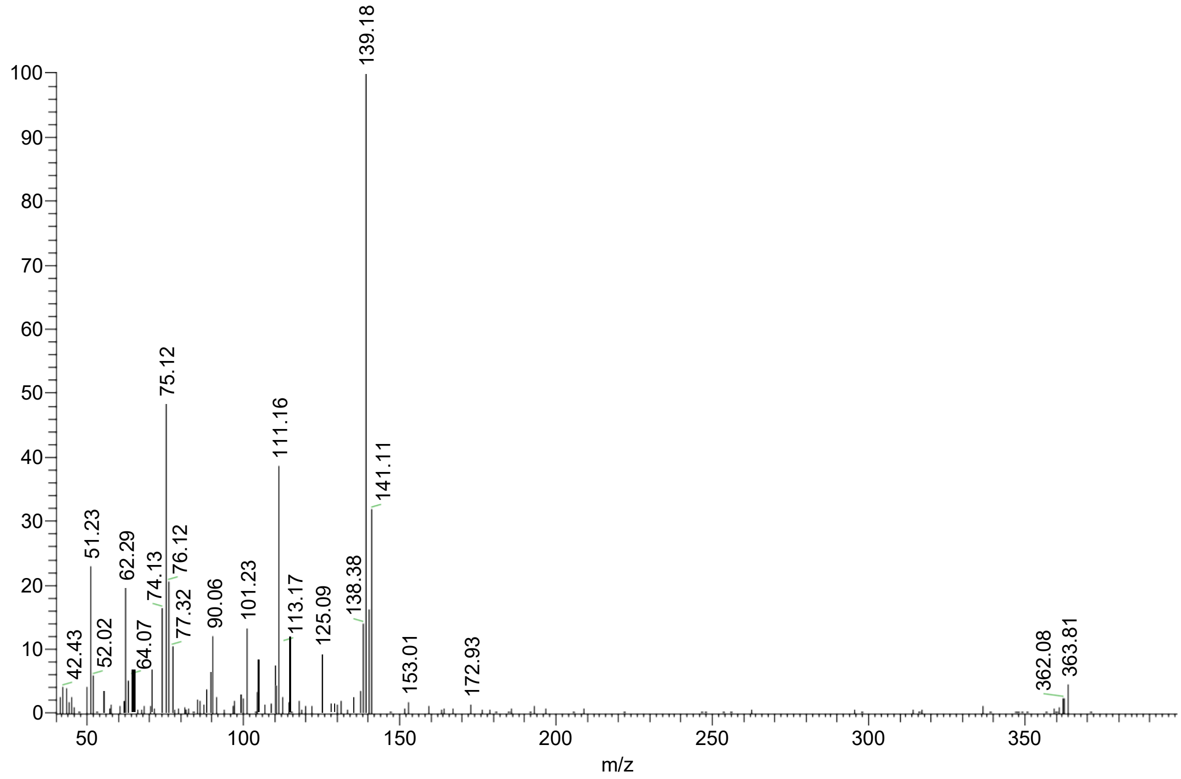

Compound 7

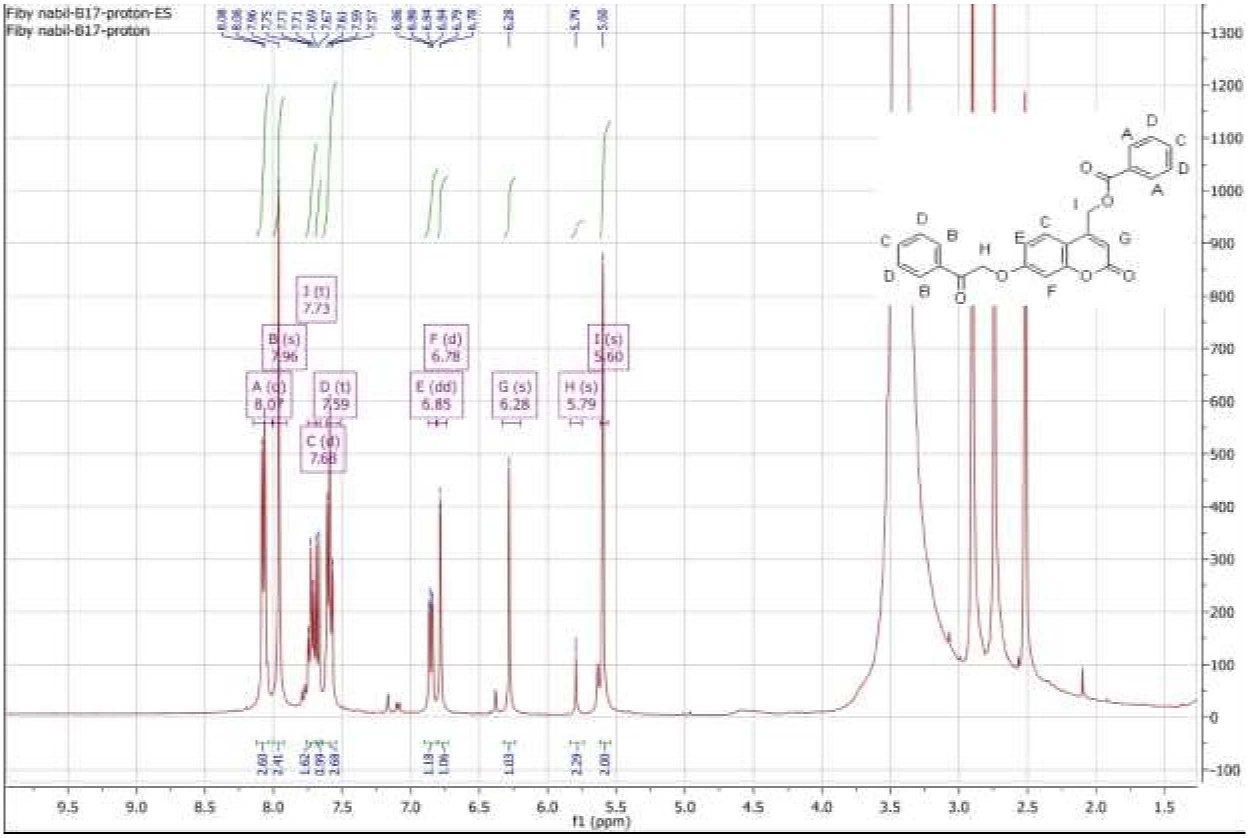

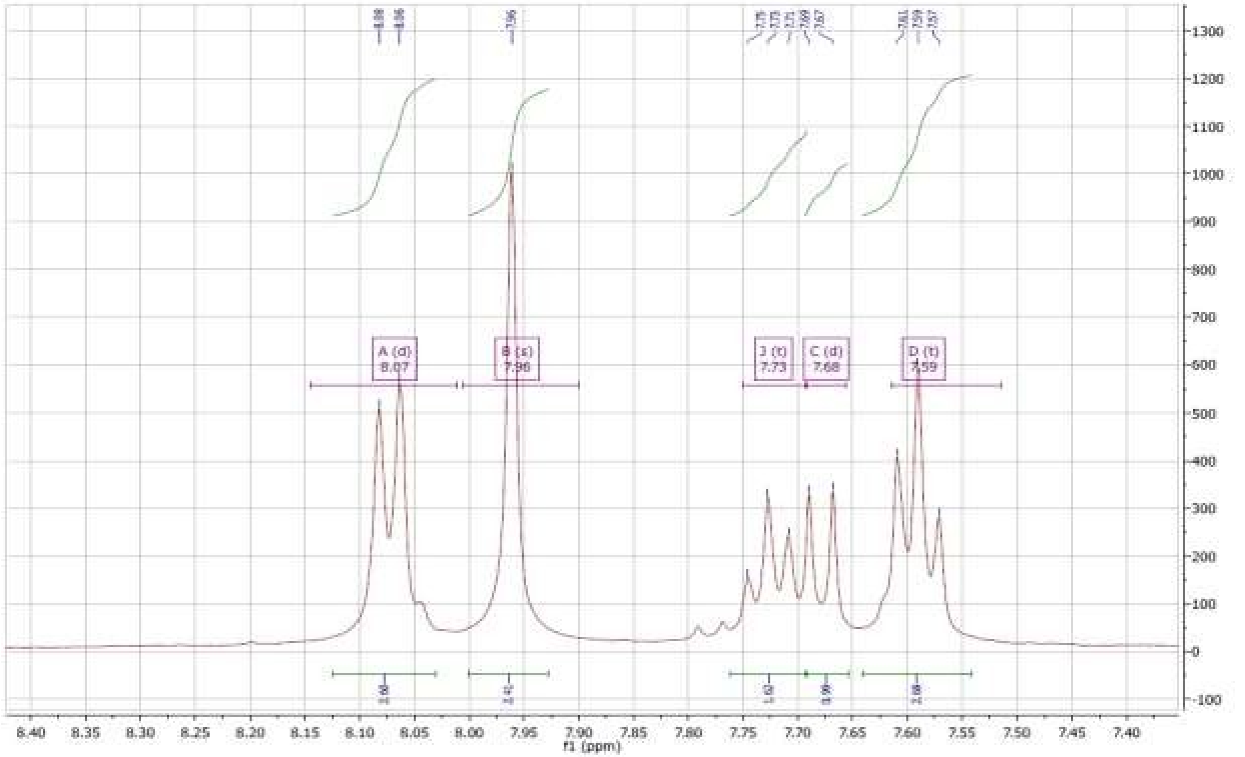

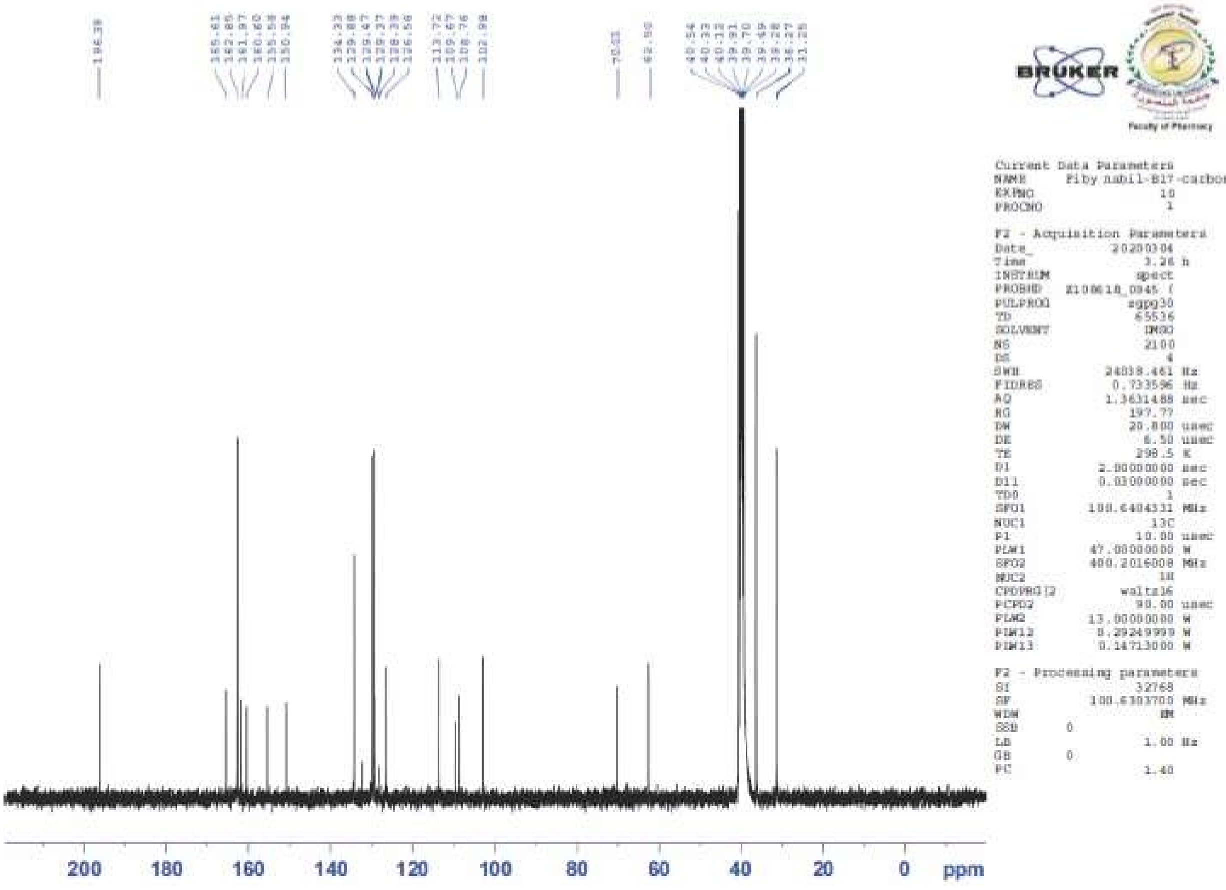
196.39
165.61
162.85
161.97
160.60
155.58
150.94
134.33
129.88
129.47
129.37
128.39
126.56
113.72
109.67
108.76
102.98
70.03
62.50
40.54
40.33
40.12
39.91
39.70
39.49
39.28
36.27
31.25
BRUKER
Faculty of Pharmacy
Current Data Parameters
NAME Fiby nabil-B17-carbon-E
EXPNO 10
PROCNO 1
F2 - Acquisition Parameters
Date_ 20200304
Time 3.26 h
INSTRUM spect
PROBHD Z108618_0945 (
PULPROG zgpg30
TD 65536
SOLVENT DMSO
NS 2100
DS 4
SWH 24038.461 Hz
FIDRES 0.733596 Hz
AQ 1.3631488 sec
RG 197.77
DW 20.800 usec
DE 6.50 usec
TE 298.5 K
D1 2.00000000 sec
D11 0.03000000 sec
TD0 1
SFO1 100.6404331 MHz
NUC1 13C
P1 10.00 usec
PLW1 47.00000000 W
SFO2 400.2016008 MHz
NUC2 1H
CPDPRG[2 waltz16
PCPD2 90.00 usec
PLW2 13.00000000 W
PLW12 0.29249999 W
PLW13 0.14713000 W
F2 - Processing parameters
SI 32768
SF 100.6303700 MHz
WDW EM
SSB 0
LB 1.00 Hz
GB 0
PC 1.40
200
180
160
140
120
100
80
60
40
20
0
ppm

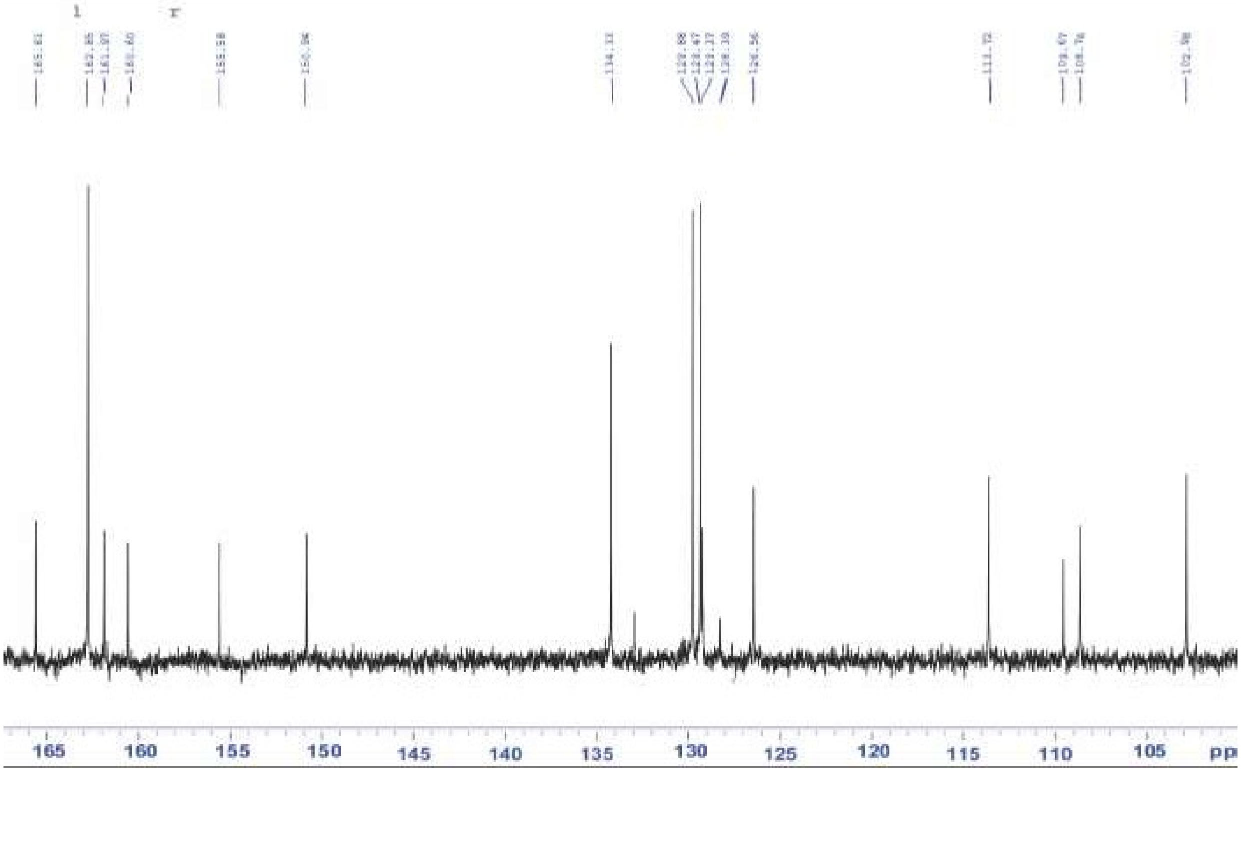
165.61
162.85
161.97
160.60
155.58
150.94
134.33
129.88
129.47
129.37
128.39
126.56
113.72
109.67
108.76
102.98
165
160
155
150
145
140
135
130
125
120
115
110
105
pp

C:\Xcalibur\data\S\Feby-Nabil-B17 20-Oct-20 3:12:54 PM

THE OGY AND BIOTECHNOLOGY

AZH

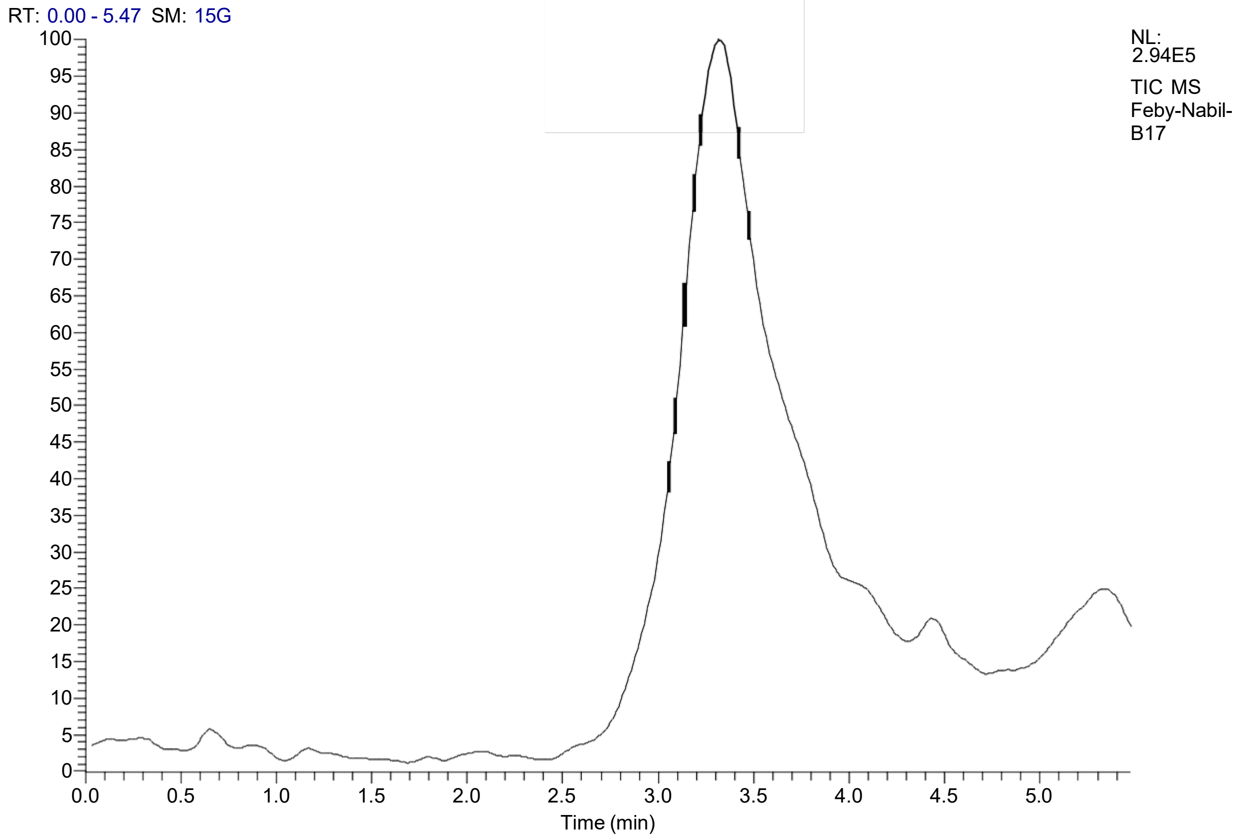

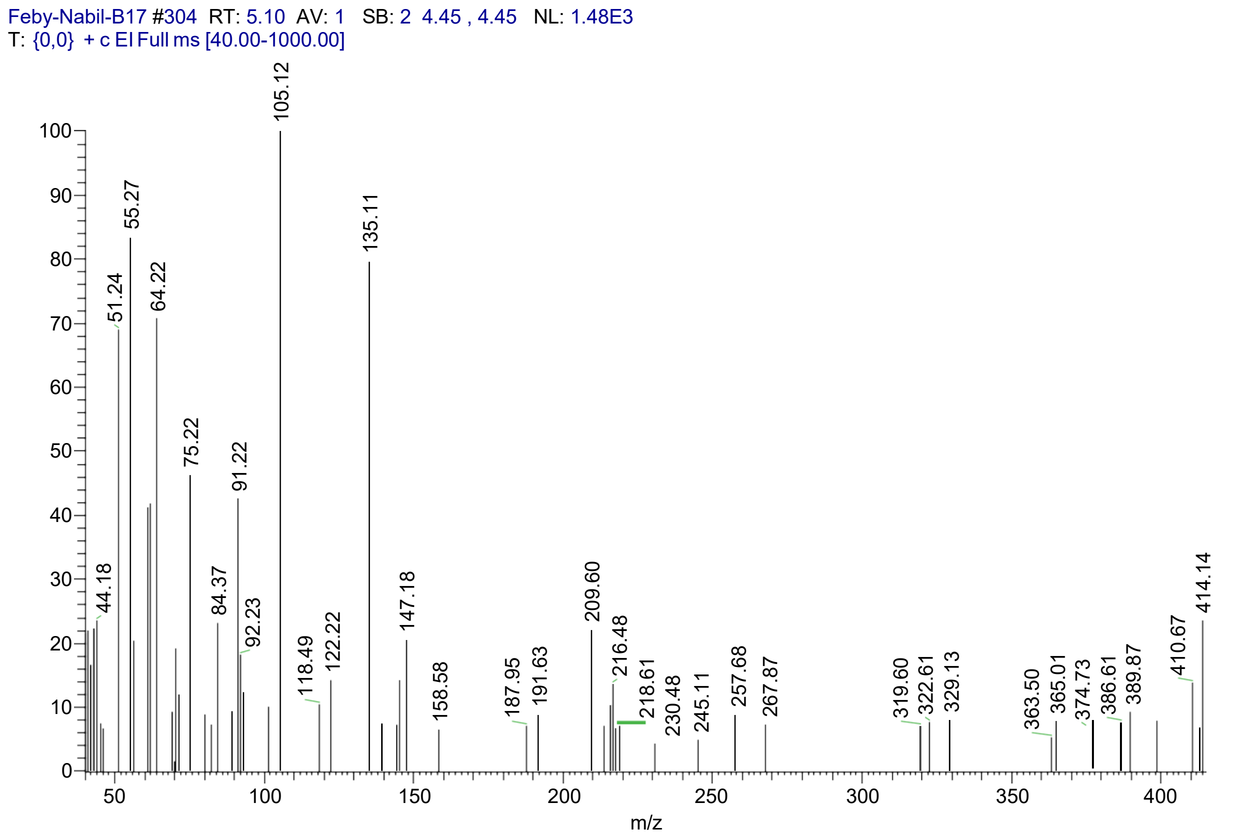

Compound 8

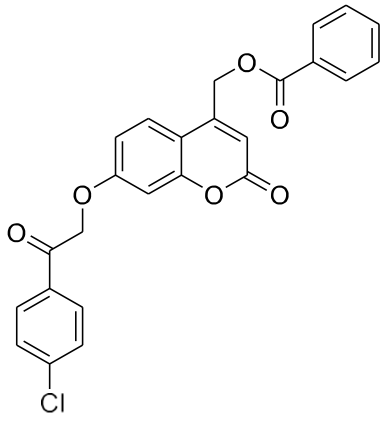
O
O
O
O
O
O
Cl

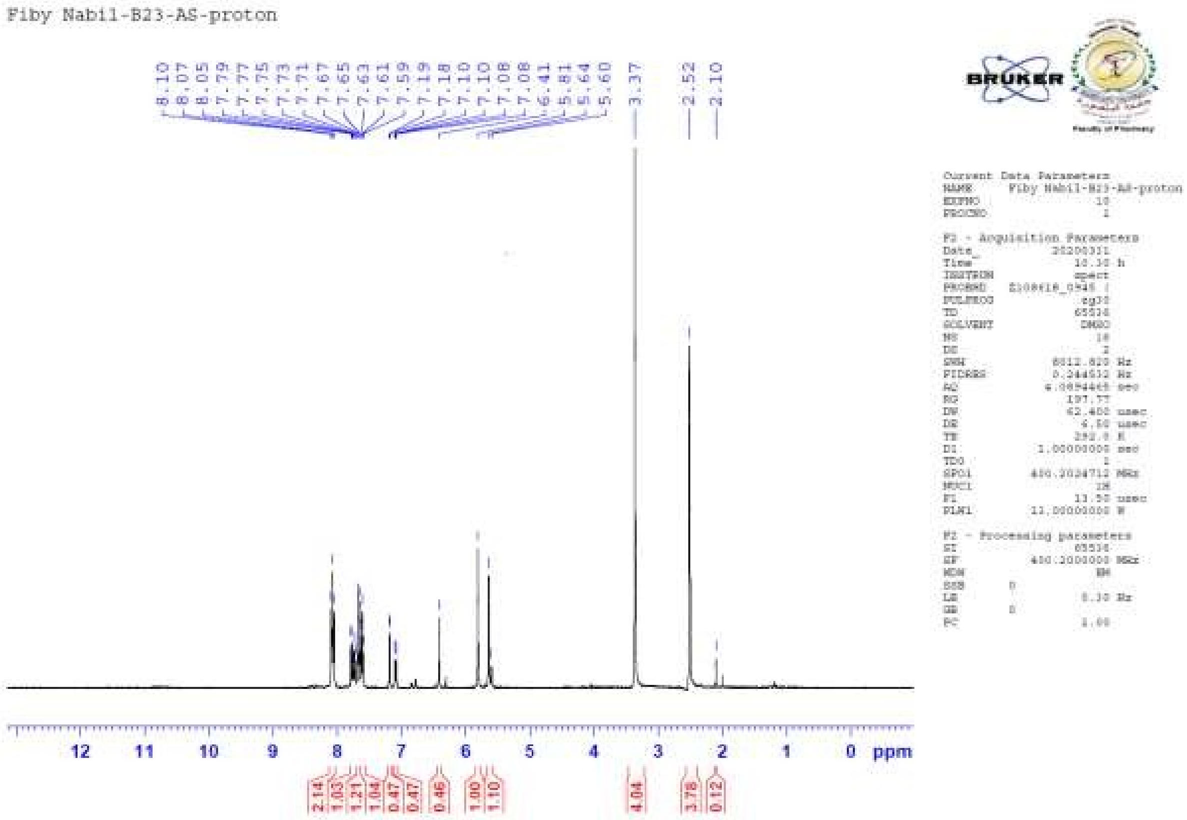
Fiby Nabil-B23-AS-proton
8.10 8.07 8.05 7.79 7.77 7.75 7.73 7.71 7.67 7.65 7.63 7.61 7.59 7.19 7.18 7.10 7.10 7.08 7.08 6.41 5.81 5.64 5.60 3.37 2.52 2.10
BRUKER
12 11 10 9 8 7 6 5 4 3 2 1 0 ppm
2.14 1.03 1.21 1.04 0.47 0.47 0.46 1.00 1.10 4.04 3.76 0.12

Fiby Nabil-B23-AS-proton

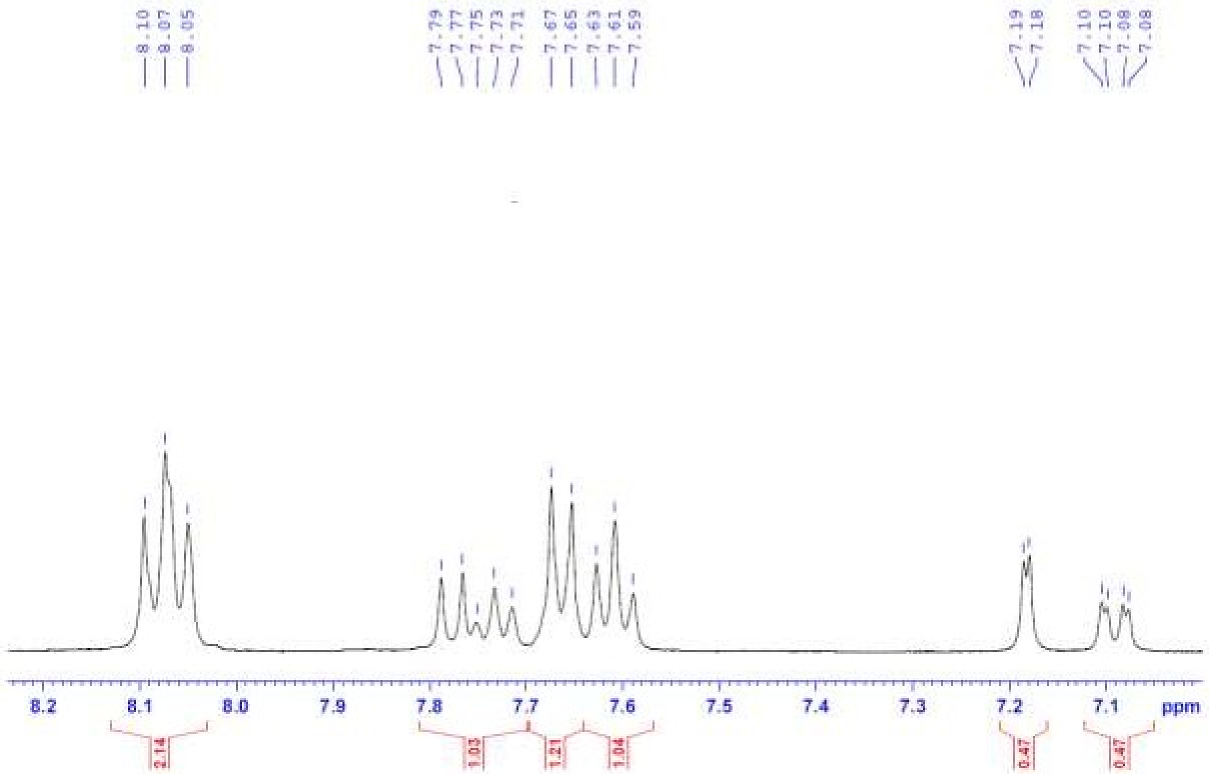

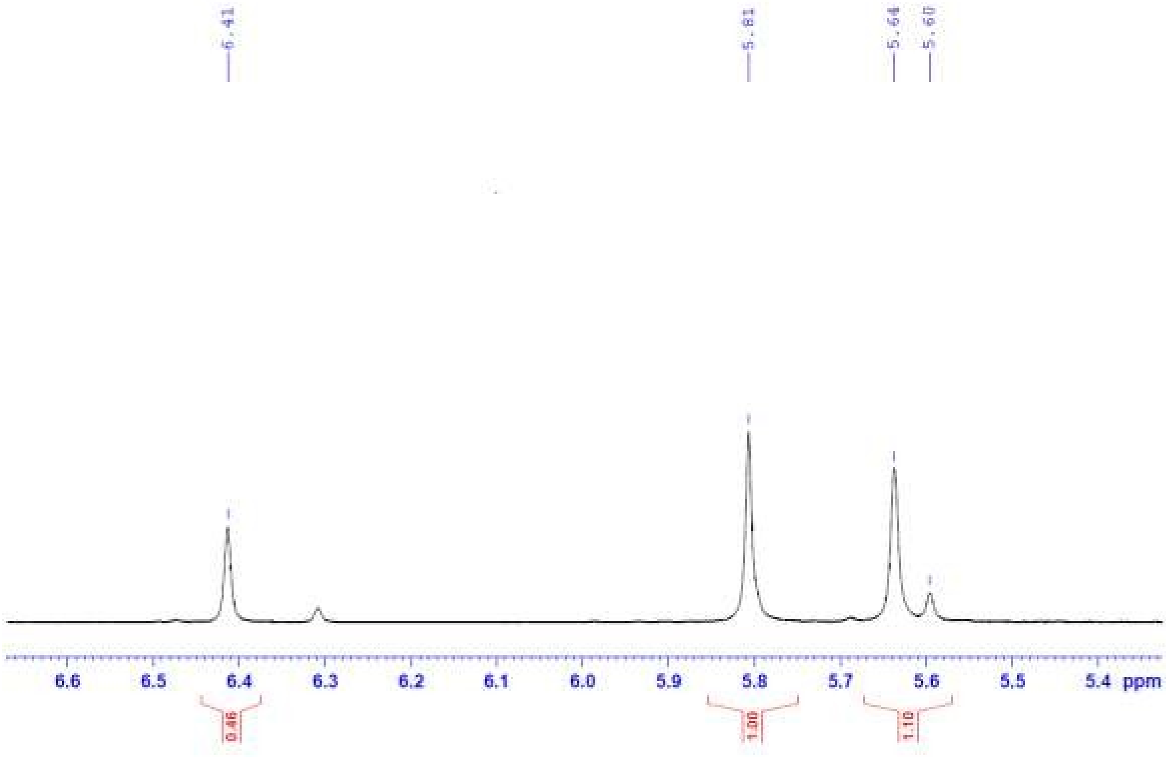

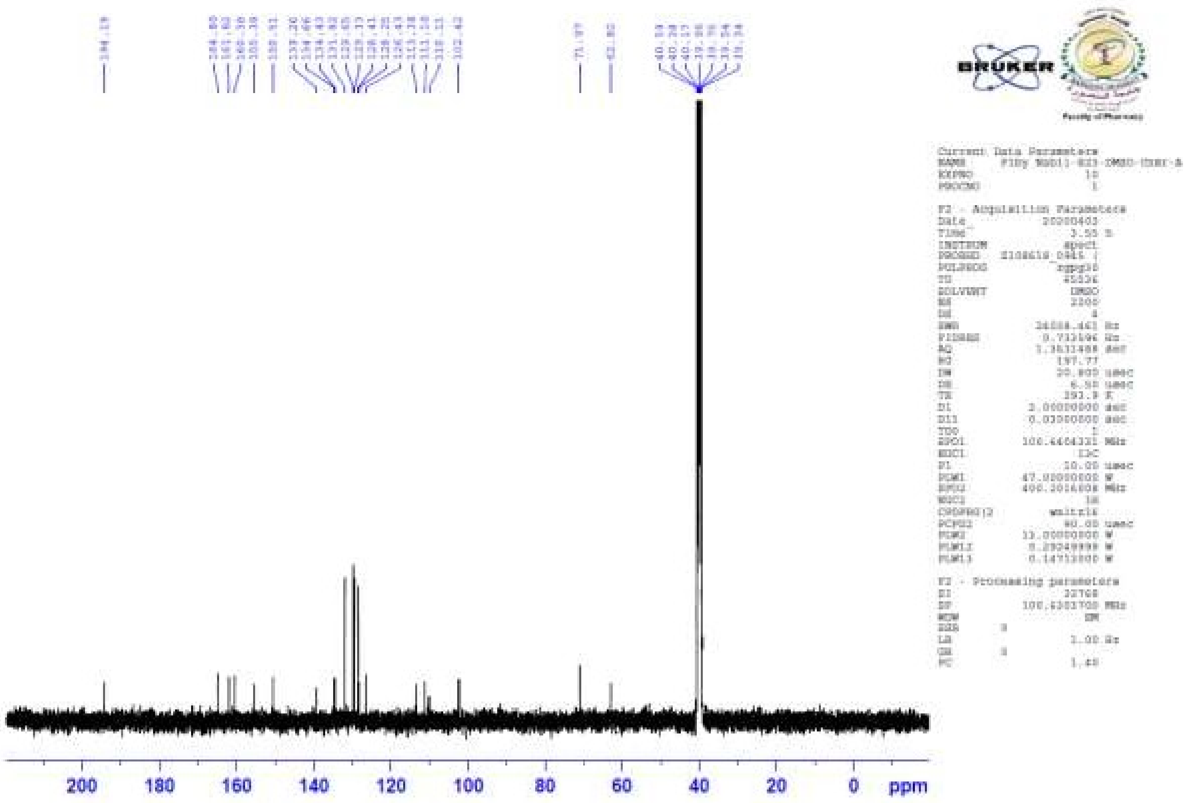
194.19
71.07
62.80
200
180
160
140
120
100
80
60
40
20
0
ppm
Current Data Parameters
F2 - Acquisition Parameters
F2 - Processing parameters

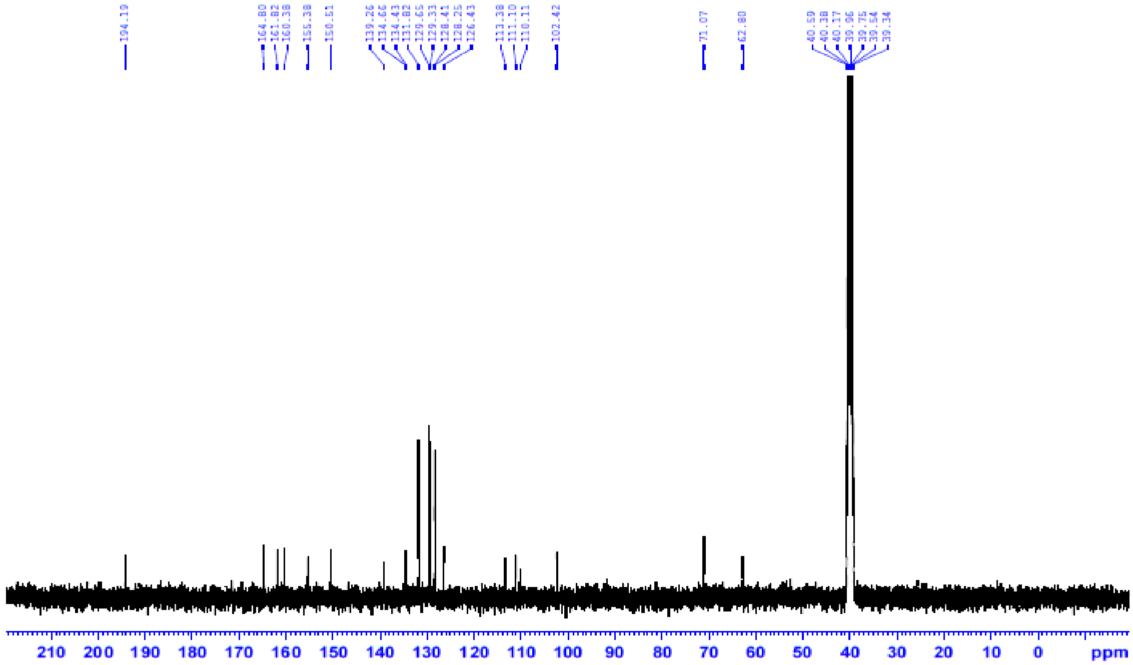
194.19
164.80
161.82
160.38
155.38
150.51
139.26
134.66
134.43
131.82
129.65
129.33
128.41
128.25
126.43
113.38
111.10
110.11
102.42
71.07
62.80
40.59
40.38
40.17
39.96
39.75
39.54
39.34
210 200 190 180 170 160 150 140 130 120 110 100 90 80 70 60 50 40 30 20 10 0 ppm

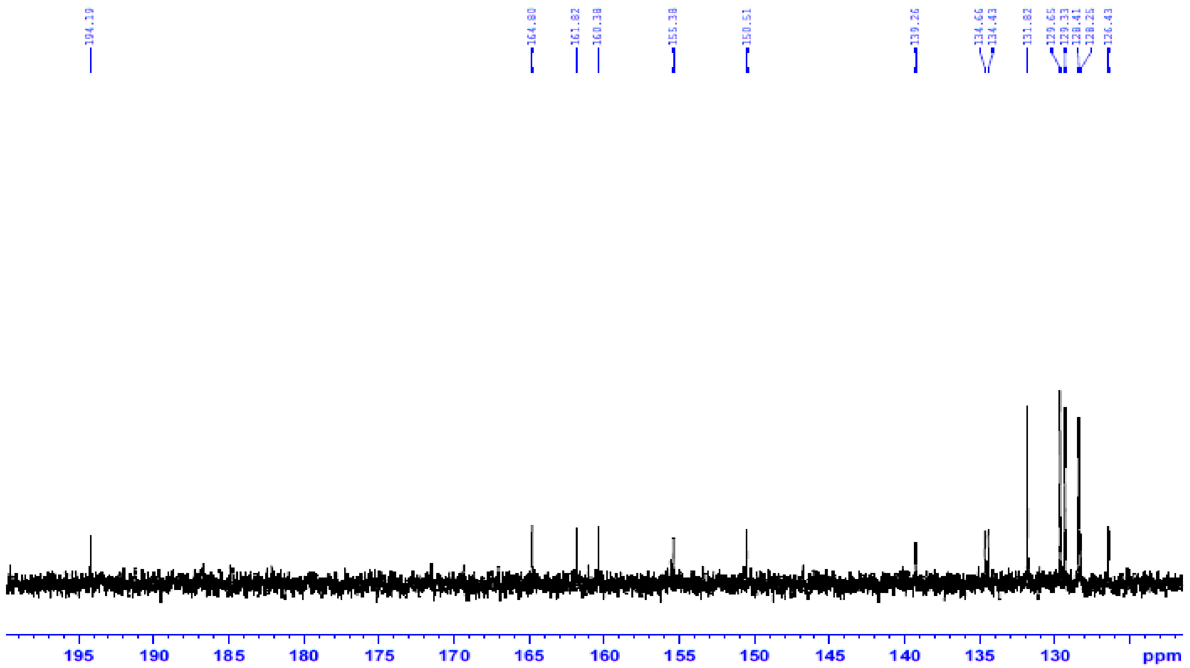
194.19
164.80
161.82
160.38
155.38
150.51
139.26
134.66
134.43
131.82
129.65
129.33
128.41
128.25
126.43
195
190
185
180
175
170
165
160
155
150
145
140
135
130
ppm

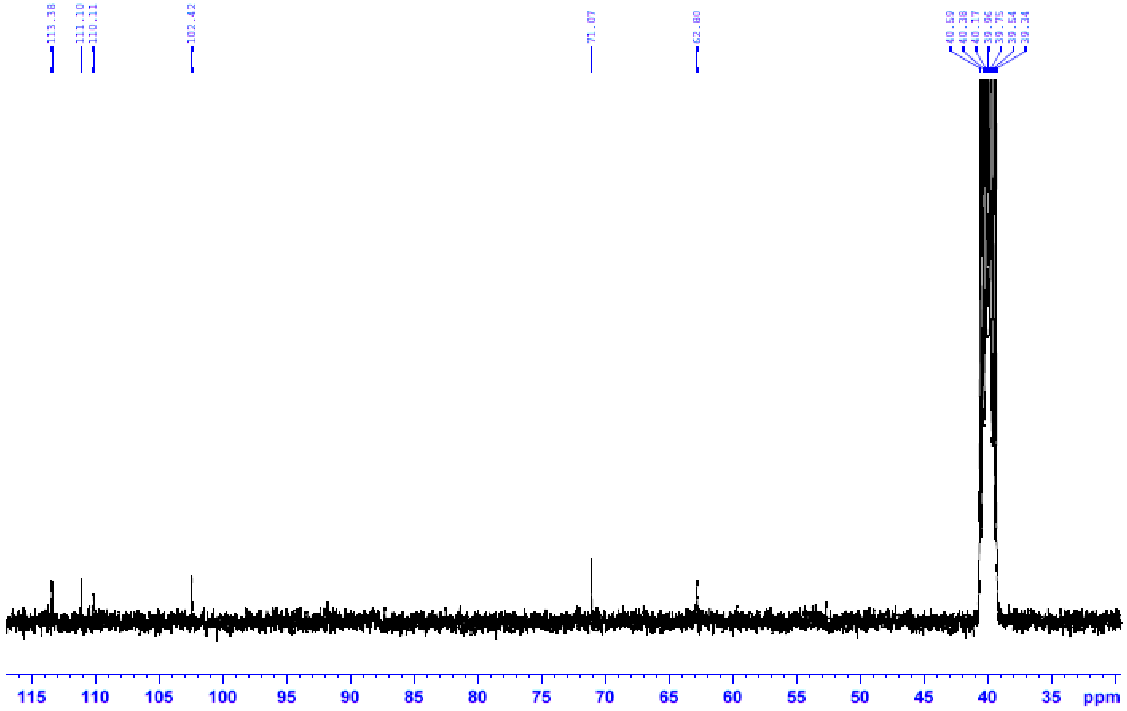
113.38
111.10
110.11
102.42
71.07
62.80
40.59
40.38
40.17
39.96
39.75
39.54
39.34
115
110
105
100
95
90
85
80
75
70
65
60
55
50
45
40
35
ppm

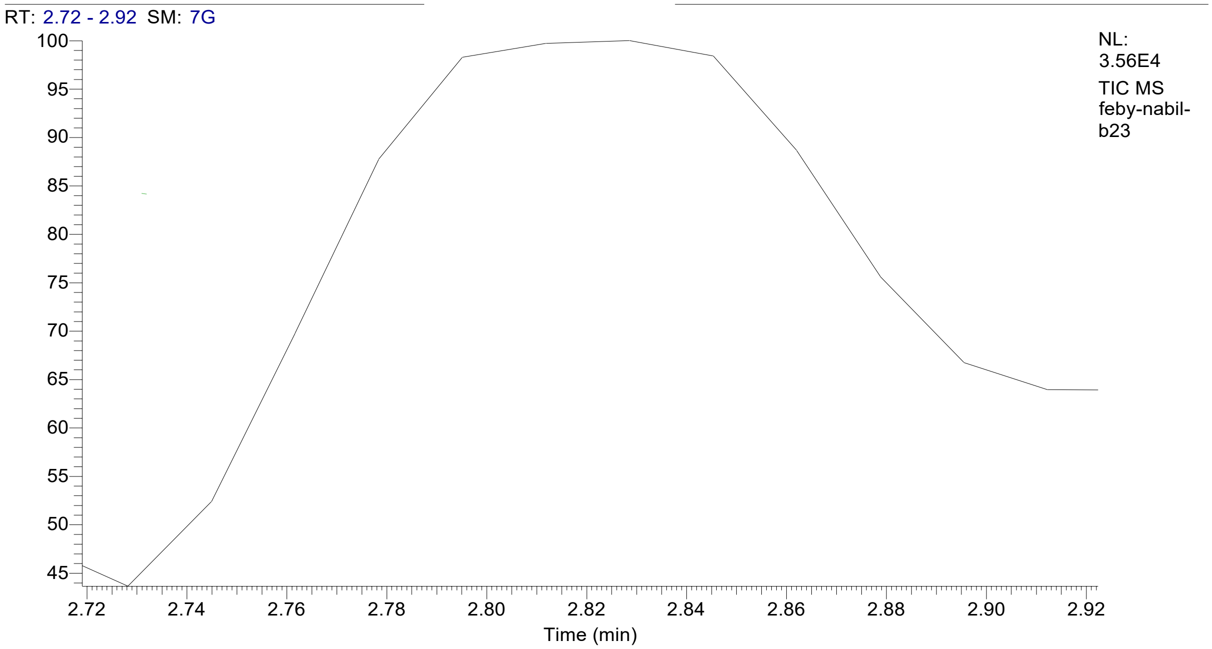
RT: 2.72 - 2.92 SM: 7G
NL: 3.56E4
TIC MS feby-nabil-b23
Time (min)

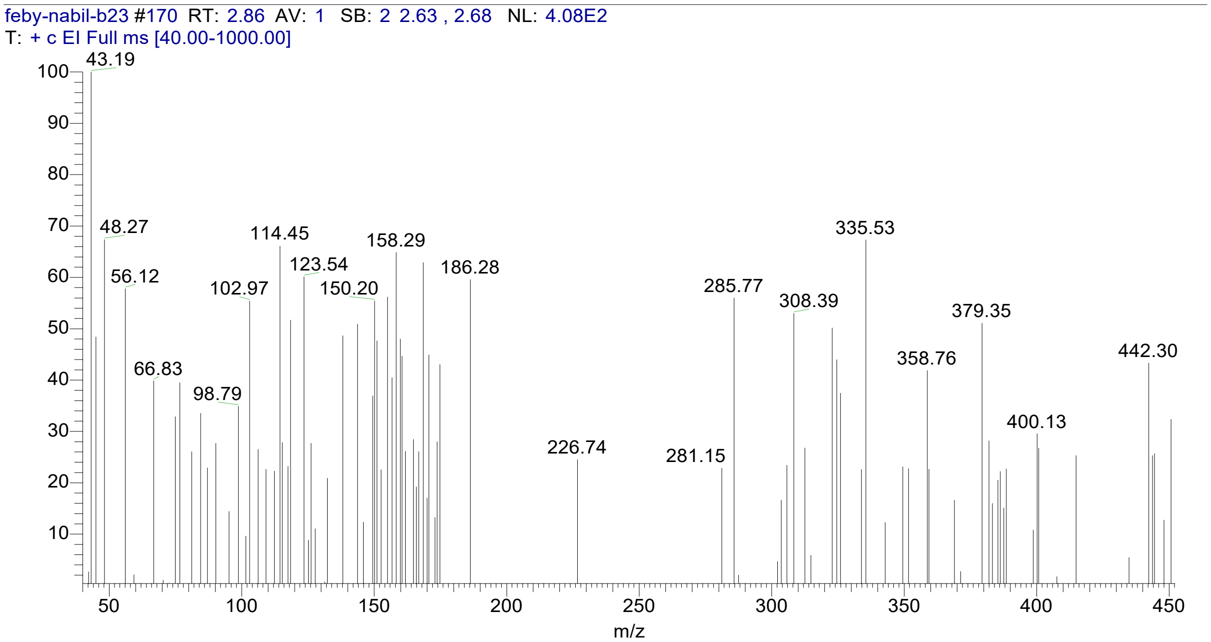
feby-nabil-b23 #170 RT: 2.86 AV: 1 SB: 2 2.63 , 2.68 NL: 4.08E2
T: + c EI Full ms [40.00-1000.00]
43.19
48.27
56.12
66.83
98.79
102.97
114.45
123.54
150.20
158.29
186.28
226.74
281.15
285.77
308.39
335.53
358.76
379.35
400.13
442.30
m/z

Compound 9

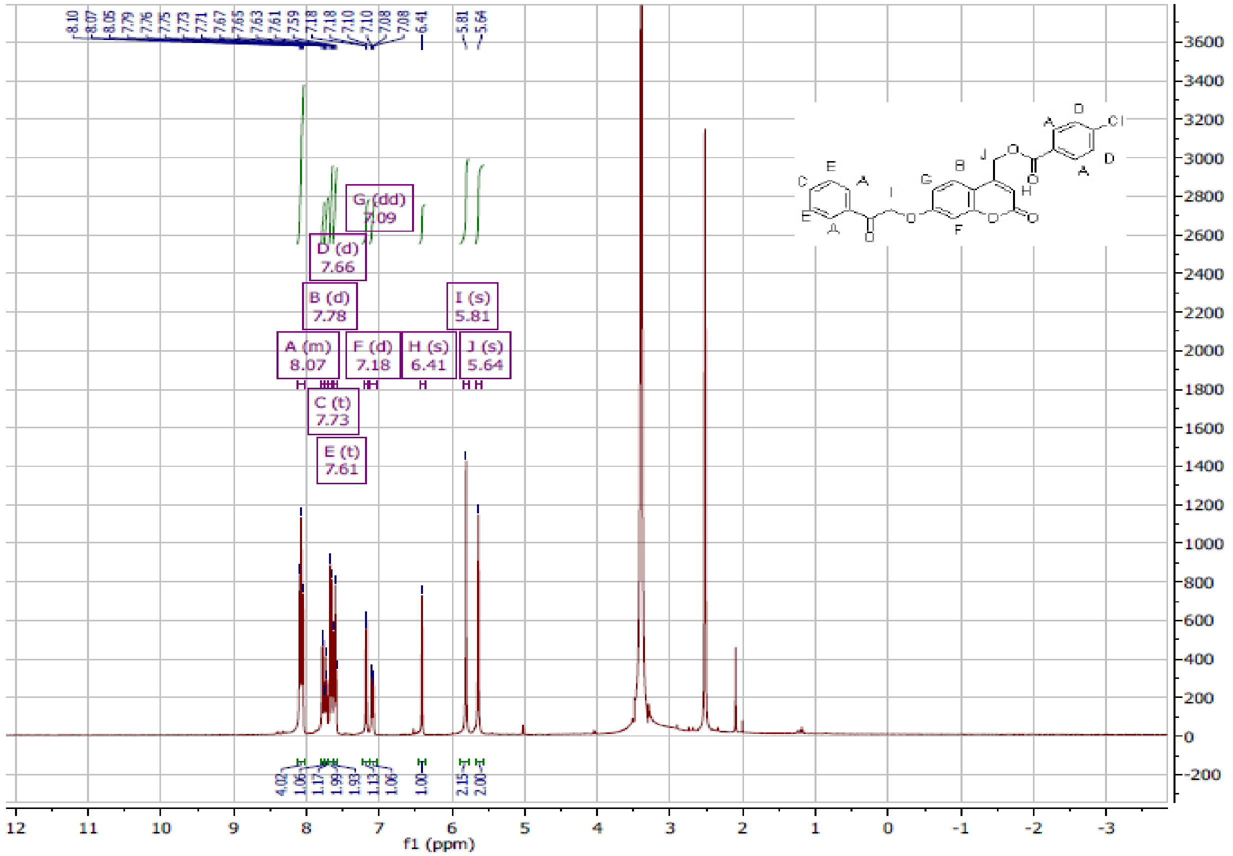

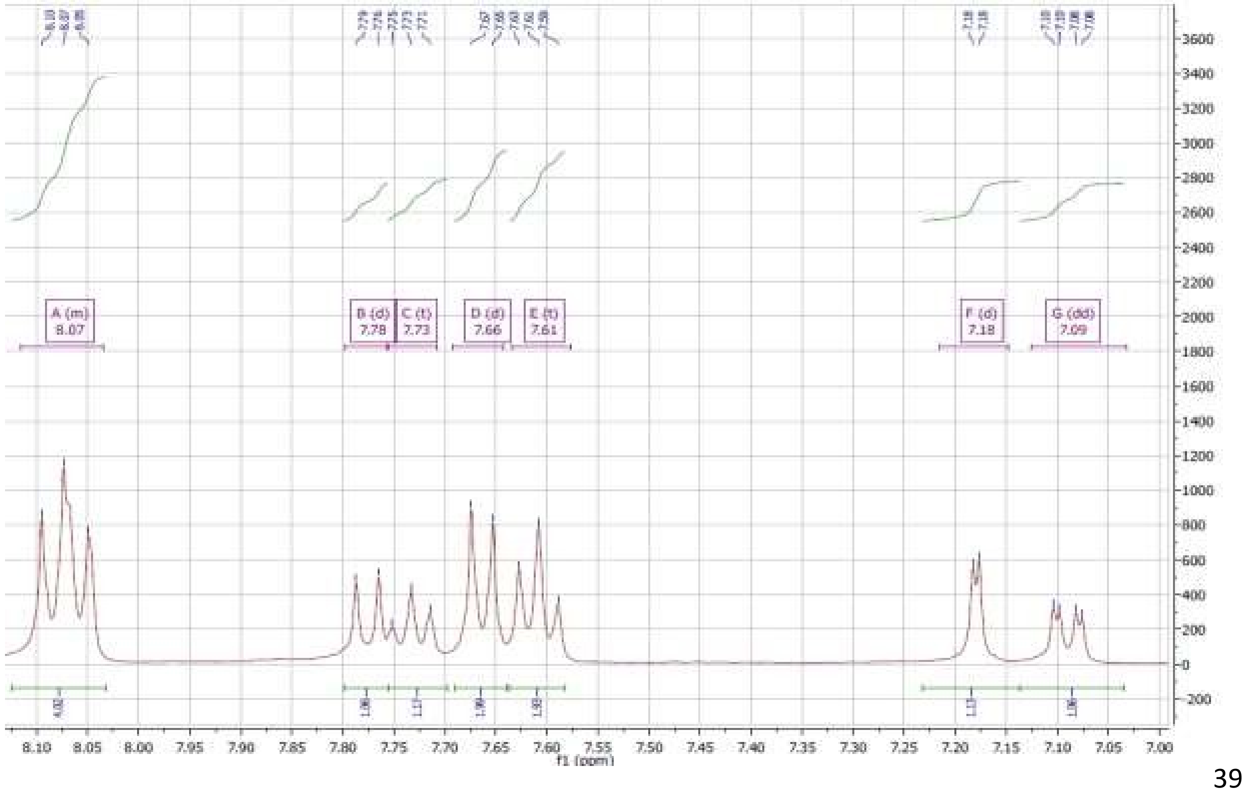

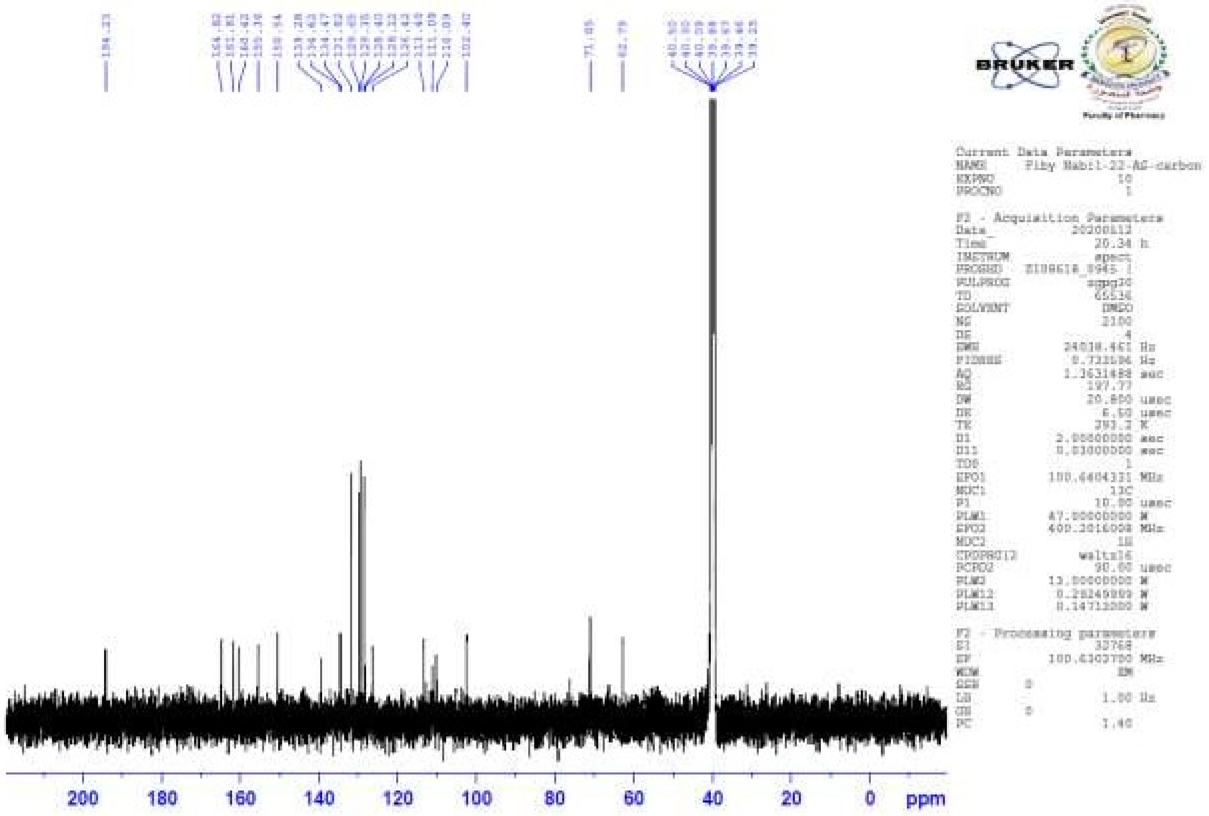
194.23
164.82
161.81
160.42
155.36
150.54
139.28
134.62
134.47
131.82
129.65
129.35
128.40
128.22
126.42
113.40
111.09
110.09
102.40
71.05
62.79
40.50
40.30
40.09
39.88
39.67
39.46
39.25
BRUKER
Current Data Parameters
F2 - Acquisition Parameters
F2 - Processing parameters
200
180
160
140
120
100
80
60
40
20
0
ppm

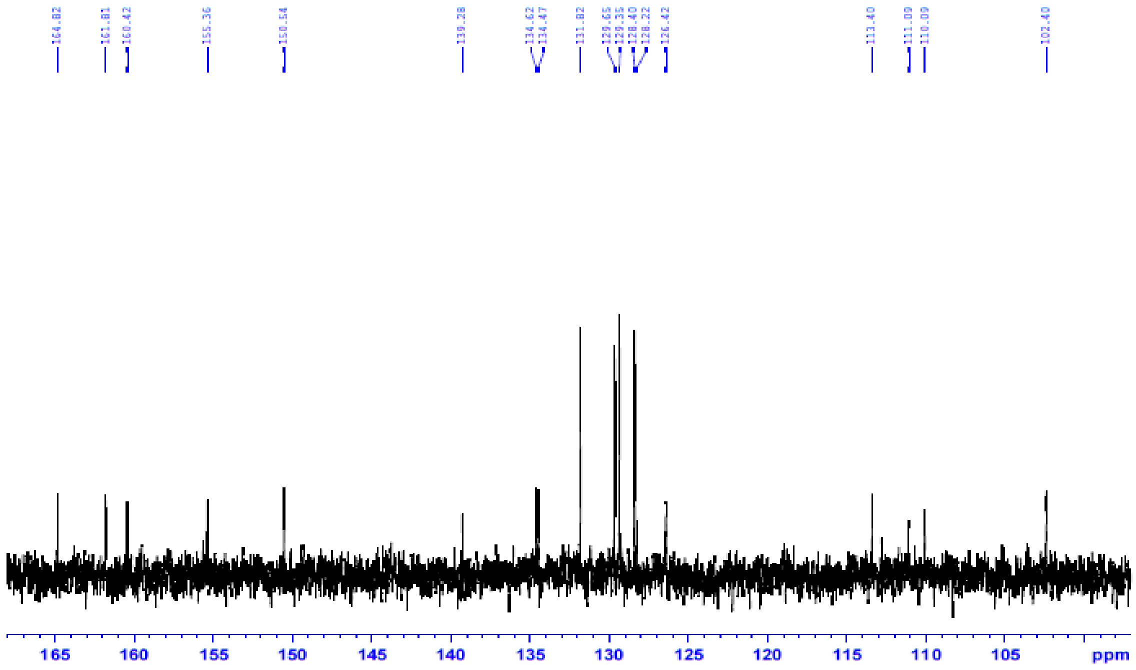
164.82
161.81
160.42
155.36
150.54
139.28
134.62
134.47
131.82
129.65
129.35
128.40
128.22
126.42
113.40
111.09
110.09
102.40
165
160
155
150
145
140
135
130
125
120
115
110
105
ppm

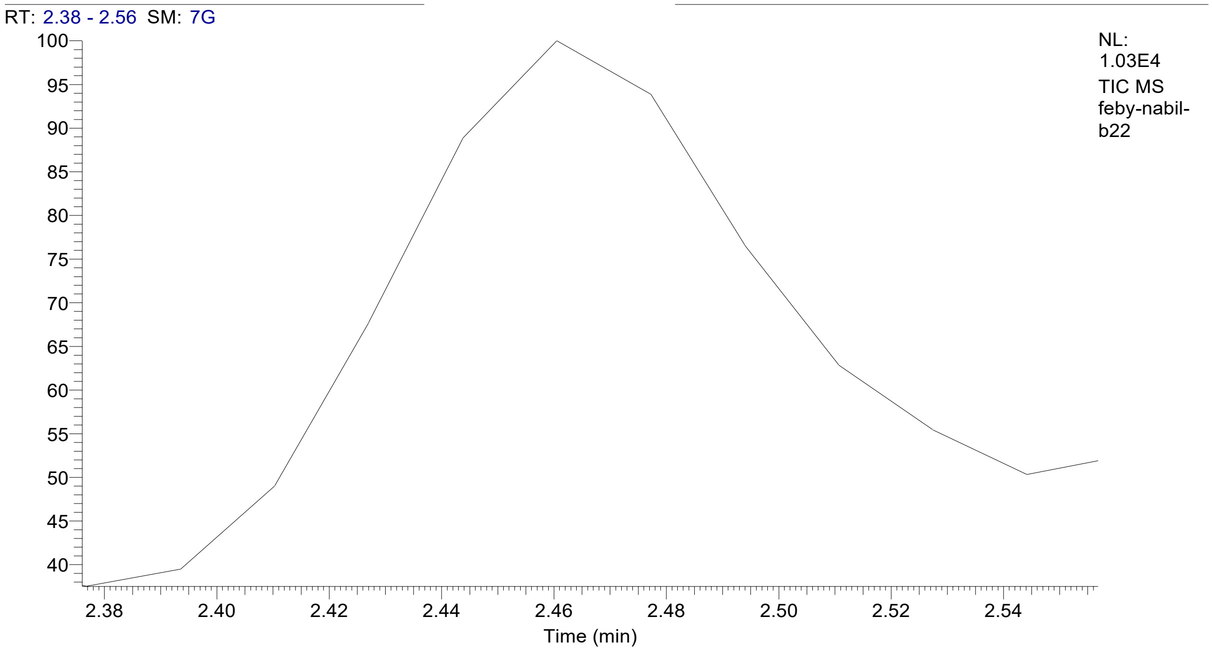
RT: 2.38 - 2.56 SM: 7G
NL: 1.03E4
TIC MS feby-nabil-b22
Time (min)

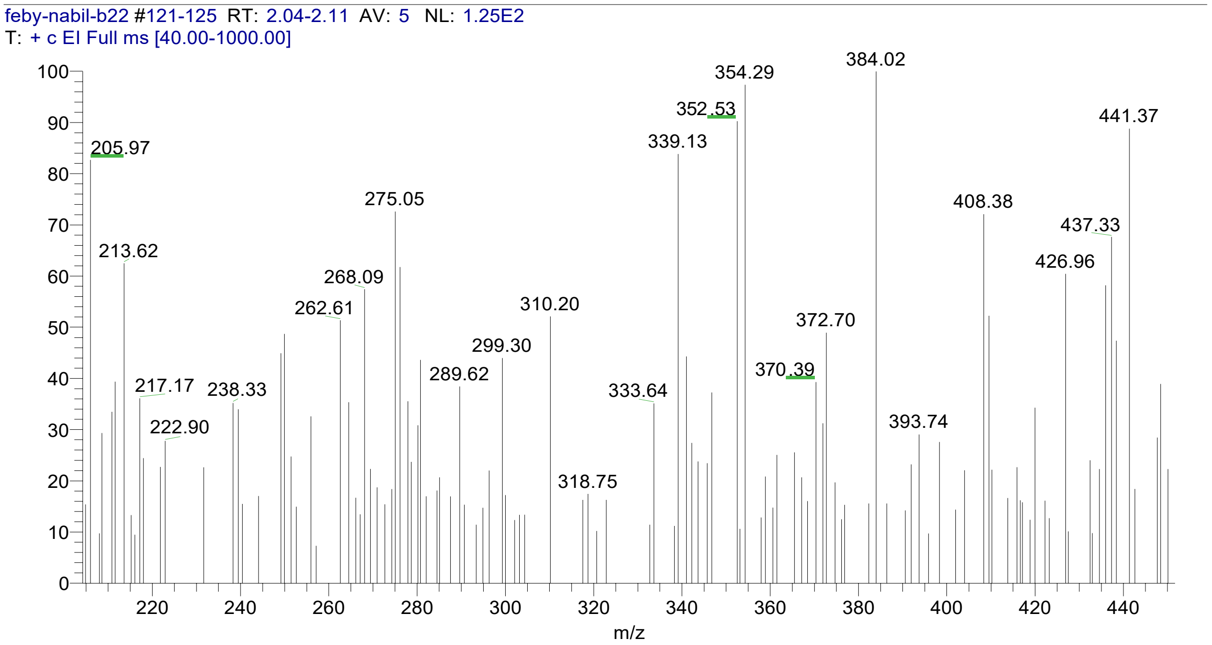
feby-nabil-b22 #121-125 RT: 2.04-2.11 AV: 5 NL: 1.25E2
T: + c EI Full ms [40.00-1000.00]
205.97
213.62
217.17
222.90
238.33
262.61
268.09
275.05
289.62
299.30
310.20
318.75
333.64
339.13
352.53
354.29
370.39
372.70
384.02
393.74
408.38
426.96
437.33
441.37
m/z

Compound 10

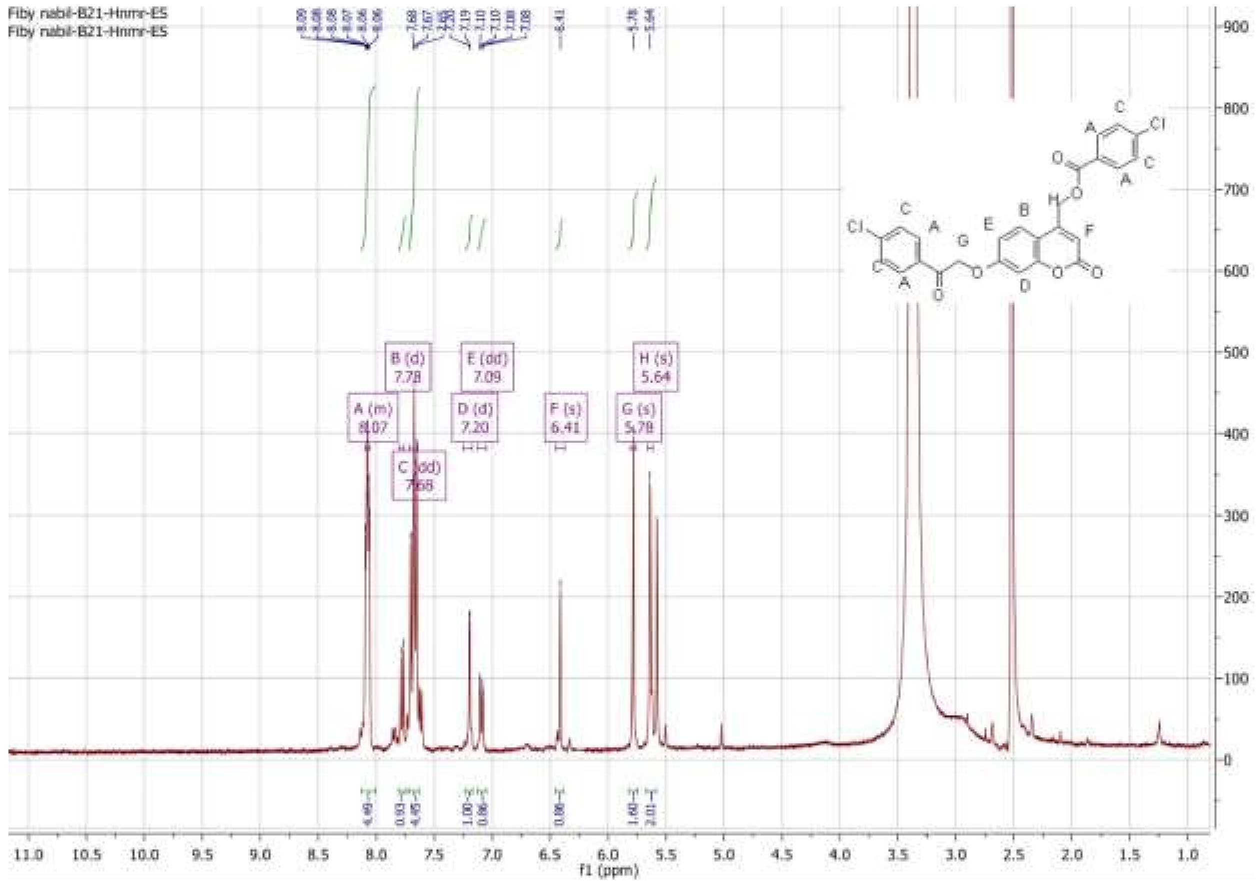

a

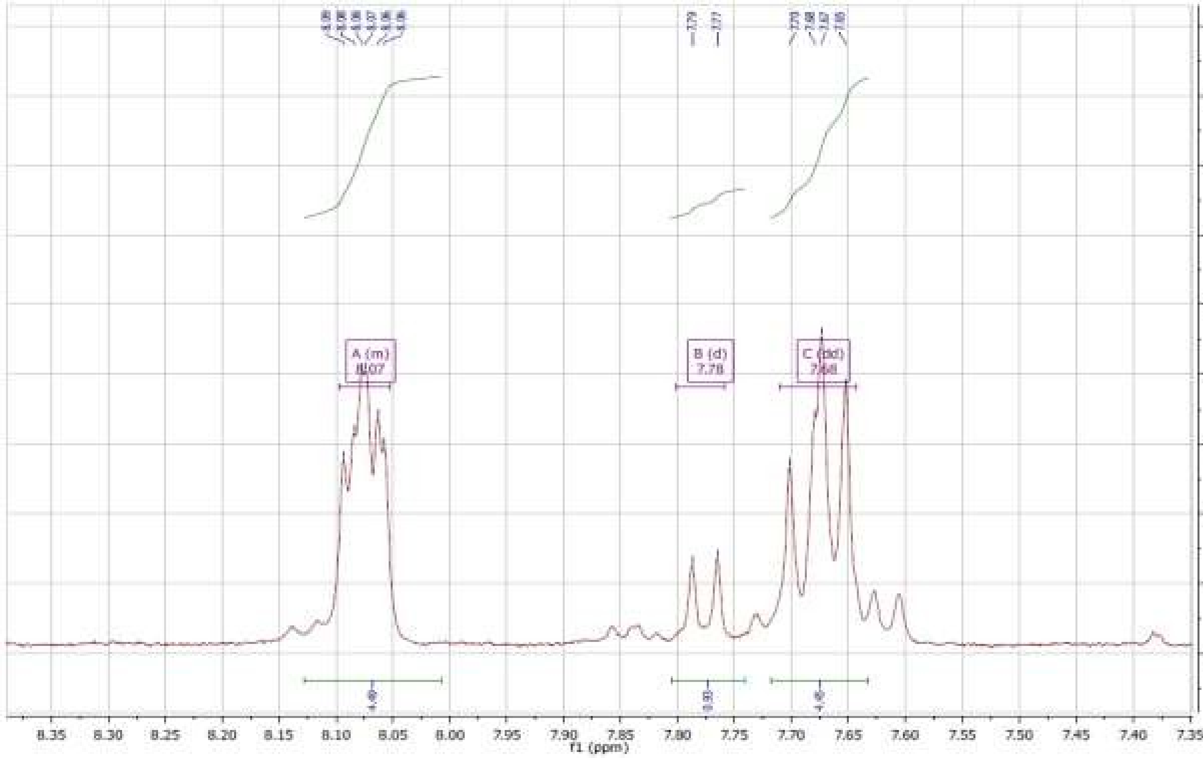

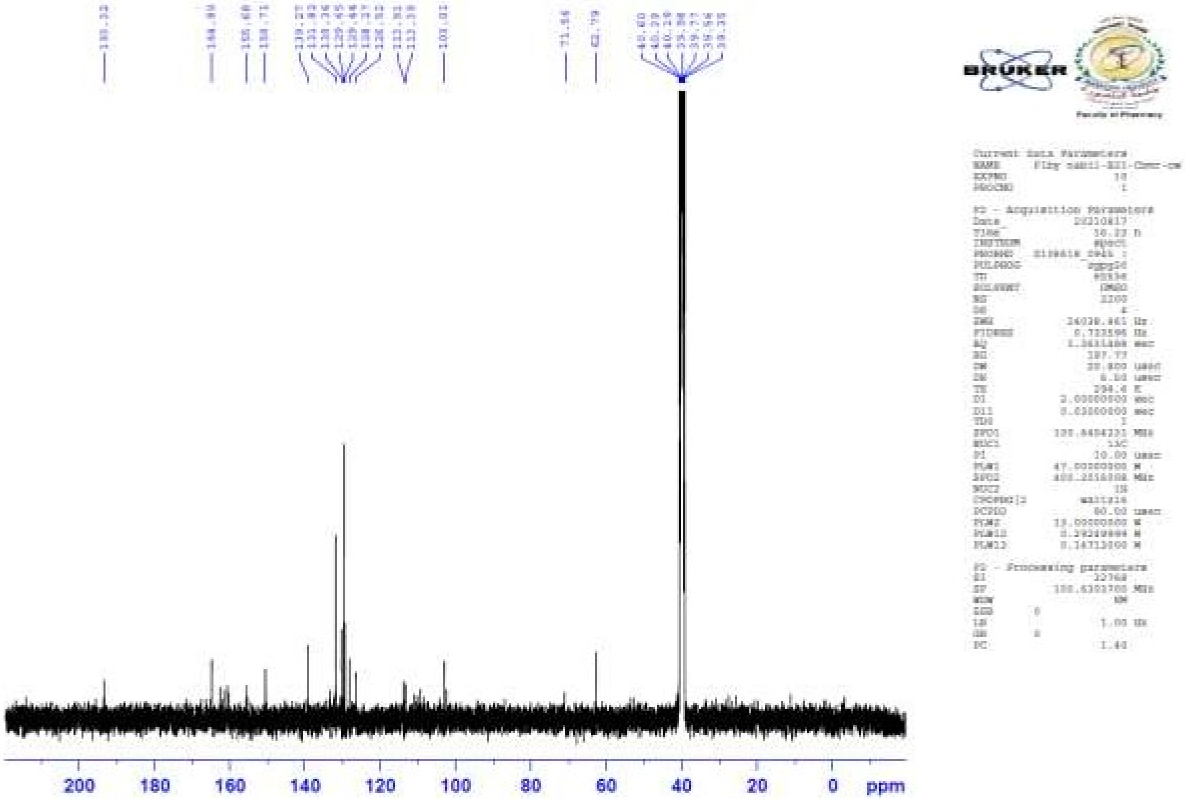

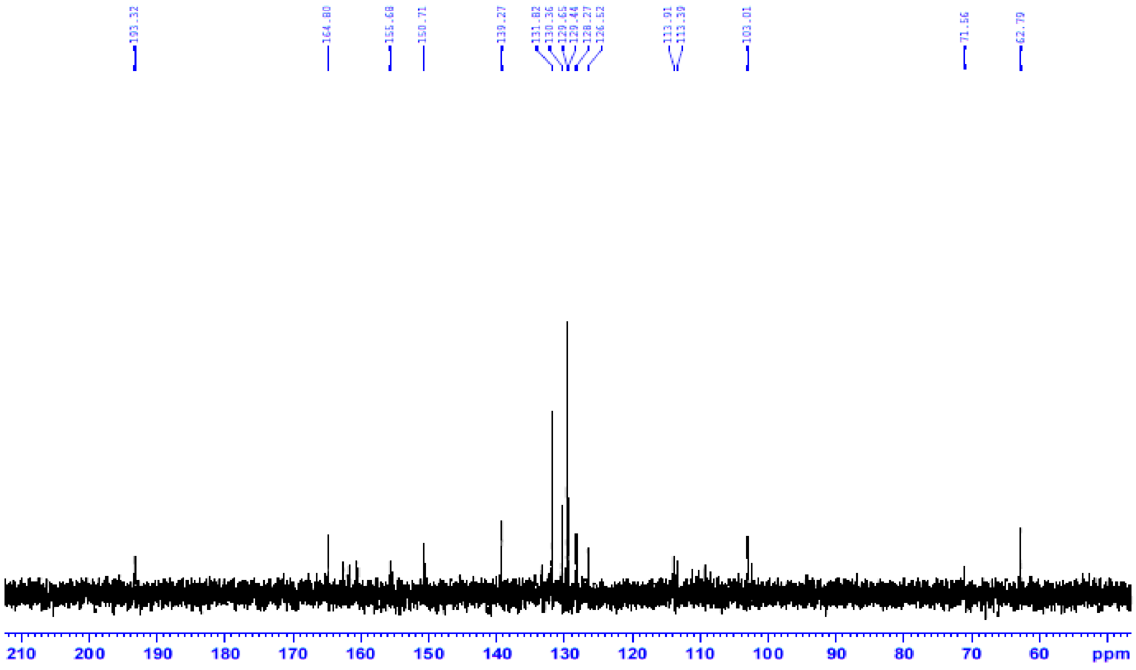
193.32
164.80
155.68
150.71
139.27
131.82
130.36
129.65
129.44
128.27
126.52
113.91
113.39
103.01
71.56
62.79
ppm

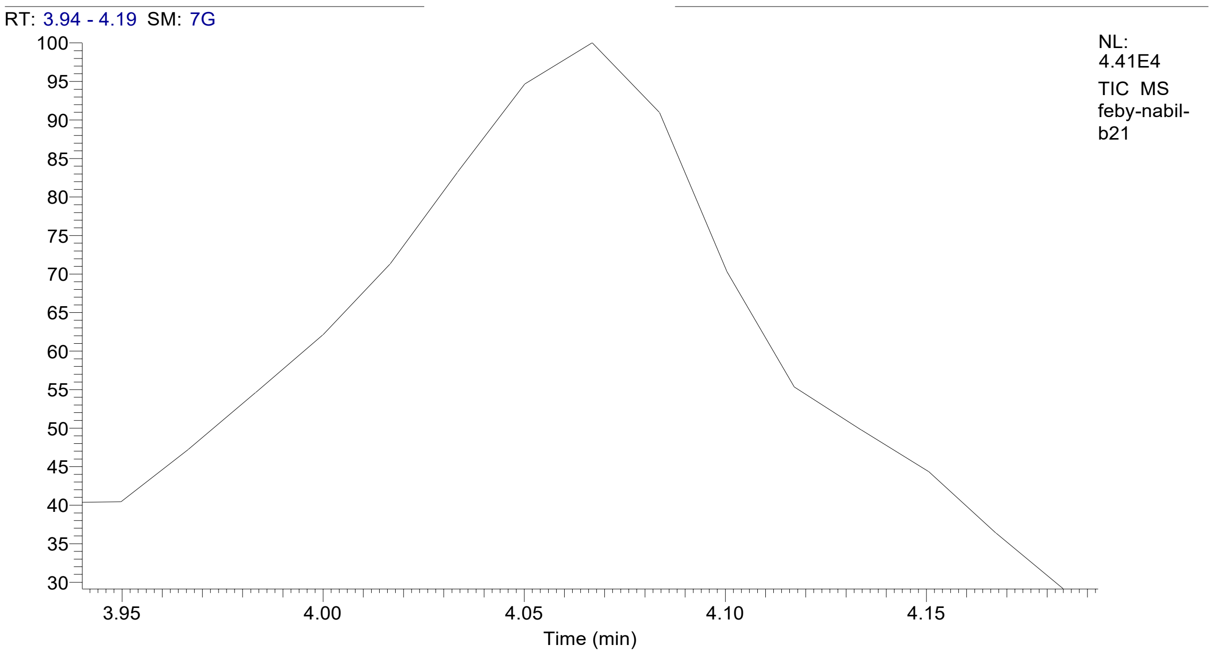
RT: 3.94 - 4.19 SM: 7G
NL: 4.41E4
TIC MS feby-nabil-b21
Time (min)

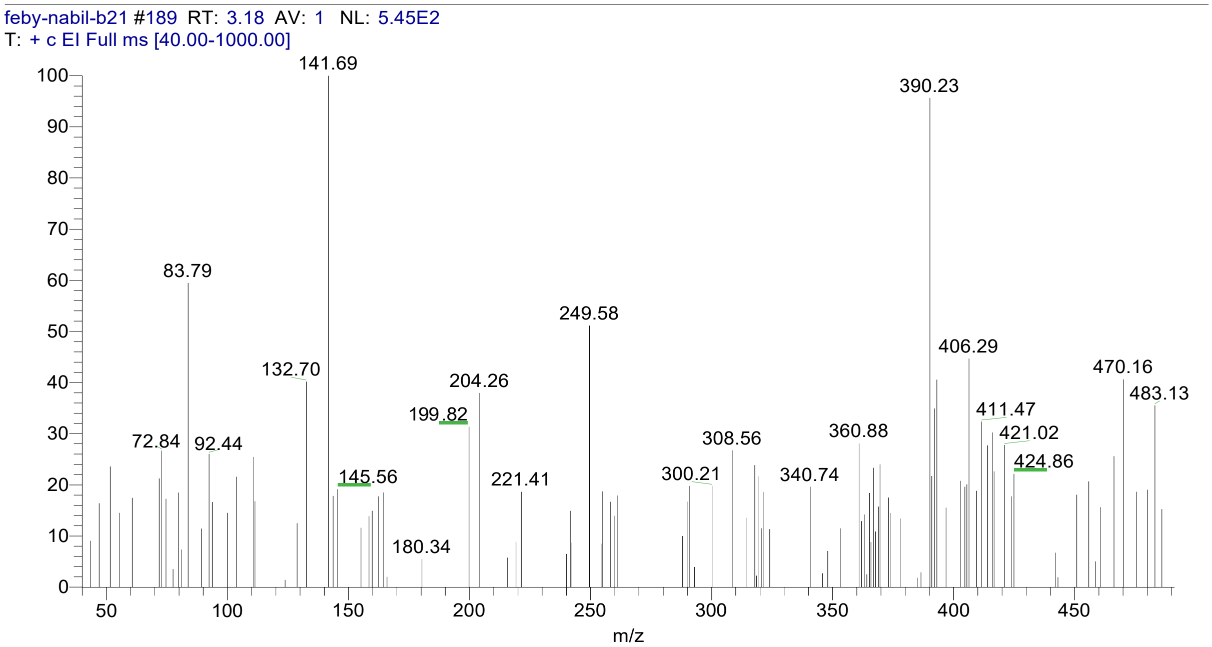
feby-nabil-b21 #189 RT: 3.18 AV: 1 NL: 5.45E2
T: + c EI Full ms [40.00-1000.00]
141.69
390.23
83.79
249.58
406.29
470.16
483.13
132.70
204.26
199.82
411.47
421.02
72.84
92.44
308.56
360.88
424.86
145.56
221.41
300.21
340.74
180.34
m/z

Compound 11

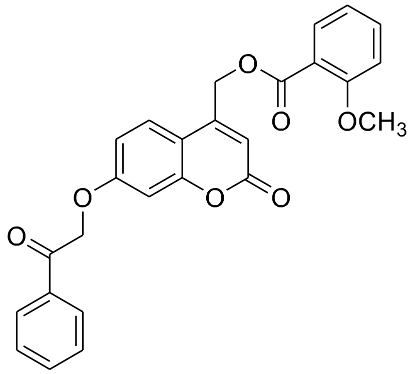
OCH3

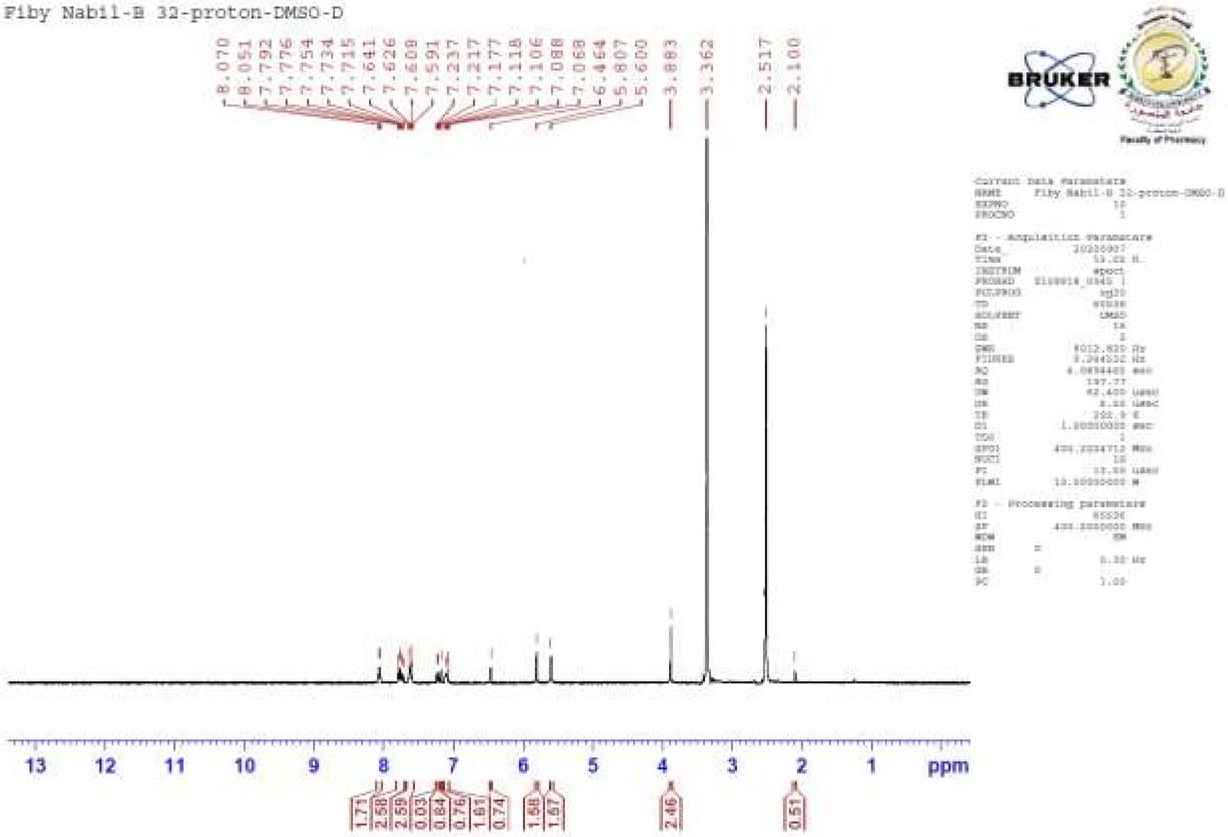
Fiby Nabil-B 32-proton-DMSO-D
8.070
8.051
7.792
7.776
7.754
7.734
7.715
7.641
7.626
7.609
7.591
7.237
7.217
7.177
7.118
7.106
7.088
7.068
6.464
5.807
5.600
3.883
3.362
2.517
2.100
BRUKER
Faculty of Pharmacy
13 12 11 10 9 8 7 6 5 4 3 2 1 ppm
1.71
2.58
2.59
0.03
0.84
0.76
1.61
0.74
1.68
1.67
2.46
0.51

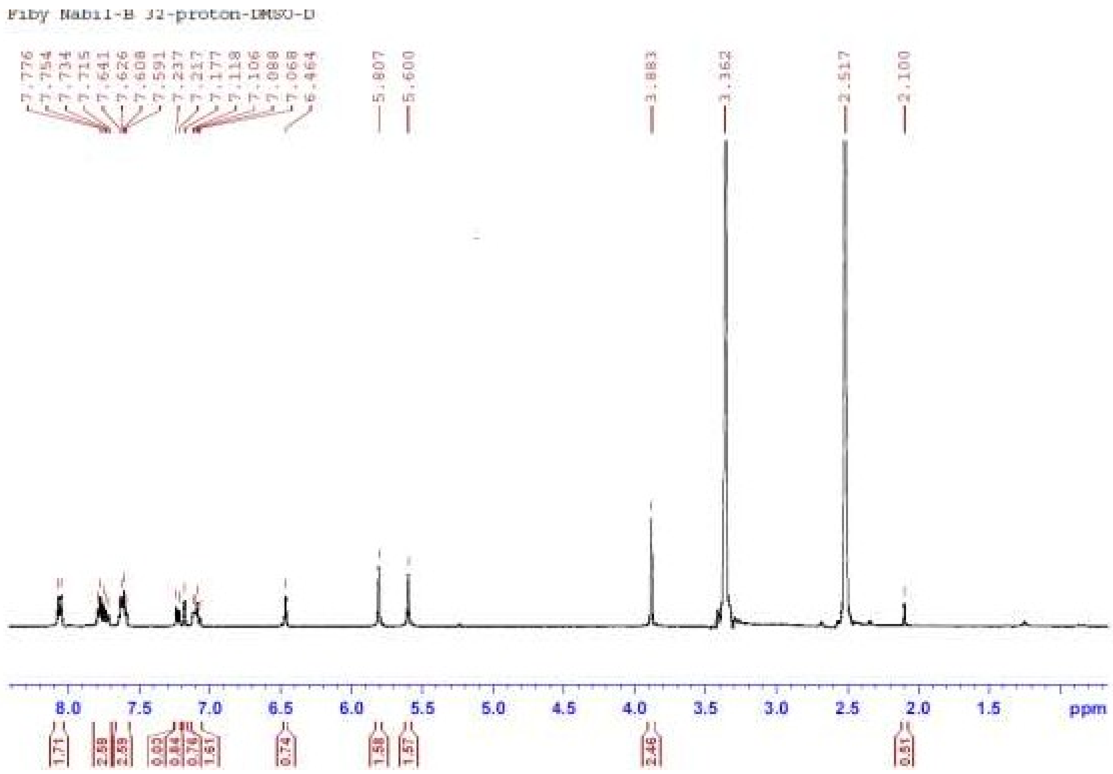
Fiby Nabil-B 32-proton-DMSO-D
7.776
7.754
7.734
7.715
7.641
7.626
7.608
7.591
7.237
7.217
7.177
7.118
7.106
7.088
7.068
6.464
5.807
5.600
3.883
3.362
2.517
2.100
8.0
7.5
7.0
6.5
6.0
5.5
5.0
4.5
4.0
3.5
3.0
2.5
2.0
1.5
ppm
1.71
2.58
2.59
1.58
1.57
0.74
2.46
0.51

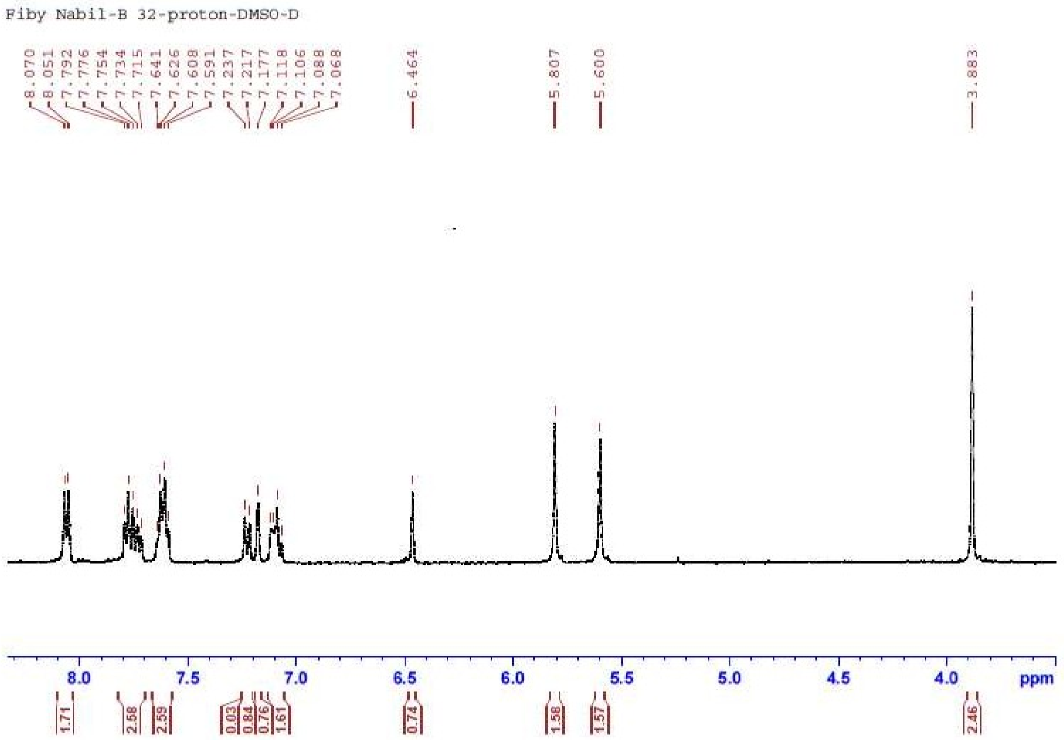
Fiby Nabil-B 32-proton-DMSO-D
8.070
8.051
7.792
7.776
7.754
7.734
7.715
7.641
7.626
7.608
7.591
7.237
7.217
7.177
7.118
7.106
7.088
7.068
6.464
5.807
5.600
3.883
8.0
7.5
7.0
6.5
6.0
5.5
5.0
4.5
4.0
ppm
1.71
2.58
2.59
0.03
0.84
0.76
1.61
0.74
1.58
1.57
2.46

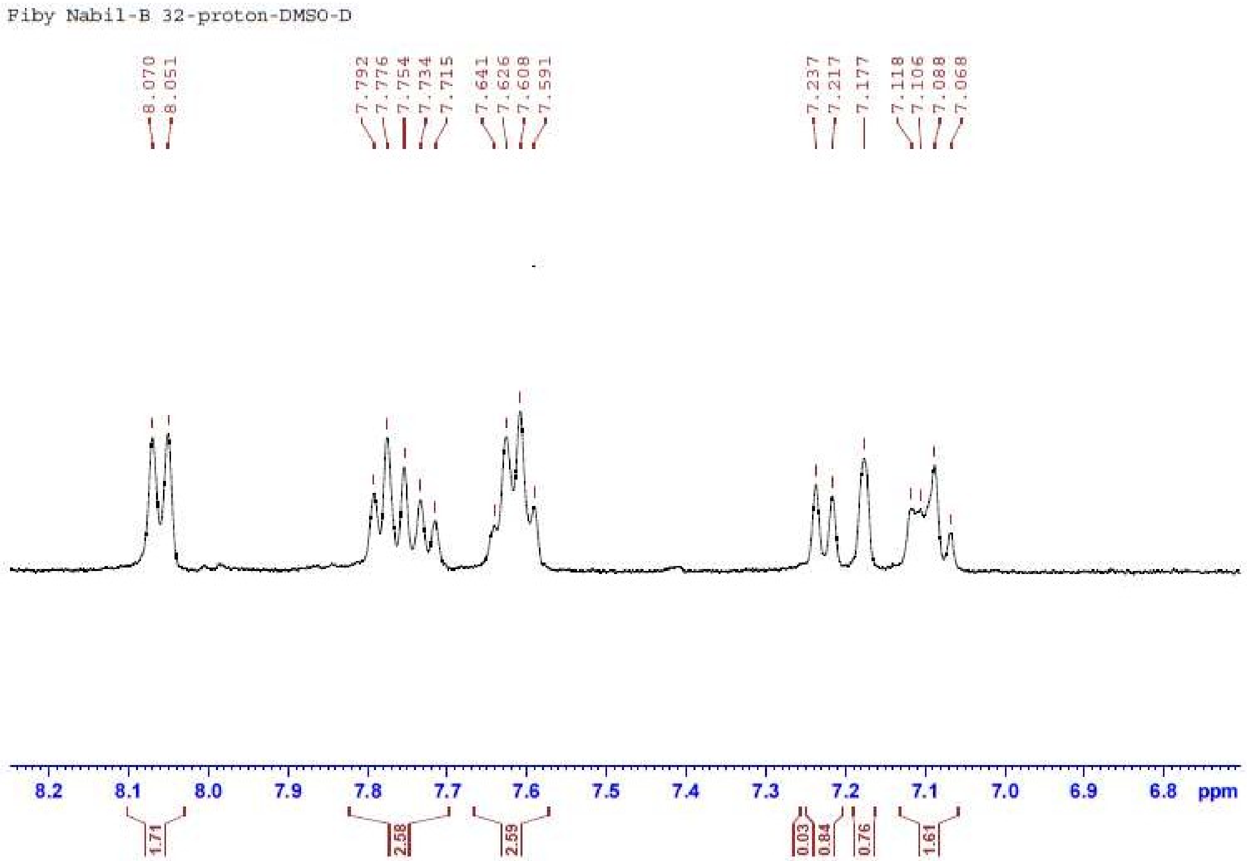
Fiby Nabil-B 32-proton-DMSO-D
8.070
8.051
7.792
7.776
7.754
7.734
7.715
7.641
7.626
7.608
7.591
7.237
7.217
7.177
7.118
7.106
7.088
7.068
8.2
8.1
8.0
7.9
7.8
7.7
7.6
7.5
7.4
7.3
7.2
7.1
7.0
6.9
6.8
ppm
1.71
2.58
2.59
0.03
0.84
0.76
1.61

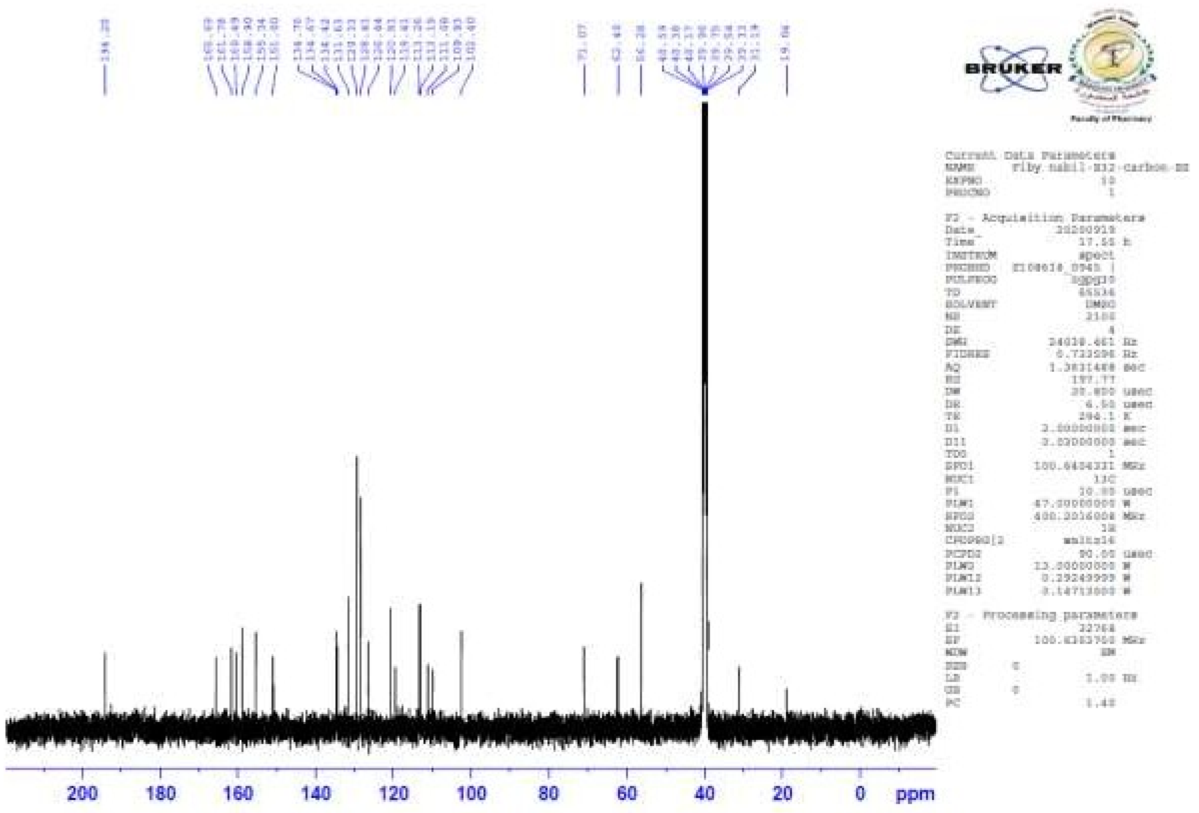
BRUKER
200
180
160
140
120
100
80
60
40
20
0
ppm

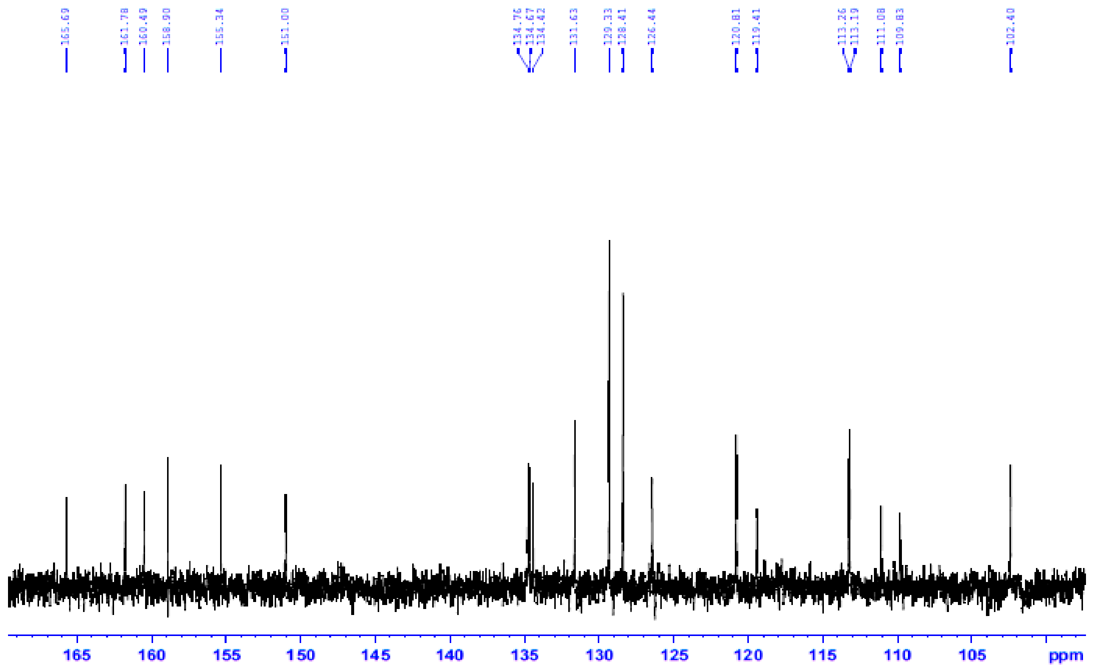
165.69
161.78
160.49
158.90
155.34
151.00
134.76
134.67
134.42
131.63
129.33
128.41
126.44
120.81
119.41
113.26
113.19
111.08
109.83
102.40
165
160
155
150
145
140
135
130
125
120
115
110
105
ppm

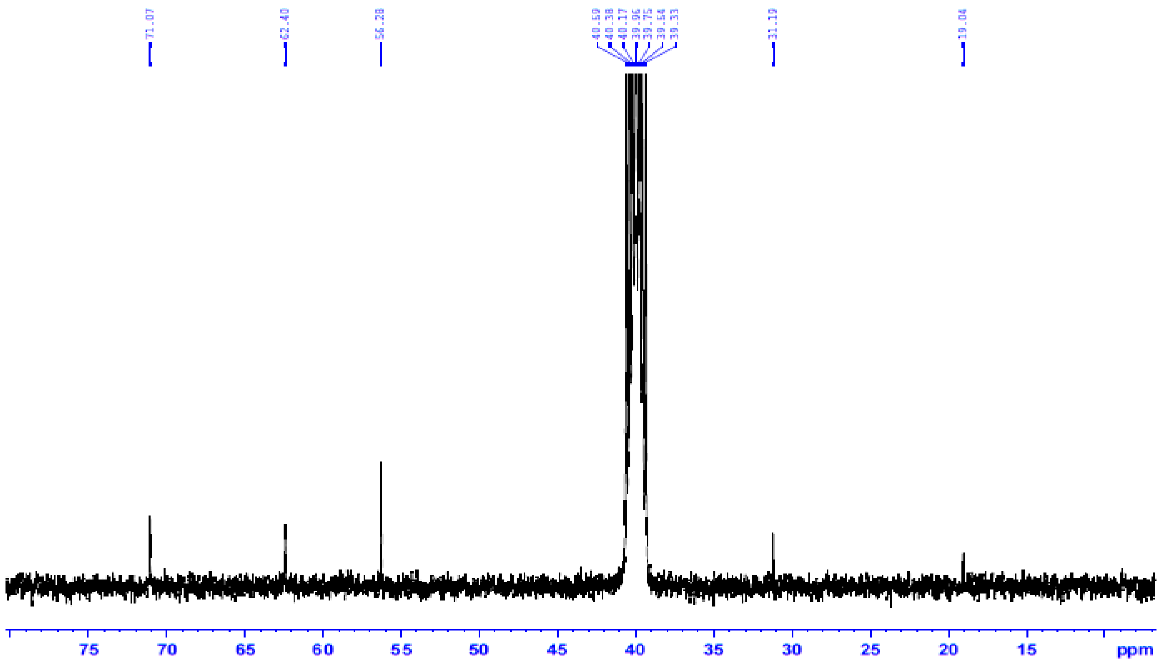
71.07
62.40
56.28
40.59
40.38
40.17
39.96
39.75
39.54
39.33
31.19
19.04
75
70
65
60
55
50
45
40
35
30
25
20
15
ppm

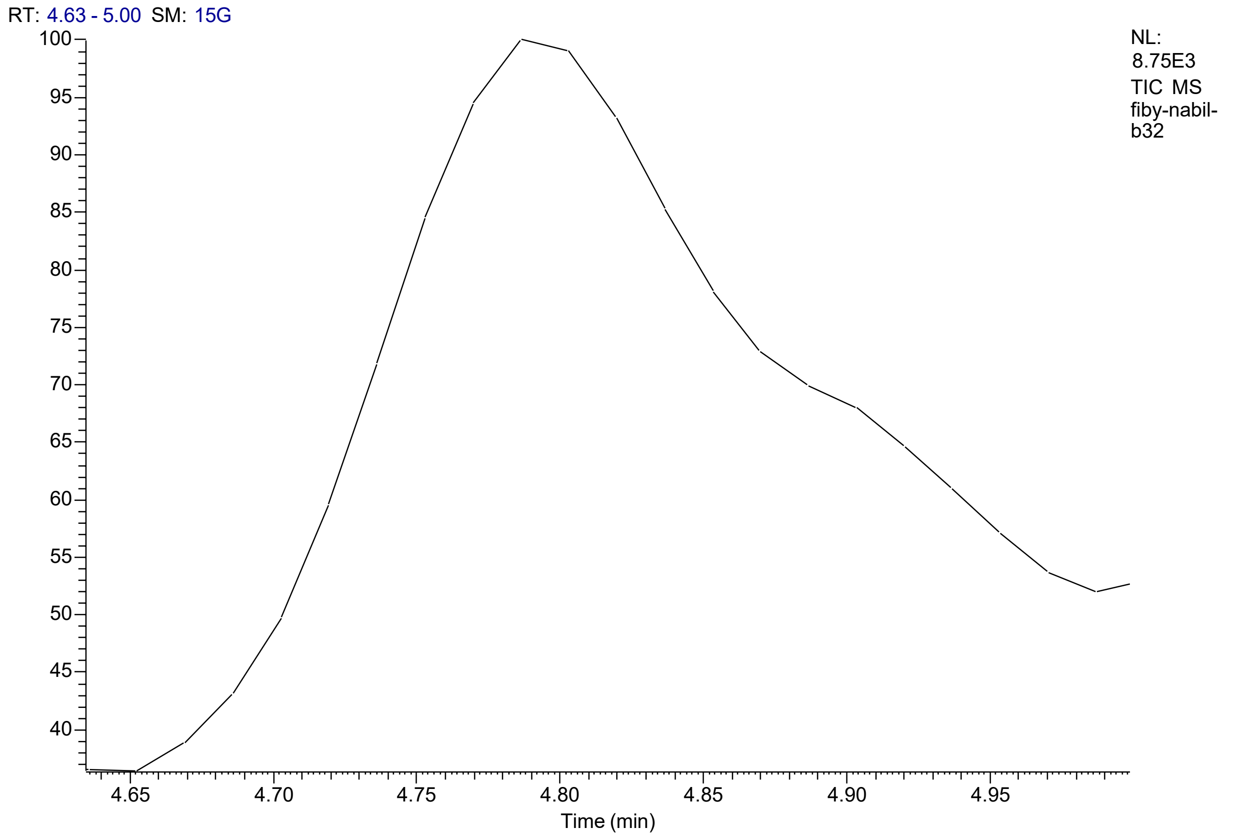
RT: 4.63 - 5.00 SM: 15G
NL: 8.75E3
TIC MS
fiby-nabil-b32
Time (min)

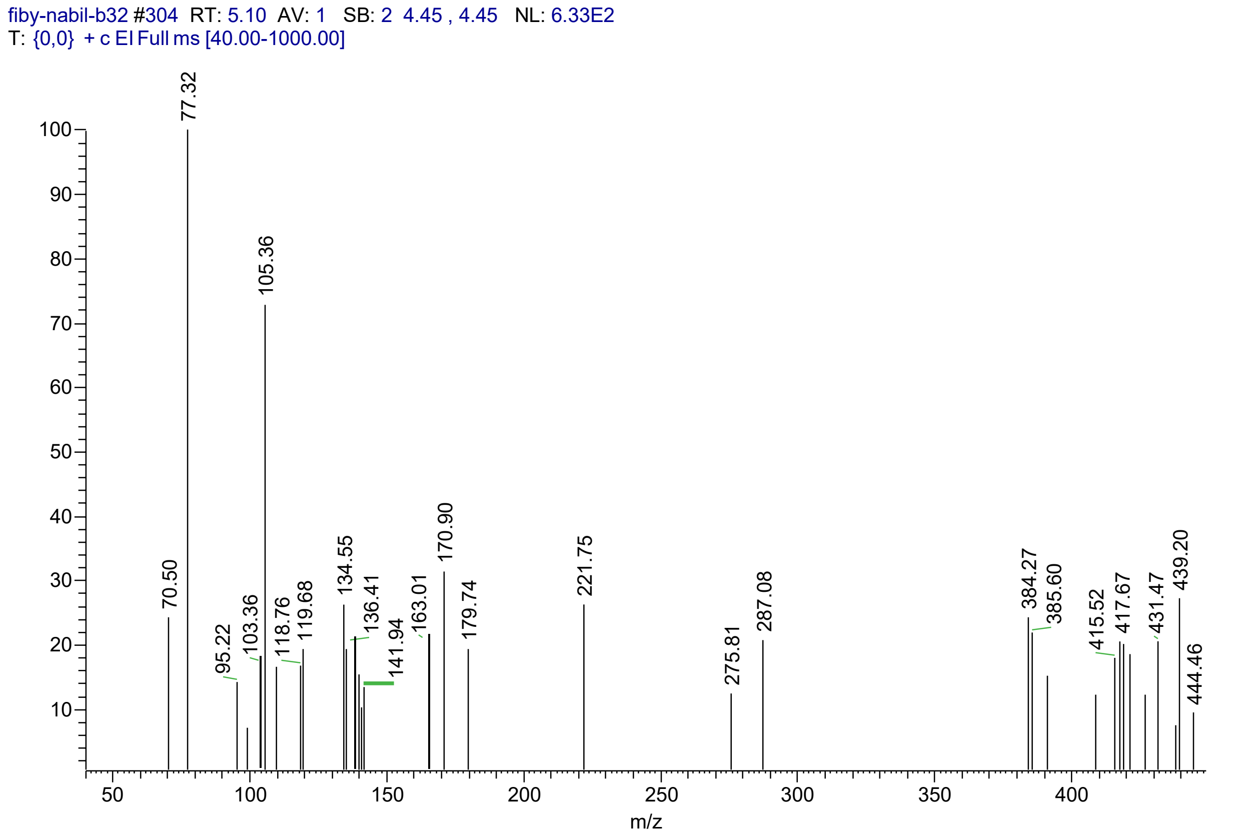
fiby-nabil-b32 #304 RT: 5.10 AV: 1 SB: 2 4.45 , 4.45 NL: 6.33E2
T: {0,0} + c EI Full ms [40.00-1000.00]
77.32
105.36
70.50
95.22
103.36
118.76
119.68
134.55
136.41
141.94
163.01
170.90
179.74
221.75
275.81
287.08
384.27
385.60
415.52
417.67
431.47
439.20
444.46
m/z

Compound 12

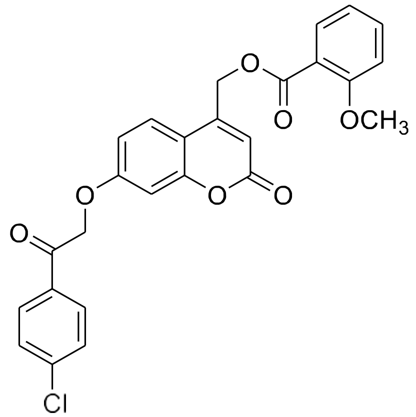

O
O
O
OCH3
O
O
O
Cl

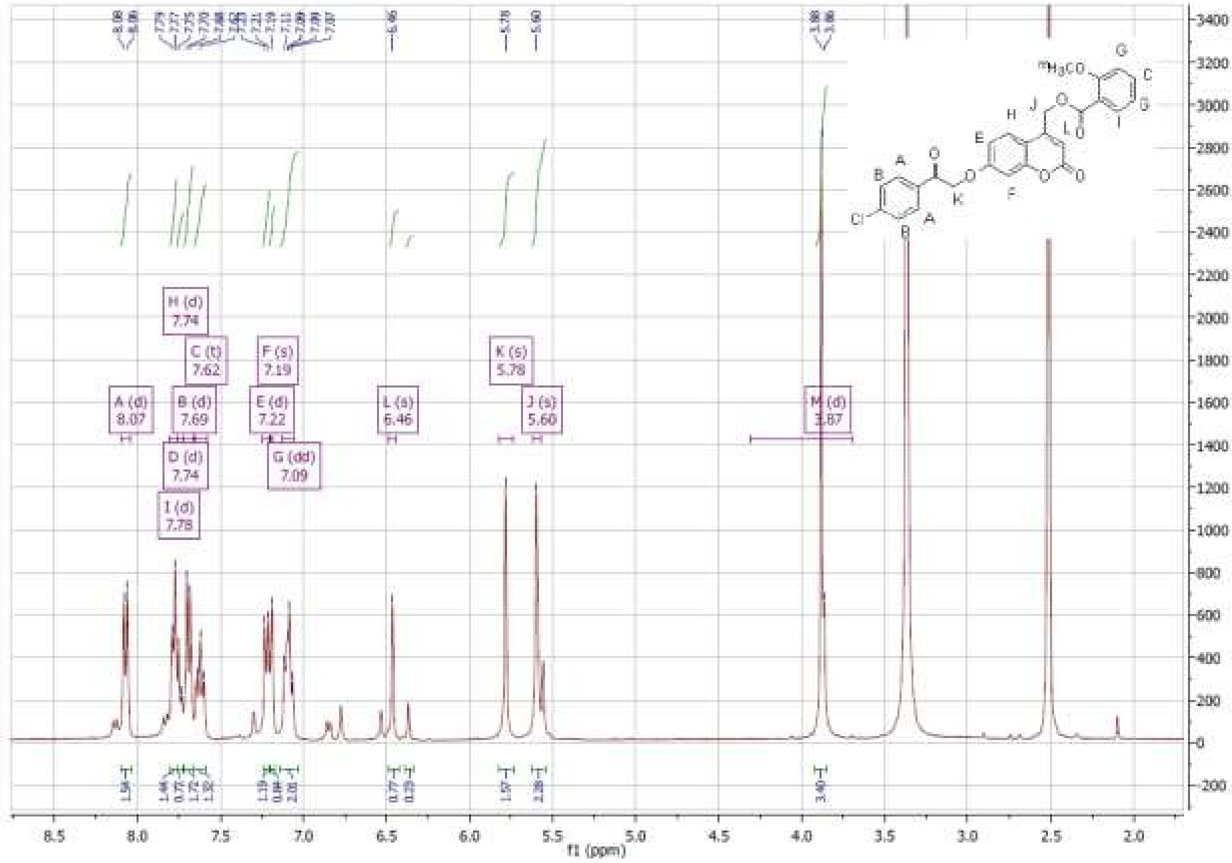

H (d)
7.74
C (t)
7.62
F (s)
7.19
K (s)
5.78
A (d)
8.07
B (d)
7.69
E (d)
7.22
L (s)
6.46
J (s)
5.60
M (d)
3.87
D (d)
7.74
G (dd)
7.09
I (d)
7.78
f1 (ppm)

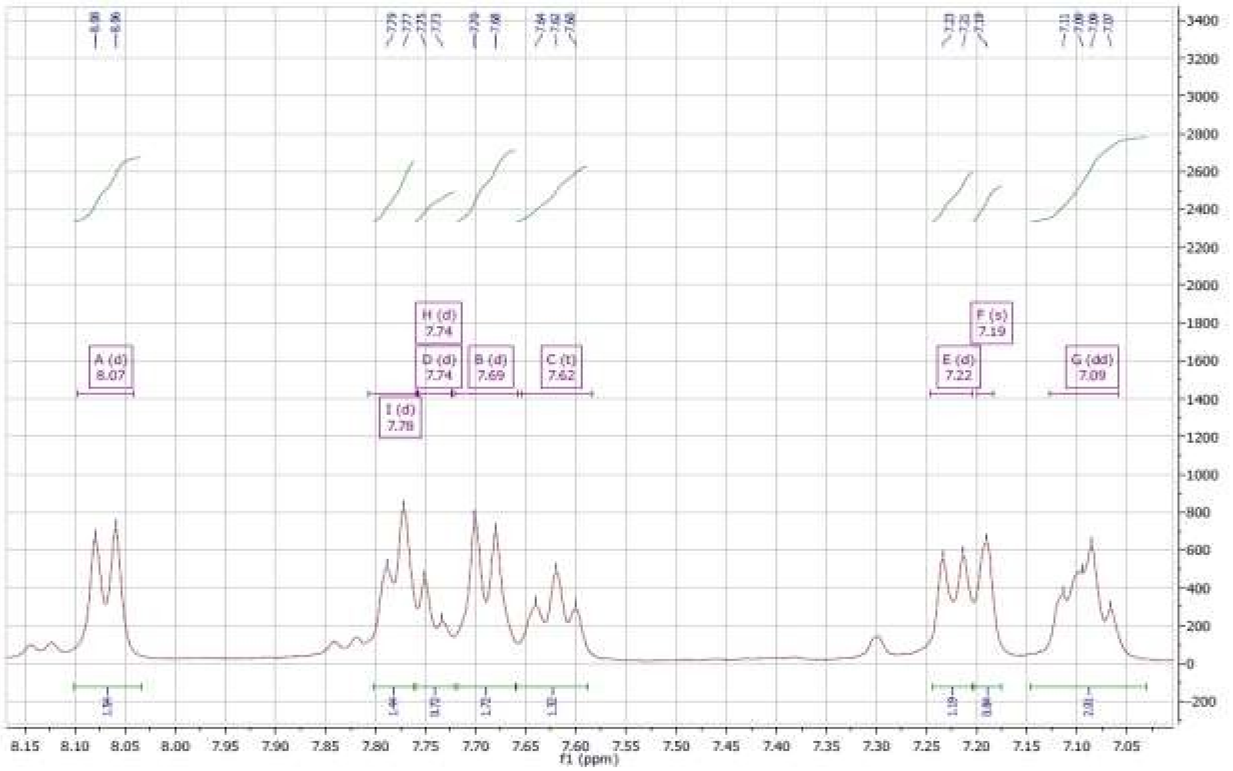

A (d)
8.07
H (d)
7.74
D (d)
7.74
B (d)
7.69
C (t)
7.62
I (d)
7.78
E (d)
7.22
F (s)
7.19
G (dd)
7.09
f1 (ppm)

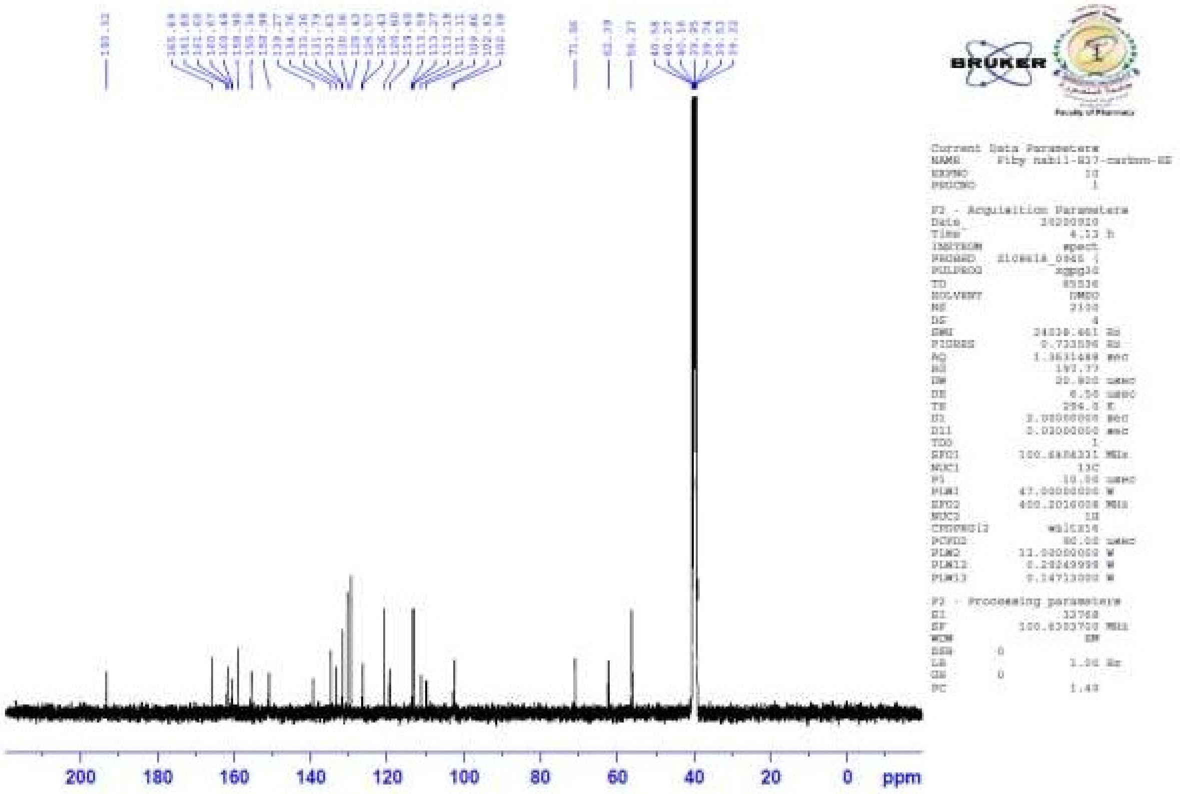

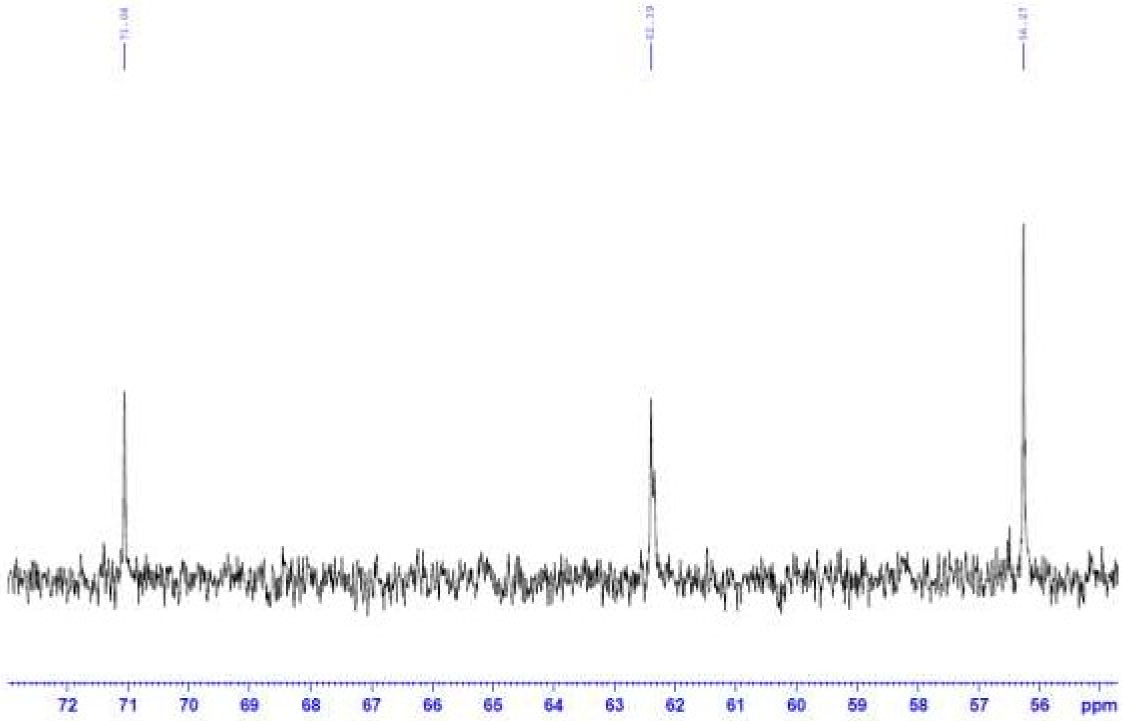

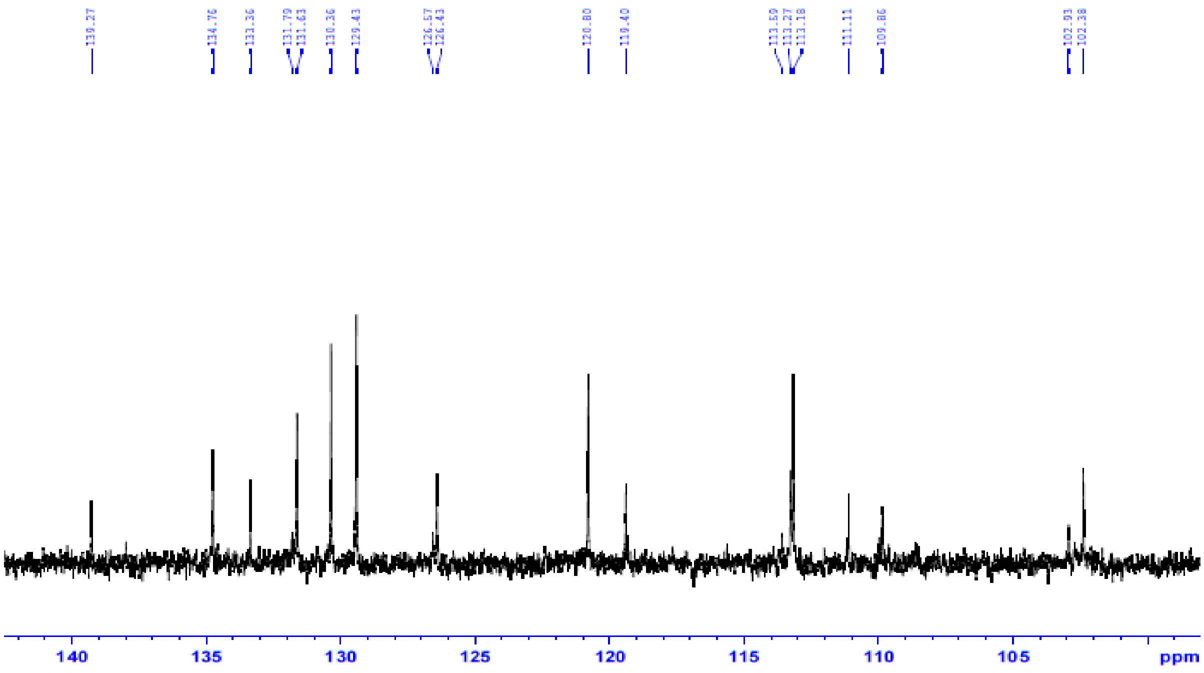

139.27
134.76
133.36
131.79
131.63
130.36
129.43
126.57
126.43
120.80
119.40
113.59
113.27
113.18
111.11
109.86
102.93
102.38
140
135
130
125
120
115
110
105
ppm

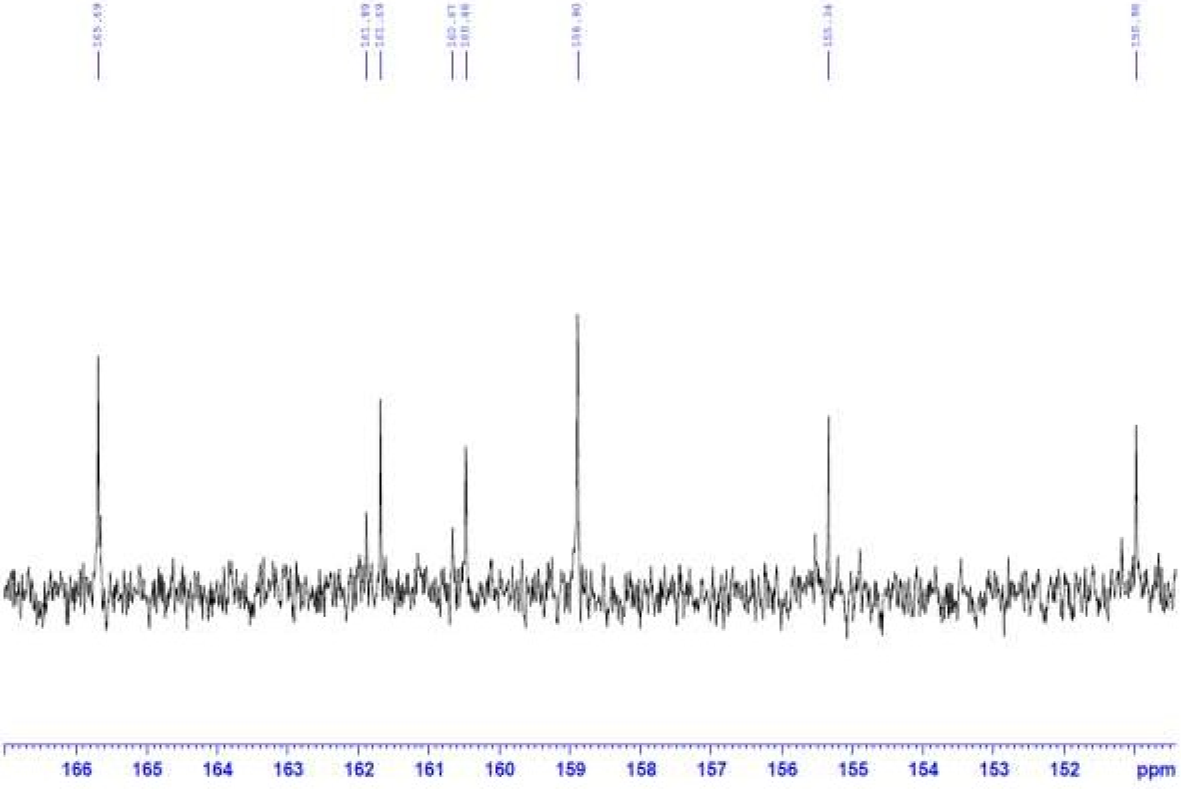

166
165
164
163
162
161
160
159
158
157
156
155
154
153
152
ppm

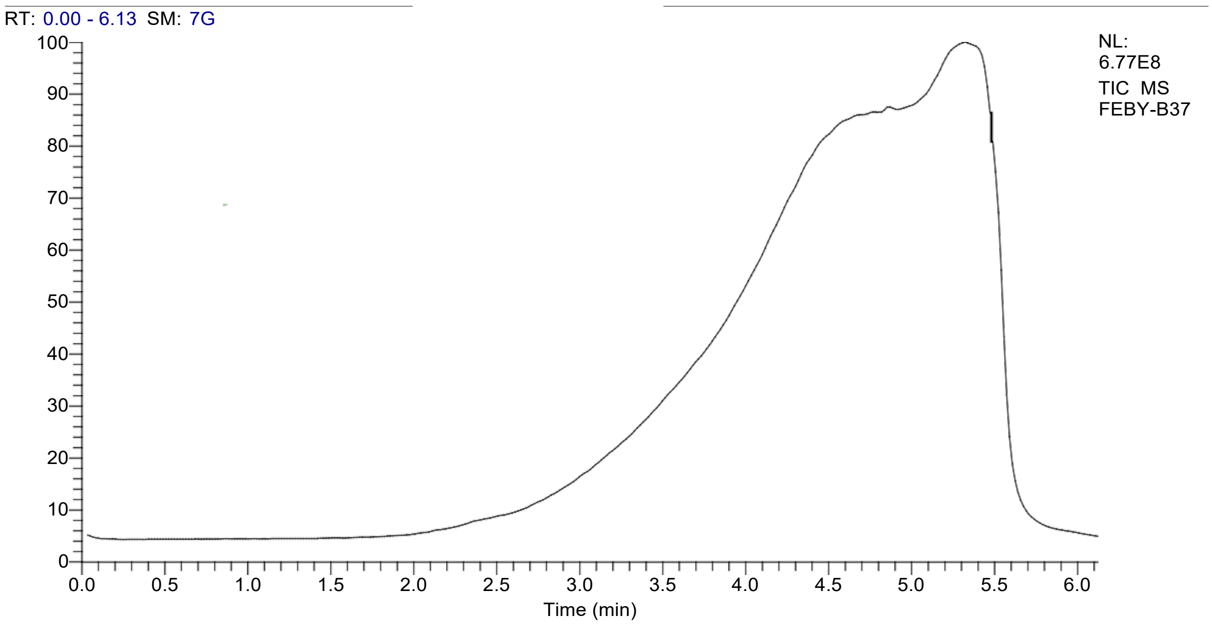
RT: 0.00 - 6.13 SM: 7G
NL:
6.77E8
TIC MS
FEBY-B37
Time (min)

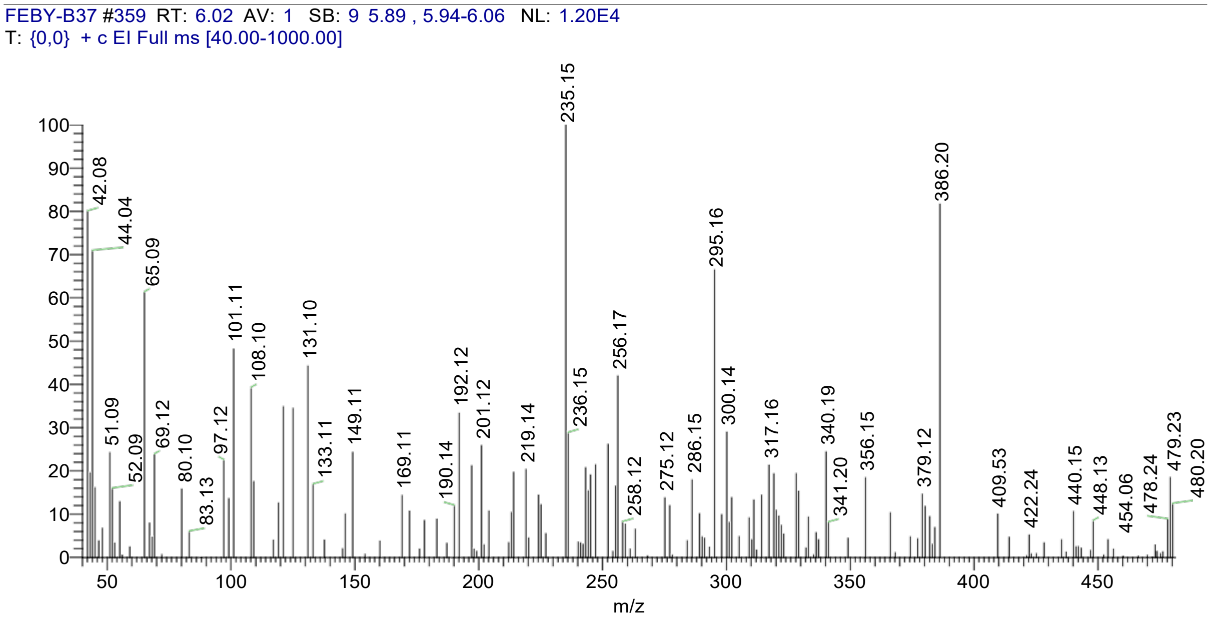
FEBY-B37 #359 RT: 6.02 AV: 1 SB: 9 5.89 , 5.94-6.06 NL: 1.20E4
T: {0,0} + c EI Full ms [40.00-1000.00]
235.15
386.20
295.16
42.08
44.04
65.09
m/z

Compound 13

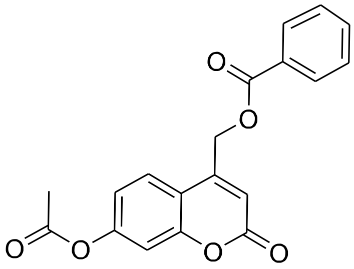

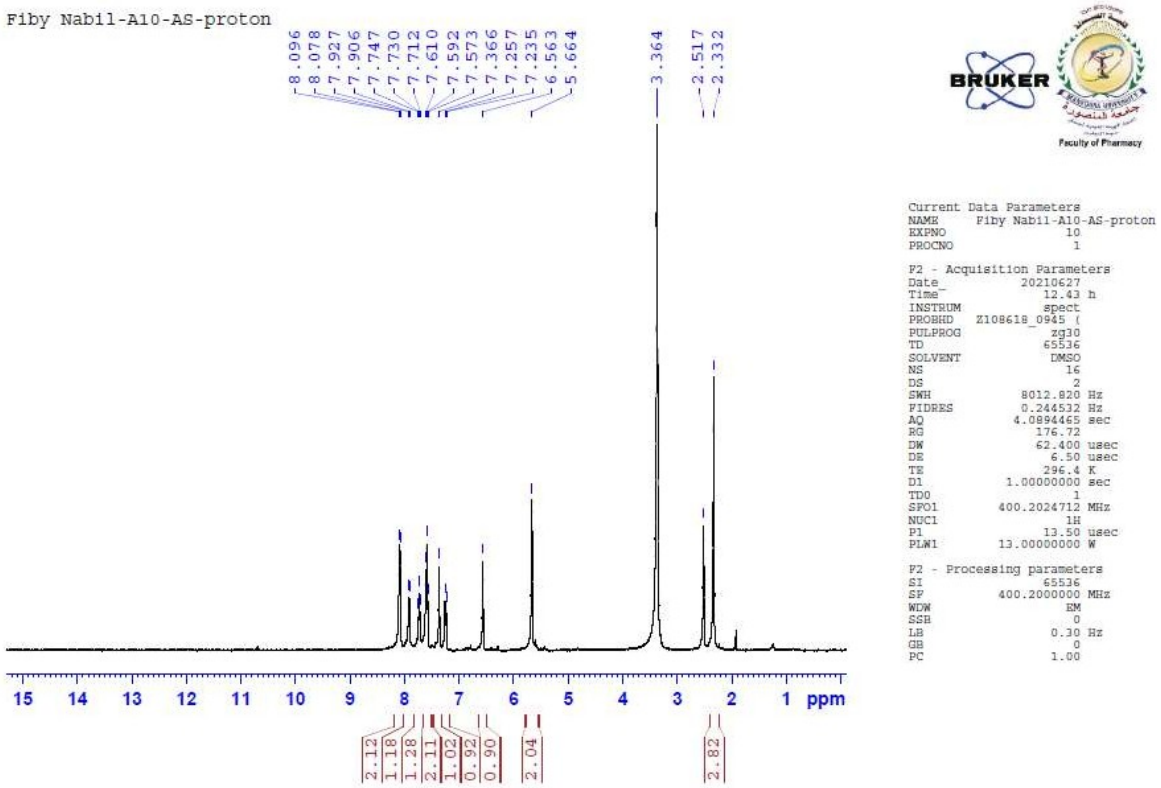

Fiby Nabil-A10-AS-proton
8.096
8.078
7.927
7.906
7.747
7.730
7.712
7.610
7.592
7.573
7.366
7.257
7.235
6.563
5.664
3.364
2.517
2.332
15 14 13 12 11 10 9 8 7 6 5 4 3 2 1 ppm
2.12
1.18
1.28
2.11
1.02
0.92
0.90
2.04
2.82
BRUKER
Faculty of Pharmacy
Current Data Parameters
NAME Fiby Nabil-A10-AS-proton
EXPNO 10
PROCNO 1
F2 - Acquisition Parameters
Date_ 20210627
Time 12.43 h
INSTRUM spect
PROBHD Z108618_0945 (
PULPROG zg30
TD 65536
SOLVENT DMSO
NS 16
DS 2
SWH 8012.820 Hz
FIDRES 0.244532 Hz
AQ 4.0894465 sec
RG 176.72
DW 62.400 usec
DE 6.50 usec
TE 296.4 K
D1 1.00000000 sec
TD0 1
SFO1 400.2024712 MHz
NUC1 1H
P1 13.50 usec
PLW1 13.00000000 W
F2 - Processing parameters
SI 65536
SF 400.2000000 MHz
WDW EM
SSB 0
LB 0.30 Hz
GB 0
PC 1.00

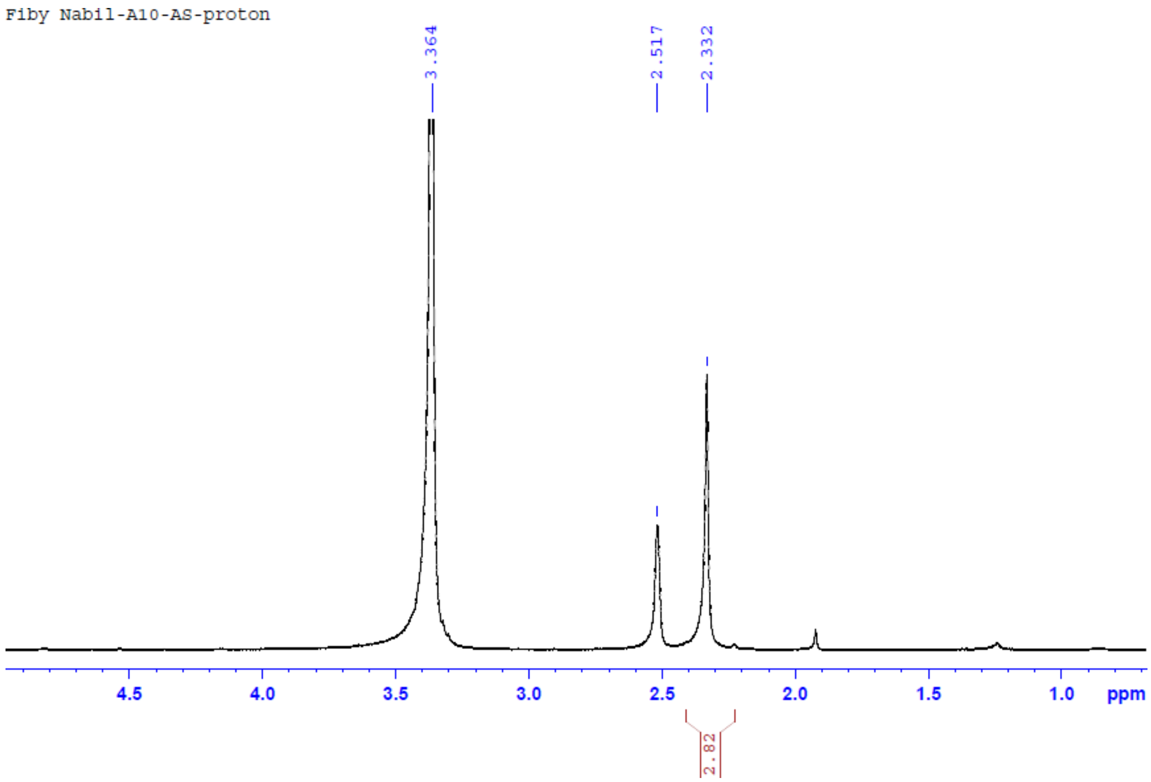
Fiby Nabil-A10-AS-proton
3.364
2.517
2.332
4.5
4.0
3.5
3.0
2.5
2.0
1.5
1.0
ppm
2.82

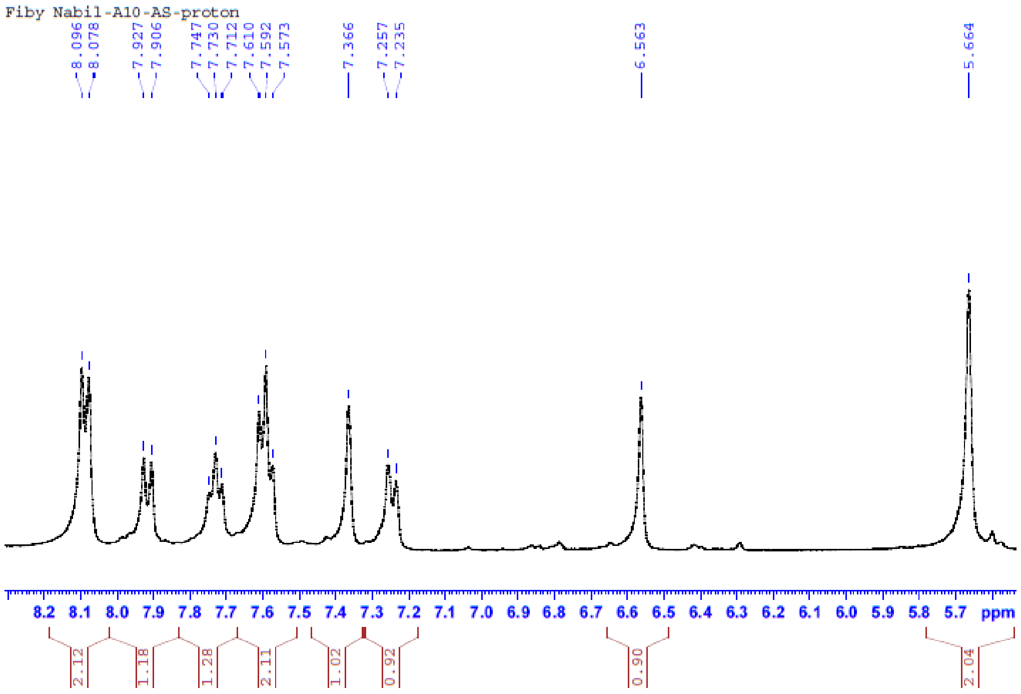
Fiby Nabil-A10-AS-proton
8.096
8.078
7.927
7.906
7.747
7.730
7.712
7.610
7.592
7.573
7.366
7.257
7.235
6.563
5.664
8.2 8.1 8.0 7.9 7.8 7.7 7.6 7.5 7.4 7.3 7.2 7.1 7.0 6.9 6.8 6.7 6.6 6.5 6.4 6.3 6.2 6.1 6.0 5.9 5.8 5.7 ppm
2.12
1.18
1.28
2.11
1.02
0.92
0.90
2.04

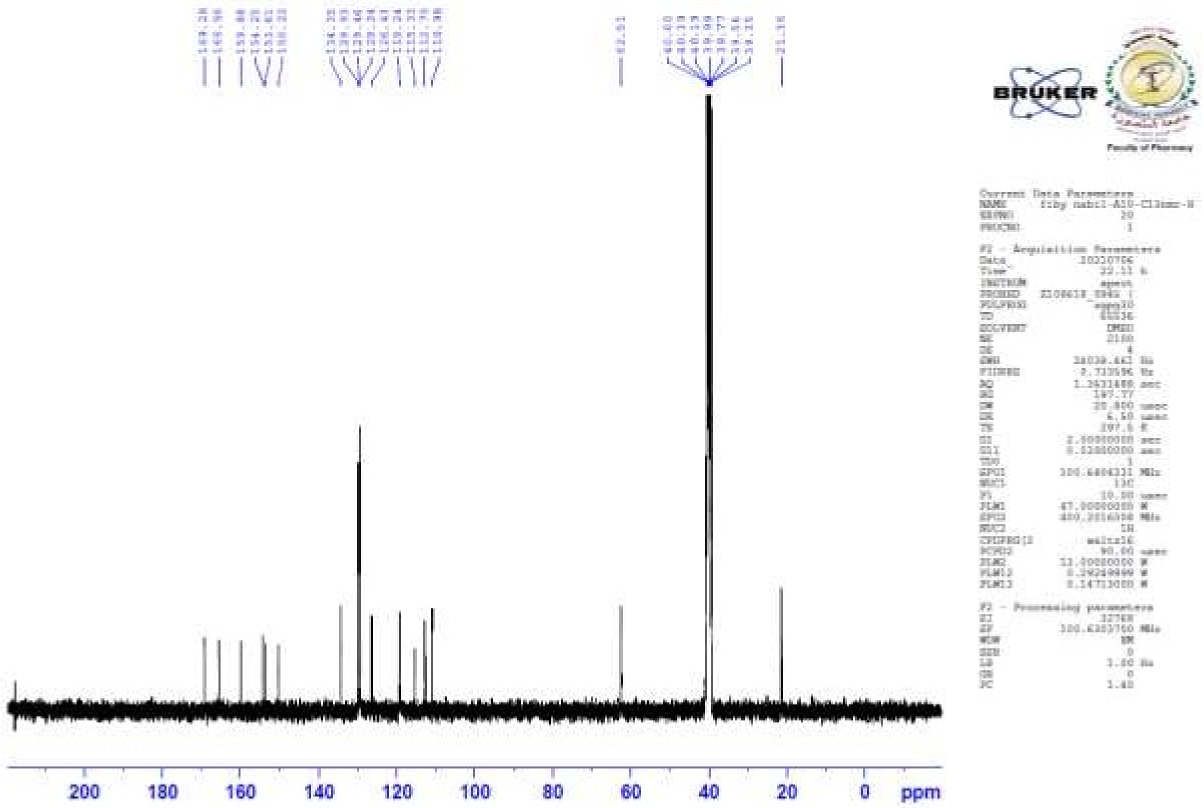

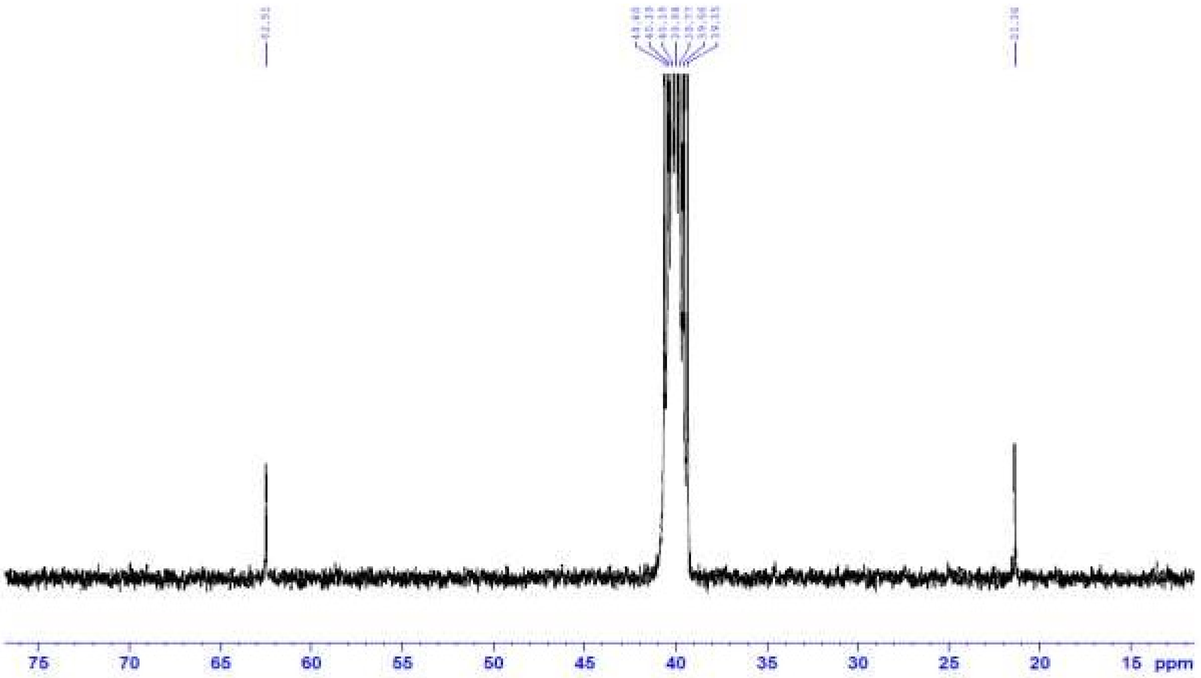

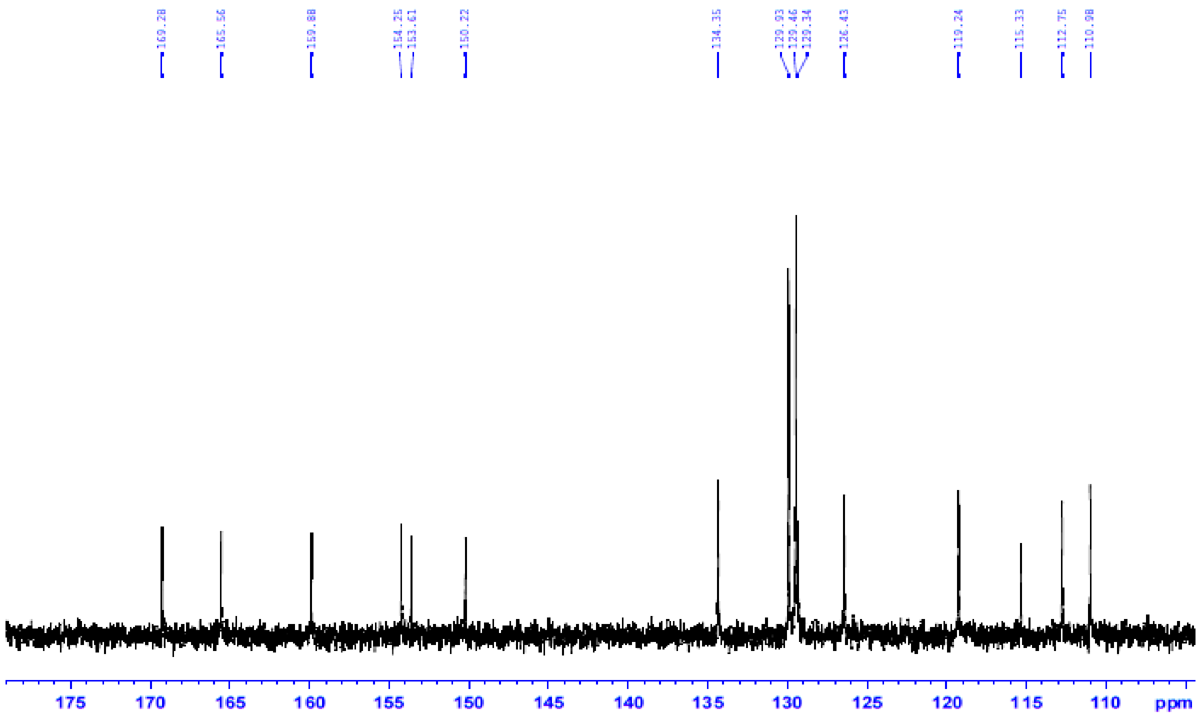
169.28
165.56
159.88
154.25
153.61
150.22
134.35
129.93
129.46
129.34
126.43
119.24
115.33
112.75
110.98
175
170
165
160
155
150
145
140
135
130
125
120
115
110
ppm

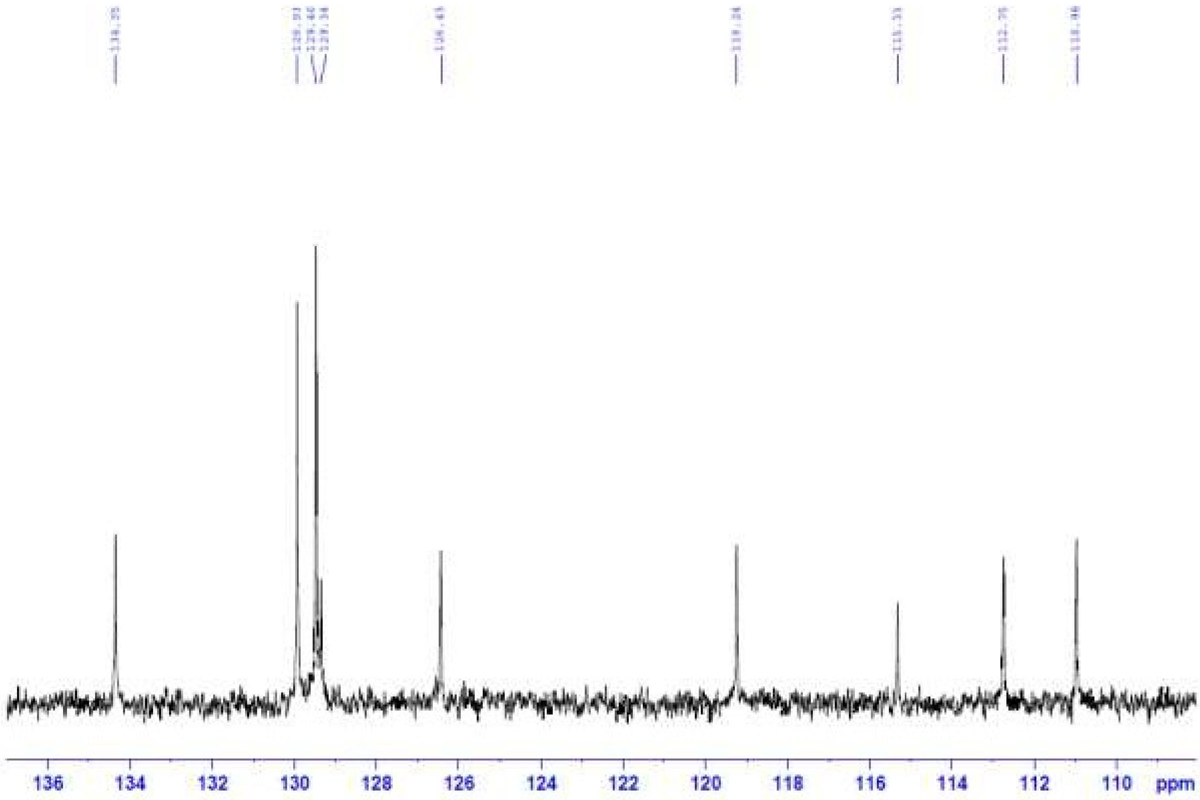
136
134
132
130
128
126
124
122
120
118
116
114
112
110
ppm

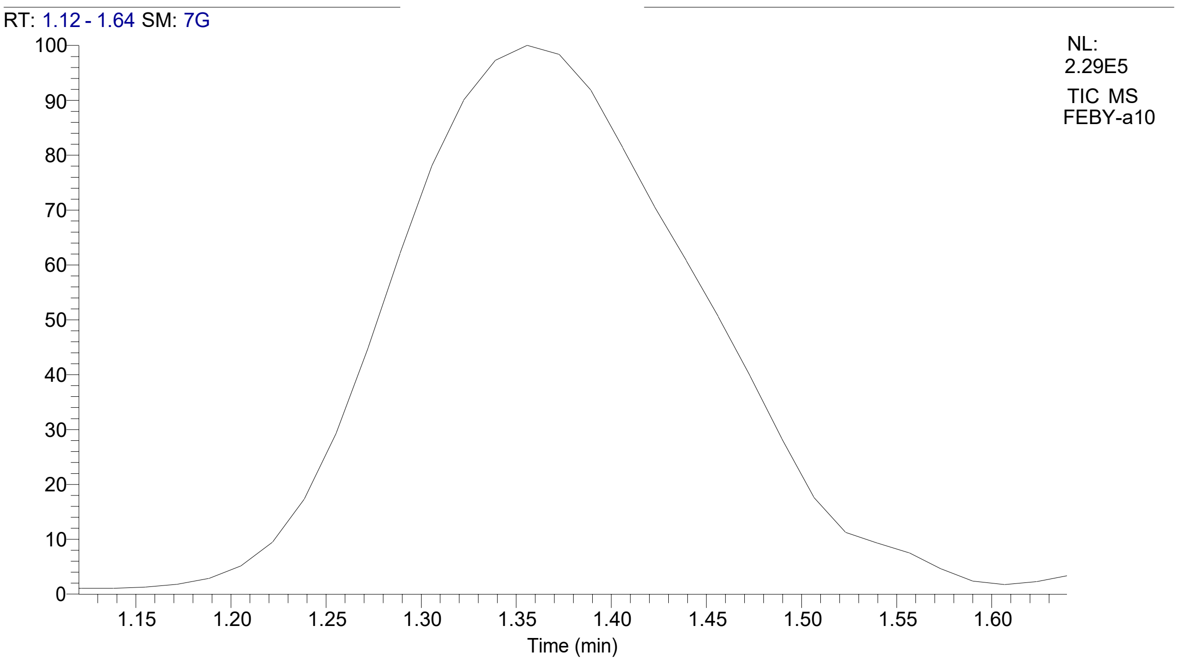
RT: 1.12 - 1.64 SM: 7G
NL: 2.29E5
TIC MS
FEBY-a10
Time (min)

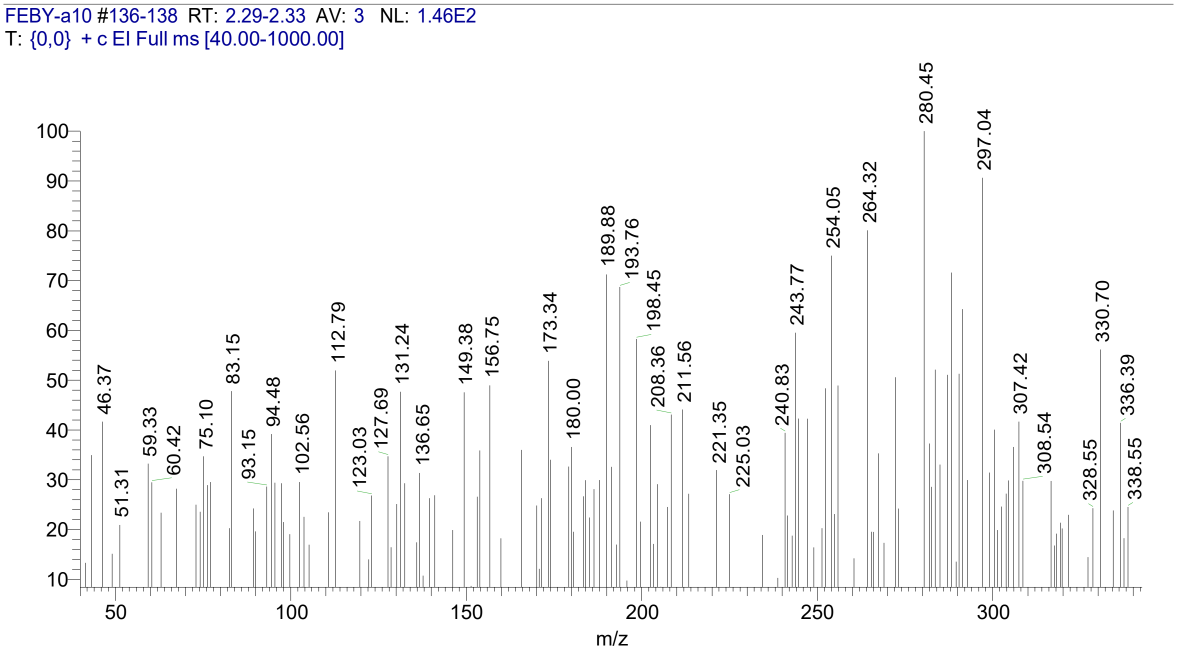
FEBY-a10 #136-138 RT: 2.29-2.33 AV: 3 NL: 1.46E2
T: {0,0} + c EI Full ms [40.00-1000.00]
m/z

Compound 14

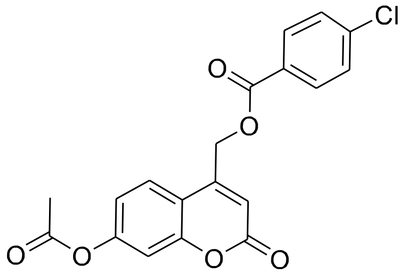
Cl
O
O
O
O
O
O

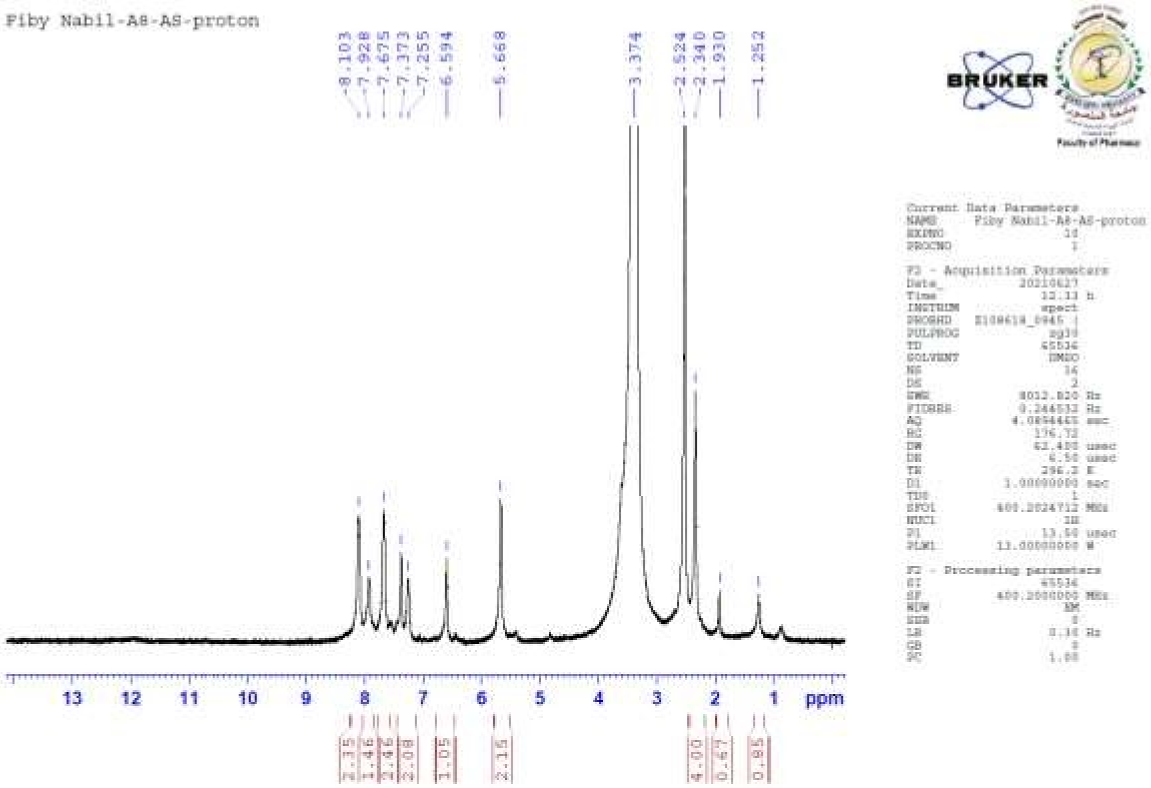
Fiby Nabil-A8-AS-proton
8.103
7.928
7.675
7.373
7.255
6.594
5.668
3.374
2.524
2.340
1.930
1.252
13
12
11
10
9
8
7
6
5
4
3
2
1
ppm
2.35
1.46
2.46
2.08
1.05
2.15
4.00
0.67
0.85

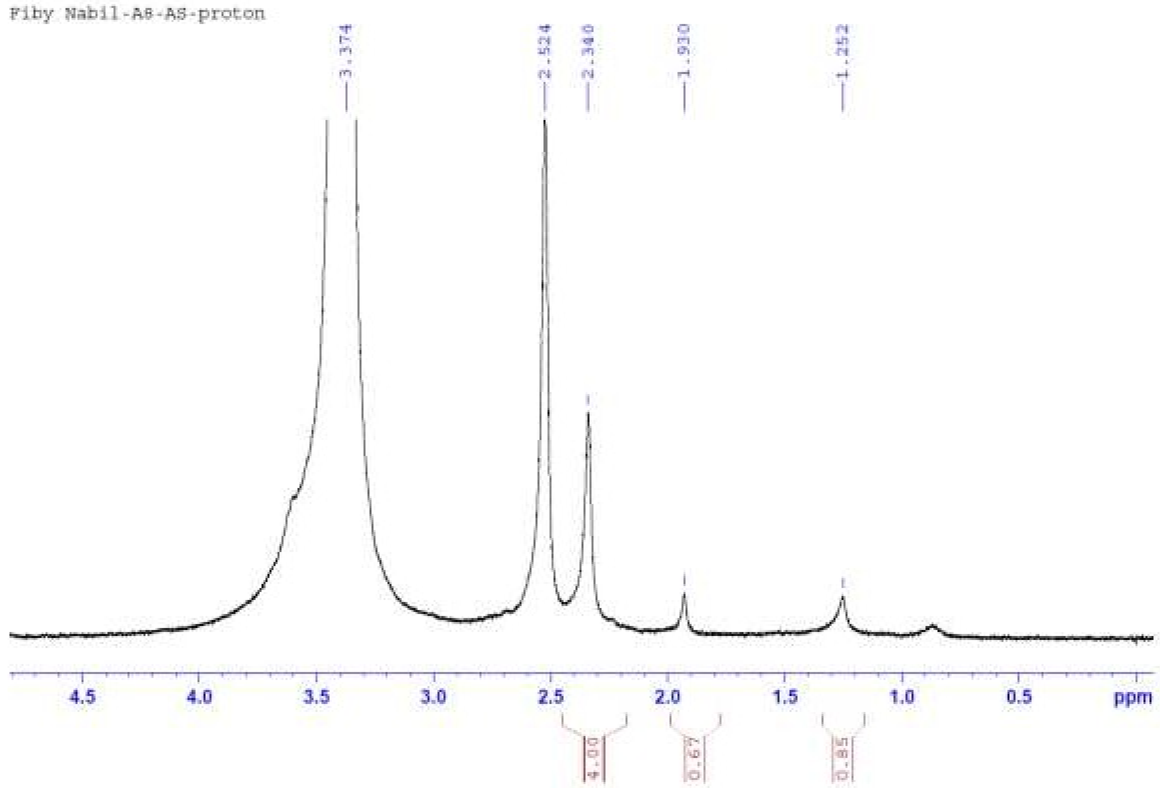
Fiby Nabil-A8-AS-proton
3.374
2.524
2.340
1.930
1.252
4.5
4.0
3.5
3.0
2.5
2.0
1.5
1.0
0.5
ppm
4.00
0.67
0.85

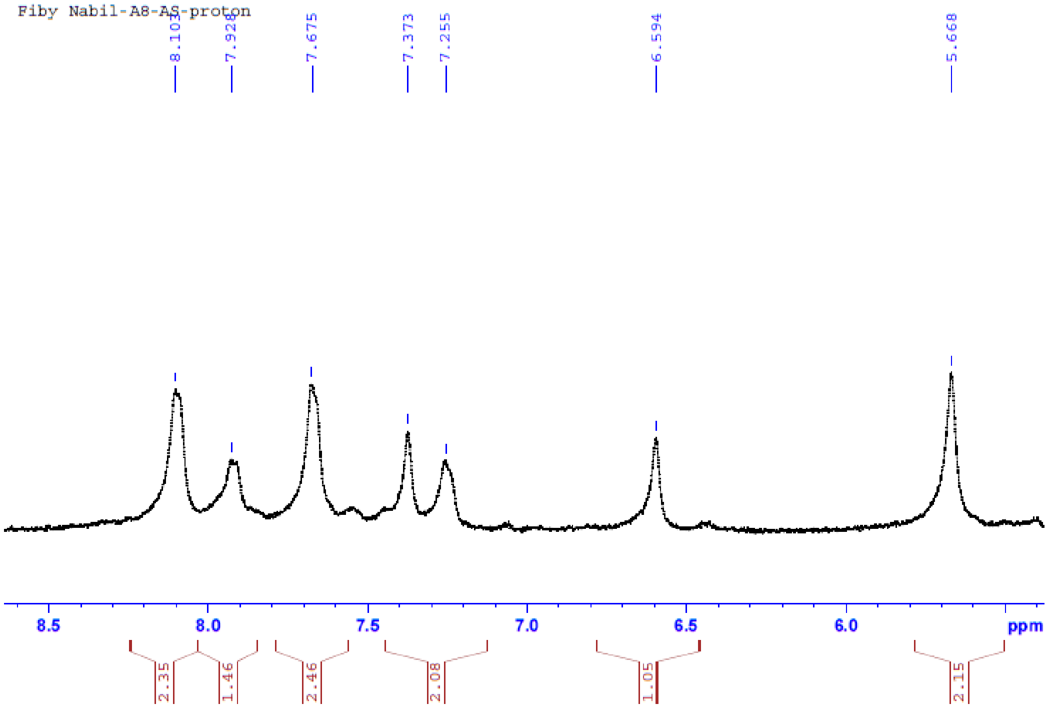
Fiby Nabil-A8-AS-proton
8.103
7.928
7.675
7.373
7.255
6.594
5.668
8.5
8.0
7.5
7.0
6.5
6.0
ppm
2.35
1.46
2.46
2.08
1.05
2.15

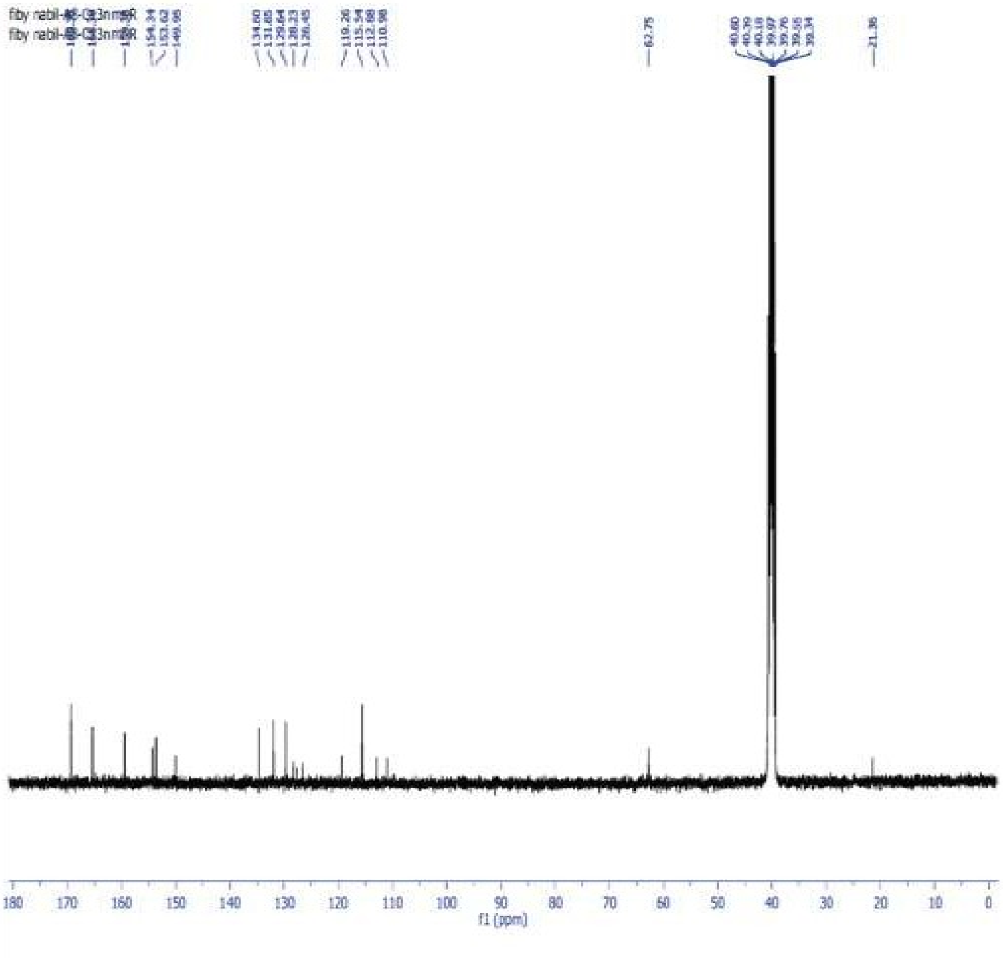


```
Current Data Parameters
NAME      fiby nabil-A10-C13nmr-R
EXPNO                  20
PROCNO                  1

F2 - Acquisition Parameters
Date_            20210706
Time                22.11 h
INSTRUM             spect
PROBHD   Z108618_0945 (
PULPROG            zgpg30
TD                  65536
SOLVENT              DMSO
NS                   2100
DS                      4
SWH             24038.461 Hz
FIDRES           0.733596 Hz
AQ              1.3631488 sec
RG                 197.77
DW                 20.800 usec
DE                   6.50 usec
TE                  297.5 K
D1             2.00000000 sec
D11            0.03000000 sec
TD0                     1
SFO1          100.6404331 MHz
NUC1                  13C
P1                  10.00 usec
PLW1          47.00000000 W
SFO2          400.2016008 MHz
NUC2                   1H
CPDPRG[2          waltz16
PCPD2               90.00 usec
PLW2          13.00000000 W
PLW12          0.29249999 W
PLW13          0.14713000 W

F2 - Processing parameters
SI                  32768
SF            100.6303700 MHz
WDW                    EM
SSB                     0
LB                   1.00 Hz
GB                      0
PC                   1.40
```

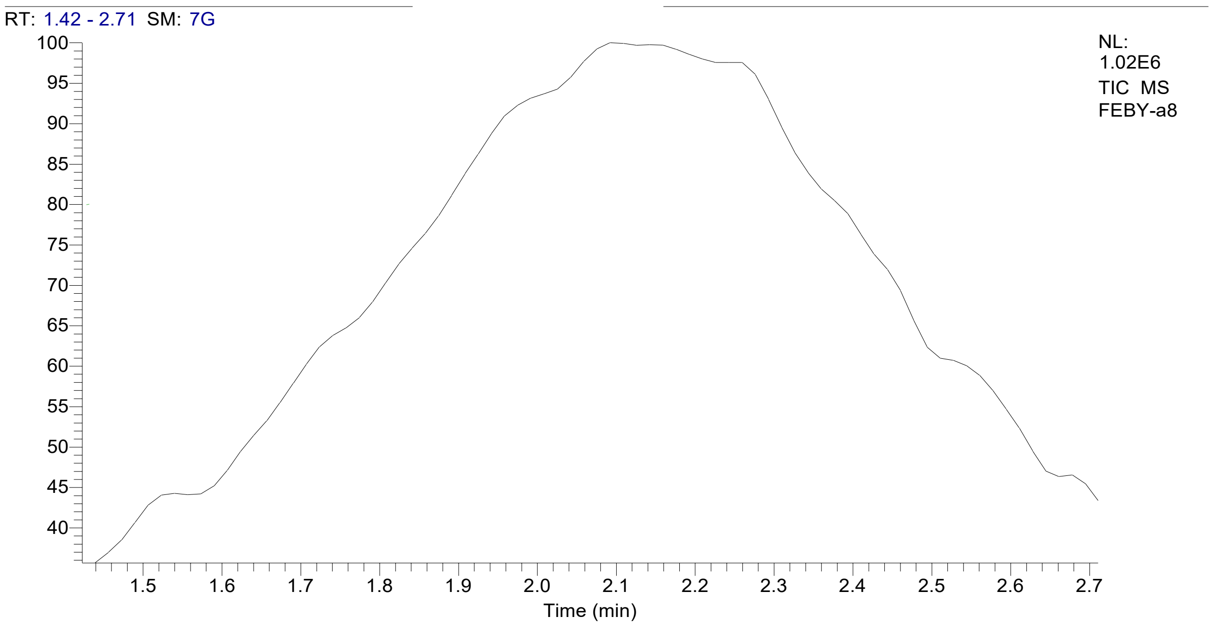
RT: 1.42 - 2.71 SM: 7G
NL: 1.02E6
TIC MS
FEBY-a8
Time (min)

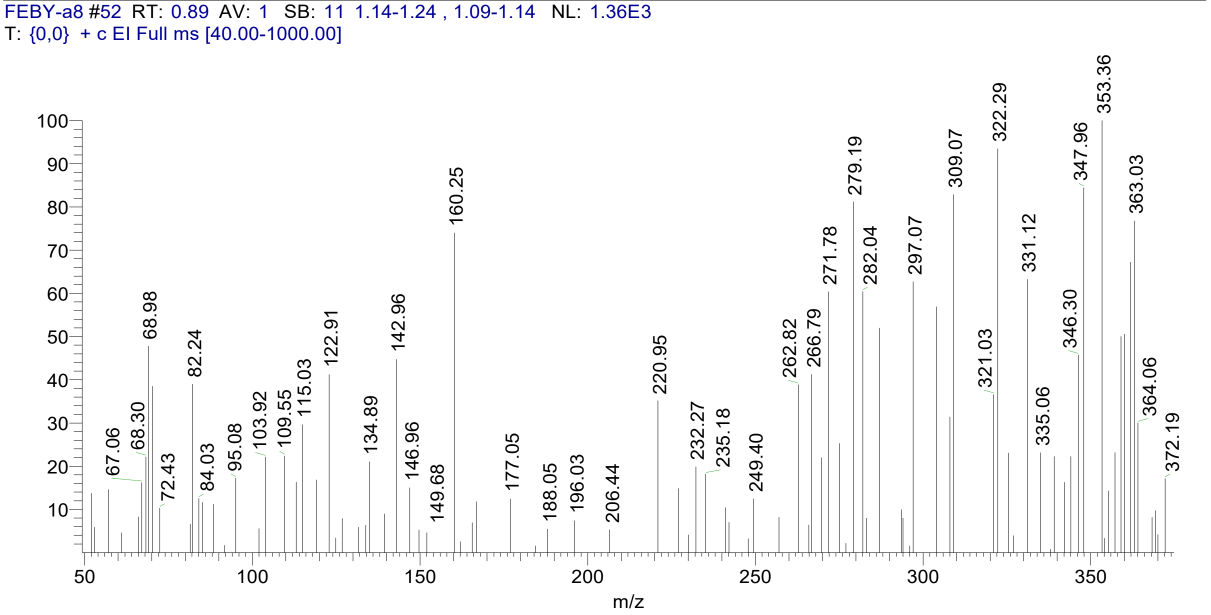
FEBY-a8 #52 RT: 0.89 AV: 1 SB: 11 1.14-1.24 , 1.09-1.14 NL: 1.36E3
T: {0,0} + c EI Full ms [40.00-1000.00]
353.36
322.29
309.07
279.19
347.96
363.03
160.25
331.12
271.78
282.04
297.07
68.98
82.24
142.96
122.91
115.03
346.30
321.03
220.95
262.82
266.79
364.06
335.06
372.19
109.55
103.92
95.08
84.03
72.43
68.30
67.06
134.89
146.96
149.68
177.05
188.05
196.03
206.44
232.27
235.18
249.40
m/z

Compound 15

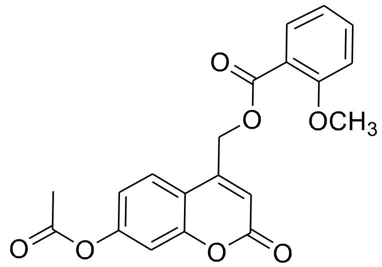

O
O
OCH3
O
O
O
O

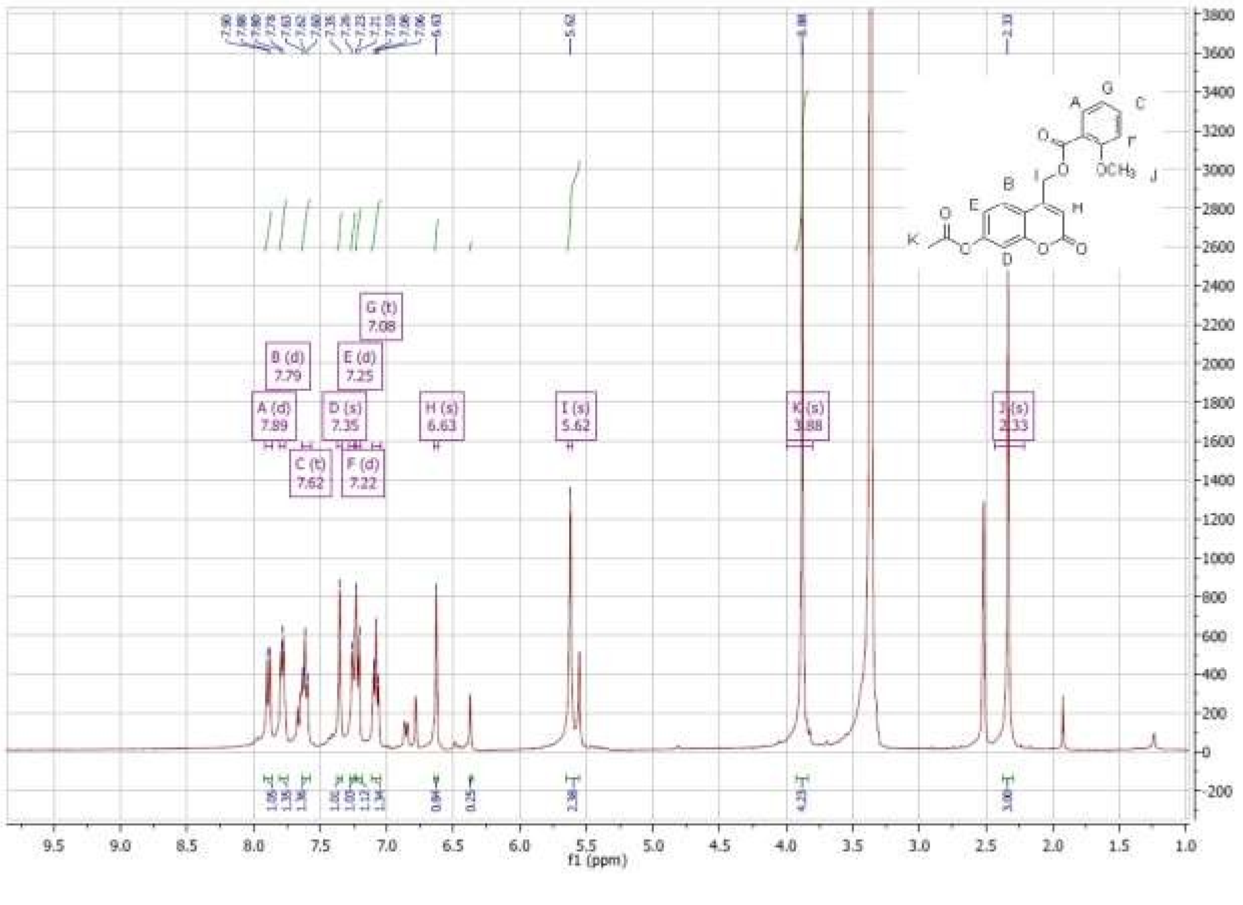

G (t)
7.08
B (d)
7.79
E (d)
7.25
A (d)
7.89
D (s)
7.35
H (s)
6.63
I (s)
5.62
K (s)
3.88
J (s)
2.33
C (t)
7.62
F (d)
7.22
f1 (ppm)

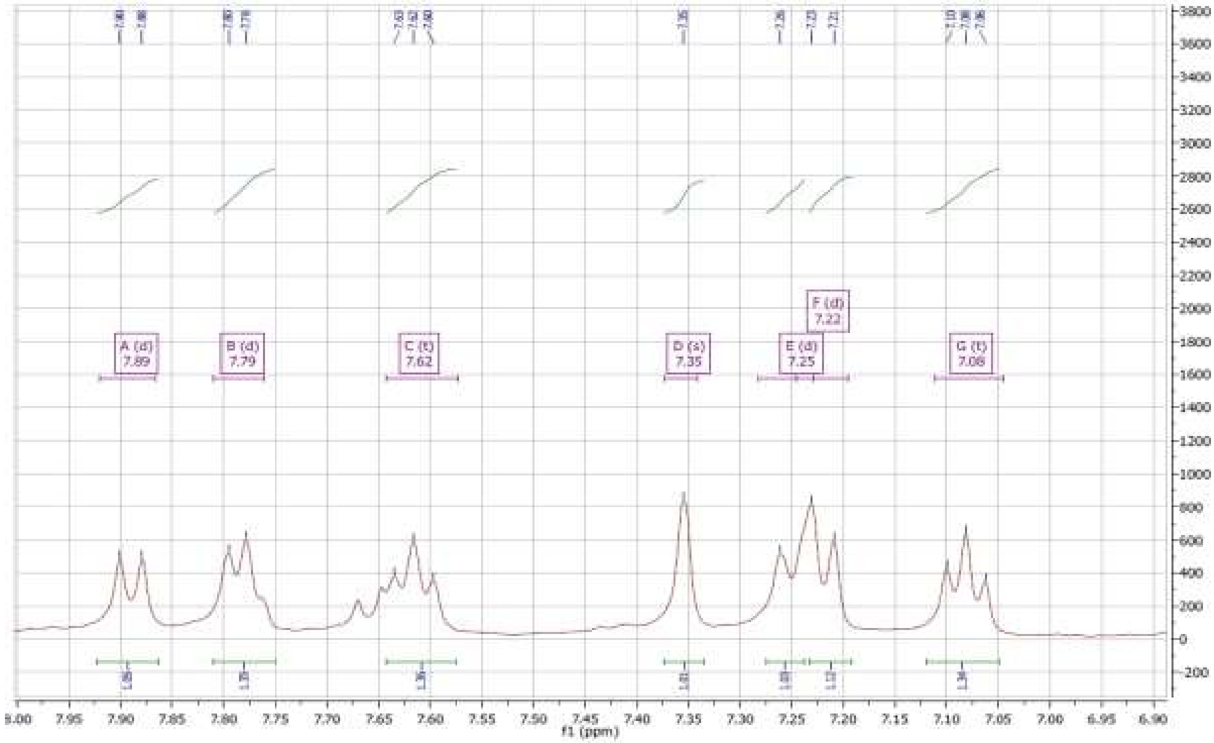
A (d)
7.89
B (d)
7.79
C (t)
7.62
D (s)
7.35
E (d)
7.25
F (d)
7.22
G (t)
7.08
f1 (ppm)

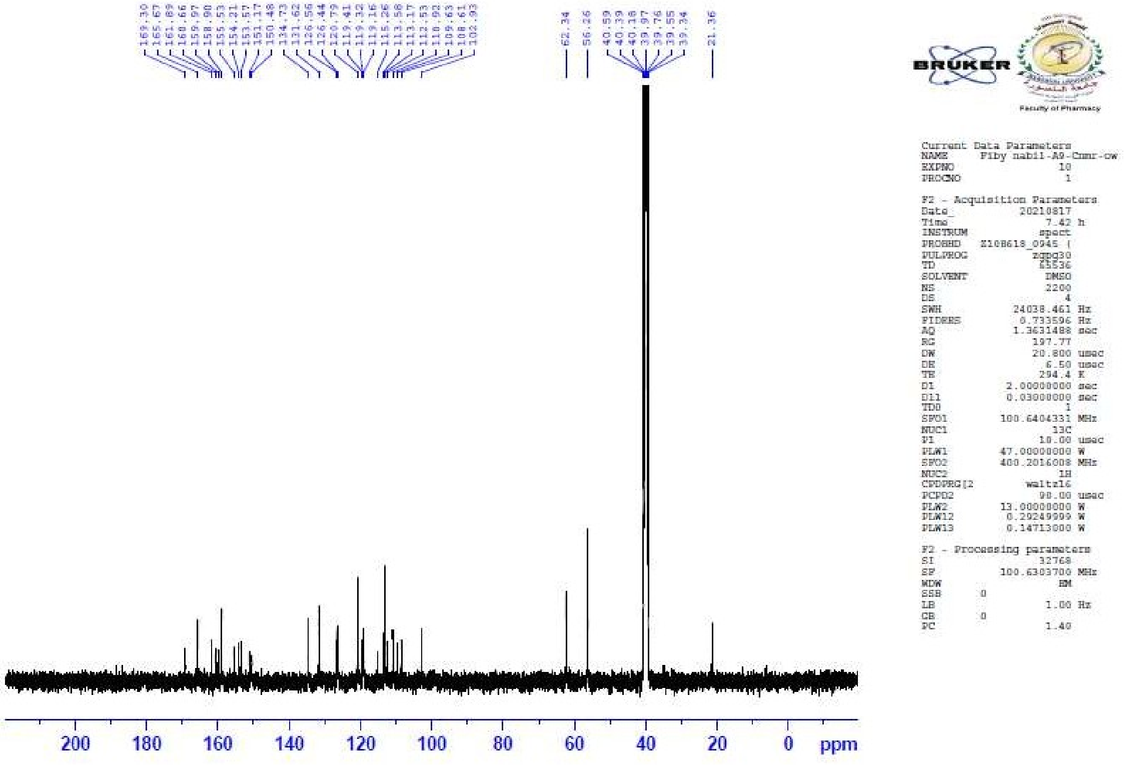
169.30
165.67
161.89
160.66
159.97
158.90
155.53
154.21
151.57
151.17
150.48
134.73
131.62
126.56
126.44
120.79
119.41
119.32
119.16
115.26
113.58
113.17
112.53
110.92
109.63
108.61
102.93
62.34
56.26
40.59
40.39
40.18
39.97
39.76
39.55
39.34
21.36
ppm
Current Data Parameters
NAME Fiby nabil-A9-Cmnr-ow
EXPNO 10
PROCNO 1
F2 - Acquisition Parameters
Date_ 20210817
Time 7.42 h
INSTRUM spect
PROBHD Z108618_0945 (
PULPROG zgpg30
TD 65536
SOLVENT DMSO
NS 2200
DS 4
SWH 24038.461 Hz
FIDRES 0.733596 Hz
AQ 1.3631488 sec
RG 197.77
DW 20.800 usec
DE 6.50 usec
TE 294.4 K
D1 2.00000000 sec
D11 0.03000000 sec
TD0 1
SFO1 100.6404331 MHz
NUC1 13C
P1 10.00 usec
PLW1 47.00000000 W
SFO2 400.2016008 MHz
NUC2 1H
CPDPRG[2 waltz16
PCPD2 90.00 usec
PLW2 13.00000000 W
PLW12 0.29249999 W
PLW13 0.14713000 W
F2 - Processing parameters
SI 32768
SF 100.6303700 MHz
WDW EM
SSB 0
LB 1.00 Hz
GB 0
PC 1.40

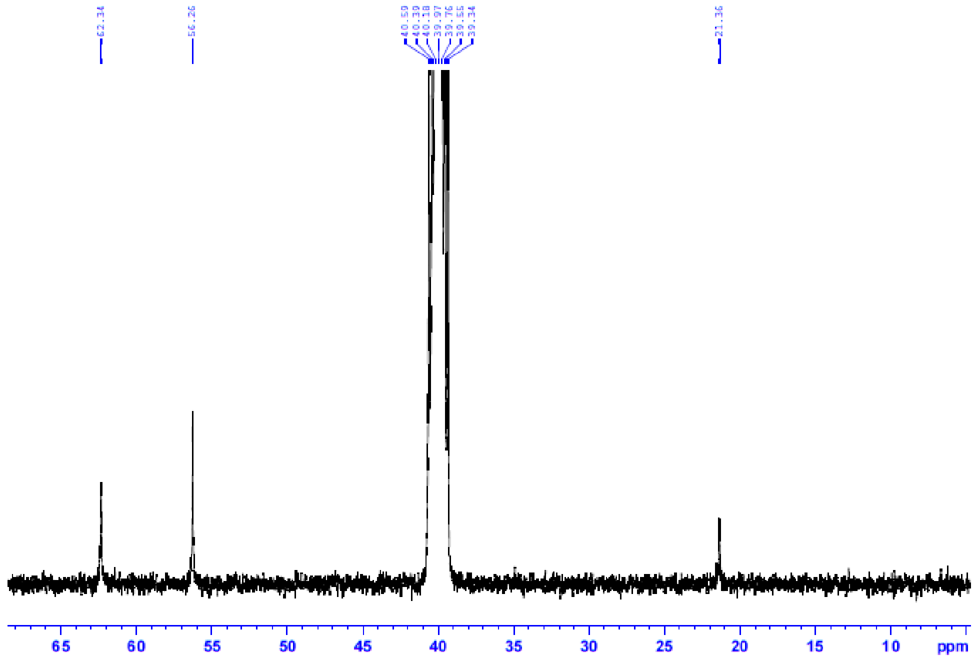
62.34
56.26
40.59
40.39
40.18
39.97
39.76
39.55
39.34
21.36
65
60
55
50
45
40
35
30
25
20
15
10
ppm

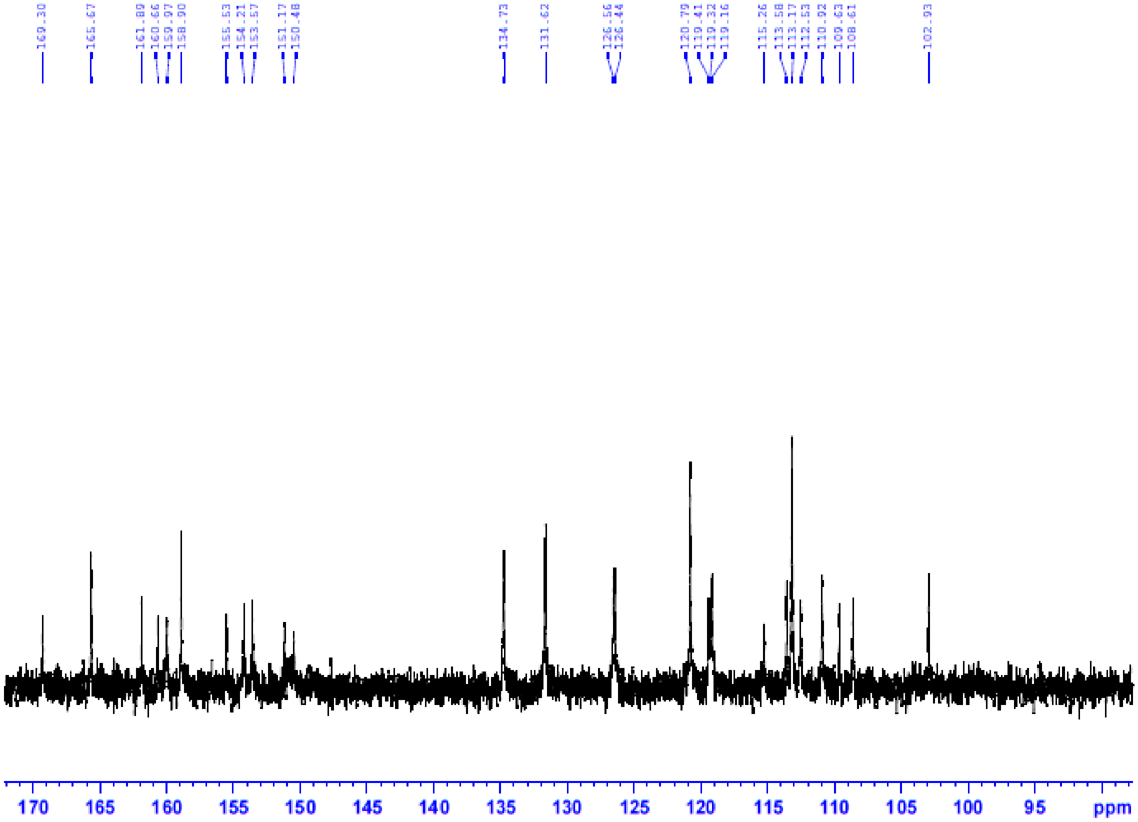
169.30
165.67
161.89
160.66
159.97
158.90
155.53
154.21
153.57
151.17
150.48
134.73
131.62
126.56
126.44
120.79
119.41
119.32
119.16
115.26
113.58
113.17
112.53
110.92
109.63
108.61
102.93
170
165
160
155
150
145
140
135
130
125
120
115
110
105
100
95
ppm

RT: 5.50 - 5.91 SM: 7G

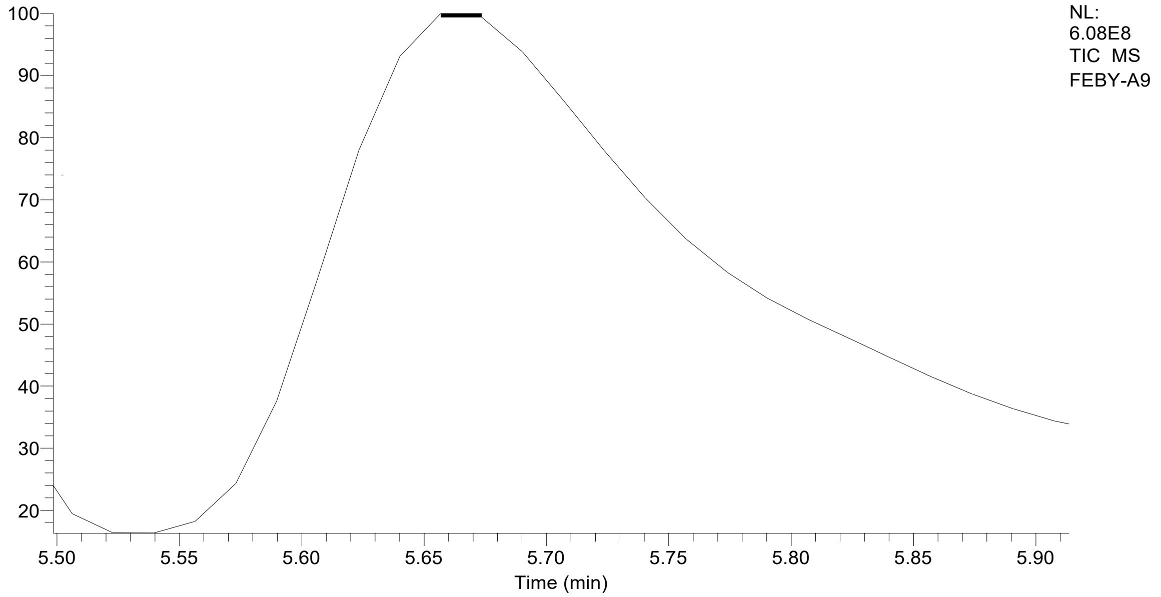

FEBY-A9 #310 RT: 5.20 AV: 1 SB: 18 5.22 , 5.02-5.29 NL: 5.25E4
T: {0,0} + c EI Full ms [40.00-1000.00]

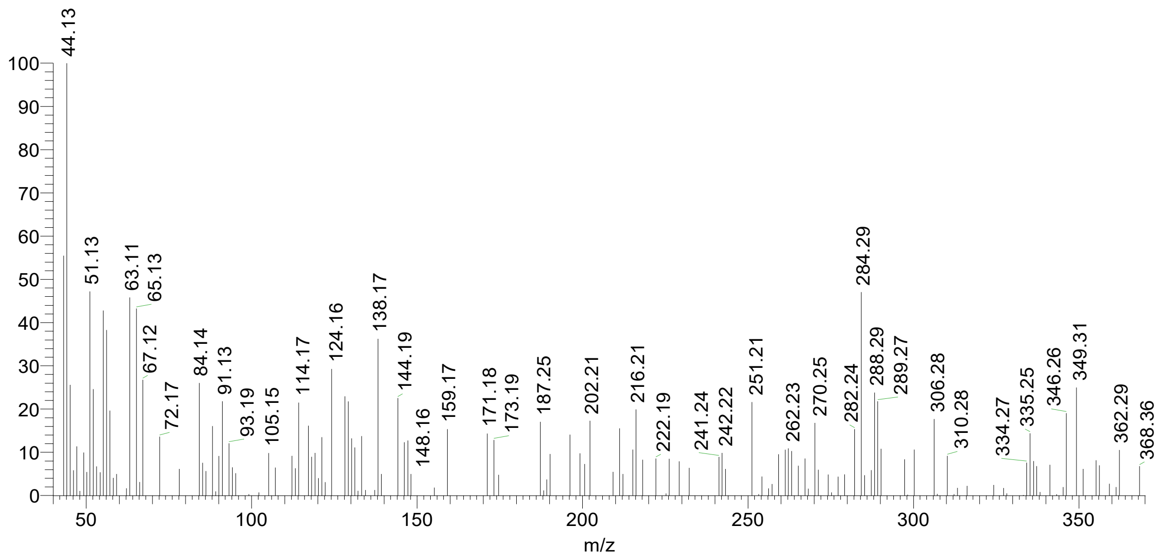

Supplement: Supplementary file 2 — Supplementary Information 2. [file 41598_2023_40232_MOESM2_ESM.pdf]
